# Highly Regioselective Synthesis of Substituted Isoindolinones *via* Ruthenium-Catalyzed Alkyne Cyclotrimerizations

**DOI:** 10.1002/adsc.201300055

**Published:** 2013-08-12

**Authors:** Robert W Foster, Christopher J Tame, Helen C Hailes, Tom D Sheppard

**Affiliations:** a Department of Chemistry, University College London, Christopher Ingold LaboratoriesLondon, WC1H 0AJ, U.K. Fax: (+44)-(0)20-7679-7463; phone: (+44)-(0)20-7679-2467; b GlaxoSmithKline, Medicines Research CentreGunnels Wood Road, Stevenage, Hertfordshire, SG1 2NY, U.K.

**Keywords:** alkynes, amide tether, cyclotrimerization, isoindolinones, ruthenium, trimethylsilyl group

## Abstract

(Cyclooctadiene)(pentamethylcyclopentadiene)ruthenium chloride [Cp*RuCl(cod)] has been used to catalyze the regioselective cyclization of amide-tethered diynes with monosubstituted alkynes to give polysubstituted isoindolinones. Notably, the presence of a trimethylsilyl group on the diyne generally led to complete control over the regioselectivity of the alkyne cyclotrimerization. The cyclization reaction worked well in a sustainable non-chlorinated solvent and was tolerant of moisture. The optimized conditions were effective with a diverse range of alkynes and diynes. The 7-silylisoindolinone products could be halogenated, protodesilylated or ring opened to access a range of usefully functionalized products.

## Introduction

Substituted isoindolinones have recently generated considerable interest because of their diverse biological activities, including the inhibition of angiogenesis,^1^ tumour necrosis factor production,^2^ MDM2-p53 protein-protein interactions,^3^ hypoxia-inducible factor-1α^4^ and histone deacetylase.^5^ The majority of existing protocols for isoindolinone synthesis require the construction of a γ-lactam adjacent to a pre-formed aromatic core.^6^ Recent examples include the one-pot transformation of 2-halobenzaldimines into chiral 3-substituted isoindolinones and the Ni-mediated cyclization of *N*-benzoyl aminals in the presence of a stoichiometric Lewis acid.^7^,^8^ However, the inevitable limitation of these approaches is the accessibility of the arene starting material itself. The synthesis of polysubstituted arenes is often non-trivial, frequently requiring numerous steps, the use of protecting group strategies and/or functional group interconversions.

The transition metal-catalyzed [2+2+2] cyclotrimerization of alkynes is emerging as an elegant, atom efficient and convergent approach to the synthesis of highly substituted arenes.^9^ The strategy allows for the regioselective synthesis of compounds that would be extremely difficult to make *via* traditional aromatic chemistry. The regioselectivity of a cyclotrimerization is normally controlled by tethering two or three of the alkyne components together, so this strategy is best suited to the synthesis of bicyclic and tricyclic ring systems. This allows for the assembly of substituted multiple-ring aromatic compounds from alkyne precursors in a single step.

**Scheme 1:**
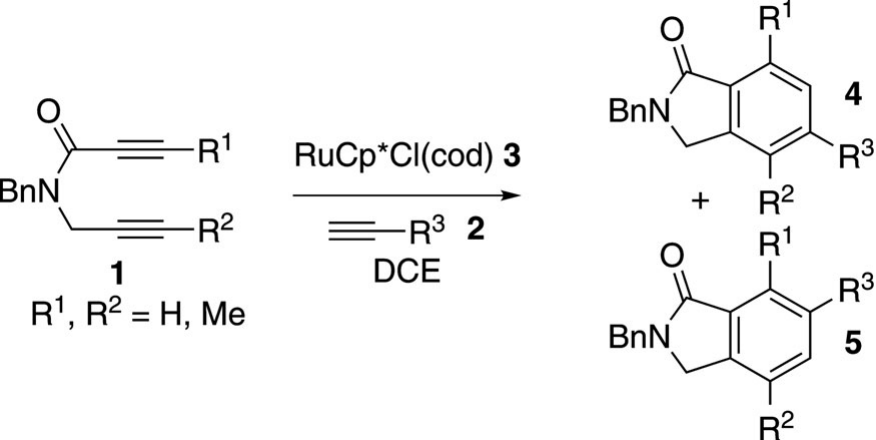
Isoindolinone synthesis as reported by Yamamoto and co-workers.^10^

Yamamoto and co-workers have previously recognized the potential of alkyne cyclotrimerizations for the synthesis of isoindolinones bearing substituents on the aromatic ring.^10^ They reported the cyclization of amide-tethered diynes **1** with monoynes **2** using Cp*RuCl(cod) **3** as the catalyst to give regioisomeric isoindolinones **4** and **5** (Scheme 1). In general the regioselectivity of the cyclotrimerization was poor to moderate, with the exception of a single example bearing a methyl group at R^1^. In addition, a significant limitation of this method is the use of 1,2-dichloroethane (DCE) as solvent, a substance which is potentially detrimental to human health and is generally avoided within industry.^11^

The aim of this study was to explore the regioselective synthesis of polysubstituted isoindolinones using more industrially viable reaction conditions, to establish the general applicability of the reaction, and to develop the synthetic potential of the cyclized products. On the basis of previously reported cyclizations we envisaged that the introduction of a trimethylsilyl group at R^1^ in diyne **1** would direct the regioselectivity of the cyclisation reaction effectively with a broad range of monoynes.^10^,^12^ The arylsilane unit present in the isoindolinone product could then be transformed using standard chemical techniques to access a variety of 7-substituted derivatives.

## Results and Discussion

### Diyne Synthesis

**Scheme 2:**
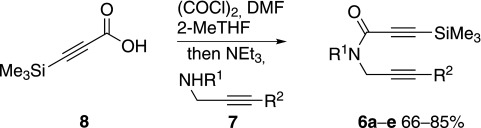
Synthesis of diynes 6a–e.

Initially several amide-tethered diynes **6** were prepared by the coupling of propargylic amines **7** with 3-(trimethylsilyl)propiolic acid **8**, *via* the corresponding acid chloride (Scheme 2).^13^ Where necessary the corresponding amines were prepared using literature procedures.^14^–^15^

### Optimization

Various conditions were screened for the cyclotrimerization of diyne **6a** with 1-hexyne **9a** to form isoindolinone **10a**, and the results are summarized in Table 1. All reactions were conducted for 16 h at which point conversion and selectivity were determined by analysis of the crude ^1^H NMR spectrum.

**Table 1:** Optimization of the cyclotrimerization of 6a and 9a.
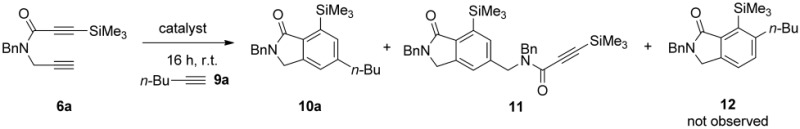

Entry	Solvent	Equivalents of 9a	Catalyst	Catalyst loading [mol%]	Conversion[a,b] [%]	Ratio10a:11^a^
1	PhMe^c^	4	RhCl(PPh_3_)_3_	5	<5	–
2	PhMe^c^	4	Co_2_(CO)_8_	10	<5	–
3	CH_2_Cl_2_^c^	4	Grubbs I	5	5	n.d.
4	DCE^c^	4	Cp^*^RuCl(cod)	1	5	n.d.
5	neat^d^	4	Cp^*^RuCl(cod)	1	50	3:2
6	neat^d^	4	Cp^*^RuCl(cod)	3	100	3:1
7	CPME	4	Cp^*^RuCl(cod)	3	100	5:1
8	CPME	4	Cp^*^RuCl(cod)	1	60	4:1
9	CPME	2	Cp^*^RuCl(cod)	3	100	2:1
10^e^	CPME	4	Cp^*^RuCl(cod)	3	100	8:1
**11**^e^	**CPME**	**2**	Cp^*^RuCl(cod)	**3**	**100**	**9:1**
12^e^	CPME	1.1	Cp^*^RuCl(cod)	3	100	5:2
13^e^	MTBE	2	Cp^*^RuCl(cod)	3	100	5:1
14^e^	2-MeTHF	2	Cp^*^RuCl(cod)	3	90	5:1
15^e^	CPME/10% water	2	Cp^*^RuCl(cod)	3	70	3:1
16	water	4	Cp^*^RuCl(cod)	3	30	3:1

[a] Determined by analysis of the crude ^1^H NMR spectrum.

[b] Conversion of **6a** into **10a** and **11** (determined by crude ^1^H NMR without the use of an internal standard).

c Solvent dried over activated 4 Å molecular sieves and degassed.

[d] Cp^*^RuCl(cod) **3** was added to the reaction mixture at 0 °C, which was then allowed to reach room temperature.

[e] Diyne **6a** in CPME was added dropwise over 3 h to a stirring solution of **9a** and **3** in CPME.

The cyclotrimerization of diyne **6a** and alkyne **9a** was examined using four different literature procedures. Neither RhCl(PPh_3_)_3_ nor Co_2_(CO)_8_ were effective in catalyzing the alkyne cyclotrimerization, with no measurable conversion of diyne **6a** (entries 1 and 2).^16^ Treating diyne **6a** with 5 mol% Grubbs’ first generation catalyst and 4 equivalents of 1-hexyne **9a** in dried, degassed CH_2_Cl_2_ resulted in formation of the target isoindolinone **10a** with only 5% conversion (entry 3).^17^ Treating diyne **6a** with 1-hexyne **9a** and 1 mol% Cp*RuCl(cod) in dried, degassed DCE also gave isoindolinone **10a**, again with 5% conversion of **6a** (entry 4).^10^ Given that the latter procedure gave a similar conversion with a lower catalyst loading, Cp*RuCl(cod) was selected for subsequent optimization.

Interestingly, treating diyne **6a** with 1-hexyne **9a** and 1 mol% Cp*RuCl(cod) with no solvent (neat) at 0 °C gave isoindolinone **10a** with a 50% conversion (entry 5). This suggests that using DCE as a solvent for this reaction is actually detrimental. In addition to the desired isoindolinone **10a**, dimer **11** was also formed as a significant by-product.^12^

Crucially, regioisomeric cyclotrimerization product **12** was not observed at all in the crude ^1^H NMR spectrum. The reaction under neat conditions reached completion within 16 h when 3 mol% of catalyst **3** was used, and with a significant reduction in the proportion of homo-coupled product **11** produced (entry 6).

We were interested in using cyclopentyl methyl ether (CPME) as a solvent for this cyclization as it has been recently established as a safer and more environmentally benign alternative to many traditional organic solvents.^18^ As shown in entry 7, when the reaction was conducted in CPME with 3 mol% of catalyst **3**, diyne **6a** was completely consumed within 16 h and an improved selectivity for the cross-coupled product **10a** was observed. By comparison, the same reaction using only 1 mol% catalyst resulted in a comparable level of selectivity, but a lower conversion (entry 8). Reducing the number of equivalents of 1-hexyne **9a** to two resulted in the complete consumption of diyne **6a** but also a significantly increased level of homo-coupling.

In an attempt to minimise the formation of dimer **11**, diyne **6a** was added dropwise over 3 h to a stirring solution of monoyne **9a** and catalyst **3**,^19^ and this proved to be highly effective (entry 10). When using the 3-hour dropwise addition it was possible to reduce the number of equivalents of 1-hexyne **9a** from four to two with no increase in homo-coupling (entry 11). A further reduction to 1.1 equivalents of 1-hexyne **9a** did result in increased homo-coupling, but target isoindolinone **10a** was still the major product (entry 12). The cyclization of **6a** and **9a** was also effective when 2-MeTHF or MTBE were used as solvents, but in both cases a greater degree of homo-coupling of **6a** was observed than with CPME (entries 13 and 14). The reaction proved to be relatively water tolerant, with a significant conversion and a reasonable selectivity observed when the reaction was conducted in the presence of 10% water (entry 15). Cyclization was even observed when the reaction was conducted in water as solvent (entry 16). This is important as it could enable the extension of the reaction to aqueous conditions for reactions of water-soluble substrates.

Following the optimization study the conditions described in entry 11 were taken as the “optimized” cyclization conditions as they required a reduced excess of monoyne and minimized the formation of dimer **11**. Crucially this protocol did not require the CPME solvent to be either degassed or dried. This, together with the environmental benefits of CPME, makes this reaction a very practical method for the synthesis of isoindolinones. Dimer **11** could be readily separated from the desired product by flash column chromatography, and the optimized conditions described in entry 11 gave the target isoindolinone **10a** in 81% isolated yield (Table 2, entry 1). This reaction was also scaled up to a 500-mg scale and isoindolinone **10a** was isolated in 66% yield (428 mg product).

**Table 2:** Reaction of diyne 6a with a selection of monoynes 9.a]
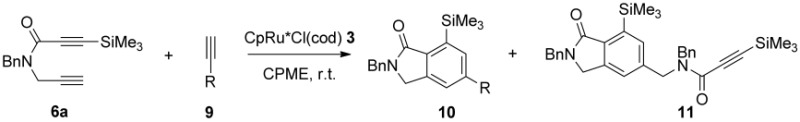

Entry	Alkyne 9		3[mol%]	Time [h]	Product10	Yield of10[%]b]	Ratio10:11^c^
1	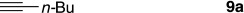	**9a**	3	16	**10a**	81	9:1
2	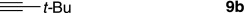	**9b**	3	16	**10b**	66	2:1
3	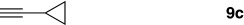	**9c**	3	16	**10c**	81	9:1
4	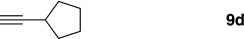	**9d**	3	16	**10d**	81	6:1
5	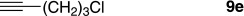	**9e**	3	16	**10e**	83	8:1
6	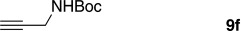	**9f**	5	24	**10f**	63	2:1
7	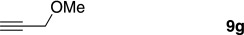	**9g**	3	16	**10g**	56	3:2
8	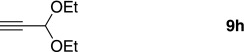	**9h**	3	24	**10h**	43	4:5
9	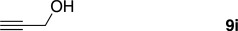	**9i**	3	16	**–**	0	–
10	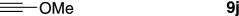	**9j**	3	16	**–**	0	–
11	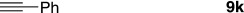	**9k**	4	24	**10k**	83	6:1
12	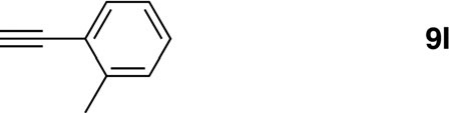	**9l**	3	16	**10l**	93	>10:1
13	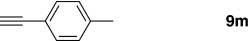	**9m**	4	24	**10m**	83	6:1
14	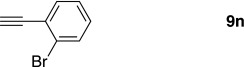	**9n**	3	16	**10n**	80	8:1
15	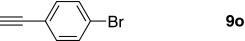	**9o**	3	24	**10o**	83	5:1
16	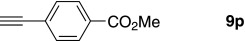	**9p**	3	24	**10p**	79	5:1
17	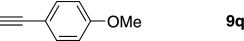	**9q**	5	24	**10q**	79	6:1
18	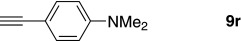	**9r**	10	24	**10r**	79	7:1
19	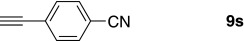	**9s**	3	16	**–**	0	-
20	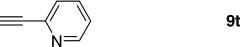	**9t**	20	24	**10t**	50	2:1
21	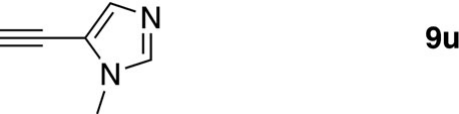	**9u**	3	16	**10u**	0	-
22	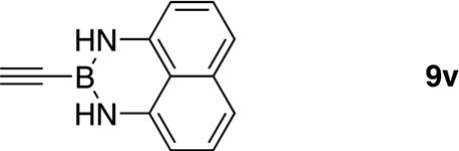	**9v**	5	24	**10v**	55	3:1

[a] *Reaction conditions:* A solution of **6a** in CPME was added dropwise to a stirring solution of **9** and **3** in CPME over 3 h at room temperature.

[b] Isolated yield.

c Determined by the analysis of crude ^1^H NMR spectra.

### Monoyne Scope

The cyclization of **6a** was then examined with a variety of monoynes using the optimized conditions described above to determine how robust the reaction was for a range of different substrates. Diyne **6a** cyclized with a wide range of monynes **9** as detailed in Table 2. Crucially, no evidence for the formation of regioisomeric isoindolinones was observed in any of the cyclization reactions. Alkyl monoynes **9a–e** cyclized efficiently with **6a** to give the corresponding isoindolinones **10a–e** in good isolated yield (entries 1–5, 66–83%). Little formation of the undesired dimer **11** was observed, except in the reaction of *tert*-butylacetylene **9b**, presumably due to high steric crowding about the monoalkyne. Carbamate **9f** cyclized with **6a** to give **10f** in reasonable yield and with modest levels of homo-coupling (entry 6).

Ether **9g** and acetal **9h** both underwent cyclotrimerization with **6a**, but with the formation of significant quantities of dimer **11**. Propargylic alcohol **9i** and methoxyacetylene **9j** both failed to cyclize with diyne **6a**, with only starting material being recovered in both cases. In addition to aliphatic monoynes, diyne **6a** cyclized effectively with a broad range of aromatic monoynes. Electron-rich (entries 12, 13, 17 and 18), electron-poor (entry 16) and sterically hindered substrates (entries 12 and 14) could all be tolerated and products were isolated in good yields (79–93%) with low levels of diyne homo-coupling. For most of these examples longer reaction times (up to 24 h), and in some cases higher catalyst loadings, were required to drive the reaction to completion. However the reactions with *ortho*-substituted arylacetylenes **9l** and **9n** reached completion within 16 h with only 3 mol% of catalyst **3** (entries 12 and 14). Monoyne **9l** also cyclized with exceptionally high selectivity for the cross-coupled product **10l** over dimer **11**, whereas *ortho*-bromo alkyne **9n** gave a slightly lower selectivity. Although Yamamoto et al. have reported the [2+2+2] cycloaddition of an electron-deficient nitrile and an amide-tethered diyne to give a pyridine,^20^ in our reaction nitrile **9s** failed to cyclize with **6a** to form any product *via* reaction of either the alkyne or the nitrile (entry 19). Only a limited quantity of **11** (∼10%) was formed in this reaction suggesting that **9s** may inhibit the catalyst. Heterocycle-containing alkyne **9t** cyclized effectively with **6a** to give the corresponding 2-pyridyl derivative **10t** in a moderate 50% yield (entry 20). In contrast *N*-methylimidazole **9u** failed to cyclize with **6a**, with unreacted starting material being recovered (entry 21). Alkyne **9v** cyclized with **6a** to give boramide **10v** in reasonable yield (entry 22).^21^

### Diyne Scope

The cyclization of amide-tethered diynes bearing different *N*-substituents was examined and the results are summarized in Table 3. *N*-*t-*Bu diyne **6b** proved to be an excellent substrate for the synthesis of 5,7-substituted isoindolinones. Treatment of **6b** with 1-hexyne **9a** under the optimized reaction conditions gave isoindolinone **13a** in 84% yield with little formation of the dimer **14a** (entry 1). The cyclization of **6b** with **9k** required 4 mol% **3** and 24 h to reach completion, giving isoindolinone **13b** in 89 % yield (entry 2). The reaction of **6b** with 2-ethynyltoluene **9l** proceeded in 94% yield without an elevated reaction time or an increased loading of catalyst **3**, and also occurred with very little formation of dimer **14a** (entry 3).

**Table 3:** Cyclizations involving diynes with different *N*-substituents.a]
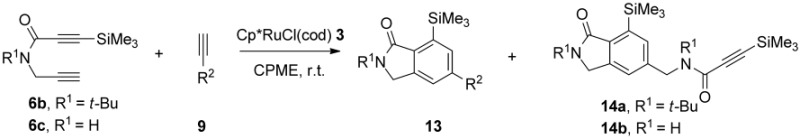

Entry	R^1^	R^2^	3[mol%]	Time [h]	Product13	Yield of13[%]b]	Ratio of13:14^c^
1	*t-*Bu **6b**	*n-*Bu **9a**	3	16	**13a**	84	10:1
2	*t-*Bu **6b**	Ph **9k**	4	24	**13b**	89	>10:1
3	*t-*Bu **6b**	*o*-tolyl **9l**	3	16	**13c**	94	>10:1
4	H **6c**	*n-*Bu **9a**	10	24	**13d**	51 (90%d])	2:1
5	H **6c**	*o*-tolyl **9l**	10	24	**13e**	62 (90%d])	7:1

[a] *Reaction conditions:* a solution of **6** in CPME was added dropwise to a stirring solution of **9** and **3** in CPME over 3 h at room temperature.

[b] Isolated yield.

c Determined by the analysis of crude ^1^H NMR spectra.

[d] Conversion of diyne **6** to **13**/**14** (determined by crude ^1^H NMR without the use of an internal standard).

The *N*-H diyne **6c** proved less effective for the synthesis of isoindolinones, with the cyclization of **6c** and 1-hexyne **9a** requiring 10 mol% Cp*RuCl(cod) **3** and 24 h to achieve a 90% conversion of diyne **6c** (entry 4). Isoindolinone **13d** was only formed in modest yield (51%) and significant formation of dimer **14b** was observed. Under the same conditions the cyclization of 2-tolylacetylene **9l** and N-*H* diyne **6c** gave the desired isoindolinone **13e** in a slightly higher yield with 90% conversion. Again, the reaction with 2-ethynyltoluene **9l** proved to be unusually selective, with **13e** and **14b** formed in the ratio 7:1 (entry 5). The lack of a sterically bulky *N*-substituent is presumably responsible for both the reduced reactivity of *N*-H diyne **6c** with monoynes and the high level of diyne homo-coupling observed in these reactions.

The cyclization of amide-tethered diynes bearing different alkyne substituents was also explored (Table 4). With doubly substituted diynes **6d** and **6e**, no homo-coupling of the diyne was observed and dropwise addition of the diyne to the reaction was unnecessary (entries 1–3). With 10 mol% of Cp*RuCl(cod), methyl-substituted diyne **6d** cyclized with 1-hexyne **9a** to form a 9:1 mixture of regioisomeric isoindolinones **15a** and **16a** (entry 1).

**Table 4:** Cyclizations involving diynes with different alkyne substitutents.a]
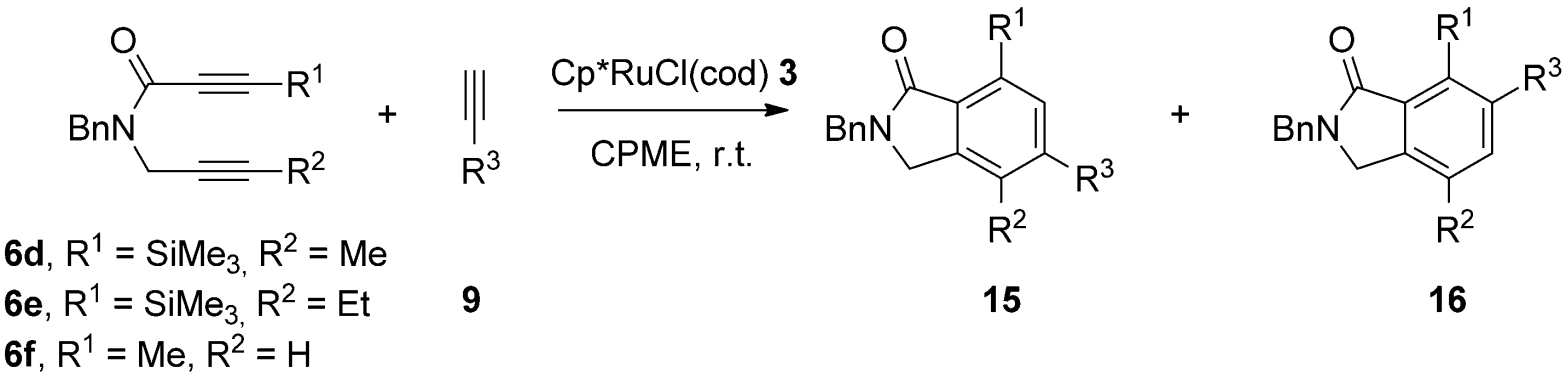

Entry	Diyne6	R^1^	R^2^	R^3^	3[mol %]	Time [h]	Isolated products	Yield of (15+16) [%]b]	Ratio of15:16^c^
1	**6d**	SiMe_3_	Me	*n-*Bu **9a**	10	24	**15a/16a**	69	9:1
2	**6e**	SiMe_3_	Et	*n-*Bu **9a**	10	24	**15b/16b**	57	2:1
3	**6e**	SiMe_3_	Et	*o*-tolyl **9l**	10	24	**15c/16c**	73	5:1
4^d^	**6f**	Me	H	*n-*Bu **9a**	3	16	**15de]**	85	>20:1
5^d^	**6f**	Me	H	*o*-tolyl **9l**	3	16	**15e**	94	>20:1

[a] *Reaction conditions:* A solution of **6** in CPME was added to a stirring solution of **9** and **3** in CPME over 1 min at room temperature.

[b] Isolated yield.

c Determined by the analysis of crude ^1^H NMR spectra.

[d] Diyne **6f** in CPME was added dropwise over 3 h to a solution of **9** and **3** in CPME.

[e] Evidence of limited homo-coupling of **6f** was observed in the crude ^1^H NMR spectrum.

Ethyl-substituted diyne **6e** reacted with 1-hexyne **11a** with lower regioselectivity, giving a 2:1 mixture of isoindolinones **15b** and **16b** (entry 2). However, diyne **6e** cyclized with 2-ethynyltoluene **9l**, to give a 5:1 mixture of isoindolinones **15c** and **16c** (entry 3). Interestingly, the presence of diastereotopic benzylic protons in the ^1^H NMR spectrum suggests that isoindolinone **15c** is a chiral molecule, presumably due to restricted rotation about the hindered biaryl unit.

The dependence of the cyclotrimerization on an SiMe_3_ regiodirecting group was also investigated. Diyne **6f** with a terminal methyl substituent reacted with 1-hexyne **9a** under the optimized cyclization conditions to give isoindolinone **15d** in 85% yield (entry 4). Crucially, there was no trace of the regioisomeric isoindolinone **16d** by crude ^1^H NMR. Similarly, diyne **6f** cyclized with 2-ethynyl toluene **9l** to give isoindolinone **15e** in 94% yield, with no evidence for the formation of regioisomer **16e** (entry 5).

### Functional Group Manipulation of Cyclized Products

**Scheme 3:**
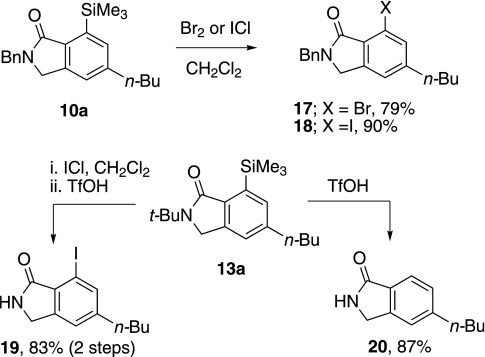
Synthesis of usefully functionalized isoindolinones.

Conversion of the cyclized isoindolinone products into a number of synthetically interesting motifs was examined. Isoindolinone **10a** was converted to aryl halides **17** and **18**, in 79% and 90% yields, respectively, *via* an *ipso* substitution of the silyl group (Scheme 3).^22^ Treatment of *N*-*t*-butylisoindolinone **13a** with triflic acid resulted in a simultaneous deprotection of the lactam and protodesilylation within 30 min to give *N*-H isoindolinone **20** in good yield.^23^ Alternatively, treatment of **13a** with iodine monochloride followed by deprotection with triflic acid gave 7-iodoisoindolinone **19** in 83% yield. Thus, an *N*-*t-*Bu diyne can be used as an indirect method for the synthesis of *N*-H isoindolinones *via* this acid-mediated deprotection.

**Scheme 4:**
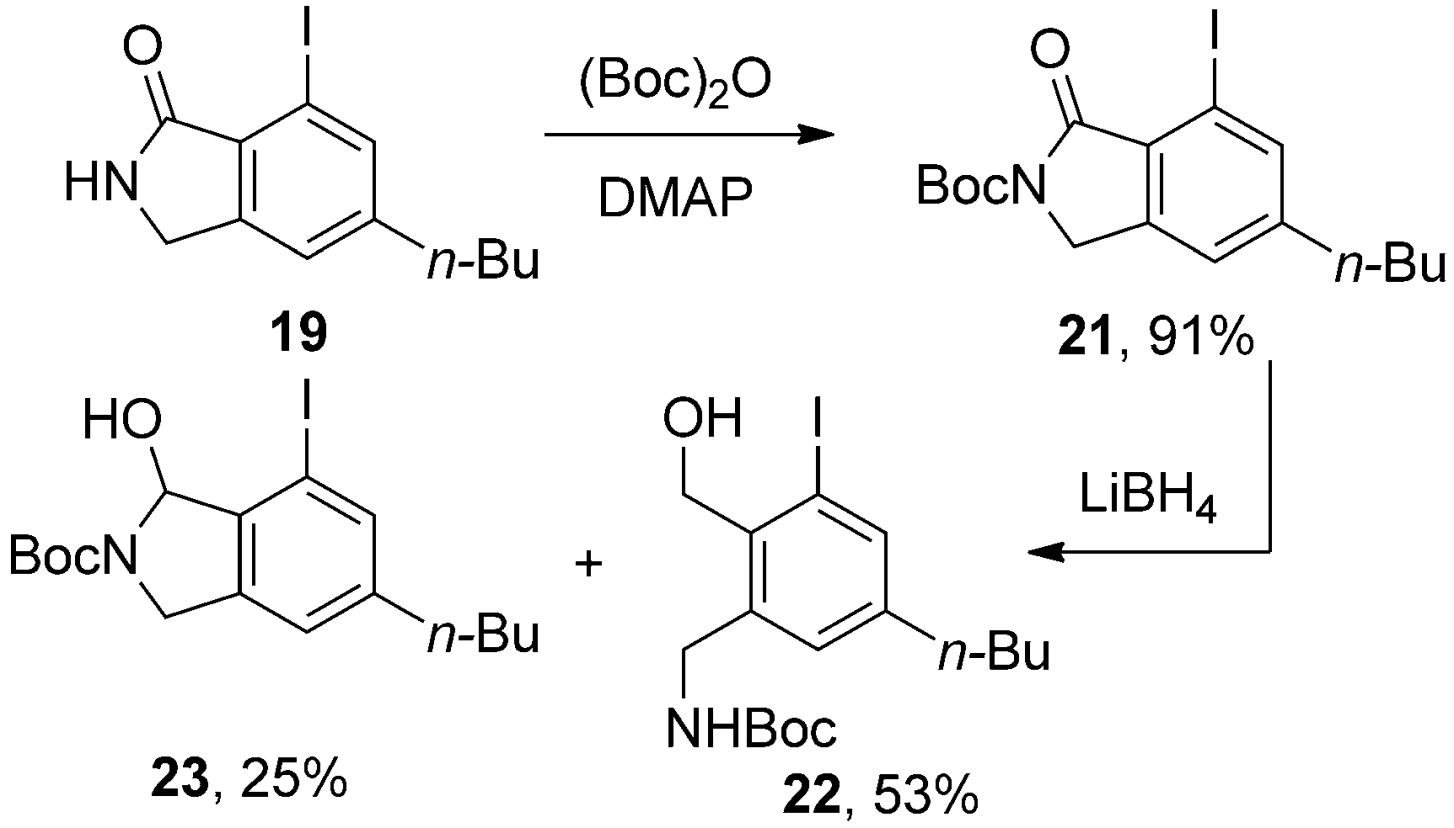
Synthesis of a tetrasubstituted benzene ring.

It was also possible to access a tetrasubstituted monocyclic benzene. Treatment of *N*-H isoindolinone **19** with di-*tert*-butyl dicarbonate gave *N*-Boc isoindolinone **21**, which could be reduced with lithium borohydride to form *N*-Boc protected amino alcohol **22**, together with cyclic aminol **23**, in a combined yield of 78% (Scheme 4). The preparation of mono-cyclic substituted arenes *via* tethered alkyne cyclotrimerizations has little precedent and such systems are somewhat difficult to access *via* traditional aromatic substitution reactions, highlighting the value of this strategy.^24^

## Conclusions

In summary, we have demonstrated the regioselective synthesis of polysubstituted isoindolinones *via* the Cp*RuCl(cod)-catalyzed cyclotrimerization of amide-tethered diynes and monoynes. This cyclization is effective with a wide range of structurally diverse monoynes and was demonstrated to work with a variety of different diynes. We have also demonstrated that the cyclization products could be converted into a range of functionalized isoindolinones and a tetrasubstituted benzene derivative.

## Experimental Section

Full experimental details are provided in the Supporting Information.

### Cp*RuCl(cod)-Catalyzed Cyclization of a Diyne and a Monoyne

A solution of **6a** (500 mg, 1.86 mmol) in CPME (11 mL) was added dropwise over 3 h to a stirring solution of 1-hexyne **9a** (0.43 mL, 300 mg, 3.7 mmol) and Cp*RuCl(cod) (21 mg, 3 mol%) in CPME (7.7 mL) at room temperature. The reaction mixture was stirred for a further 13 h before being filtered through a silica pad, eluting with ethyl acetate. The solvent was removed under vacuum to give the crude product, which was purified by flash column chromatography (13:1 petrol:ethyl acetate) to give 2-benzyl-5-butyl-7-(trimethylsilyl)isoindolin-1-one **10a**; yield: 428 mg (1.22 mmol, 66 %); *R*_f_=0.36 (6:1 petrol:ethyl acetate); IR (film): ν_max_=2955 (m, C–H), 2930 (m, C–H), 1688 (s, C–O), 1454 (m), 1409 cm^−1^ (m); ^1^H NMR (600 MHz, DMSO-*d*_6_): *δ*=7.34–7.21 (7 H, m, Ar*H*), 4.68 (2 H, s, C*H*_2_N), 4.24 (2 H, s, C*H*_2_N), 2.60 (2 H, t, *J*=7.7, ArC*H*_2_CH_2_), 1.51, (2 H, m, ArCH_2_C*H*_2_), 1.26 (2 H, m, C*H*_2_CH_3_), 0.83 (3 H, t, *J*=7.4, CH_2_C*H*_3_), 0.34 [9 H, s, Si(C*H*_3_)_3_]; ^13^C NMR (125 MHz, DMSO-*d*_6_): *δ*=168.5, 144.8, 142.1, 137.7, 136.9, 134.3, 134.0, 128.6, 127.6, 127.2, 123.7, 48.9, 45.4, 35.1, 33.2, 21.8, 13.7, −0.4; HR-MS (EI^+^): *m/z*=351.2011 [M]^+^, C_22_H_29_ONSi requires 351.2013.

## Supporting Information

As a service to our authors and readers, this journal provides supporting information supplied by the authors. Such materials are peer reviewed and may be re-organized for online delivery, but are not copy-edited or typeset. Technical support issues arising from supporting information (other than missing files) should be addressed to the authors.
