# Supplementary material for: Highly Regioselective Synthesis of Substituted Isoindolinones via Ruthenium-Catalyzed Alkyne Cyclotrimerizations
Source: Adv Synth Catal. 2013 Aug 12;355(11-12):2353–60. doi: 10.1002/adsc.201300055 (PMC3793232; doi:10.1002/adsc.201300055)

Advanced Synthesis & Catalysis

Supporting Information

© Copyright Wiley-VCH Verlag GmbH & Co. KGaA, 69451 Weinheim, 2013

Highly Regioselective Synthesis of Substituted Isoindolinones via Ruthenium-Catalysed Alkyne Cyclotrimerizations

Robert W. Foster,[a] Christopher J. Tame,[b] Helen C. Hailes[a] and Tom D. Sheppard[a]*

[a] Department of Chemistry, University College London, Christopher Ingold Laboratories, WC1H 0AJ London, UK Tel: +(44)-(0)20-7679-2467; Fax: +(44)-(0)20-7679-7463; e-mail: tom.sheppard@ucl.ac.uk

[b] GlaxoSmithKline, Medicines Research Centre, Gunnels Wood Road, Stevenage, Hertfordshire, UK, SG1 2NY

Table of Contents

General Experimental Details....S2
General Experimental Procedures....S2
Compound Synthesis: Experimental Details & Compound Characterisation....S2
References....S25
NMR Spectra....S27

General Experimental Details

All solvents and chemicals were used as obtained from commercial suppliers. Column chromatography was carried out using BDH (40-63 μm) silica gel and analytical thin layer chromatography was carried out using Merck Kieselgel aluminium-backed plates coated with silica gel. Components were visualised using combinations of UV (254 nm) and potassium permanganate. Infrared (IR) spectra were recorded on a Perkin-Elmer spectrum 100 FT-IR spectrometer as thin films. ^{1}H and ^{13}C NMR spectra were recorded respectively at 400 MHz and 100 MHz on a Bruker Avance 400 spectrometer, 500 MHz and 125 MHz in a Bruker Avance 500 or at 600 MHz and 150 MHz on a Bruker Avance 600 spectrometer in the stated solvent. Mass spectra were obtained using either a VG70-SE or MAT 900XP spectrometer at the Department of Chemistry, University College London.

General Experimental Procedures

General Amide Formation Procedure

According to the modified procedure of Sato *et al.*[1]: Oxalyl chloride (1.1 eq.) was added dropwise to a stirring solution of 3-(trimethylsilyl)propiolic acid **8** (1.2 eq.) and dimethylformamide (a few drops) in 2-MeTHF (0.85 mL/mmol of acid **8**) at room temperature. The reaction was stirred for 1 h before the crude acid chloride solution was added dropwise to a stirring solution of amine **7** (1.0 eq.) and triethylamine (2.5 eq.) in 2-MeTHF (4.5 mL/mmol of amine **7**) at room temperature. The reaction was stirred for 1 h before being filtered through a silica plug, eluting with ethyl acetate. The solvent removed *in vacuo* to give the crude amide.

General Alkyne Cyclotrimerization Procedure

A solution of diyne (0.26 mmol) in CPME (1.6 mL) was added dropwise over 3 h to a stirring solution of monoyne (2.0 eq.) and Cp*RuCl(cod) **3** in CPME (1.1 mL) at room temperature. The reaction was stirred for a specific period of time before the reaction mixture was filtered through a silica pad, eluting with ethyl acetate. The solvent was removed in vacuo to give the crude isoindolinone product.

Compound Synthesis: Experimental Details & Compound Characterisation

3-(Trimethylsilyl)propiolic acid 8

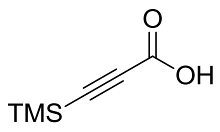

According to the modified procedure of Fleming *et al.*[2]: A solution of ethynyltrimethylsilane (22 mL, 15 g, 150 mmol) in dry THF (120 mL) was added slowly to a stirring solution of ethyl magnesium bromide (1 M in THF, 191 mL, 191 mmol) at 0 °C. The reaction mixture was stirred for 2 h at room temperature before being cooled to -20 °C. The solution was cautiously added to dry ice (20 g, 450 mmol) and was stirred for 16 h, with the reaction allowed to slowly reach room temperature. The reaction was cooled to 0 °C and was quenched by dropwise addition of 1 M aq. HCl. The mixture was extracted with petrol (4 × 100 mL), washed with brine (200 mL), dried ($MgSO_4$) and the solvent removed *in vacuo* to give the crude product, which was purified by vacuum distillation (b.p. 78–80 °C at 8 Torr) to give 3-(trimethylsilyl)propiolic acid **8** as a white crystalline solid (16.7 g, 117 mmol, 78%); m.p. 38–40 °C; b.p. 78–80 °C at 8 Torr (literature 108–100 °C at 10 Torr)[3]; R_f = 0.25 (1:1 petrol:ethyl acetate); ν_{max} (film/cm^{-1}) 3000m br. (O-H), 2965s (C-H), 2181w (C≡C), 1691s (C=O), 1402m;

^{1}H NMR (400 MHz, $CDCl_3$) 10.28 (1H, br. s, C(O)O*H*), 0.28 (9H, s, $Si(CH_3)_3$); ^{13}C NMR (100 MHz, $CDCl_3$) 157.5, 97.5, 93.7, -1.0; data in accordance with the literature.[2]

N-Benzylprop-2-yn-1-amine 7a

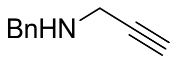

According to the modified procedure of Burton and Hess[4]: Propargyl bromide (6.7 mL, 8.8 g, 80 wt. % in toluene, 60 mmol) was added dropwise to benzylamine (40 mL, 39 g, 360 mmol) with continuous stirring at room temperature. The reaction was stirred for 16 h and then partitioned between 2 M aq. sodium hydroxide (50 mL) and diethyl ether (50 mL). The aqueous extract was washed with diethyl ether (2 × 50 mL) and the combined organic extracts were washed with brine (100 mL), dried ($MgSO_4$) and the solvent was removed *in vacuo.* The crude product was purified by flash column chromatography (3:1 petrol:ethyl acetate) to give *N*-benzylprop-2-yn-1-amine **7a** (7.05 g, 48.6 mmol, 81%) as a yellow oil; $R_f = 0.36$ (3:1 petrol:ethyl acetate); ν_{max} (film/cm^{-1}) 3292s (CC-H, N-H), 2838s (C-H), 1494s; ^{1}H NMR (400 MHz, $CDCl_3$) 7.39–7.26 (5H, m, Ar*H*), 3.91 (2H, s, CH_2Ph), 3.46 (2H, d, $J = 2.4$, CH_2CC), 2.29 (1H, t, $J = 2.4$, CC*H*) 1.58 (1H, br. s, N*H*); ^{13}C NMR (100 MHz, $CDCl_3$) 139.4, 128.5, 128.5, 127.2, 82.1, 71.6, 52.3, 37.4; data in accordance with the literature.[4]

N-(*tert*-Butyl)prop-2-yn-1-amine 7b

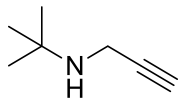

According to the modified procedure of Sulpizo *et al.*[5]: Propargyl bromide (13 mL, 17 g, 80 wt. % in toluene, 120 mmol) was added dropwise to a stirring solution of 2-methylpropan-2-amine (37 mL, 26 g, 350 mmol) in diethyl ether (35 mL) at room temperature. The reaction was stirred at room temperature for 36 h before being concentrated *in vacuo*. The crude product was purified by distillation at atmospheric pressure (b.p. = 125–127 °C) to give *N*-(*tert*-butyl)prop-2-yn-1-amine **7b** as a colourless oil (4.10 g, 79 wt. % with toluene, 29 mmol, 24%); ν_{max} (film/cm^{-1}) 3306s (CC-H), 3245m br. (N-H), 2963s (C-H), 1476s; ^{1}H NMR (600 MHz, $CDCl_3$) 3.27 (2H, d, $J = 2.3$, CH_2N), 2.11 (1H, t, $J = 2.3$, CC*H*), 1.03 (9H, s, $C(CH_3)_3$); ^{13}C NMR (150 MHz, $CDCl_3$) 83.4, 70.8, 50.9, 32.0, 28.8; data in accordance with the literature.[5]

N-Benzylbut-2-yn-1-amine 7d

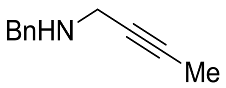

According to the modified procedure of Burton and Hess[4]: 1-Bromobut-2-yne (0.66 mL, 1.0 g, 7.5 mmol) was added dropwise to benzylamine **78** (4.9 mL, 4.8 g, 45 mmol) with continuous stirring at 0 °C. The reaction was stirred for 16 h at room temperature and then partitioned between 1 M aq. sodium hydroxide (30 mL) and diethyl ether (30 mL). The aqueous extract was washed with diethyl ether (2 × 30 mL) and the combined organic extracts were washed with brine (100 mL), dried ($MgSO_4$) and the solvent was removed *in vacuo.* The crude product was purified by flash column chromatography (5:1 petrol:ethyl acetate) to give *N*-benzylbut-2-yn-1-amine **7d** as a colourless oil (738 mg, 4.64 mmol, 62%); $R_f = 0.38$ (2:1 petrol:ethyl acetate); ν_{max} (film/cm^{-1}) 3319m (N-H), 2917s (C-H), 1495s, 1453s; ^{1}H NMR (600 MHz, $CDCl_3$) 7.36–7.31 (4H, m, Ar*H*), 7.27–7.23 (1H, m, Ar*H*), 3.85 (2H, s, CH_2Ph), 3.38 (2H, q, $J = 2.3$, $CH_2C{\equiv}C$), 1.85 (3H, t, $J = 2.3$, CH_3), 1.51 (1H, br. s, N*H*); ^{13}C NMR (150 MHz, $CDCl_3$) 139.8, 128.5, 128.5, 127.2, 79.3, 77.3, 52.7, 38.0, 3.7; data in accordance with the literature.[6]

N-Benzylpent-2-yn-1-amine 7e

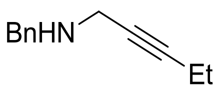

According to the modified procedure of Burton and Hess[4]: 1-bromopent-2-yne (0.72 mL, 1.0 g, 7.0 mmol) was added dropwise to benzylamine **78** (4.5 mL, 4.5 g, 42 mmol) with continuous stirring at 0 °C. The reaction was stirred for 16 h at room temperature and then partitioned between 1 M aq. sodium hydroxide (30 mL) and diethyl ether (30 mL). The aqueous extract was washed with diethyl ether (2 × 30 mL) and the combined organic extracts were washed with brine (100 mL), dried ($MgSO_4$) and the solvent was removed *in vacuo*. The crude product was purified by flash column chromatography (10:1 petrol:ethyl acetate) to give *N*-benzylpent-2-yn-1-amine **7e** as a pale yellow oil (858 mg, 4.96 mmol, 71%); $R_f = 0.24$ (5:1 petrol:ethyl acetate); ν_{max} (film/cm^{-1}) 3313w br. (N-H), 2975s (C-H), 1639s, 1453s; ^{1}H NMR (400 MHz, $CDCl_3$) 7.38–7.32 (4H, m, Ar*H*), 7.30–7.28 (1H, m, Ar*H*), 3.88 (2H, s, CH_2Ph), 3.42 (2H, t, *J* = 2.2, NCH_2C≡C), 2.25 (2H, qt, *J* = 7.5, 2.2, C$H_2$$CH_3$), 1.53 (1H, br. s, N*H*), 1.17 (3H, t, *J* = 7.5, CH_3); ^{13}C NMR (150 MHz, $CDCl_3$) 139.8, 128.5, 128.5, 127.2, 85.4, 77.4, 52.6, 38.0, 14.3, 12.6; data in accordance with the literature.[4]

N-Benzyl-*N*-(prop-2-yn-1-yl)-3-(trimethylsilyl)propiolamide 6a

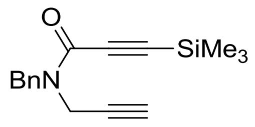

Prepared from *N*-benzylprop-2-yn-1-amine **7a** (2.84 g, 19.6 mmol) according to the General Amide Formation Procedure and purified by flash column chromatography (9:1 petrol:ethyl acetate) to give *N*-benzyl-*N*-(prop-2-yn-1-yl)-3-(trimethylsilyl)propiolamide **6a** (4.48 g, 16.6 mmol, 85%) as a yellow oil; $R_f = 0.44$ (6:1 petrol:ethyl acetate); ν_{max} (film/cm^{-1}) 3292m (CC-H), 2962m (C-H), 1634s (C=O), 1417s; ^{1}H NMR (400 MHz, $CDCl_3$) a mixture of rotamers R_1 (major) and R_2 (minor); 7.40–7.28 (5H, m, Ar*H* R_1; 5H, m, Ar*H* R_2), 4.93 (2H, s, CH_2Ph R_1), 4.74 (2H, s, CH_2Ph R_2), 4.27 (2H, d, *J* = 2.5, CH_2CC R_2), 4.12 (2H, d, *J* = 2.5, CH_2CC R_1), 2.35 (1H, t, *J* = 2.5, CC*H* R_2), 2.25 (1H, t, *J* = 2.5, CC*H* R_1), 0.25 (9H, s, Si(CH_3)$_3$ R_2), 0.22 (9H, s, Si(CH_3)$_3$ R_1); ^{13}C NMR (125 MHz, $CDCl_3$) a mixture of rotamers; 153.6, 153.5, 135.7, 135.5, 128.9, 128.7, 128.6, 128.2, 128.0, 127.9, 98.7, 98.5, 95.7, 95.3, 77.7, 77.6, 73.3, 72.6, 51.5, 46.5, 37.6, 32.3, -0.7, -0.8; HRMS (ESI$^+$) found $[M+H]^+$ 270.1308; $C_{16}H_{20}NOSi$ requires 270.1314.

N-(*tert*-Butyl)-*N*-(prop-2-yn-1-yl)-3-(trimethylsilyl)propiolamide 6b

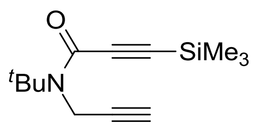

Prepared from *N*-(*tert*-butyl)prop-2-yn-1-amine **7b** (0.500 mg, 87 wt. % in toluene, 3.9 mmol) according to the General Amide Formation Procedure and purified by flash column chromatography (12:1 petrol:ethyl acetate) to give *N*-(*tert*-butyl)-*N*-(prop-2-yn-1-yl)-3-(trimethylsilyl)propiolamide **6b** as a white crystalline solid (612 mg, 2.6 mmol, 66%); m.p. 25–27 °C; $R_f = 0.34$ (12:1 petrol:ethyl acetate); ν_{max} (film/cm^{-1}) 3252s (CC-H), 2965s (C-H), 1633s (C=O); ^{1}H NMR (600 MHz, DMSO-d_6) a mixture of rotamers R_1 (major) and R_2 (minor) 4.44 (2H, d, *J* = 0.5, CH_2N R_1), 4.23 (2H, br. s, CH_2N R_2), 3.35 (1H, t, *J* = 0.5, CC*H* R_1), 3.32 (1H, br. s, CC*H* R_2), 1.60 (9H, s, C(CH_3)$_3$ R_2), 1.42 (9H, s, C(CH_3)$_3$ R_1), 0.22 (9H, s, Si(CH_3)$_3$ R_1; 9H, s, Si(CH_3)$_3$ R_2); ^{13}C NMR (125 MHz, DMSO-d_6) 153.4, 97.9, 94.7, 80.9, 74.7, 57.6, 29.8, 27.6, -0.8; HRMS (ES$^+$) found $[M+Na]^+$ 258.1294; $C_{13}H_{21}NONaSi$ requires 258.1290.

N-(Prop-2-ynyl)-3-(trimethylsilyl)propiolamide 6c

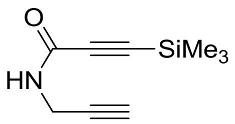

Prepared from propargylamine (0.34 mL, 0.29 g, 5.5 mmol) according to the General Amide Formation Procedure and purified by flash column chromatography (5:1 petrol:ethyl acetate) to give *N*-(prop-2-yn-1-yl)-3-(trimethylsilyl)propiolamide **6c** (789 mg, 4.40 mmol, 80%) as a white crystalline solid; m.p. 42–44 °C; R_f = 0.38 (5:1 petrol:ethyl acetate); ν_{max} (film/cm^{-1}) 3275s (CC-H and N-H), 2963m (C-H), 1638s (C=O), 1526s; ^{1}H NMR (600 MHz, DMSO-d_6) 9.15 (1H, t, *J* = 5.3, N*H*), 3.85 (2H, dd, *J* = 5.3, 2.4, C*H*$_2$N), 3.13 (1H, t, *J* = 2.4, CC*H*), 0.21 (9H, s, Si(C*H*$_3$)$_3$); ^{13}C NMR (125 MHz, DMSO-d_6) 151.4, 98.3, 90.5, 80.1, 73.3, 28.1, -0.8; HRMS (CI$^+$) found [M+H]$^+$ 180.0847; $C_9H_{14}NOSi$ requires 180.0847.

N-Benzyl-*N*-(but-2-yn-1-yl)-3-(trimethylsilyl)propiolamide 6d

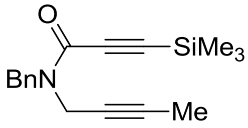

Prepared from *N*-benzylbut-2-yn-1-amine **7d** (650 mg, 4.09 mmol) according to the General Amide Formation Procedure and purified by flash column chromatography (11:1 petrol:ethyl acetate) to give *N*-benzyl-*N*-(but-2-yn-1-yl)-3-(trimethylsilyl)propiolamide **6d** as a colourless oil (826 mg, 2.92 mmol, 71%); R_f = 0.31 (12:1 petrol:ethyl acetate); ν_{max} (film/cm^{-1}) 2961s (C-H), 1631s (C=O), 1496s; ^{1}H NMR (500 MHz, DMSO-d_6) a mixture of rotamers R_1 (major) and R_2 (minor); 7.40–7.21 (5H, m, Ar*H* R_1; 5H, m, Ar*H* R_2), 4.81 (2H, s, PhC*H*$_2$ R_1), 4.56 (2H, s, PhC*H*$_2$ R_2), 4.25 (2H, q, *J* = 2.3, C*H*$_2$C≡C R_2), 4.01 (2H, q, *J* = 2.3, C*H*$_2$C≡C R_1), 1.77 (3H, t, *J* = 2.3, CC*H*$_3$ R_2), 1.74 (3H, t, *J* = 2.3, CC*H*$_3$ R_1), 0.22 (9H, s, Si(C*H*$_3$)$_3$ R_2), 0.17 (9H, s, Si(C*H*$_3$)$_3$ R_1); ^{13}C NMR (125 MHz, DMSO-d_6) a mixture of rotamers; 152.6, 152.5, 136.3, 136.2, 128.6, 128.5, 127.8, 127.7, 127.4, 97.6, 97.3, 96.2, 95.8, 80.9, 80.0, 73.7, 73.6, 51.3, 46.9, 38.5, 33.4, 3.0, 3.0, -0.9, -1.0; HRMS (CI$^+$) found [M+H]$^+$ 284.1459; $C_{17}H_{22}NOSi$ requires 284.1465.

N-Benzyl-*N*-(pent-2-yn-1-yl)-3-(trimethylsilyl)propiolamide 6e

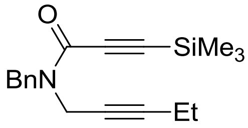

Prepared from *N*-Benzylpent-2-yn-1-amine **7e** (710 mg, 4.10 mmol) according to the General Amide Formation Procedure and purified by flash column chromatography (12:1 petrol:ethyl acetate) to give *N*-benzyl-*N*-(pent-2-yn-1-yl)-3-(trimethylsilyl)propiolamide **6e** as a colourless oil (823 mg, 2.95 mmol, 72%); R_f = 0.39 (12:1 petrol:ethyl acetate); ν_{max} (film/cm^{-1}) 2964s (C-H), 1632s (C=O), 1415s; ^{1}H NMR (600 MHz, DMSO-d_6) a mixture of rotamers R_1 (major) and R_2 (minor); 7.40–7.23 (5H, m, Ar*H* R_1; 5H, m, Ar*H* R_2), 4.82 (2H, s, PhC*H*$_2$ R_1), 4.57 (2H, s, PhC*H*$_2$ R_2), 4.28 (2H, t, *J* = 2.0, NC*H*$_2$C≡C R_2), 4.05 (2H, t, *J* = 2.0, NC*H*$_2$C≡C R_1), 2.18–2.11 (3H, m, C*H*$_2$CH$_3$ R_1; 3H, m, C*H*$_2$CH$_3$ R_2), 1.03–0.98 (3H, m, CH$_2$C*H*$_3$ R_1; 3H, m, CH$_2$C*H*$_3$ R_2;), 0.24 (9H, s, Si(C*H*$_3$)$_3$ R_2), 0.18 (9H, s, Si(C*H*$_3$)$_3$ R_1); ^{13}C NMR (125 MHz, DMSO-d_6) a mixture of rotamers; 152.7, 152.5, 136.4, 136.3, 128.7, 128.5, 127.9, 127.7, 127.5, 127.5, 97.6, 97.4, 96.2, 95.8, 86.4, 85.7, 74.0, 73.8, 51.4, 47.1, 38.6, 33.5, 13.6, 11.6, 11.6, -0.9, -1.0; HRMS (CI$^+$) found [M+H]$^+$ 298.1617; $C_{18}H_{24}NOSi$ requires 298.1622.

N-Benzyl-*N*-(prop-2-yn-1-yl)but-2-ynamide 6f

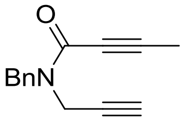

According to the modified procedure of Yamamoto *et al.*[7]: A solution of 2-butynoic acid (410 mg, 4.88 mmol) in dry CH_2Cl_2 (3.0 mL) was added dropwise to a stirring solution of *N*-benzylprop-2-yn-1-amine **79** (653 mg, 4.50 mmol), 4-(dimethylamino)pyridine (62 mg, 0.51 mmol) and *N*-(3-dimethylaminopropyl)-*N′*-ethylcarbodiimide hydrochloride (978 mg, 5.09 mmol) in dry CH_2Cl_2 (8.6 mL) at 0 °C. The reaction was stirred for 16 h at room temperature before the reaction was partitioned between ethyl acetate (30 mL) and 1 M NaOH (30 mL). The aqueous extract was washed with ethyl acetate (3 × 30 mL) and the combined organic extracts were washed with brine (100 mL), dried ($MgSO_4$) and the solvent was removed *in vacuo* to give the crude product. This was dissolved in ethyl acetate (30 mL) and stirred with Amberlyst® 15 (1.0 g) for 5 minutes. The mixture was then filtered and the resin washed with ethyl acetate (30 mL). The filtrate was concentrated *in vacuo* and purified by flash column chromatography (4:1 petrol:ethyl acetate) to give *N*-benzyl-*N*-(prop-2-yn-1-yl)but-2-ynamide as a colourless oil (678 mg, 3.21 mmol, 71%); R_f = 0.28 (4:1 petrol:ethyl acetate); ν_{max} (film/cm^{-1}) 3290s (CC-H), 2919w (C-H), 1624s (C=O), 1415s; ^{1}H NMR (600 MHz, DMSO-d_6) a mixture of rotamers R_1 (major) and R_2 (minor); 7.41–7.38 (2H, m, Ar*H* R_1/R_2), 7.36–7.27 (6H, m, Ar*H*, R_1/R_2), 7.24–7.21 (2H, m, Ar*H* R_1/R_2), 4.82 (2H, s, PhC*H*$_2$ R_1), 4.57 (2H, s, PhC*H*$_2$ R_2), 4.30 (2H, d, *J* = 2.4, C*H*$_2$C≡C R_2), 4.02 (2H, d, *J* = 2.4, C*H*$_2$C≡C R_1), 3.37 (1H, t, *J* = 2.4, C≡C*H* R_2), 3.22 (1H, t, *J* = 2.4, C≡C*H* R_1), 2.06 (3H, s, C*H*$_3$ R_2), 2.03 (3H, s, C*H*$_3$ R_2); ^{13}C NMR (125 MHz, DMSO-d_6) a mixture of rotamers; 153.5, 153.4, 136.3, 136.2, 128.8, 128.6, 127.8, 127.8, 127.5, 127.5, 90.7, 90.4, 78.7, 79.5, 75.6, 74.7, 72.8, 72.6, 51.2, 46.8, 38.0, 32.7, 3.5, 3.4; data in accordance with the literature.[7]

tert-Butyl prop-2-yn-1-ylcarbamate 9f

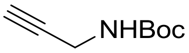

According to the modified procedure of Molander and Cadoret[8]: Di-*tert*-butyl dicarbonate (7.50 g, 34.4 mmol) was added portionwise to a stirring solution of propargylamine (2.2 mL, 1.9 g, 34 mmol) in CH_2Cl_2 (60 mL) at room temperature. The reaction mixture was stirred at room temperature for 1 h before the solvent was removed *in vacuo* to give the crude product, which was purified by recrystallization (diethyl ether/hexane) to give *tert*-butyl prop-2-yn-1-ylcarbamate **9f** as an off-white crystalline solid (4.98 g, 32.1 mmol, 93%); m.p. 32–34 °C (literature 41–42 °C)[8]; R_f = 0.29 (12:1 petrol:ethyl acetate); ν_{max} (film/cm^{-1}) 3306s (CC-H or N-H), 3281s (CC-H or N-H), 2980s (C-H), 1809m, 1682s (C=O), 1527s; ^{13}C NMR (500 MHz, $CDCl_3$) 4.85–4.69 (1H, br. s, N*H*), 3.92–3.81 (2H, br. s, C*H*$_2$N), 2.20 (1H, t, *J* = 2.5, C≡C-*H*), 1.43 (9H, s, C(C*H*$_3$)$_3$); ^{1}H NMR (500 MHz, $CDCl_3$) 155, 80.2, 71.3, 30.4, 28.4, 27.5; data in accordance with the literature.[8]

Ethyl 4-((trimethylsilyl)ethynyl)benzoate

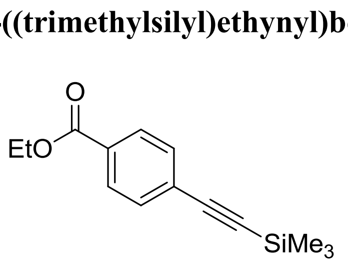

According to the modified procedure of Keana *et al.*[9]: Ethyl 4-iodobenzolate (0.43 mL, 700 mg, 2.5 mmol), ethynyltrimethylsilane (0.43 mL, 300 mg, 3.0 mmol), $Pd(PPh_3)_2Cl_2$ (53 mg, 0.076 mmol, 3.0 mol%) and CuI (29 mg, 0.15 mmol, 6.1 mol%) were added to a stirring degassed solution of triethylamine (1.3 mL, 0.95 g,

9.3 mmol) in THF (5.0 mL) in a sealed tube. The reaction mixture was stirred at 80 °C for 2 h before being allowed to cool. The reaction mixture was filtered and the residue washed with ethyl acetate. The solvent was removed *in vacuo* and the crude product was purified by flash column chromatography (40:1 petrol:ethyl acetate) to give ethyl 4-((trimethylsilyl)ethynyl)benzoate as a white crystalline solid (615 mg, 2.50 mmol, 100%); m.p. 24–26 °C (literature 30 °C)[8]; R_f = 0.29 (40:1 petrol:ethyl acetate); ν_{max} (film/cm^{-1}) 2961s (C-H), 2159s, 1718s (C=O), 1605s; ^{1}H NMR (600 MHz, DMSO-d$_6$) 7.98–7.95 (2H, m, Ar*H*), 7.52–7.49 (2H, m, Ar*H*), 4.36 (2H, q, *J* = 7.2, C*H*$_2$O), 1.38 (3H, t, *J* = 7.2, C*H*$_3$CH$_2$), 0.26 (9H, s, Si(C*H*$_3$)$_3$); ^{13}C NMR (125 MHz, DMSO-d$_6$) 166.1, 131.9, 130.1, 129.4, 127.7, 104.2, 97.6, 61.2, 14.4, -0.1; data in agreement with the literature.[10]

Methyl 4-ethynylbenzoate 9q

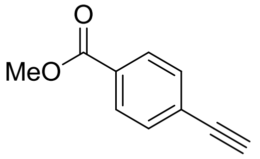

Potassium carbonate (345 mg, 2.50 mmol) was added to a stirring solution of ethyl 4-((trimethylsilyl)ethynyl)benzoate (615 mg, 2.50 mmol) in methanol (5.0 mL) at room temperature. The suspension was stirred for 30 minutes before the reaction mixture was filtered through a silica plug, eluting with ethyl acetate. The solvent was remove *in vacuo* and the crude product was purified by flash column chromatography (40:1 petrol:ethyl acetate) to give methyl 4-ethynylbenzoate **9q** as a white crystalline solid (366 mg, 2.28 mmol, 91%); m.p. 80–82 °C (literature 92–93 °C)[11]; R_f = 0.26 (40:1 petrol:ethyl acetate); ν_{max} (film/cm^{-1}) 3242s (CC-H), 2952m (C-H), 1700s (C=O), 1607s, 1434s; ^{1}H NMR (600 MHz, DMSO-d$_6$) 7.94 (2H, d, *J* = 8.3, Ar*H*), 7.61 (2H, d, *J* = 8.3, Ar*H*), 4.49 (1H, s, CC*H*), 3.85 (3H, s, C*H*$_3$O); ^{13}C NMR (150 MHz, DMSO-d$_6$) 165.6, 132.1, 129.6, 129.5, 126.5, 84.0, 82.6, 52.4; data in agreement with the literature.[11]

2,2,5,5,8,8-Hexamethyl-3,7-dioxa-2,8-disilanonane

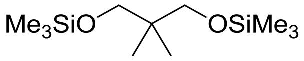

According to the procedure of Vaultier *et al.*[12]: 2-dimethylpropane-1,3-diol (5.00 g, 48.0 mmol) was added portionwise to a stirring mixture of bis(trimethylsilyl)amine (10 mL, 48 mmol) and chlorotrimethylsilane (1.2 mL, 9.6 mmol) at room temperature. The reaction was heated to 70 °C for 2 h before being left to cool to room temperature. Water (50 mL) and diethyl ether (50 mL) were added to the reaction mixture and the aqueous layer was extracted with diethyl ether (3 × 50 mL). The combined organic extracts were dried ($MgSO_4$) and the solvent removed *in vacuo* to give 2,2,5,5,8,8-hexamethyl-3,7-dioxa-2,8-disilanonane as a colourless oil (10.0 g, 40.3 mmol, 84%); R_f = 0.66 (100:1 petrol:ethyl acetate); ν_{max} (film/cm^{-1}) 2957s (C-H), 1476m; ^{1}H NMR (500 MHz, $CDCl_3$) 3.28 (4H, s, C*H*$_2$O), 0.80 (6H, s, C(C*H*$_3$)$_2$), 0.08 (18H, s, Si(C*H*$_3$)$_3$); ^{13}C NMR (125 MHz, $CDCl_3$) 67.9, 37.2, 21.4, -0.5; data in agreement with the literature.[12]

2-Ethynyl-2,3-dihydro-1*H*-naphtho[1,8-*de*][1,3,2]diazaborinine 9v

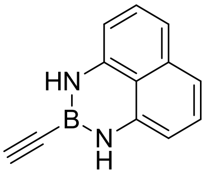

According to the modified procedure of Gandon *et al.*[13]: 2,2,5,5,8,8-Hexamethyl-3,7-dioxa-2,8-disilanonane (4.46 g, 17.9 mmol) and chlorotrimethylsilane (4.6 mL, 3.9 g, 36 mmol) were added dropwise to a stirring solution of potassium ethynyltrifluoroborate (2.50 g, 95% purity, 17.5 mmol) in dry acetone (22 mL) at room

temperature. The reaction was stirred at room temperature for 20 h before the reaction mixture was filtered and the solvent was removed from the filtrate *in vacuo*. The crude intermediate was dissolved in toluene (300 mL) and 1,8-diaminonapthaline (2.06 g, 13.0 mmol) was added portionwise to the stirring solution at room temperature. The reaction was heated under reflux for 24 h before it was allowed to cool to room temperature. The solvent was removed *in vacuo* to give the crude product, which was purified by flash column chromatography (6:1 petrol:ethyl acetate) to give 2-ethynyl-2,3-dihydro-1*H*-naphtho[1,8-*de*][1,3,2]diazaborinine **9v** as a white crystalline solid (0.950 g, 4.95 mmol, 38%); m.p. 87–89 °C (literature 92 °C)[13]; R_f = 0.26 (40:1 petrol:ethyl acetate); ν_{max} (film/cm^{-1}) 3426s (N-H), 3405s (N-H), 3241s (CC-H), 2076s, 1595s, 1500s, 1403s; ^{1}H NMR (600 MHz, DMSO-d$_6$) 8.30 (2H, s, N*H*), 7.03 (2H, ap. t, *J* = 8.1, Ar*H*), 6.88 (2H, d, *J* = 8.1, Ar*H*), 6.42 (2H, d, *J* = 8.1, Ar*H*), 3.42 (1H, s, C≡C-*H*); ^{13}C NMR (150 MHz, DMSO-d$_6$) 141.7, 135.9, 127.6, 119.9, 116.7, 105.5, 94.1, 85.4; data in agreement with the literature.[13]

2-Benzyl-5-butyl-7-(trimethylsilyl)isoindolin-1-one 10a

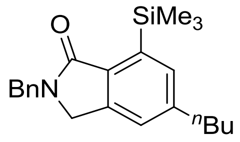

Prepared from *N*-benzyl-*N*-(prop-2-yn-1-yl)-3-(trimethylsilyl)propiolamide **6a**, 1-hexyne **9a** and Cp*RuCl(cod) **3** (3 mg, 3 mol%) over 16 h according to the General Alkyne Cyclotrimerization Procedure (crude ratio **10a**:**11** = 9:1) and purified by flash column chromatography (13:1 petrol:ethyl acetate) to give 2-benzyl-5-butyl-7-(trimethylsilyl)isoindolin-1-one **10a** as a colourless oil (75 mg, 0.21 mmol, 81%); R_f = 0.36 (6:1 petrol:ethyl acetate); ν_{max} (film/cm^{-1}) 2955m (C-H), 2930m (C-H), 1688s (C=O), 1454m, 1409m; ^{1}H NMR (600 MHz, DMSO-d$_6$) 7.34–7.21 (7H, m, Ar*H*), 4.68 (2H, s, C*H*$_2$N), 4.24 (2H, s, C*H*$_2$N), 2.60 (2H, t, *J* = 7.7, ArC*H*$_2$CH$_2$), 1.51 (2H, m, ArCH$_2$C*H*$_2$), 1.26 (2H, m, C*H*$_2$CH$_3$), 0.83 (3H, t, *J* = 7.4, CH$_2$C*H*$_3$), 0.34 (9H, s, Si(C*H*$_3$)$_3$); ^{13}C NMR (125 MHz, DMSO-d$_6$) 168.5, 144.8, 142.1, 137.7, 136.9, 134.3, 134.0, 128.6, 127.6, 127.2, 123.7, 48.9, 45.4, 35.1, 33.2, 21.8, 13.7, -0.4; HRMS (EI$^+$) found [M]$^+$ 351.2011; $C_{22}H_{29}NOSi$ requires 351.2013.

2-Benzyl-5-(*tert*-butyl)-7-(trimethylsilyl)isoindolin-1-one 10b

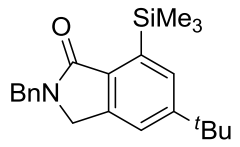

Prepared from *N*-benzyl-*N*-(prop-2-yn-1-yl)-3-(trimethylsilyl)propiolamide **6a**, 3,3-dimethyl-1-butyne **9b** and Cp*RuCl(cod) **3** (3 mg, 3 mol%) over 16 h according to the General Alkyne Cyclotrimerization Procedure (crude ratio **10b**:**11** = 2:1) and purified by flash column chromatography (12:1 petrol:ethyl acetate) to give 2-benzyl-5-(*tert*-butyl)-7-(trimethylsilyl)isoindolin-1-one **10b** as a white crystalline solid (61 mg, 0.17 mmol, 66%); m.p. 100–102 °C; R_f = 0.31 (12:1 petrol:ethyl acetate); ν_{max} (film/cm^{-1}) 2958s (C-H), 1689s (C=O), 1598m, 1410s; ^{1}H NMR (600 MHz, DMSO-d$_6$) 8.00 (1H, s, Ar*H*), 7.97 (1H, s, Ar*H*), 7.77–7.73 (2H, m, Ar*H*), 7.69–7.66 (3H, m, Ar*H*), 5.13 (2H, s, C*H*$_2$N), 4.72 (2H, s, C*H*$_2$N), 1.71 (9H, s, C(C*H*$_3$)$_3$), 0.79 (9H, s, Si(C*H*$_3$)$_3$); ^{13}C NMR (125 MHz, DMSO-d$_6$) 168.8, 153.2, 142.4, 138.1, 136.8, 134.5, 130.9, 129.0, 128.0, 127.6, 121.5, 49.5, 45.7, 31.5, 29.5, 0.0; HRMS (CI$^+$) found [M+H]$^+$ 352.2093; $C_{22}H_{30}NOSi$ requires 352.2097.

2-Benzyl-5-cyclopropyl-7-(trimethylsilyl)isoindolin-1-one 10c

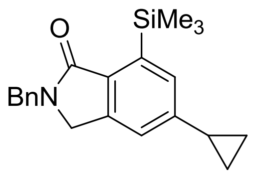

Prepared from *N*-benzyl-*N*-(prop-2-yn-1-yl)-3-(trimethylsilyl)propiolamide **6a**, cyclopropyl acetylene **9c** and Cp*RuCl(cod) **3** (3 mg, 3 mol%) over 16 h according to the General Alkyne Cyclotrimerization Procedure (crude ratio **10c**:**11** = 10:1) and purified by flash column chromatography (13:1 petrol:ethyl acetate) to give 2-benzyl-5-cyclopropyl-7-(trimethylsilyl)isoindolin-1-one **99** as a white crystalline solid (72 mg, 0.21 mmol, 81%); m.p. 66–68 °C; R_f = 0.31 (13:1 petrol:ethyl acetate); ν_{max} (film/cm^{-1}) 2951s (C-H), 1686s (C=O), 1600m, 1454s, 1410s; ^{1}H NMR (600 MHz, DMSO-d$_6$) 7.35–7.23 (6H, m, Ar*H*), 7.13 (1H, s, Ar*H*), 4.69 (2H, s, C*H*$_2$N), 4.25 (2H, s, C*H*$_2$N), 2.03–1.98 (1H, m, C*H*(CH$_2$)$_2$), 1.00–0.97 (2H, m, CH(C*H*H')$_2$), 0.73–0.69 (2H, m, CH(CH*H'*)$_2$), 0.35 (9H, s, Si(C*H*$_3$)$_3$); ^{13}C NMR (150 MHz, DMSO-d$_6$) 168.5, 146.5, 142.3, 137.7, 136.7, 134.0, 131.9, 128.7, 127.6, 127.3, 120.0, 48.9, 45.4, 15.5 , 10.1, -0.4; HRMS (CI$^+$) found [M+H]$^+$ 336.1784; $C_{21}H_{26}NOSi$ requires 336.1784.

2-Benzyl-5-cyclopentyl-7-(trimethylsilyl)isoindolin-1-one 10d

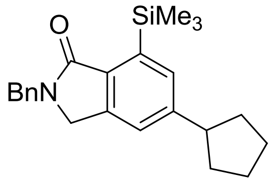

Prepared from *N*-benzyl-*N*-(prop-2-yn-1-yl)-3-(trimethylsilyl)propiolamide **6a**, cyclopentyl acetylene **9d** and Cp*RuCl(cod) **3** (3 mg, 3 mol%) over 16 h according to the General Alkyne Cyclotrimerization Procedure (crude ratio **10d**:**11** = 6:1) and purified by flash column chromatography (15:1 petrol:ethyl acetate) to give 2-benzyl-5-cyclopentyl-7-(trimethylsilyl)isoindolin-1-one **10d** as a colourless oil (78 mg, 0.21 mmol, 81%); R_f = 0.43 (15:1 petrol:ethyl acetate); ν_{max} (film/cm^{-1}) 2951s (C-H), 1686s (C=O), 1599m, 1453s, 1409s; ^{1}H NMR (600 MHz, DMSO-d$_6$) 7.45–7.23 (7H, m, Ar*H*), 4.70 (2H, s, C*H*$_2$N), 4.28 (2H, s, C*H*$_2$N), 3.06–3.00 (1H, m, C*H*(CH$_2$)$_2$), 2.03–1.98 (2H, m, CHC*H*H'), 1.78–1.72 (2H, m, CHCH$_2$C*H*H'), 1.66–1.59 (2H, m, CHCH$_2$CH*H'*), 1.56–1.49 (2H, m, CHCH*H'*), 0.35 (9H, s, Si(C*H*$_3$)$_3$); ^{13}C NMR (150 MHz, DMSO-d$_6$) 168.5, 148.6, 142.2, 137.7, 136.8, 134.5, 133.0, 128.7, 127.6, 127.3, 122.4, 49.0, 45.6, 45.4, 34.5, 25.1, -0.4; HRMS (CI$^+$) found [M+H]$^+$ 364.2091; $C_{23}H_{30}NOSi$ requires 364.2097.

2-Benzyl-5-(3-chloropropyl)-7-(trimethylsilyl)isoindolin-1-one 10e

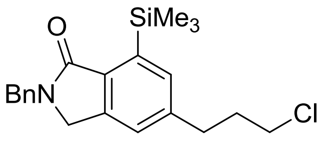

Prepared from *N*-benzyl-*N*-(prop-2-yn-1-yl)-3-(trimethylsilyl)propiolamide **6a**, 5-chloro-1-pentyne **9e** and Cp*RuCl(cod) **3** (3 mg, 3 mol%) over 16 h according to the General Alkyne Cyclotrimerization Procedure (crude ratio **10e**:**11** = 9:1) and purified by flash column chromatography (12:1 petrol:ethyl acetate) to give 2-benzyl-5-(3-chloropropyl)-7-(trimethylsilyl)isoindolin-1-one **10e** as a colourless oil (81 mg, 0.22 mmol, 83%); R_f = 0.31 (12:1 petrol:ethyl acetate); ν_{max} (film/cm^{-1}) 2951s (C-H), 1684s (C=O), 1600m, 1453s, 1409s; ^{1}H NMR (600 MHz, DMSO-d$_6$) 7.40 (1H, s, Ar*H*), 7.36 (1H, s, Ar*H*), 7.35–7.32 (2H, m, Ar*H*), 7.30–7.24 (3H, m, Ar*H*), 4.70 (2H, s, C*H*$_2$N), 4.29 (2H, s, C*H*$_2$N), 3.61 (2H, t, *J* = 6.5, C*H*$_2$Cl), 2.78 (2H, t, *J* = 7.6, C*H*$_2$CH$_2$CH$_2$Cl), 2.01 (2H, m, C*H*$_2$CH$_2$Cl), 0.36 (9H, s, Si(C*H*$_3$)$_3$); ^{13}C NMR (150 MHz, DMSO-d$_6$) 168.4, 143.3, 142.3, 137.7, 137.1,

134.7, 134.2, 128.7, 128.5, 127.3, 125.1, 49.0, 45.4, 44.7, 33.8, 32.5, -0.4; HRMS (CI$^+$) found $[M+H]^+$ 372.1548; $C_{21}H_{27}NOSiCl$ requires 372.1550.

***tert*-Butyl ((2-benzyl-1-oxo-7-(trimethylsilyl)isoindolin-5-yl)methyl)carbamate 10f**

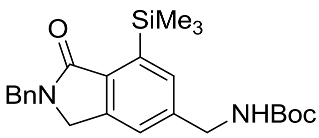

Prepared from *N*-benzyl-*N*-(prop-2-yn-1-yl)-3-(trimethylsilyl)propiolamide **6a**, *tert*-butyl prop-2-yn-1-ylcarbamate **9f** and Cp*RuCl(cod) **3** (5 mg, 5 mol%) over 24 h according to the General Alkyne Cyclotrimerization Procedure (crude ratio **10f**:**11** = 2:1) and purified by flash column chromatography (8:1 petrol:ethyl acetate) to give *tert*-butyl ((2-benzyl-1-oxo-7-(trimethylsilyl)isoindolin-5-yl)methyl)carbamate **10f** as a white crystalline solid (71 mg, 0.17 mmol, 63%); m.p. 124–126 °C; R_f = 0.44 (4:1 petrol:diethyl ether); ν_{max} (film/cm^{-1}) 3334s (N-H), 2975s (C-H), 1685s (lactam and carbamate C=O), 1516s, 1454s, 1411s; ^{1}H NMR (600 MHz, DMSO-d$_6$) a mixture of rotamers R_1 (major) and R_2 (minor) 7.51–7.45 (2H, m, Ar*H*, N*H* R_1; 2H, m, Ar*H*, N*H* R_2), 7.38–7.34 (3H, m, Ar*H* R_1; 3H, m, Ar*H* R_2), 7.30–7.24 (3H, m, Ar*H* R_1; 3H, m, Ar*H* R_2), 4.71 (2H, s, C*H*$_2$NCH$_2$ R_1; 2H, s, C*H*$_2$NCH$_2$ R_2), 4.32 (2H, s, C*H*$_2$NCH$_2$ R_1; 2H, s, C*H*$_2$NCH$_2$ R_2), 4.20 (2H, s, J = 6.2, C*H*$_2$NH R_1) 4.13 (2H, br. s, C*H*$_2$NH R_2) 1.39 (9H, s, C(C*H*$_3$)$_3$ R_1), 1.29 (9H, s, C(C*H*$_3$)$_3$ R_2), 0.35 (9H, s, Si(C*H*$_3$)$_3$ R_1; 9H, s, Si(C*H*$_3$)$_3$ R_2); ^{13}C NMR (150 MHz, DMSO-d$_6$) 168.4, 155.9, 142.7, 142.1, 137.7, 136.9, 135.2, 132.6, 128.7, 127.7, 127.3, 122.6, 77.9, 49.1, 45.5, 43.5, 28.3, -0.4; HRMS (ES$^+$) found $[M+H]^+$ 425.2274; $C_{24}H_{33}N_2O_3Si$ requires 425.2260.

2-Benzyl-5-(methoxymethyl)-7-(trimethylsilyl)isoindolin-1-one 10g

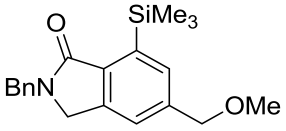

Prepared from *N*-benzyl-*N*-(prop-2-yn-1-yl)-3-(trimethylsilyl)propiolamide **6a**, 3-methoxy-1-propyne **9g** and Cp*RuCl(cod) **3** (3 mg, 3 mol%) over 16 h according to the General Alkyne Cyclotrimerization Procedure (crude ratio **10g**:**11** = 3:2) and purified by flash column chromatography (12:1 petrol:ethyl acetate) to give 2-benzyl-5-(methoxymethyl)-7-(trimethylsilyl)isoindolin-1-one **10g** as a white crystalline solid (50 mg, 0.15 mmol, 56%); m.p. 57–59 °C; R_f = 0.26 (12:1 petrol:ethyl acetate); ν_{max} (film/cm^{-1}) 2896s (C-H), 1684s (C=O), 1453s, 1409s; ^{1}H NMR (600 MHz, DMSO-d$_6$) 7.48 (s, 2H, Ar*H*), 7.37–7.33 (2H, m, Ar*H*), 7.29–7.26 (3H, m, Ar*H*), 4.71 (2H, s, C*H*$_2$N), 4.49 (2H, s, C*H*$_2$O), 4.33 (2H, s, C*H*$_2$N), 3.31 (3H, s, OC*H*$_3$), 0.36 (9H, s, Si(C*H*$_3$)$_3$); ^{13}C NMR (150 MHz, DMSO-d$_6$) 168.4, 142.1, 140.7, 137.6, 137.0, 135.9, 132.9, 128.7, 127.7, 127.4, 123.1, 73.5, 57.8, 49.1, 45.5, -0.4; HRMS (ESI$^+$) found $[M]^+$ 339.1647; $C_{20}H_{25}O_2NSi$ requires 339.1649.

2-Benzyl-5-(diethoxymethyl)-7-(trimethylsilyl)isoindolin-1-one 10h

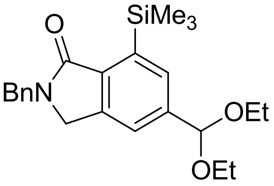

Prepared from *N*-benzyl-*N*-(prop-2-yn-1-yl)-3-(trimethylsilyl)propiolamide **6a**, 3,3-diethoxyprop-1-yne **9h** and Cp*RuCl(cod) **3** (3 mg, 3 mol%) over 24 h according to the General Alkyne Cyclotrimerization Procedure (crude ratio **10h**:**11** = 0.8:1) and purified by flash column chromatography (17:1 petrol:ethyl acetate) to give 2-benzyl-5-

(diethoxymethyl)-7-(trimethylsilyl)isoindolin-1-one **10h** (45 mg, 0.11 mmol, 43%) as a colourless oil; R_f = 0.27 (17:1 petrol:ethyl acetate); ν_{max} (film/cm^{-1}) 2874s (C-H), 1689s (C=O), 1454s, 1409s; ^{1}H NMR (600 MHz, DMSO-d$_6$) 7.59 (1H, s, Ar*H*), 7.57 (1H, s, Ar*H*), 7.37–7.26 (5H, m, Ar*H*), 5.56 (1H, s, C*H*O$_2$), 4.72 (2H, s, C*H*$_2$N), 4.36 (2H, s, C*H*$_2$N), 3.59–3.53 (2H, m, OC*H*H'), 3.32–3.46 (2H, m, OCH*H*'), 1.14 (6H, t, *J* = 7.0, CH$_2$C*H*$_3$), 0.36 (9H, s, Si(C*H*$_3$)$_3$); ^{13}C NMR (150 MHz, DMSO-d$_6$) 168.3, 141.9, 141.3, 137.6, 136.8, 136.6, 132.0, 128.7, 127.7, 127.4, 122.4, 100.9, 61.1, 49.2, 45.5, 15.2, -0.4; HRMS (ES$^+$) found [M+Na]$^+$ 420.1970; $C_{23}H_{31}NO_3NaSi$ requires 420.1971.

2-Benzyl-5-phenyl-7-(trimethylsilyl)isoindolin-1-one 10k

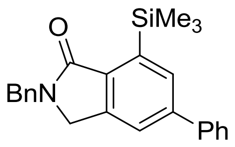

Prepared from *N*-benzyl-*N*-(prop-2-yn-1-yl)-3-(trimethylsilyl)propiolamide **6a**, phenylacetylene **9k** and Cp*RuCl(cod) **3** (4 mg, 4 mol%) over 24 h according to the General Alkyne Cyclotrimerization Procedure (crude ratio **10k**:**11** = 6:1) and purified by flash column chromatography (15:1 petrol:ethyl acetate) to give 2-benzyl-5-phenyl-7-(trimethylsilyl)isoindolin-1-one **10k** as a white crystalline solid (81 mg, 0.22 mmol, 83%); m.p. 117–119 °C; R_f = 0.25 (15:1 petrol:ethyl acetate); ν_{max} (film/cm^{-1}) 2950s (C-H), 1687s (C=O), 1597m, 1454s, 1409s; ^{1}H NMR (600 MHz, DMSO-d$_6$) 7.80 (1H, s, Ar*H*), 7.75 (1H, d, *J* = 1.3, Ar*H*), 7.69–7.66 (2H, m, Ar*H*), 7.50 (2H, t, *J* = 4.8, Ar*H*), 7.42–7.35 (3H, m, Ar*H*), 7.30–7.27 (3H, m, Ar*H*), 4.75 (2H, s, C*H*$_2$N), 4.40 (2H, s, C*H*$_2$N), 0.41 (9H, s, Si(C*H*$_3$)$_3$); ^{13}C NMR (150 MHz, DMSO-d$_6$) 168.3, 142.7, 142.2, 140.1, 137.8, 137.6, 135.7, 132.6, 129.1, 128.8, 128.0, 127.7, 127.4, 127.2, 127.8, 49.2, 45.5, -0.4; HRMS (CI$^+$) found [M+H]$^+$ 372.1779; $C_{24}H_{26}NOSi$ requires 372.1784.

2-Benzyl-5-*o*-tolyl-7-(trimethylsilyl)isoindolin-1-one 10l

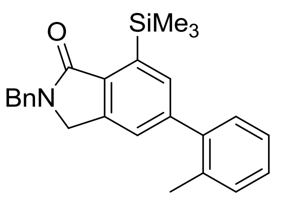

Prepared from *N*-benzyl-*N*-(prop-2-yn-1-yl)-3-(trimethylsilyl)propiolamide **6a**, 2-ethynyltoluene **9l** and Cp*RuCl(cod) **3** (3 mg, 3 mol%) over 16 h according to the General Alkyne Cyclotrimerization Procedure (crude ratio **10l**:**11** = >10:1) and purified by flash column chromatography (17:1 petrol:ethyl acetate) to give 2-benzyl-5-*o*-tolyl-7-(trimethylsilyl)isoindolin-1-one **10l** as a colourless oil (95 mg, 0.25 mmol, 93%); R_f = 0.28 (17:1 petrol:ethyl acetate); ν_{max} (film/cm^{-1}) 2956s (C-H), 1689s (C=O), 1598m, 1454s, 1410s; ^{1}H NMR (600 MHz, DMSO-d$_6$) 7.52 (1H, s, Ar*H*), 7.45 (1H, s, Ar*H*), 7.38–7.21 (9H, m, Ar*H*), 4.74 (2H, s, C*H*$_2$N), 4.38 (2H, s, C*H*$_2$N), 2.22 (3H, s, ArC*H*$_3$), 0.38 (9H, s, Si(C*H*$_3$)$_3$); ^{13}C NMR (150 MHz, DMSO-d$_6$) 168.3, 143.1, 142.0, 140.9, 137.6, 136.9, 135.3, 134.7, 134.6, 130.5, 129.6, 128.8, 127.8, 127.7, 127.4, 126.1, 124.8, 49.2, 45.5, 20.2, -0.4; HRMS (CI$^+$) found [M+H]$^+$ 386.1939; $C_{25}H_{28}NOSi$ requires 386.1935.

2-Benzyl-5-(*p*-tolyl)-7-(trimethylsilyl)isoindolin-1-one 10m

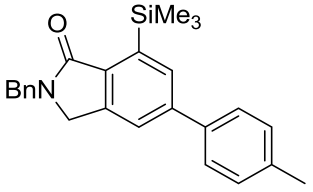

Prepared from *N*-benzyl-*N*-(prop-2-yn-1-yl)-3-(trimethylsilyl)propiolamide **6a**, 4-ethynyltoluene **9m** and Cp*RuCl(cod) **3** (4 mg, 4 mol%) over 24 h according to the General Alkyne Cyclotrimerization Procedure (crude ratio **10m**:**11** = 6:1) and purified by flash column chromatography (15:1 petrol:ethyl acetate) to give 2-benzyl-5-(*p*-tolyl)-7-(trimethylsilyl)isoindolin-1-one **10m** as a white crystalline solid (82 mg, 0.22 mmol, 81%); m.p. 78–80 °C; R_f = 0.50 (6:1 petrol:ethyl acetate); ν_{max} (film/cm^{-1}) 2950s (C-H), 1686s (C=O), 1453s, 1409s; ^{1}H NMR (600 MHz, DMSO-d$_6$) 7.76 (1H, s, Ar*H*), 7.73–7.72 (1H, m, Ar*H*), 7.58–7.56 (2H, m, Ar*H*), 7.38–7.34 (2H, m, Ar*H*), 7.30–7.27 (5H, m, Ar*H*), 4.74 (2H, s, CH_2N), 4.38 (2H, s, CH_2N), 2.34 (3H, s, ArCH_3), 0.40 (9H, s, Si(CH_3)$_3$); ^{13}C NMR (150 MHz, DMSO-d$_6$) 168.3, 142.7, 142.1, 137.7, 137.6, 137.4, 137.1, 135.4, 132.3, 129.7, 128.8, 127.7, 127.3, 127.0, 122.4, 49.2, 45.5, 20.7, -0.4; HRMS (ESI$^+$) found [M-Me]$^+$ 370.1620; $C_{24}H_{24}NOSi$ requires 370.1627.

2-Benzyl-5-(2-bromophenyl)-7-(trimethylsilyl)isoindolin-1-one 10n

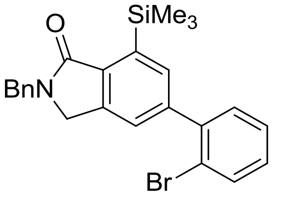

Prepared from *N*-benzyl-*N*-(prop-2-yn-1-yl)-3-(trimethylsilyl)propiolamide **6a**, 1-bromo-2-ethynylbenzene **9m** and Cp*RuCl(cod) **3** (3 mg, 3 mol%) over 16 h according to the General Alkyne Cyclotrimerization Procedure (crude ratio **10n**:**11** = 8:1) and purified by flash column chromatography (20:1 petrol:ethyl acetate) to give 2-benzyl-5-(2-bromophenyl)-7-(trimethylsilyl)isoindolin-1-one **10n** as a colourless oil (95 mg, 0.21 mmol, 80%); R_f = 0.37 (15:1 petrol:ethyl acetate); ν_{max} (film/cm^{-1}) 2974s (C-H), 1688s (C=O), 1602m, 1452s, 1409s; ^{1}H NMR (600 MHz, DMSO-d$_6$) 7.74 (1H, dd, *J* = 8.0, 0.8, Ar*H*), 7.56 (2H, d, *J* = 2.9, Ar*H*), 7.47–7.45 (1H, m, Ar*H*), 7.42–7.40 (1H, m, Ar*H*), 7.37–7.26 (6H, m, Ar*H*), 4.75 (2H, s, CH_2N), 4.38 (2H, s, CH_2N), 0.38 (9H, s, Si(CH_3)$_3$); ^{13}C NMR (150 MHz, DMSO-d$_6$) 168.2, 142.1, 141.8, 141.4, 137.6, 136.7, 135.9, 134.9, 133.2, 131.5, 129.8, 128.7, 128.2, 127.8, 127.4, 125.0, 121.6, 49.2, 45.5, -0.4; HRMS (CI$^+$) found [M+H]$^+$ 450.0870; $C_{24}H_{25}BrNOSi$ requires 450.0889.

2-Benzyl-5-(4-bromophenyl)-7-(trimethylsilyl)isoindolin-1-one 10o

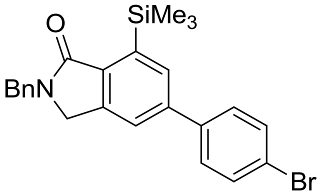

Prepared from *N*-benzyl-*N*-(prop-2-yn-1-yl)-3-(trimethylsilyl)propiolamide **6a**, 1-bromo-4-ethynylbenzene **9o** and Cp*RuCl(cod) **3** (3 mg, 3 mol%) over 24 h according to the General Alkyne Cyclotrimerization Procedure (crude ratio **10o**:**11** = 5:1) and purified by flash column chromatography (12:1 petrol:ethyl acetate) to give 2-benzyl-5-(4-bromophenyl)-7-(trimethylsilyl)isoindolin-1-one **10o** as a colourless oil (98 mg, 0.22 mmol, 83%); R_f = 0.20 (15:1 petrol:ethyl acetate); ν_{max} (film/cm^{-1}) 2950s (C-H), 1684s (C=O), 1600s, 1495s, 1453s, 1409s;

^{1}H NMR (600 MHz, DMSO-d$_6$) 7.77 (1H, s, Ar*H*), 7.72 (1H, s, Ar*H*), 7.29–7.26 (3H, m, Ar*H*), 7.37–7.33 (2H, m, Ar*H*), 7.66–7.60 (4H, m, Ar*H*), 4.74 (2H, s, C*H*$_2$N), 4.37 (2H, s, C*H*$_2$N), 0.40 (9H, s, Si(C*H*$_3$)$_3$); ^{13}C NMR (150 MHz, DMSO-d$_6$) 168.2, 142.7, 140.8, 139.2, 137.9, 137.6.9, 136.0, 132.4, 132.0, 129.3, 128.7, 127.7, 127.4, 122.6, 121.6, 49.2, 46.2, -0.4; HRMS (CI$^+$) found [M+H]$^+$ 450.0877; $C_{24}H_{24}BrNOSi$ requires 450.0889.

Methyl 4-(2-benzyl-1-oxo-7-(trimethylsilyl)isoindolin-5-yl)benzoate 10p

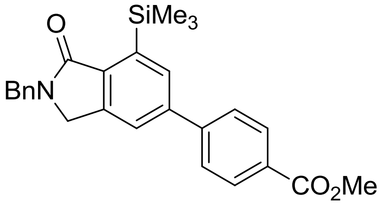

Prepared from *N*-benzyl-*N*-(prop-2-yn-1-yl)-3-(trimethylsilyl)propiolamide **6a**, methyl 4-ethynylbenzoate **10p** and Cp*RuCl(cod) **3** (3 mg, 3 mol%) over 24 h according to the General Alkyne Cyclotrimerization Procedure (crude ratio **10p**:**11** = 5:1) and purified by flash column chromatography (15:1 petrol:ethyl acetate) to give methyl 4-(2-benzyl-1-oxo-7-(trimethylsilyl)isoindolin-5-yl)benzoate **10p** as a colourless oil (89 mg, 0.21 mmol, 79%); R_f = 0.29 (9:1 petrol:ethyl acetate); ν_{max} (film/cm^{-1}) 2951s (C-H), 1720s (ester C=O) 1686s (lactam C=O), 1608s, 1434s, 1410s; ^{1}H NMR (600 MHz, DMSO-d$_6$) 8.05 (1H, s, Ar*H*), 8.03 (1H, s, Ar*H*), 7.83–7.77 (4H, m, Ar*H*), 7.36–7.32 (2H, m, Ar*H*), 7.29–7.26 (3H, m, Ar*H*), 4.73 (2H, s, C*H*$_2$N), 4.37 (2H, s, C*H*$_2$N), 3.89 (3H, s, OC*H*$_3$)), 0.41 (9H, s, Si(C*H*$_3$)$_3$); ^{13}C NMR (150 MHz, DMSO-d$_6$) 168.1, 166.0, 144.5, 142.7, 140.8, 138.0, 137.5, 136.5, 132.7, 129.9, 128.9, 128.7, 127.7, 127.5, 127.4, 123.1, 52.2, 49.2, 45.5, -0.4; HRMS (ES$^+$) found [M+Na]$^+$ 452.1642; $C_{26}H_{27}O_3NSiNa$ requires 452.1658.

2-Benzyl-5-(4-methoxyphenyl)-7-(trimethylsilyl)isoindolin-1-one 10q

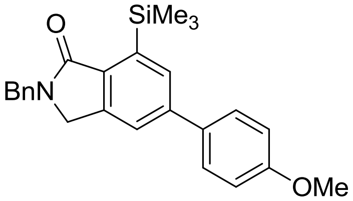

Prepared from *N*-benzyl-*N*-(prop-2-yn-1-yl)-3-(trimethylsilyl)propiolamide **6a**, 1-ethynyl-4-methoxybenzene **9q** and Cp*RuCl(cod) **3** (5 mg, 5 mol%) over 24 h according to the General Alkyne Cyclotrimerization Procedure (crude ratio **10q**:**11** = 6:1) and purified by flash column chromatography (12:1 petrol:ethyl acetate) to give 2-benzyl-5-(4-methoxyphenyl)-7-(trimethylsilyl)isoindolin-1-one **10q** (83 mg, 0.21 mmol, 79%) as a colourless oil; R_f = 0.28 (15:1 petrol:ethyl acetate); ν_{max} (film/cm^{-1}) 2952s (C-H), 1685s (C=O), 1606s 1516s, 1454s, 1409s; ^{1}H NMR (600 MHz, DMSO-d$_6$) 7.73 (1H, s, Ar*H*), 7.71 (1H, s, Ar*H*), 7.61 (2H, d, *J* = 8.7, Ar*H*), 7.36–7.33 (2H, m, Ar*H*), 7.28–7.26 (3H, m, Ar*H*), 7.04 (2H, d, *J* = 8.7 Ar*H*), 4.72 (2H, s, C*H*$_2$N), 4.35 (2H, s, C*H*$_2$N), 3.78 (3H, s, OC*H*$_3$), 0.40 (9H, s, Si(C*H*$_3$)$_3$); ^{13}C NMR (150 MHz, DMSO-d$_6$) 168.4, 159.3, 142.7, 141.8, 137.6, 137.6, 135.0, 132.3, 132.1, 128.7, 128.3, 127.7, 127.4, 122.1, 114.5, 55.2, 49.1, 45.5, -0.4; HRMS (ES$^+$) found [M+Na]$^+$ 424.1694; $C_{25}H_{27}NO_2NaSi$ requires 424.1709.

2-Benzyl-5-(4-(dimethylamino)phenyl)-7-(trimethylsilyl)isoindolin-1-one 10r

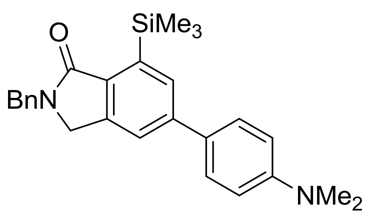

Prepared from *N*-benzyl-*N*-(prop-2-yn-1-yl)-3-(trimethylsilyl)propiolamide **6a**, 4-ethynyl-*N*,*N*-dimethylaniline **9r** and Cp*RuCl(cod) **3** (10 mg, 10 mol%) over 24 h according to the General Alkyne Cyclotrimerization Procedure (crude ratio **10r**:**11** = 7:1)and purified by flash column chromatography (10:1 petrol:ethyl acetate) to give 2-benzyl-5-(4-(dimethylamino)phenyl)-7-(trimethylsilyl)isoindolin-1-one **10r** (86 mg, 0.21 mmol, 79%) as a colourless oil; $R_f = 0.27$ (10:1 petrol:ethyl acetate); ν_{max} (film/cm^{-1}) 2897s (C-H), 1682s (C=O), 1609s 1592s 1526s, 1452s, 1409s; ^{1}H NMR (600 MHz, DMSO-d$_6$) 7.70 (2H, s, Ar*H*), 7.53 (2H, d, *J* = 8.8, Ar*H*), 7.37–7.34 (2H, m, Ar*H*), 7.30–7.26 (3H, m, Ar*H*), 6.81 (2H, d, *J* = 8.8 Ar*H*), 4.72 (2H, s, C*H*$_2$N), 4.35 (2H, s, C*H*$_2$N), 2.93 (6H, s, N(C*H*$_3$)$_2$)), 0.40 (9H, s, Si(C*H*$_3$)$_3$); ^{13}C NMR (150 MHz, DMSO-d$_6$) 168.5, 150.2, 142.7, 142.3, 137.7, 137.4, 134.2, 131.4, 128.7, 127.7, 127.7, 127.4, 127.1, 121.2, 112.7, 49.2, 45.5, 39.9, -0.3; HRMS (ES$^+$) found [M+H]$^+$ 415.2219; $C_{26}H_{31}N_2OSi$ requires 415.2206.

2-Benzyl-5-(pyridin-2-yl)-7-(trimethylsilyl)isoindolin-1-one 10t

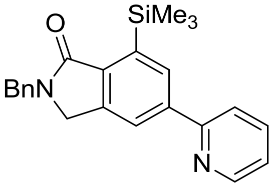

Prepared from *N*-benzyl-*N*-(prop-2-yn-1-yl)-3-(trimethylsilyl)propiolamide **6a**, 2-ethynylpyridine **10t** and Cp*RuCl(cod) **3** (20 mg, 20 mol%) over 24 h according to the General Alkyne Cyclotrimerization Procedure (crude ratio **10t**:**11** = 2:1) and purified by flash column chromatography (3:1 petrol:diethyl ether) to give 2-benzyl-5-(pyridin-2-yl)-7-(trimethylsilyl)isoindolin-1-one **10t** as a colourless oil (49 mg, 0.13 mmol, 50%); $R_f = 0.40$ (1:1 petrol:diethyl ether); ν_{max} (film/cm^{-1}) 2973s (C-H), 1686s (C=O), 1587s, 1409s; ^{1}H NMR (600 MHz, DMSO-d$_6$) 8.71–8.70 (1H, m, Ar*H*), 8.26–8.25 (1H, m, Ar*H*), 8.20–8.20 (1H, m, Ar*H*), 7.99–7.98 (1H, m, Ar*H*), 7.93–7.89 (1H, m, Ar*H*), 7.40–7.35 (3H, m, Ar*H*), 7.31–7.27 (3H, m, Ar*H*), 4.75 (2H, s, C*H*$_2$N), 4.42 (2H, s, C*H*$_2$N), 0.42 (9H, s, Si(C*H*$_3$)$_3$); ^{13}C NMR (150 MHz, DMSO-d$_6$) 168.2, 155.7, 149.8, 142.5, 140.3, 137.6, 137.5, 137.5, 137.1, 132.3, 128.8, 127.7, 127.4, 123.1, 122.5, 121.0, 49.3, 45.6, -0.3. HRMS (CI$^+$) found [M+H]$^+$ 371.1708; $C_{23}H_{25}N_2OSi$ requires 373.1731.

2-Benzyl-5-(1*H*-naphtho[1,8-*de*][1,3,2]diazaborinin-2(3*H*)-yl)-7-(trimethylsilyl)isoindolin-1-one 10v

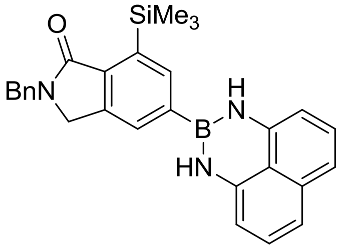

Prepared from *N*-benzyl-*N*-(prop-2-yn-1-yl)-3-(trimethylsilyl)propiolamide **6a**, 2-ethynyl-2,3-dihydro-1*H*-naphtho[1,8-*de*][1,3,2]diazaborinine **9v** and Cp*RuCl(cod) **3** (5 mg, 5 mol%) over 24 h according to the General

Alkyne Cyclotrimerization Procedure (crude ratio **10v**:**11** = 3:1) and purified by flash column chromatography (3:1 petrol:diethyl ether) to give 2-benzyl-5-(1*H*-naphtho[1,8-*de*][1,3,2]diazaborinin-2(3*H*)-yl)-7-(trimethylsilyl)isoindolin-1-one **10v** as a white crystalline solid (67 mg, 0.15 mmol, 55%); m.p. 96–98 °C; R_f = 0.56 (2:3 petrol:diethyl ether); ν_{max} (film/cm^{-1}) 3333s (N-H), 2952s (C-H), 1672s (C=O), 1599s, 1512s, 1405s; ^{1}H NMR (600 MHz, DMSO-d$_6$) 8.35 (2H, s, N*H*), 8.02 (1H, s, Ar*H*), 7.96 (1H, s, Ar*H*), 7.39–7.36 (2H, m, Ar*H*), 7.31–7.28 (3H, m, Ar*H*), 7.09 (2H, t, *J* = 7.8, Ar*H*), 6.91 (2H, d, *J* = 8.1, Ar*H*) 6.59 (2H, d, *J* = 7.2, Ar*H*), 4.76 (2H, s, C*H*$_2$N), 4.42 (2H, s, C*H*$_2$N), 0.44 (9H, s, Si(C*H*$_3$)$_3$); ^{13}C NMR (150 MHz, DMSO-d$_6$) 168.6, 142.3, 140.8, 138.0, 137.9, 137.7, 136.0, 136.0, 128.8, 128.4, 127.7, 127.7, 127.4, 119.8, 116.4, 105.8, 49.2, 45.5, -0.1, N_2BC not observed; HRMS (EI$^+$) found [M]$^+$ 461.2089; $C_{28}H_{28}BN_3OSi$ requires 461.2089.

N-Benzyl-*N*-((2-benzyl-1-oxo-7-(trimethylsilyl)isoindolin-5-yl)methyl)-3-(trimethylsilyl)propiolamide 11

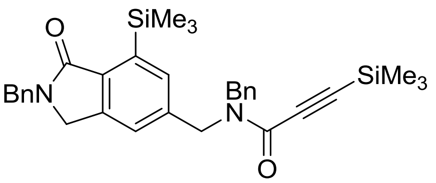

Isolated from the certain alkyne cyclotrimerization reaction involving *N*-benzyl-*N*-(prop-2-yn-1-yl)-3-(trimethylsilyl)propiolamide **6a**.

N-benzyl-*N*-((2-benzyl-1-oxo-7-(trimethylsilyl)isoindolin-5-yl)methyl)propiolamide **11** was isolated as a colourless oil; R_f = 0.24 (6:1 petrol:ethyl acetate); ν_{max} (film/cm^{-1}) 3279s (CC-H), 2960w (C-H), 2108w, 1630s (C=O), 1415s; ^{1}H NMR (600 MHz, DMSO-d$_6$) a 1:1 mixture of rotamers; 7.46–7.17 (12H, m, Ar*H*; 12H, m, Ar*H*), 4.79 (2H, s, C*H*$_2$N), 4.73 (2H, s, C*H*$_2$N), 4.72 (2H, s, C*H*$_2$N), 4.71 (2H, s, C*H*$_2$N), 4.55 (2H, s, C*H*$_2$N), 4.47 (2H, s, C*H*$_2$N), 4.32 (2H, s, C*H*$_2$N), 4.30 (2H, s, C*H*$_2$N), 0.34 (9H, s, Si(C*H*$_3$)$_3$), 0.33 (9H, s, Si(C*H*$_3$)$_3$), 0.17 (9H, s, Si(C*H*$_3$)$_3$), 0.13 (9H, s, Si(C*H*$_3$)$_3$); ^{13}C NMR (125 MHz, DMSO-d$_6$) a mixture of rotamers; 168.2, 168.2, 153.3, 153.3, 142.3, 138.9, 138.7, 137.6, 137.6, 137.4, 137.2, 136.5, 136.5, 136.0, 135.7, 133.6, 133.0, 128.7, 128.7, 128.5, 127.9, 127.7, 127.7, 127.7, 127.5, 127.4, 127.4, 123.4, 123.3, 97.4, 97.4, 96.4, 51.9, 51.9, 49.0, 47.3, 47.3, 45.4, -0.4, -0.4, -1.0, -1.0; HRMS (EI$^+$) found [M]$^+$ 539.2548; $C_{32}H_{39}N_2O_2Si_2$ requires 539.2550.

2-(*tert*-Butyl)-5-butyl-7-(trimethylsilyl)isoindolin-1-one 13a

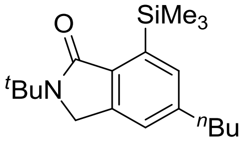

Prepared from *N*-(*tert*-butyl)-*N*-(prop-2-yn-1-yl)-3-(trimethylsilyl)propiolamide **6b**, 1-hexyne **9a** and Cp*RuCl(cod) **3** (3 mg, 3 mol%) over 16 h according to the General Alkyne Cyclotrimerization Procedure (crude ratio **13a**:**14a** = 10:1) and purified by flash column chromatography (30:1 petrol:ethyl acetate) to give 2-(*tert*-butyl)-5-butyl-7-(trimethylsilyl)isoindolin-1-one **13a** as a colourless oil (70 mg, 0.22 mmol, 84%); R_f = 0.36 (30:1 ethyl acetate:petrol); ν_{max} (film/cm^{-1}) 2957s (C-H), 1681s (C=O), 1455s; ^{1}H NMR (600 MHz, DMSO-d$_6$) 7.32 (1H, s, Ar*H*), 7.31 (1H, s, Ar*H*), 4.48 (2H, s, C*H*$_2$N), 2.65 (2H, t, *J* = 7.7 ArC*H*$_2$CH$_2$), 1.57–1.52 (2H, m, ArCH$_2$C*H*$_2$), 1.47 (9H, s, C(C*H*$_3$)$_3$) 1.30 (2H, ap. sextet, *J* = 7.3, C*H*$_2$CH$_3$), 0.89 (3H, t, *J* = 7.3, CH$_2$C*H*$_3$), 0.31 (9H, s, Si(C*H*$_3$)$_3$); ^{13}C NMR (150 MHz, DMSO-d$_6$) 168.9, 144.3, 141.7, 136.3, 136.0, 134.0, 123.4, 53.5, 47.8, 35.1, 33.4, 27.5, 21.8, 13.8, -0.2; HRMS (CI$^+$) found [M+H]$^+$ 318.2252; $C_{19}H_{32}NOSi$ requires 318.2248.

2-(*tert*-Butyl)-5-phenyl-7-(trimethylsilyl)isoindolin-1-one 13b

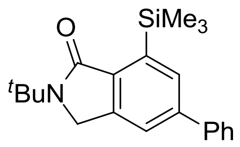

Prepared from *N*-(*tert*-butyl)-*N*-(prop-2-yn-1-yl)-3-(trimethylsilyl)propiolamide **6b**, phenylacetylene **9k** and Cp*RuCl(cod) **3** (4 mg, 4 mol%) over 24 h according to the General Alkyne Cyclotrimerization Procedure (crude ratio **13b**:**14a** = >10:1) and purified by flash column chromatography (35:1 petrol:ethyl acetate) to give 2-(*tert*-butyl)-5-phenyl-7-(trimethylsilyl)isoindolin-1-one **13b** as a white crystalline solid (79 mg, 0.23 mmol, 89%); m.p. 73–75 °C; R_f = 0.36 (30:1 ethyl acetate: petrol); ν_{max} (film/cm^{-1}) 2963s (C-H), 1677s (C=O), 1448s; ^{1}H NMR (600 MHz, DMSO-d$_6$) 7.78 (1H, s, Ar*H*), 7.72–7.70 (1H, m, Ar*H*), 7.69–7.67 (2H, m, Ar*H*), 7.50 (2H, t, *J* = 9.3, Ar*H*), 7.43–7.39 (1H, m, Ar*H*), 4.60 (2H, s, C*H*$_2$N), 1.51 (9H, s, C(C*H*$_3$)$_3$), 0.37 (9H, s, Si(C*H*$_3$)$_3$); ^{13}C NMR (150 MHz, DMSO-d$_6$) 168.6, 142.2, 141.8, 140.2, 137.7, 137.0, 132.4, 129.1, 127.9, 127.2, 122.2, 53.7, 48.1, 27.5, -0.3; HRMS (ES$^+$) found [M+H]$^+$ 338.1940; $C_{21}H_{28}NOSi$ requires 338.1940.

2-(*tert*-Butyl)-5-(o-tolyl)-7-(trimethylsilyl)isoindolin-1-one 13c

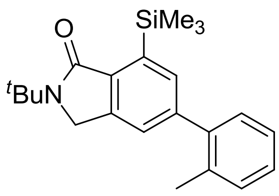

Prepared from *N*-(*tert*-butyl)-*N*-(prop-2-yn-1-yl)-3-(trimethylsilyl)propiolamide **6b**, 2-ethynyltoluene **9l** and Cp*RuCl(cod) **3** (3 mg, 3 mol%) over 16 h according to the General Alkyne Cyclotrimerization Procedure (crude ratio **13c**:**14a** >10:1) and purified by flash column chromatography (20:1 petrol:ethyl acetate) to give 2-(*tert*-butyl)-5-(*o*-tolyl)-7-(trimethylsilyl)isoindolin-1-one **13c** as a yellow oil (87 mg, 0.25 mmol, 94%); R_f = 0.31 (20:1 ethyl acetate: petrol); ν_{max} (film/cm^{-1}) 2973s (C-H), 1680s (C=O), 1452s; ^{1}H NMR (600 MHz, DMSO-d$_6$) 7.48 (1H, s, Ar*H*), 7.40 (1H, s, Ar*H*), 7.31–7.21 (3H, m, Ar*H*), 7.21–7.18 (1H, m, Ar*H*), 4.58 (2H, s, C*H*$_2$N), 2.21 (3H, s, ArC*H*$_3$), 1.49 (9H, s, C(C*H*$_3$)$_3$), 0.33 (9H, s, Si(C*H*$_3$)$_3$); ^{13}C NMR (150 MHz, DMSO-d$_6$) 168.6, 142.9, 141.5, 141.1, 137.3, 136.1, 134.7, 134.4, 130.5, 129.6, 127.6, 126.0, 124.3, 53.6, 48.0, 27.4, 20.2, -0.3; HRMS (CI$^+$) found [M+H]$^+$ 352.2088; $C_{22}H_{30}NOSi$ requires 352.2097.

5-Butyl-7-(trimethylsilyl)isoindolin-1-one 13d

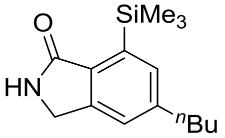

Prepared from *N*-(prop-2-yn-1-yl)-3-(trimethylsilyl)propiolamide **6c**, 1-hexyne **9a** and Cp*RuCl(cod) **3** (10 mg, 10 mol%) over 24 h according to the General Alkyne Cyclotrimerization Procedure (crude ratio **13d**:**14b** = 2:1) and purified by flash column chromatography (5:1 petrol:ethyl acetate) to give 5-butyl-7-(trimethylsilyl)isoindolin-1-one **13d** as a white crystalline solid (35 mg, 0.13 mmol, 51%); m.p. 140–142 °C; R_f = 0.57 (2:1 petrol:ethyl acetate); ν_{max} (film/cm^{-1}) 3307m (N-H), 2968s (C-H), 1690s (C=O); ^{1}H NMR (600 MHz, DMSO-d$_6$) 8.37 (1H, s, N*H*), 7.36 (1H, s, Ar*H*), 7.33 (1H, s, Ar*H*), 4.30 (2H, s, C*H*$_2$N), 2.66 (2H, t, *J* = 7.7, ArC*H*$_2$CH$_2$), 1.59–1.53 (2H, m, ArCH$_2$C*H*$_2$), 1.32 (2H, ap. sextet, *J* = 7.4, C*H*$_2$CH$_3$), 0.90 (3H, t, *J* = 7.4,

CH_2CH_3), 0.32 (9H, s, Si(C*H*$_3$)$_3$); ^{13}C NMR (150 MHz, DMSO-d$_6$) 171.2, 144.7, 144.6, 136.6, 134.9, 133.9, 124.1, 44.7, 35.1, 33.3, 21.9, 13.8, -0.3; HRMS (ES$^+$) found [M-Me]$^+$ 246.1302; $C_{14}H_{20}$NOSi requires 246.1314.

5-(*o*-Tolyl)-7-(trimethylsilyl)isoindolin-1-one 13e

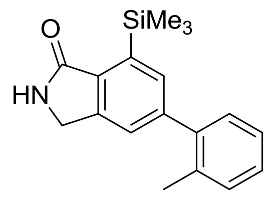

Prepared from *N*-(prop-2-yn-1-yl)-3-(trimethylsilyl)propiolamide **6c**, 2-ethynyltoluene **9l** and Cp*RuCl(cod) **3** (10 mg, 10 mol%) over 24 h according to the General Alkyne Cyclotrimerization Procedure (crude ratio **13e**:**14b** = 7:1) and purified by flash column chromatography (2:1 petrol:ethyl acetate) to give 5-(*o*-tolyl)-7-(trimethylsilyl)isoindolin-1-one **13e** as a white crystalline solid (48 mg, 0.16 mmol, 62%); m.p. 133–135 °C; R_f = 0.30 (2:1 ethyl acetate: petrol); ν_{max} (film/cm^{-1}) 3190 (N-H), 2947s (C-H), 1690s (C=O), 1450s; ^{1}H NMR (600 MHz, DMSO-d$_6$) 8.53 (1H, s, N*H*), 7.53 (1H, s, Ar*H*), 7.43 (1H, s, Ar*H*), 7.33–7.24 (4H, m, Ar*H*), 4.40 (2H, s, C*H*$_2$N), 2.24 (3H, s, ArC*H*$_3$), 0.32 (9H, s, Si(C*H*$_3$)$_3$); ^{13}C NMR (150 MHz, DMSO-d$_6$) 171.0, 144.4, 143.0, 141.0, 136.7, 135.9, 134.7, 134.4, 130.5, 129.6, 127.7, 126.1, 125.0, 45.0, 20.2, -0.3; HRMS (CI$^+$) found [M+H]$^+$ 296.1458; $C_{18}H_{22}$NOSi requires 296.1471.

***N*-(*tert*-Butyl)-*N*-((2-(*tert*-butyl)-1-oxo-7-(trimethylsilyl)isoindolin-5-yl)methyl)-3-(trimethylsilyl)propiolamide 14a**

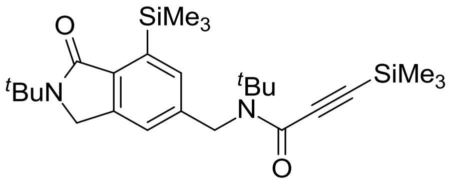

Cp*RuCl(cod) **3** (8 mg, 0.011 mmol, 10 mol%) was added dropwise to a stirring solution of *N*-(*tert*-butyl)-*N*-(prop-2-yn-1-yl)-3-(trimethylsilyl)propiolamide **6b** (50 mg, 0.11 mmol) in CPME (2.2 mL) at room temperature. The reaction was stirred for 16 h before the reaction mixture was filtered through a silica pad, eluting with ethyl acetate. The solvent was removed *in vacuo* to give the crude product, which was purified by flash column chromatography (10:1 ethyl acetate:petrol) to give *N*-((1-oxo-7-trimethylsiyl)isoindolin-5-yl)methyl)-3-(trimethylsilyl)propiolamide **14a** as a colourless oil (50 mg, 0.11 mmol, 100%); R_f = 0.43 (10:1 petrol: elthyl acetate) ;ν_{max} (film/cm^{-1}) 2963s (C-H), 1680s (C=O), 1631s (C=O); ^{1}H NMR (400 MHz, DMSO-d$_6$) 7.47 (1H, s, Ar*H*) 7.41 (1H, s, Ar*H*), 5.00 (2H, s, C*H*$_2$N), 4.54 (2H, s, C*H*$_2$N), 1.47 (9H, s, C(C*H*$_3$)$_3$), 1.31 (9H, s, C(C*H*$_3$)$_3$), 0.31 (9H, s, Si(C*H*$_3$)$_3$), 0.0 (9H, s, Si(C*H*$_3$)$_3$); ^{13}C NMR (125 MHz, DMSO-d$_6$) 168.5, 154.4, 141.8, 141.1, 137.5, 136.2, 131.6, 121.4, 98.6, 93.6, 57.7, 53.6, 50.4, 47.9, -0.4, -1.1; HRMS (CI$^+$) found [M]$^+$ 470.2791; $C_{26}H_{42}N_2O_2Si_2$ requires 470.2779.

N-((1-Oxo-7-trimethylsiyl)isoindolin-5-yl)methyl)-3-(trimethylsilyl)propiolamide 14b

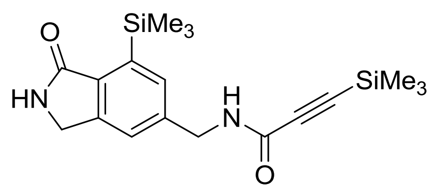

A solution of Cp*RuCl(cod) **3** (10 mg, 0.026 mmol, 10 mol%) in CPME (1.1 mL) was added dropwise to a stirring solution of *N*-(prop-2-ynyl)-3-(trimethylsilyl)propiolamide **6c** (47 mg, 0.26 mmol) in CPME (1.6 mL) at room temperature. The reaction was stirred for 16 h before the reaction mixture was filtered through a silica pad, eluting with ethyl acetate. The solvent was removed *in vacuo* to give the crude product, which was purified by flash column chromatography (2:1 ethyl acetate:petrol) to give *N*-((1-oxo-7-trimethylsiyl)isoindolin-5-yl)methyl)-3-(trimethylsilyl)propiolamide **14b** as a white crystalline solid (36 mg, 0.10 mmol, 77%); m.p. 104–106 °C; R_f = 0.36 (2:1 ethyl acetate:petrol); ν_{max} (film/cm^{-1}) 3321m (N-H), 3208m (N-H), 2957m (C-H), 1674s (C=O), 1655s (C=O), 1601m, 1531s; ^{1}H NMR (600 MHz, DMSO-d$_6$) 9.30 (1H, t, *J* = 5.7, N*H* straight chain amide), 8.46 (1H, s, N*H* lactam), 7.42 (1H, s, Ar*H*), 7.40 (1H, s, Ar*H*), 4.38–4.31 (4H, m, CH_2N, CH_2N), 0.32 (9H, s, Si(CH_3)$_3$), 0.30 (9H, s, Si(CH_3)$_3$); ^{13}C NMR (150 MHz, DMSO-d$_6$) 171.0, 151.8, 144.6, 140.7, 136.9, 136.1, 133.1, 123.3, 98.8, 90.1, 44.8, 42.5, -0.4, -0.7; HRMS (CI$^+$) found [M+H]$^+$ 359.1617; $C_{18}H_{27}N_2O_2Si_2$ requires 359.1611.

2-Benzyl-5-butyl-4-methyl-7-(trimethylsilyl)isoindolin-1-one 15a and 2-Benzyl-6-butyl-4-methyl-7-(trimethylsilyl)isoindolin-1-one 16a

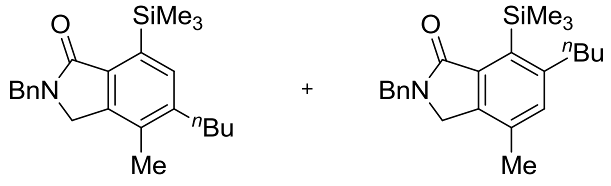

15a **16a**

A solution of *N*-benzyl-*N*-(but-2-yn-1-yl)-3-(trimethylsilyl)propiolamide **6d** (74 mg, 0.26 mmol) in CPME (1.6 mL) was added dropwise over 1 minute to a stirring solution of 1-hexyne (61 µL, 0.52 mmol) and Cp*RuCl(cod) **3** (10 mg, 10 mol%) in CPME (1.1 mL) at room temperature. The reaction was stirred for 24 h before the reaction mixture was filtered through a silica pad, eluting with ethyl acetate. The solvent was removed in vacuo to give the crude product (crude ratio **15a**:**16a** = 9:1). This was purified by flash column chromatography (15:1 petrol:ethyl acetate) to give a mixture of 2-benzyl-5-butyl-4-methyl-7-(trimethylsilyl)isoindolin-1-one **15a** and 2-benzyl-6-butyl-4-methyl-7-(trimethylsilyl)isoindolin-1-one **16a** as a colourless oil (10 mg, 0.027 mmol, 11%; **15a**:**16a** = 3:1). Further elution of the column gave more 2-benzyl-5-butyl-4-methyl-7-(trimethylsilyl)isoindolin-1-one **15a** as a colourless oil (55 mg, 0.15 mmol, 58%).

2-Benzyl-5-butyl-4-methyl-7-(trimethylsilyl)isoindolin-1-one 15a: R_f = 0.75 (9:1 petrol:ethyl acetate); ν_{max} (film/cm^{-1}) 2956s (C-H), 1688s (C=O), 1454s, 1409s; ^{1}H NMR (600 MHz, DMSO-d$_6$) 7.37–7.34 (2H, m, Ar*H*), 7.31 (1H, s, Ar*H*), 7.29–7.26 (3H, m, Ar*H*), 4.71 (2H, s, NCH_2), 4.27 (2H, s, NCH_2), 2.65 (2H, t, *J* = 7.8, ArCH_2CH$_2$), 2.17 (3H, s, ArCH_3), 1.50–1.45 (2H, m, ArCH$_2$CH_2), 1.35 (2H, sextet, *J* =7.3, CH_2CH$_3$), 0.91 (3H, t, *J* = 7.3, CH$_2$CH_3), 0.34 (9H, s, Si(CH_3)$_3$); ^{13}C NMR (150 MHz, DMSO-d$_6$) 169.0, 143.0, 141.6, 137.8, 135.0, 134.2, 133.6, 131.6, 128.7, 127.7, 123.3, 48.6, 45.5, 32.5, 32.4, 22.1, 13.8, 13.7, -0.3; HRMS (CI$^+$) found [M+H]$^+$ 366.2246; $C_{23}H_{32}NOSi$ requires 366.2248. A NOESY experiment showed an NOE between; 2.65 (ArCH_2CH$_2$) and 2.17 (ArCH_3); 7.31 (Ar*H*) and 2.65 (ArCH_2CH$_2$); 7.31 (Ar*H*) and 0.34 (Si(CH_3)$_3$).

2-Benzyl-6-butyl-4-methyl-7-(trimethylsilyl)isoindolin-1-one 16a: R_f = 0.77 (9:1 petrol:ethyl acetate); ^{1}H NMR (600 MHz, DMSO-d_6) 7.37–7.34 (2H, m, Ar*H*), 7.30–7.26 (3H, m, Ar*H*), 7.13 (1H, s, Ar*H*), 4.70 (2H, s, NCH_2), 4.22 (2H, s, NCH_2), 2.73 (2H, t, *J* = 7.7, $ArCH_2CH_2$), 2.20 (3H, s, $ArCH_3$), 1.44–1.40 (2H, m, $ArCH_2CH_2$), 1.32 (2H, sextet, *J* =7.4, CH_2CH_3), 0.89 (3H, t, *J* = 7.4, CH_2CH_3), 0.40 (9H, s, $Si(CH_3)_3$); ^{13}C NMR (150 MHz, DMSO-d_6) 168.9, 149.1, 139.1, 137.4, 133.7, 133.3, 132.4, 128.5, 127.7, 47.7, 45.6, 36.4, 35.7, 22.1, 16.9, 14.0, 3.4. Two aromatic ^{13}C resonances were obscured by compound **15a**.

2-Benzyl-5-butyl-4-ethyl-7-(trimethylsilyl)isoindolin-1-one 15b and 2-Benzyl-6-butyl-4-ethyl-7-(trimethylsilyl)isoindolin-1-one 16b

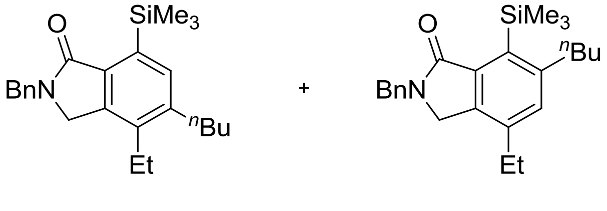

15b **16b**

A solution of *N*-benzyl-*N*-(pent-2-yn-1-yl)-3-(trimethylsilyl)propiolamide **6e** (79 mg, 0.26 mmol) in CPME (1.6 mL) was added dropwise over 1 minute to a stirring solution of 1-hexyne (61µL, 0.52 mmol) and Cp*RuCl(cod) **3** (10 mg, 10 mol%) in CPME (1.1 mL) at room temperature. The reaction was stirred for 24 h before the reaction mixture was filtered through a silica pad, eluting with ethyl acetate. The solvent was removed in vacuo to give the crude product (crude ratio **15b**:**16b** = 2:1). This was purified by flash column chromatography (18:1 petrol:ethyl acetate) to give a mixture of 2-benzyl-5-butyl-4-ethyl-7-(trimethylsilyl)isoindolin-1-one **15b** and 2-benzyl-6-butyl-4-ethyl-7-(trimethylsilyl)isoindolin-1-one **16b** as a colourless oil (40 mg, 0.11 mmol, 40%, **15b**:**16b** = 3:2). Further elution of the column gave more 2-benzyl-5-butyl-4-ethyl-7-(trimethylsilyl)isoindolin-1-one **15b** as a colourless oil (17 mg, 0.045 mmol, 17%).

2-Benzyl-5-butyl-4-ethyl-7-(trimethylsilyl)isoindolin-1-one 15b: R_f = 0.25 (18:1 petrol:ethyl acetate); ν_{max} (film/cm^{-1}) 2957s (C-H), 1690s (C=O), 1409s; ^{1}H NMR (600 MHz, DMSO-d_6) 7.36–7.33 (3H, m, Ar*H*), 7.29–7.26 (3H, m, Ar*H*), 4.71 (2H, s, CH_2N), 4.33 (2H, s, CH_2N), 2.65 (2H, t, *J* = 7.9, $ArCH_2CH_2$), 2.58 (2H, q, *J* = 7.6, $ArCH_2CH_3$), 1.52–1.47 (2H, m, $ArCH_2CH_2$), 1.37 (2H, sextet, *J* = 7.4, $CH_2CH_2CH_3$), 1.06 (3H, t, *J* = 7.6, $ArCH_2CH_3$), 0.91 (3H, t, *J* = 7.4, $CH_2CH_2CH_3$), 0.34 (9H, s, $Si(CH_3)_3$); ^{13}C NMR (150 MHz, DMSO-d_6) 169.0 (*C*(O)), 142.4, 140.9, 137.8, 137.4, 135.5, 134.4, 133.9, 128.7, 127.7, 127.3, 48.2 (CH_2N), 45.5 (CH_2N), 33.6 ($ArCH_2CH_2$), 31.5 ($ArCH_2CH_2$), 22.2 ($ArCH_2CH_2CH_2$), 21.4 ($ArCH_2CH_3$), 14.3 ($ArCH_2CH_3$), 13.9 ($CH_2CH_2CH_3$), -0.3 ($Si(CH_3)_3$); HRMS (CI$^+$) found $[M+H]^+$ 380.2410; $C_{24}H_{34}NOSi$ requires 380.2404. A NOESY experiment showed an NOE between; 7.19 (Ar*H*) and 2.65 ($ArCH_2CH_2$); 2.65 ($ArCH_2CH_2$) and 2.57 ($ArCH_2CH_3$); 7.19 (Ar*H*) and 0.36 ($Si(CH_3)_3$).

2-Benzyl-6-butyl-4-ethyl-7-(trimethylsilyl)isoindolin-1-one 16b: R_f = 0.31 (18:1 petrol:ethyl acetate); ^{1}H NMR (600 MHz, DMSO-d_6) 7.36–7.32 (2H, m, Ar*H*), 7.29–7.25 (3H, m, Ar*H*), 7.14 (1H, s, Ar*H*), 4.69 (2H, s, CH_2N), 4.25 (2H, s, CH_2N), 2.74 (2H, t, *J* = 7.9, $ArCH_2CH_2$), 2.52 (2H, q, *J* = 7.6, $ArCH_2CH_3$), 1.45–1.40 (2H, m, $ArCH_2CH_2$), 1.31 (2H, sextet, *J* = 7.2, $CH_2CH_2CH_3$), 1.13 (3H, t, *J* = 7.6, $ArCH_2CH_3$), 0.87 (3H, t, *J* = 7.2, $CH_2CH_2CH_3$), 0.40 (9H, s, $Si(CH_3)_3$); ^{13}C NMR (150 MHz, DMSO-d_6) 169.0 (*C*(O)), 149.3 , 139.0 , 138.3 , 137.5 , 135.5 , 132.6 , 131.9 , 127.7 , 47.4 (CH_2N), 45.6 (CH_2N), 36.5 ($ArCH_2CH_2$), 35.9 ($ArCH_2CH_2$), 23.9 ($ArCH_2CH_2$), 22.1 ($ArCH_2CH_2CH_2$), 14.0 (CH_2CH_3), 13.8 (CH_2CH_3), 0.3 ($Si(CH_3)_3$). Two aromatic ^{13}C resonances were obscured by compound **15b**.

2-Benzyl-4-ethyl-5-(*o*-tolyl)-7-(trimethylsilyl)isoindolin-1-one 15c and 2-Benzyl-4-ethyl-6-(*o*-tolyl)-7-(trimethylsilyl)isoindolin-1-one 16c

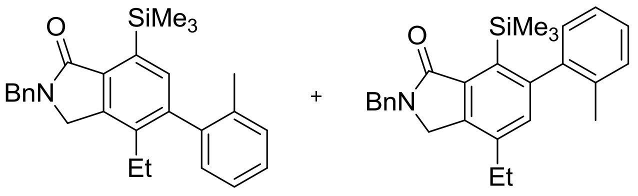

15c **16c**

A solution of *N*-benzyl-*N*-(pent-2-yn-1-yl)-3-(trimethylsilyl)propiolamide **6e** (79 mg, 0.26 mmol) in CPME (1.6 mL) was added dropwise over 1 minute to a stirring solution of 2-ethynyltoluene (66μL, 0.52 mmol) and Cp*RuCl(cod) **3** (10 mg, 10 mol%) in CPME (1.1 mL) at room temperature. The reaction was stirred for 24 h before the reaction mixture was filtered through a silica pad, eluting with ethyl acetate. The solvent was removed in vacuo to give the crude product (crude ratio **15c**:**16c** = 5:1). This was purified by flash column chromatography (18:1 petrol:ethyl acetate) to give a mixture of 2-benzyl-4-ethyl-5-(*o*-tolyl)-7-(trimethylsilyl)isoindolin-1-one **15c** and 2-benzyl-4-ethyl-6-(*o*-tolyl)-7-(trimethylsilyl)isoindolin-1-one **16c** as a colourless oil (80 mg, 0.19 mmol, 73%; **15c**:**16c** = 5:1); R_f = 0.33 (18:1 petrol:ethyl acetate); ν_{max} (film/cm^{-1}) 2964s (C-H), 1690s (C=O), 1410s; ^{1}H NMR (600 MHz, DMSO-d$_6$) 7.39–7.23 (m; 8H, Ar*H* **15c**; 8H, Ar*H* **16c**), 7.19 (1H, s, C*H*CSi **15c**), 7.11 (1H, d, *J* = 7.6, Ar*H* **15c**), 7.04 (1H, s, C*H*CSi **16c**), 7.00 (1H, d, *J* = 7.6, Ar*H* **16c**), 5.78–4.73 (m; 2H, C*H*$_2$N **15c**; 2H, C*H*$_2$N, **16c**), 4.48–4.40 (m; 2H, C*H*$_2$N **15c**; 2H, C*H*$_2$N, **16c**), 2.60 (2H, q, *J* = 7.8, C*H*$_2$CH$_3$ **16c**), 2.46 (1H, sextet, *J* = 7.7, C*H*H'CH$_3$ **15c**), 2.23 (1H, sextet, *J* = 7.7, CH*H*'CH$_3$ **15c**), 2.05 (3H, s, ArC*H*$_3$ **16c**), 1.98 (3H, s, ArC*H*$_3$ **15c**), 1.15 (3H, t, *J* = 7.6, CH$_2$C*H*$_3$ **16c**), 0.82 (3H, t, *J* = 7.7, CH$_2$C*H*$_3$ **15c**), 0.34 (9H, s, Si(C*H*$_3$)$_3$ **15c**), -0.06 (9H, s, Si(C*H*$_3$)$_3$ **16c**); ^{13}C NMR (150 MHz, DMSO-d$_6$) 168.8, 168.7, 148.4, 144.0, 142.6, 141.0, 140.0, 139.4, 138.8, 137.8, 137.7, 137.4, 135.9, 135.4, 135.2, 135.1, 134.1, 133.1, 131.5, 130.0, 129.7, 129.2, 128.7, 127.8, 127.7, 127.7, 127.5, 127.4, 125.6, 125.3, 48.4, 47.7, 45.7, 45.6, 23.8, 22.2, 20.2, 19.9, 13.6, 1.6, -0.3; HRMS (CI$^+$) found [M+H]$^+$ 414.2245; $C_{27}H_{32}NOSi$ requires 414.2248. A NOESY experiment showed an NOE between; 7.19 (Ar*H* **15c**) and 1.98 (ArC*H*$_3$ **15c**); 7.19 (Ar*H* **15c**) and 0.36 (Si(C*H*$_3$)$_3$ **15c**).

2-Benzyl-5-butyl-7-methylisoindolin-1-one 15d

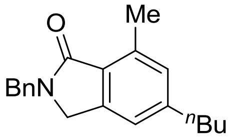

Prepared from *N*-benzyl-*N*-(prop-2-yn-1-yl)but-2-ynamide **6f**, 1-hexyne and Cp*RuCl(cod) (3 mg, 3 mol%) over 16 h according to the General Alkyne Cyclotrimerization Procedure and purified by flash column chromatography (7:1 petrol:ethyl acetate) to give 2-benzyl-5-butyl-7-methylisoindolin-1-one **15d** as a colourless oil (65 mg, 0.22 mmol, 85%); R_f = 0.31 (7:1 petrol:ethyl acetate:); ν_{max} (film/cm^{-1}) 2927s (C-H), 1681s (C=O), 1615s, 1453s, 1407s; ^{1}H NMR (600 MHz, DMSO-d$_6$) 7.36–7.32 (2H, m, Ar*H*), 7.28–7.24 (3H, m, Ar*H*), 7.13 (1H, s, Ar*H*), 7.04 (1H, s, Ar*H*), 4.67 (2H, s, C*H*$_2$N), 4.24 (2H, s, C*H*$_2$N), 2.61–2.57 (5H, m, ArC*H*$_3$; ArC*H*$_2$CH$_2$), 1.53 (2H, quintet, *J* = 7.5, ArCH$_2$C*H*$_2$), 1.28 (2H, sextet, *J* = 7.5, C*H*$_2$CH$_3$), 0.87 (3H, t, *J* = 7.5, CH$_2$C*H*$_3$); ^{13}C NMR (150 MHz, DMSO-d$_6$) 168.2, 145.9, 142.6, 137.7, 136.0, 129.9, 128.7, 127.7, 127.3, 126.8, 120.6, 48.5, 45.1, 34.9, 33.1, 21.8, 16.7, 13.8; data in accordance with the literature.[7]

2-Benzyl-7-methyl-5-(o-tolyl)isoindolin-1-one 15e

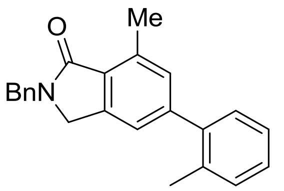

Prepared from *N*-benzyl-*N*-(prop-2-yn-1-yl)but-2-ynamide **6f**, 2-ethynyltoluene and Cp*RuCl(cod) (3 mg, 3 mol%) over 16 h according to the General Alkyne Cyclotrimerization Procedure to give 2-benzyl-7-methyl-5-(*o*-tolyl)isoindolin-1-one **15e** as a yellow oil (80 mg, 0.24 mmol, 94%); R_f = 0.31 (5:1 petrol:ethyl acetate); ν_{max} (film/cm^{-1}) 2923s (C-H), 1684s (C=O), 1614s 1495s, 1453s; ^{1}H NMR (600 MHz, DMSO-d$_6$) 7.37–7.34 (2H, m, Ar*H*), 7.30–7.22 (7H, m, Ar*H*), 71.9–7.17 (2H, m, Ar*H*), 4.71 (2H, s, C*H*$_2$N), 4.31 (2H, s, C*H*$_2$N), 2.63 (3H, s, ArC*H*$_3$), 2.21 (3H, s, ArC*H*$_3$); ^{13}C NMR (150 MHz, DMSO-d$_6$) 168.0, 144.1, 142.4, 140.7, 137.7, 136.1, 134.7, 130.5, 130.4, 129.5, 128.7, 127.8, 127.7, 127.4, 126.0, 121.5, 48.7, 45.3, 20.2, 16.8; HRMS (CI$^+$) found [M+H]$^+$ 328.1688; $C_{23}H_{21}NO$ requires 328.1696.

2-Benzyl-7-bromo-5-butylisoindolin-1-one 17

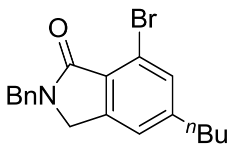

According to the modified procedure of Snieckus *et al.*[14]: Bromine (118 mg, 0.738 mmol) in CH_2Cl_2 (0.36 mL) was added dropwise to a stirring solution of 2-benzyl-5-butyl-7-(trimethylsilyl)isoindolin-1-one **10a** (26 mg, 0.074 mmol) in CH_2Cl_2 (0.36 mL) at room temperature. The reaction was stirred for 16 h and aq. sat. $Na_2S_2O_3$ was added to afford a colourless solution. The reaction was extracted with CH_2Cl_2 (2 × 20 mL), dried ($MgSO_4$), and the solvent was removed *in vacuo*. The crude product was purified by flash column chromatography (10:1 petrol:ethyl acetate) to give 2-benzyl-7-bromo-5-butylisoindolin-1-one **17** as a colourless oil (21 mg, 0.059 mmol, 79%); R_f = 0.50 (5:1 petrol:ethyl acetate); ν_{max} (film/cm^{-1}) 2928s (C-H), 1692s (C=O), 1615s, 1453s, 1408s; ^{1}H NMR (600 MHz, DMSO-d$_6$) 7.49 (1H, s, Ar*H*), 7.37–7.33 (3H, m, Ar*H*), 7.29–7.26 (3H, m, Ar*H*), 4.69 (2H, s, C*H*$_2$N), 4.28 (2H, s, C*H*$_2$N), 2.64 (2H, t, *J* = 7.7, ArC*H*$_2$CH$_2$), 1.54 (2H, m, ArCH$_2$C*H*$_2$), 1.28 (2H, ap. sextet, *J* = 7.4, C*H*$_2$CH$_3$), 0.87 (3H, t, *J* = 7.4, C*H*$_3$); ^{13}C NMR (150 MHz, DMSO-d$_6$) 165.3, 148.3, 145.0, 137.4, 132.4, 128.7, 127.7, 127.4, 126.9, 122.9, 117.2, 48.1, 45.4, 34.4, 32.9, 21.7, 13.8; HRMS (ESI$^+$) found [M+H]$^+$ 358.0807; $C_{19}H_{21}BrNO$ requires 358.0807.

2-Benzyl-5-butyl-7-iodoisoindolin-1-one 18

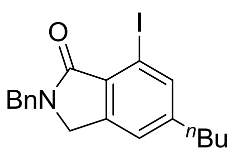

According to the modified procedure of Clayden *et al.*[15]: Iodine monochloride (93 mg, 0.57 mmol) in CH_2Cl_2 (0.57 mL) was added dropwise to a stirring solution of 2-benzyl-5-butyl-7-(trimethylsilyl)isoindolin-1-one **10a** (26 mg, 0.074 mmol) in CH_2Cl_2 (17 mL) at room temperature. The reaction was stirred for 3.5 h and aq. sat. $Na_2S_2O_3$ was added to afford a colourless solution. The reaction was extracted with CH_2Cl_2 (2 × 20 mL), dried ($MgSO_4$), and the solvent was removed *in vacuo*. The crude product was purified by flash column chromatography (5:1 petrol:ethyl acetate) to give 2-benzyl-5-butyl-7-iodoisoindolin-1-one **18** as a colourless oil

(27 mg, 0.067 mmol, 90%); R_f = 0.20 (6:1 petrol:ethyl acetate); ν_{max} (film/cm^{-1}) 2929s (C-H), 1690s (C=O), 1609s, 1453s, 1408s; ^{1}H NMR (600 MHz, DMSO-d$_6$) 7.74 (1H, s, Ar*H*), 7.38 (1H, s, Ar*H*), 7.36–7.33 (2H, m, Ar*H*), 7.29–7.25 (3H, m, Ar*H*), 4.69 (2H, s, C*H*$_2$N), 4.23 (2H, s, C*H*$_2$N), 2.60 (2H, t, *J* = 7.7, ArC*H*$_2$CH$_2$), 1.53 (2H, m, ArCH$_2$C*H*$_2$), 1.27 (2H, ap. sextet, *J* = 7.4, C*H*$_2$CH$_3$), 0.87 (3H, t, *J* = 7.4, C*H*$_3$); ^{13}C NMR (150 MHz, DMSO-d$_6$) 165.9, 148.1, 144.6, 139.0, 137.4, 129.4, 128.7, 127.7, 127.4, 123.4, 89.9, 47.7, 45.6, 34.2, 32.9, 21.7, 13.8; HRMS (ES$^+$) found [M+H]$^+$ 406.0663; $C_{19}H_{21}INO$ requires 406.0668.

2-(*tert*-Butyl)-5-butyl-7-iodoisoindolin-1-one

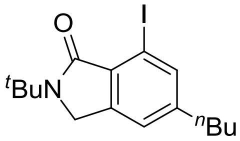

According to the modified procedure of Clayden *et al.*[15]: Iodine monochloride (400 mg, 2.49 mmol) in CH_2Cl_2 (5.0 mL) was added dropwise to a stirring solution of 2-(*tert*-butyl)-5-butyl-7-(trimethylsilyl)isoindolin-1-one **13a** (158 mg, 0.498 mmol) in CH_2Cl_2 (7.0 mL) at room temperature. The reaction was stirred for 2 h and the reaction mixture was diluted with sat. aq. $NaHCO_3$ (10 mL), before sat. aq. $Na_2S_2O_3$ (10 mL) was added dropwise to afford a colourless solution. The reaction was extracted with CH_2Cl_2 (2 × 20 mL), dried ($MgSO_4$), and the solvent was removed *in vacuo* to give 2-(*tert*-butyl)-5-butyl-7-iodoisoindolin-1-one as a yellow oil (183 mg, 0.492 mmol, 99%); R_f = 0.31 (6:1 petrol:ethyl acetate); ν_{max} (film/cm^{-1}) 2957s (C-H), 2928s (C-H), 1678s (C=O), 1607s, 1455s; ^{1}H NMR (600 MHz, DMSO-d$_6$) 7.67 (1H, s, Ar*H*), 7.33 (1H, s, Ar*H*), 4.39 (2H, s, C*H*$_2$N), 2.57 (2H, t, *J* = 7.6, ArC*H*$_2$CH$_2$), 1.52–1.47 (2H, m, ArCH$_2$C*H*$_2$), 1.44 (9H, s, C(C*H*$_3$)$_3$), 1.25 (2H, ap. sextet, *J* = 7.4, C*H*$_2$CH$_3$), 0.85 (3H, t, *J* = 7.4, C*H*$_3$); ^{13}C NMR (150 MHz, DMSO-d$_6$) 165.8, 147.4, 144.1, 138.8, 130.5, 122.9, 89.0, 53.8, 46.3, 34.3, 33.0, 27.4, 21.7, 13.8; HRMS (CI$^+$) found [M+H]$^+$ 372.0837; $C_{16}H_{22}INO$ requires 372.0824.

5-Butyl-7-iodoisoindolin-1-one 19

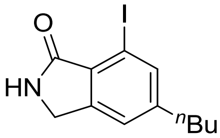

According to the modified procedure of Miranda *et al.*[16]: Triflic acid (0.75 mL) was added to 2-(*tert*-butyl)-5-butyl-7-iodoisoindolin-1-one (156 mg, 0.420 mmol). The resulting solution was stirred at room temperature for 48 h before being partitioned between ethyl acetate (20 mL) and water (20 mL). The aqueous extract was washed with ethyl acetate (3 × 20 mL), dried ($MgSO_4$) and concentrated *in vacuo* to give the crude product. This was purified by flash column chromatography (1:1 petrol:ethyl acetate) to give 5-butyl-7-iodoisoindolin-1-one **19** as a white crystalline solid (112 mg, 0.355, 85%); m.p. 137–139 °C; R_f = 0.19 (1:1 petrol:ethyl acetate); ν_{max} (film/cm^{-1}) 3171s (N-H), 3073s (C-H), 2928s (C-H), 1702s (C=O), 1607s, 1457s; ^{1}H NMR (600 MHz, DMSO-d$_6$); 8.64 (1H, s, N*H*), 7.73 (1H, s, Ar*H*), 7.41 (1H, s, Ar*H*), 4.23 (2H, s, C*H*$_2$N), 2.63 (2H, t, *J* = 7.7, ArC*H*$_2$CH$_2$), 1.59–1.53 (2H, m, ArCH$_2$C*H*$_2$), 1.29 (2H, ap. sextet, *J* = 7.4, C*H*$_2$CH$_3$), 0.88 (3H, t, *J* = 7.4, C*H*$_3$); ^{13}C NMR (150 MHz, DMSO-d$_6$); 168.5, 147.9, 146.8, 138.8, 129.7, 123.6, 89.7, 43.0, 34.2, 33.0, 21.7, 13.8; HRMS (ES$^+$) found [M]$^+$ 315.0108; $C_{12}H_{14}INO$ requires 315.0120.

5-Butylisoindolin-1-one 20

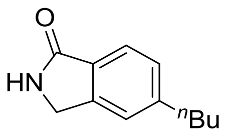

According to the modified procedure of Miranda *et al.*[16]: Triflic acid (0.5 mmol) was added to 2-(*tert*-butyl)-5-butyl-7-(trimethylsilyl)isoindolin-1-one **13a** (50 mg, 0.16 mmol). The resulting solution was stirred at room temperature for 30 minutes before being partitioned between ethyl acetate (20 mL) and water (20 mL). The aqueous extract was washed with ethyl acetate (2 × 20 mL) and the combined organic extracts were washed with aq. sat. Na_2CO_3 (50 mL) and brine (50 mL), dried ($MgSO_4$) and concentrated *in vacuo* to give the crude product. This was purified by flash column chromatography (3:1 ethyl acetate:petrol) to give 5-butylisoindolin-1-one **20** as a white crystalline solid (26 mg, 0.014 mmol, 87%); m.p. 145–147 °C; R_f = 0.57 (2:1 petrol:ethyl acetate); ν_{max} (film/cm^{-1}); 3203s (N-H), 3083s (C-H), 2932s (C-H), 1673s (C=O), 1451s; ^{1}H NMR (600 MHz, DMSO-d$_6$) 8.50 (1H, s, N*H*), 7.56 (1H, d, *J* = 7.7, Ar*H*), 7.37 (1H, s, Ar*H*), 7.28 (1H, d, *J* = 7.7, Ar*H*), 4.32 (2H, s, C*H*$_2$N), 2.67 (2H, t, *J* = 7.7, ArC*H*$_2$CH$_2$), 1.59–1.54 (2H, m, ArCH$_2$C*H*$_2$), 1.30 (2H, ap. sextet, *J* = 7.4, C*H*$_2$CH$_3$), 0.89 (3H, t, *J* = 7.4, C*H*$_3$); ^{13}C NMR (150 MHz, DMSO-d$_6$) 170.0, 146.2, 144.5, 130.4, 128.0, 123.4, 122.6, 44.8, 39.1, 35.0, 21.8, 13.8; HRMS (EI$^+$) found [M]$^+$ 189.1153; $C_{12}H_{15}NO$ requires 189.1148.

tert-Butyl 5-butyl-7-iodo-1-oxoisoindoline-2-carboxylate 21

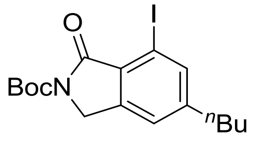

According to the modified procedure of Miranda *et al.*[17]: Di-*tert*-butyl dicarbonate (55 mg, 0.25 mmol) and *N*,*N*-dimethylpyridin-4-amine (1.5 mg, 0.012 mmol) was added to a stirring solution of 5-butyl-7-iodoisoindolin-1-one **19** (40 mg, 0.13 mmol) in THF (2.0 mL) at room temperature. The resulting solution was stirred at room temperature for 16 h, after which a further quantity of di-*tert*-butyl dicarbonate (55 mg, 0.25 mmol) and *N*,*N*-dimethylpyridin-4-amine (1.5 mg, 0.012 mmol) was added. After a further 3 h the reaction mixture was concentrated *in vacuo* to give the crude product, which was purified by flash column chromatography (10:1 petrol:ethyl acetate) to give *tert*-butyl 5-butyl-7-iodo-1-oxoisoindoline-2-carboxylate **21** as a colourless oil (48 mg, 0.12 mmol, 91%); R_f = 0.24 (10:1 petrol:ethyl acetate); ν_{max} (film/cm^{-1}), 2930s (C-H), 1777s (C=O), 1747s (C=O), 1712s (C=O), 1605s, 1456s; ^{1}H NMR (600 MHz, DMSO-d$_6$); 7.82 (1H, s, Ar*H*), 7.47 (1H, s, Ar*H*), 4.63 (2H, s, C*H*$_2$N), 2.64 (2H, t, *J* = 7.7, ArC*H*$_2$CH$_2$), 1.59–1.53 (2H, m, ArCH$_2$C*H*$_2$), 1.52 (9H, s, C(C*H*$_3$)$_3$) 1.30 (2H, ap. sextet, *J* = 7.4, C*H*$_2$CH$_3$), 0.89 (3H, t, *J* = 7.4, CH$_2$C*H*$_3$); ^{13}C NMR (150 MHz, DMSO-d$_6$); 164.1, 150.2, 149.8, 144.5, 139.7, 128.0, 123.5, 91.3, 82.0, 47.3, 34.4, 32.7, 27.7, 21.8, 13.7; HRMS (CI$^+$) found [M+H]$^+$ 416.0717; $C_{17}H_{23}INO_3$ requires 416.0723.

tert-Butyl 5-butyl-2-(hydroxymethyl)-3-iodobenzylcarbamate 22 and tert-Butyl 5-butyl-1-hydroxy-7-iodoisoindoline-2-carboxylate 23

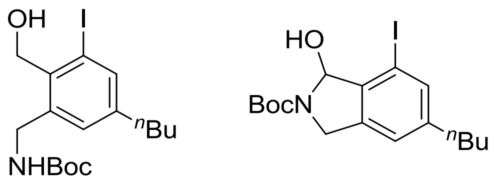

22 **23**

According to the modified procedure of Ohno *et al.*[18]: Lithium borohydride (4.2 mg, 0.19 mmol) was added to a solution of *tert*-butyl 5-butyl-7-iodo-1-oxoisoindoline-2-carboxylate **21** (32 mg, 0.077 mmol) and methanol (3.7 mg, 0.12 mmol) in diethyl ether (1.0 mL) at 0 °C. The resulting solution was stirred at room temperature for 90 minutes, after which a further portion of lithium borohydride (0.42 mg, 0.19 mmol) was added. The reaction was stirred at room temperature for a further 16 h before the reaction was diluted with aq. sat. ammonium chloride (10 mL) and diethyl ether (10 mL). The aqueous extract was washed with diethyl ether (3 × 10 mL) and the combined organic extracts were washed with water (10 mL) and brine (10 mL), dried ($MgSO_4$) and concentrated *in vacuo* to give the crude product. This was purified by flash column chromatography (8:1 petrol:ethyl acetate) to give *tert*-butyl 5-butyl-1-hydroxy-7-iodoisoindoline-2-carboxylate **23** as a colourless oil (8.0 mg, 0.019 mmol, 25%); R_f = 0.20 (10:1 petrol:ethyl acetate); ν_{max} (film/cm^{-1}) 2974s (C-H), 1755s, 1707s (C=O), 1456s; ^{1}H NMR (600 MHz, DMSO-d_6) a 50:50 mixture of rotamers R_1 and R_2; 7.55 (1H, s, Ar*H* R_1; 1H, s, Ar*H* R_2) 7.20 (1H, s, Ar*H* R_1 or R_2), 7.18 (1H, s, Ar*H* R_1 or R_2), 6.39 (1H, d, *J* = 8.0, O*H* R_1), 6.22 (1H, d, *J* = 8.3, O*H* R_2), 6.03 (1H, d, *J* = 8.0, C*H*$_2$O R_1), 5.94 (1H, d, *J* = 8.3, C*H*$_2$O R_2), 4.59–4.45 (2H, m, C*H*$_2$N R_1; 2H, m, C*H*$_2$N R_2), 2.56 (2H, t, *J* = 7.7, ArC*H*$_2$CH$_2$ R_1; 2H, t, *J* = 7.7, ArC*H*$_2$CH$_2$ R_2), 1.54–1.50 (2H, m, ArCH$_2$C*H*$_2$ R_1; 2H, m, ArCH$_2$C*H*$_2$ R_2), 1.47 (9H, s, C(C*H*$_3$)$_3$ R_1 or R_2), 1.46 (9H, s, C(C*H*$_3$)$_3$ R_1 or R_2), 1.28 (2H, ap. sextet, *J* = 7.3, C*H*$_2$CH$_3$ R_1; 2H, ap. sextet, *J* = 7.3, C*H*$_2$CH$_3$ R_2), 0.88 (3H, d, *J* = 7.3, CH$_2$C*H*$_3$ R_1; 3H, d, *J* = 7.3, CH$_2$C*H*$_3$ R_2); ^{13}C NMR (150 MHz, DMSO-d_6) a mixture of rotamers; 153.1, 153.0, 145.4, 140.7, 140.2, 138.7, 138.4, 136.9, 136.9, 122.6, 122.5, 91.7, 85.6, 85.6, 79.4, 79.2, 50.7, 50.4, 34.1, 33.1, 28.1, 21.7, 13.8; HRMS (CI$^+$) found [M+H]$^+$ 418.0870; $C_{17}H_{25}INO_3$ requires 418.0874. Further elution of the column gave *tert*-butyl 5-butyl-2-(hydroxymethyl)-3-iodobenzylcarbamate **22** as a white crystalline solid (17 mg, 0.041 mmol, 53%); m.p. 78–76 °C; R_f = 0.11 (5:1 petrol:ethyl acetate); ν_{max} (film/cm^{-1}), 3353s (O-H, N-H), 2958s (C-H), 2925s (C-H), 2856s (C-H), 1687s (C=O) 1601s, 1507s, 1458s; ^{1}H NMR (600 MHz, DMSO-d_6) a mixture of rotamers R_1 and R_2; 7.47 (1H, s, Ar*H*), 7.26 (1H, t, *J* = 5.9, N*H*), 7.09 (1H, s, Ar*H*), 5.01 (1H, t, *J* = 5.0, CH$_2$O*H*), 4.62 (2H, d, *J* = 5.0, C*H*$_2$OH), 4.28 (2H, d, *J* = 5.9, C*H*$_2$N), 2.51–2.48 (2H, m, ArC*H*$_2$CH$_2$ and solvent peak), 1.49 (2H, ap. quintet, *J* = 7.4, ArCH$_2$C*H*$_2$), 1.39 (9H, s, C(C*H*$_3$)$_3$), 1.27 (2H, ap. sextet, *J* = 7.4, C*H*$_2$CH$_3$), 0.87 (3H, t, *J* = 7.4, CH$_2$C*H*$_3$); ^{13}C NMR (150 MHz, DMSO-d_6); 155.7, 143.8, 140.4, 137.7, 137.4, 127.8, 102.5, 78.0, 64.8, 42.7, 33.9, 32.9, 28.2, 21.6, 13.8; HRMS (CI+) found [M+H]$^+$ 420.1027; $C_{17}H_{27}INO_3$ requires 420.1030.

References

[1] T. Hamada , D. Suzuki, H. Urabe, F. Sato, *J. Am. Chem. Soc.* **1999**, *121*, 7342-7344.
http://dx.doi.org/10.1021/ja9905694

[2] I. Fleming, S. Gill, A. K. Sarkar, T. Schmidlin, *J. Chem. Soc. Perkin Trans. 1* **1992**, 3351-3361.
http://dx.doi.org/10.1039/P19920003351

[3] M. Shi, C.-J. Wang, *J. Chem. Res.* **2004**, *2004*, 107-110.
http://dx.doi.org/10.3184/030823404323000378

[4] W. Hess, J. W. Burton, *Chem. Eur. J.* **2010**, *16*, 12303-12306.
http://dx.doi.org/10.1002/chem.201001951

[5] A. Nudelman, Y. Binnes, N. Shmueli-Broidea, Y. Odessaa, J. P. Hieble, A. C. Sulpizio, *Arch. Pharm. Pharm. Med. Chem.* **1996**, *329*, 125-132.
http://dx.doi.org/10.1002/ardp.19963290304

[6] Y. Hirata, T. Yukawa, N. Kashihara, Y. Nakao, T. Hiyama, *J. Am. Chem. Soc.*, **2009**, *131*, 10964–10973.
http://dx.doi.org/10.1021/ja901374v

[7] Y. Yamamoto, K. Kinpara, T. Saigoku, H. Nishiyamaa, K. Itoh, *Org. Biomol. Chem.*, **2004**, *2*, 1287–1294.
http://dx.doi.org/10.1039/B402649G

[8] G. A. Molander, F. Cadoret, *Tet. Lett.* **2011**, *52*, 2199-2202.
http://dx.doi.org/10.1016/j.tetlet.2010.11.162

[9] Q. Li, A. V. Rukavishnikov, P. A. Petukhov, T. O. Zaikova, C. Jin, J. F. W. Keana, *J. Org. Chem.* **2003**, 68, 4862-4869.
http://dx.doi.org/10.1021/jo026923p

[10] A. Schaate, P. Roy, T. Preuße, S. J. Lohmeier, A. Godt, P. Behrens, *Chem. Eur. J.* **2011**, *17*, 9320-9325.
http://dx.doi.org/10.1002/chem.201101015

[11] S. R. Parsons, J. F. Hooper, M. C. Willis, *Org. Lett.* **2011**, *13*, 998-1000.
http://dx.doi.org/10.1021/ol1030662

[12] C. Blanchard, E. Framery, M. Vaultier, *Synthesis* **1996**, 45-47.
http://dx.doi.org/10.1055/s-0031-1289665

[13] L. Iannazzo, K. P. C. Vollhardt, M. Malacria, C. Aubert, V. Gandon, *Eur. J. Org. Chem.* **2011**, 3283-3292.
http://dx.doi.org/10.1002/ejoc.201100371

[14] S. L. MacNeil, M. Gray, D. G. Gusev, L. E. Briggs, V. Snieckus, *J. Org. Chem.* **2008**, *73*, 9710-9719. http://dx.doi.org/10.1021/jo801856n

[15] J. Clayden, L. Vallverdu, J. Clayton, M. Helliwell, *Chem. Commun.* **2008**, *5*, 561-563. http://dx.doi.org/10.1039/B716105K

[16] G. Lopez-Valdez, S. Oliguín-Uribe, L. D. Miranda, *Tetrahedron. Lett.* **2007**, *48*, 8285-8289. http://dx.doi.org/10.1016/j.tetlet.2007.09.142

[17] A. Arizpe, F. J. Sayago, A. I. Jimenez, C. Cativiela, M. Ordonez, *Eur. J. Org. Chem.* **2011**, 6732-6738. http://dx.doi.org/10.1002/ejoc.201101014

[18] H. Chiba, S. Oishi, N. Fujii, H. Ohno, *Angew. Chem. Int. Ed.* **2012**, *51*, 9169–9172. http://dx.doi.org/10.1002/anie.201205106

NMR Spectra

3-(Trimethylsilyl)propiolic acid

^{1}H NMR (400 MHz, $CDCl_3$)

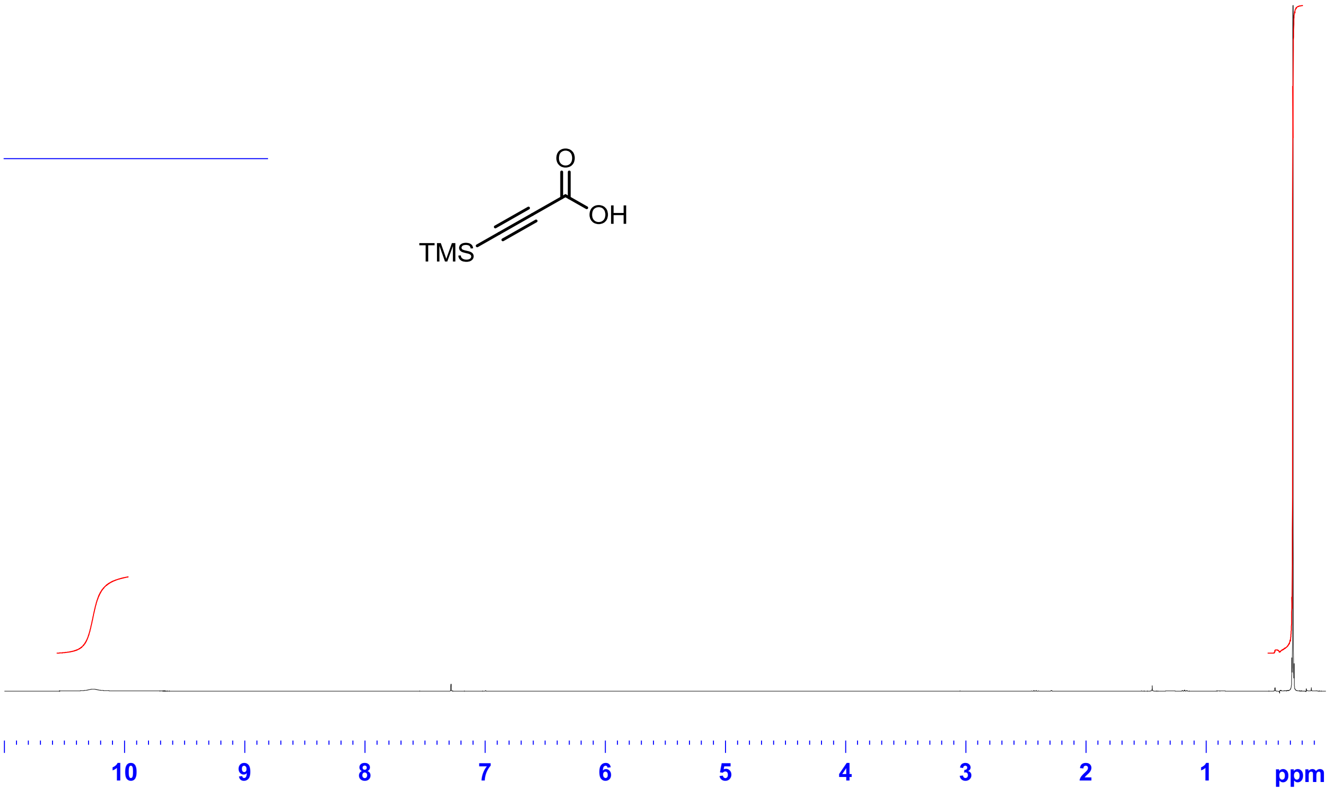

^{13}C NMR (100 MHz, $CDCl_3$)

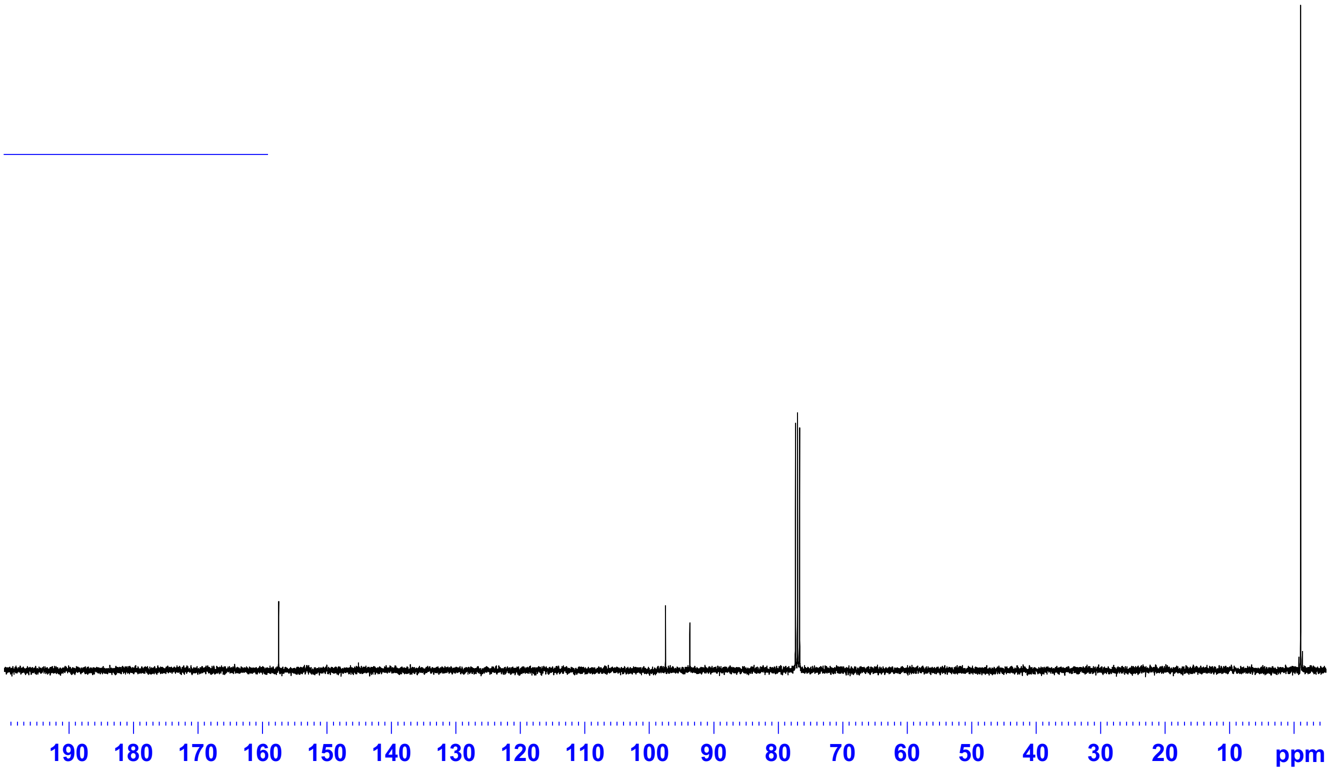

***N*-Benzylprop-2-yn-1-amine 7a**

^{1}H NMR (400 MHz, $CDCl_3$)

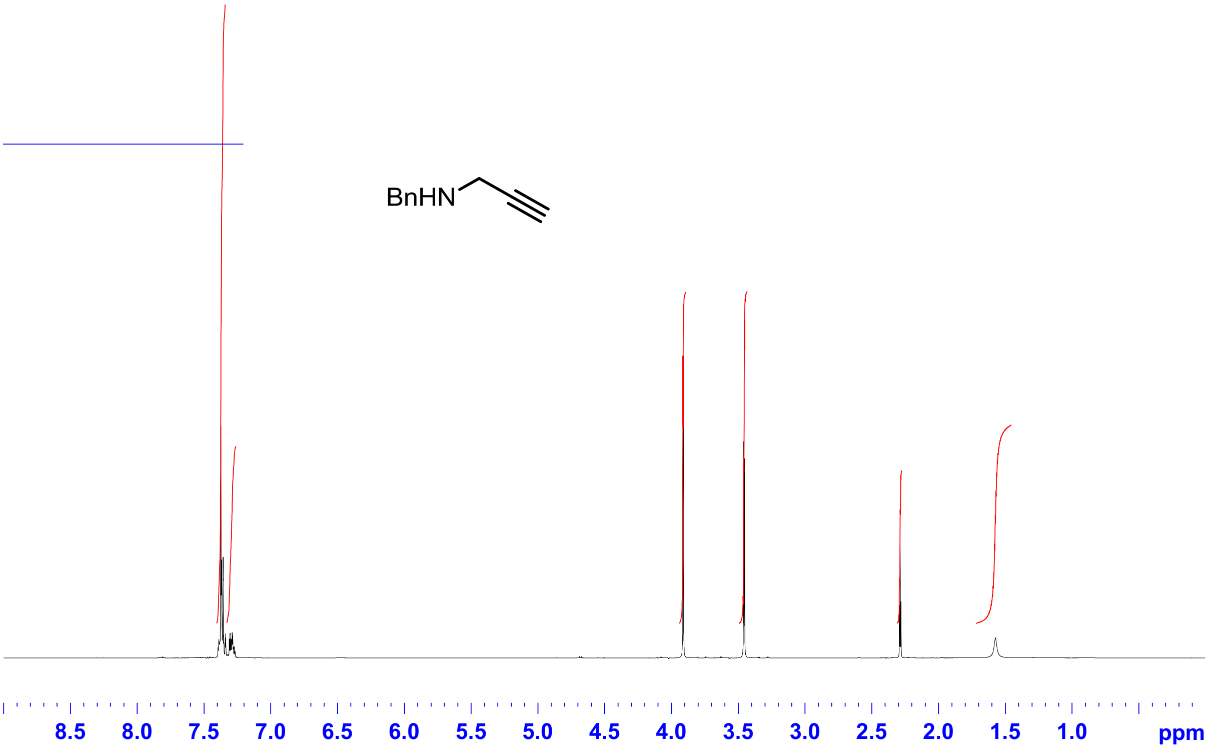

^{13}C NMR (100 MHz, $CDCl_3$)

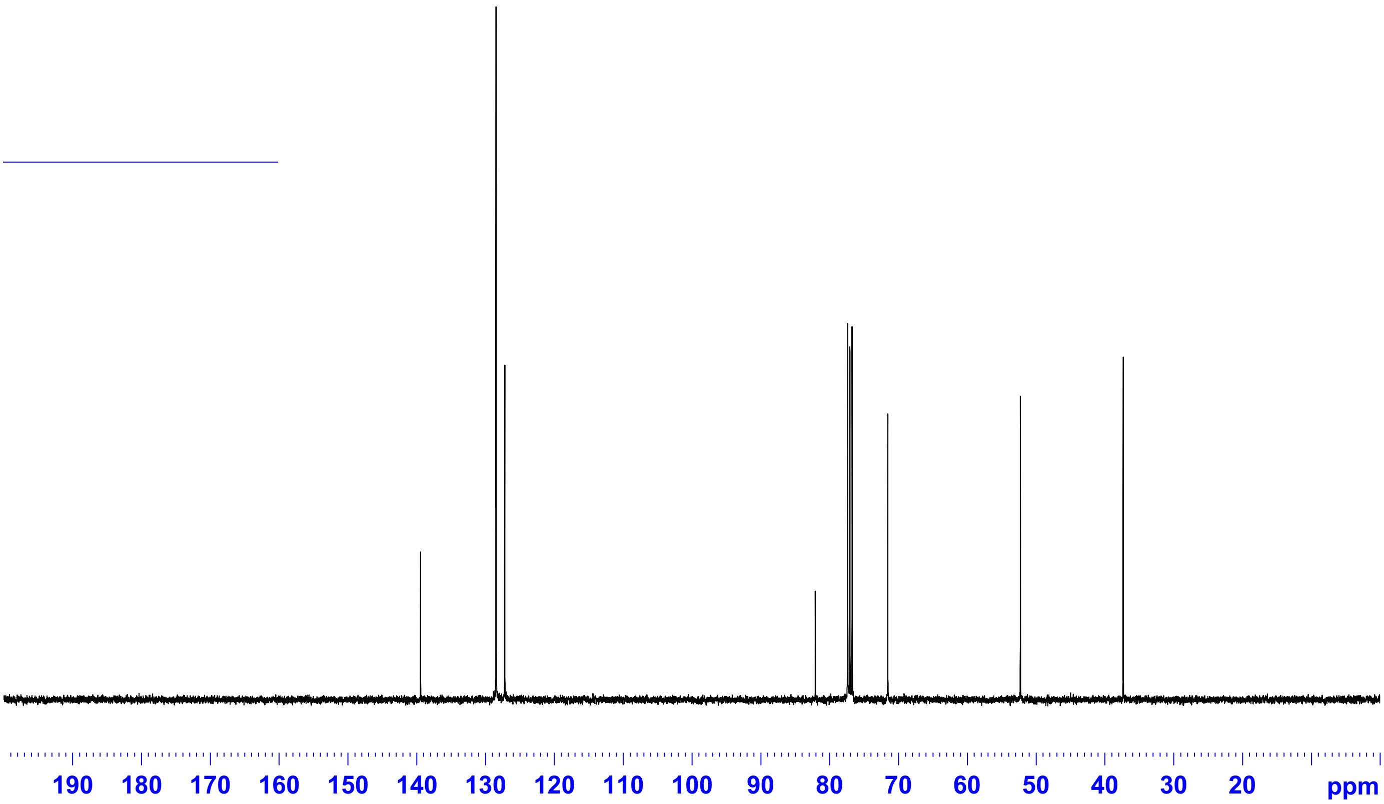

***N*-(*tert*-Butyl)prop-2-yn-1-amine 7b**

^{1}H NMR (600 MHz, DMSO-d$_6$)

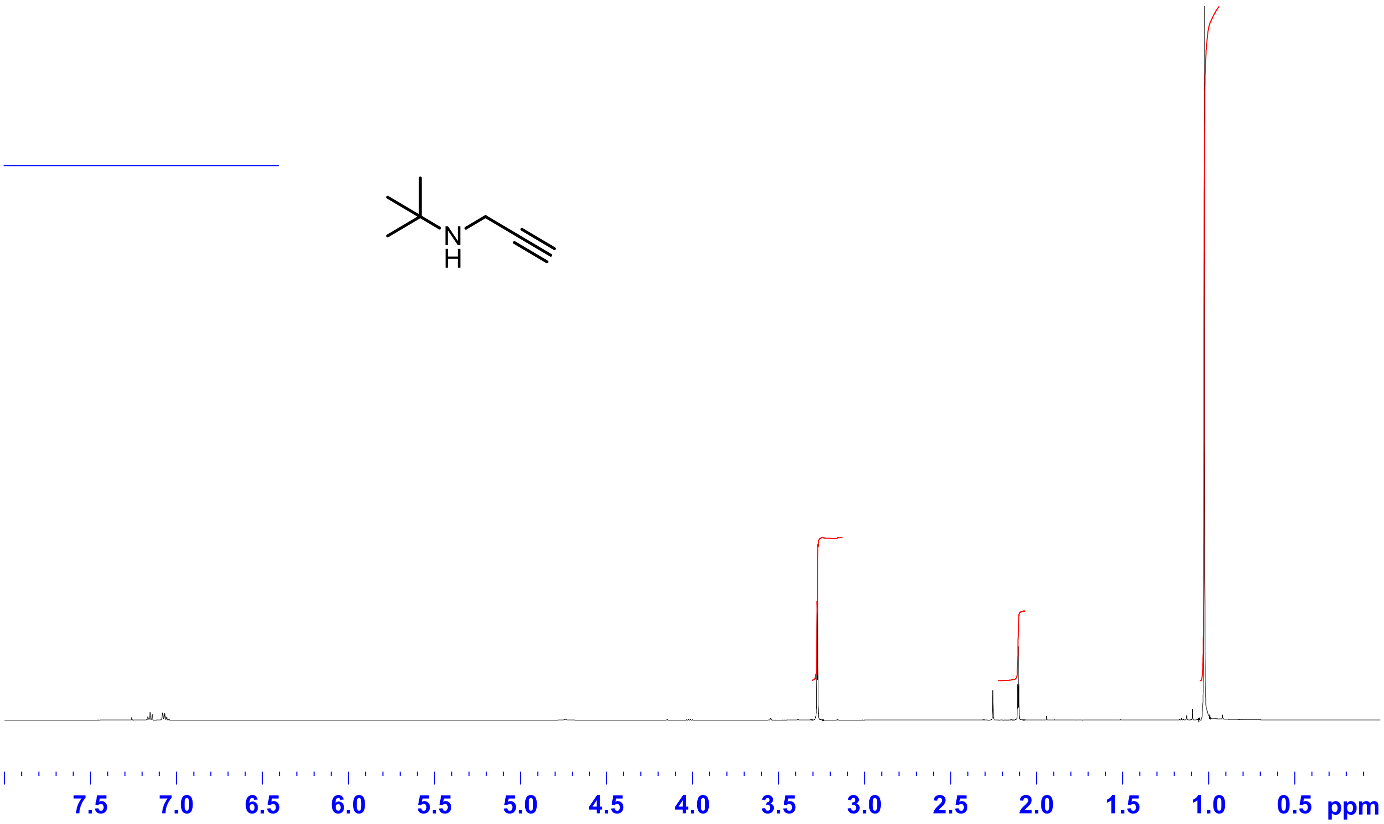

^{13}C NMR (150 MHz, DMSO-d$_6$)

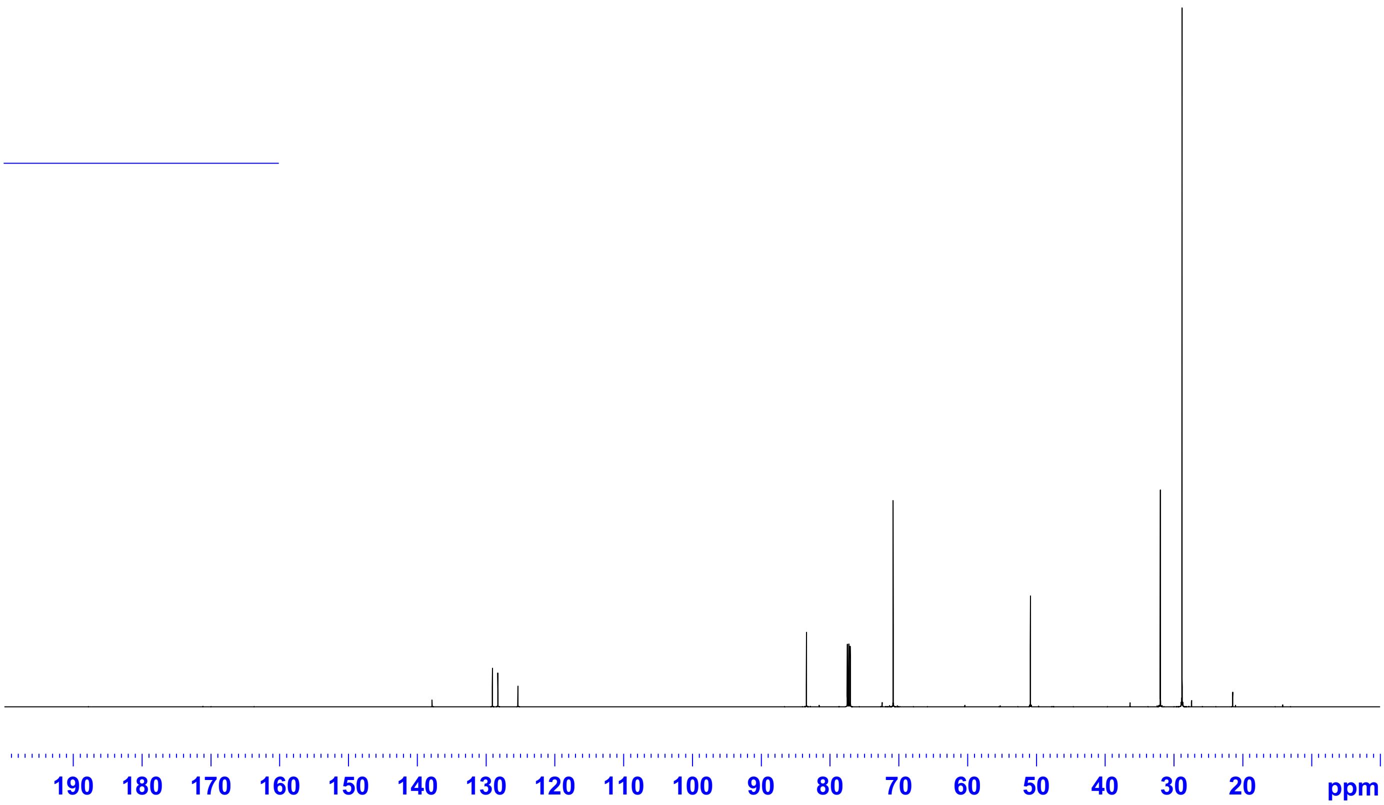

***N*-Benzylbut-2-yn-1-amine 7d**

^{1}H NMR (400 MHz, $CDCl_3$)

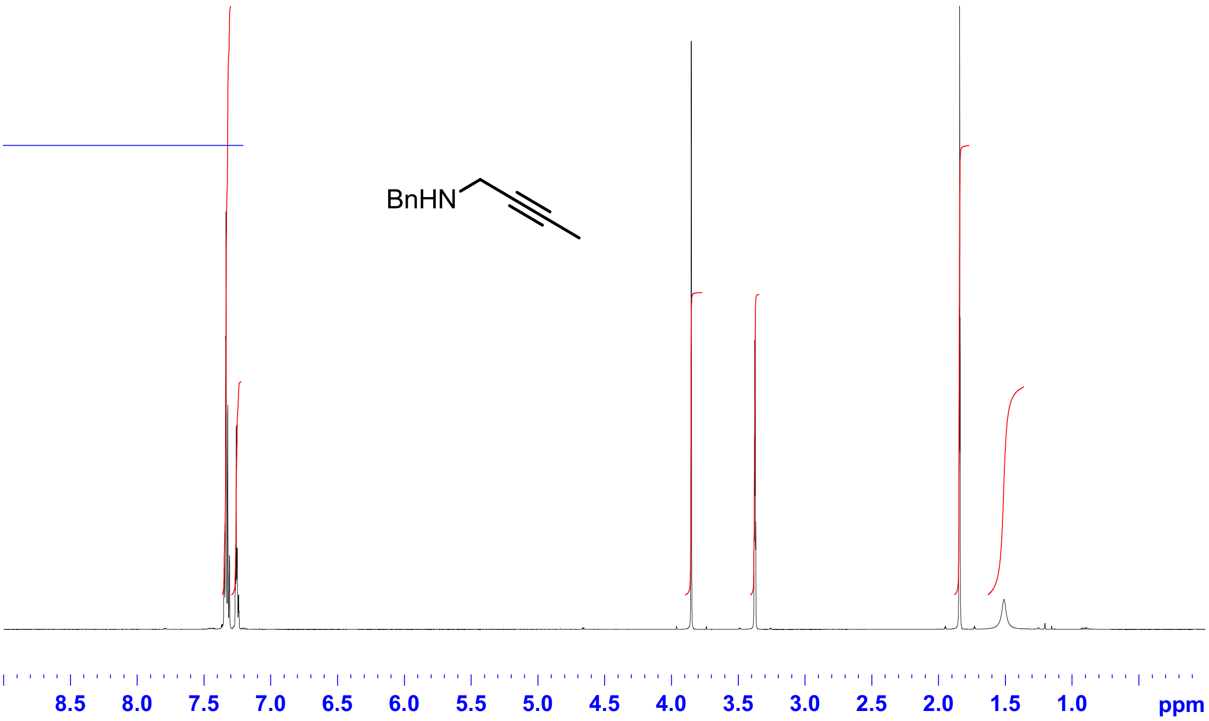

^{13}C NMR (150 MHz, $CDCl_3$)

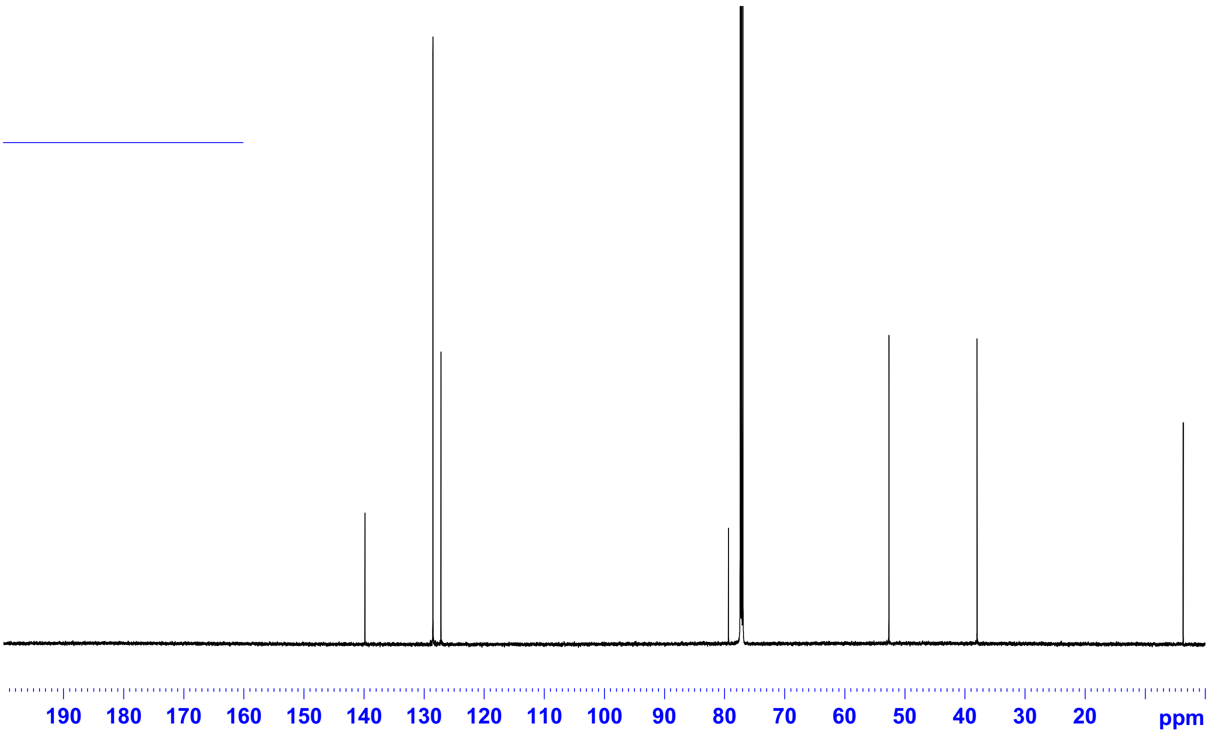

***N*-Benzylpent-2-yn-1-amine 7e**

^{1}H NMR (400 MHz, $CDCl_3$)

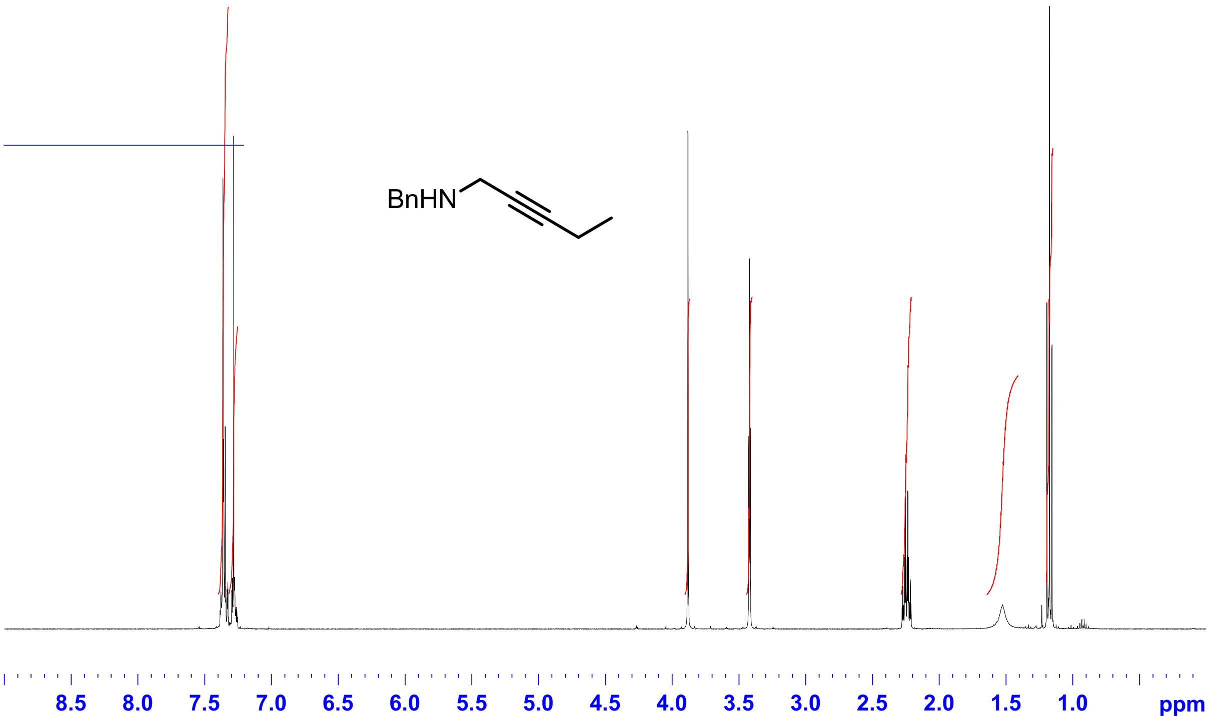

^{13}C NMR (150 MHz, $CDCl_3$)

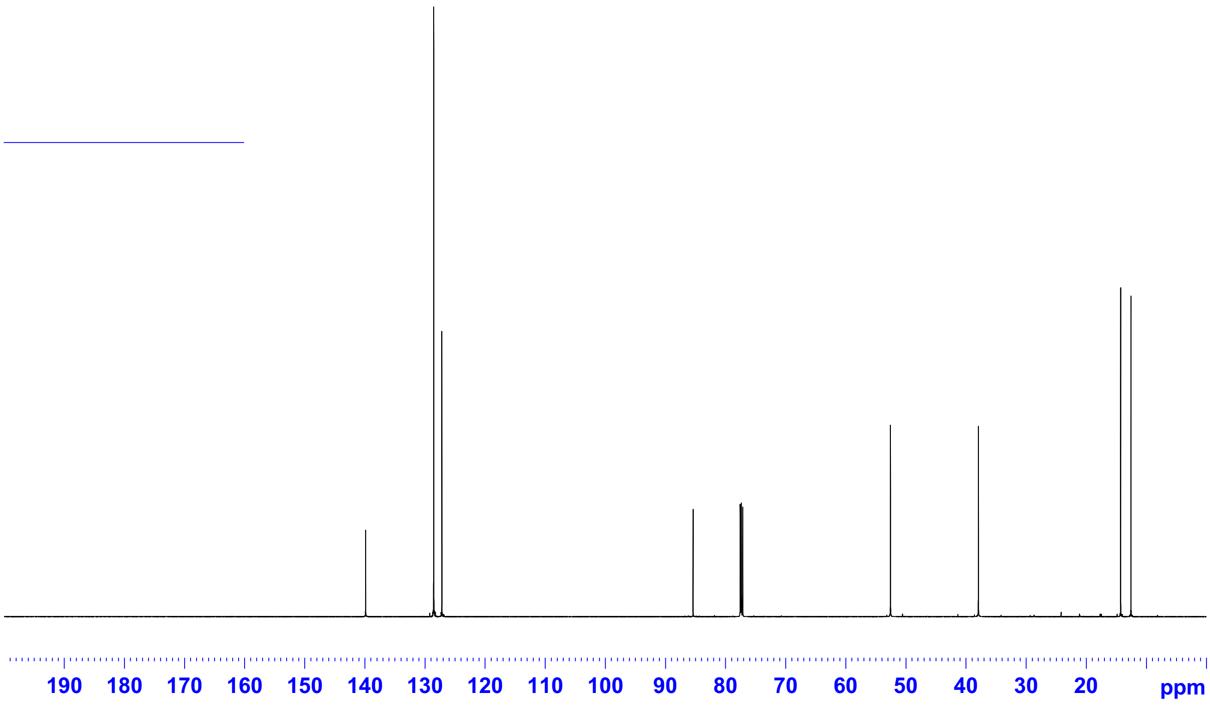

***N*-Benzyl-*N*-(prop-2-yn-1-yl)-3-(trimethylsilyl)propiolamide 6a**

^{1}H NMR (400 MHz, $CDCl_3$)

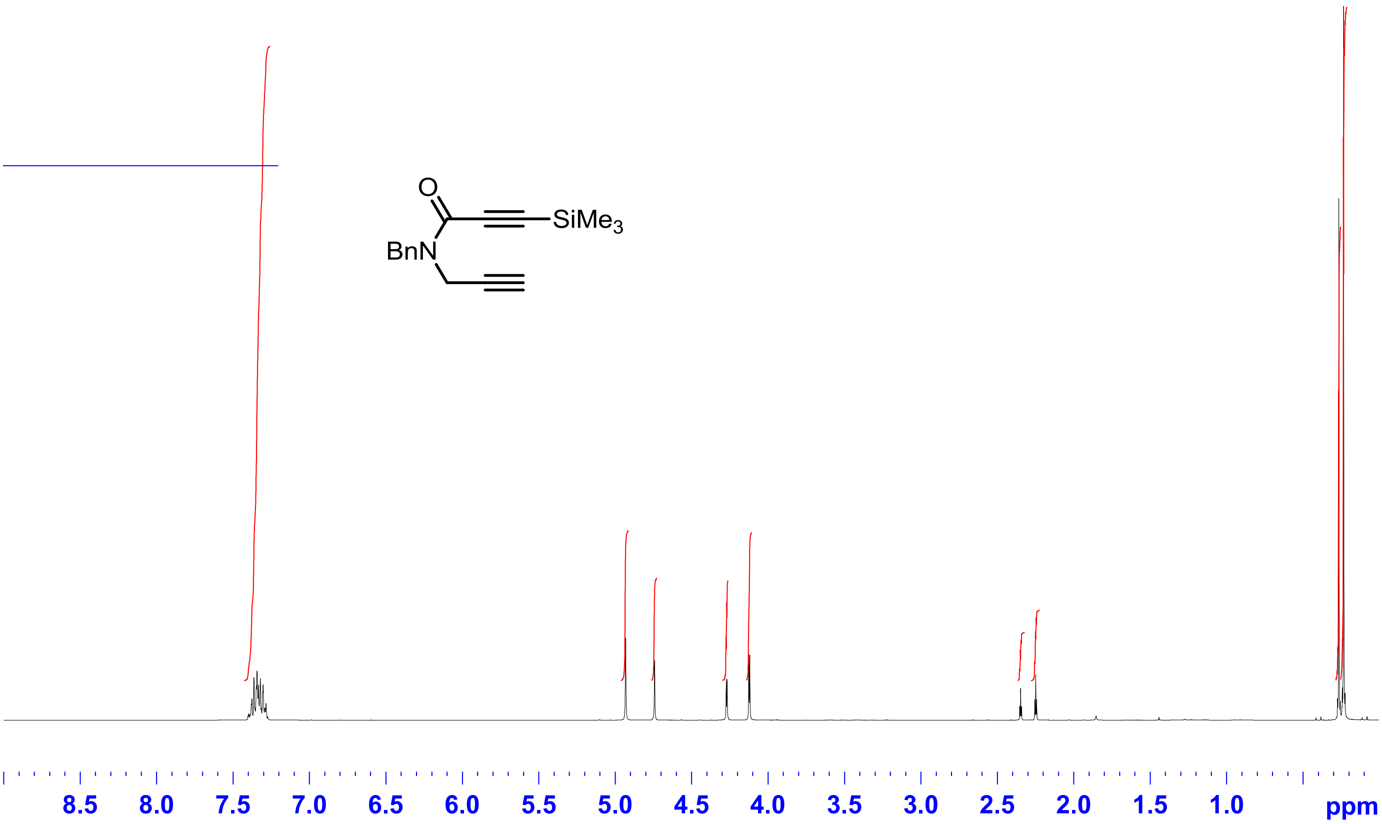

^{13}C NMR (100 MHz, $CDCl_3$)

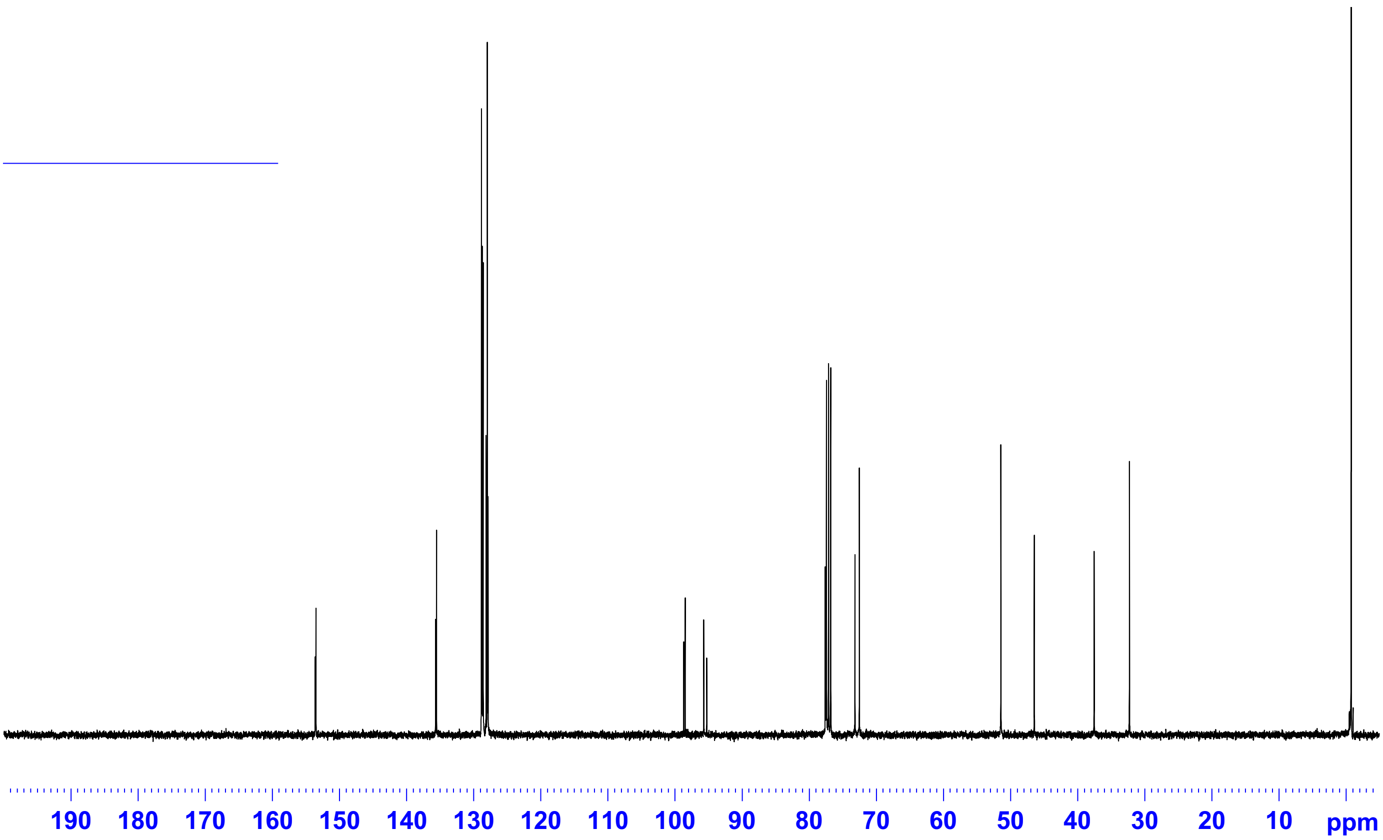

_N_-(_tert_-Butyl)-_N_-(prop-2-yn-1-yl)-3-(trimethylsilyl)propiolamide 6b

^{1}H NMR (600 MHz, DMSO-d$_6$)

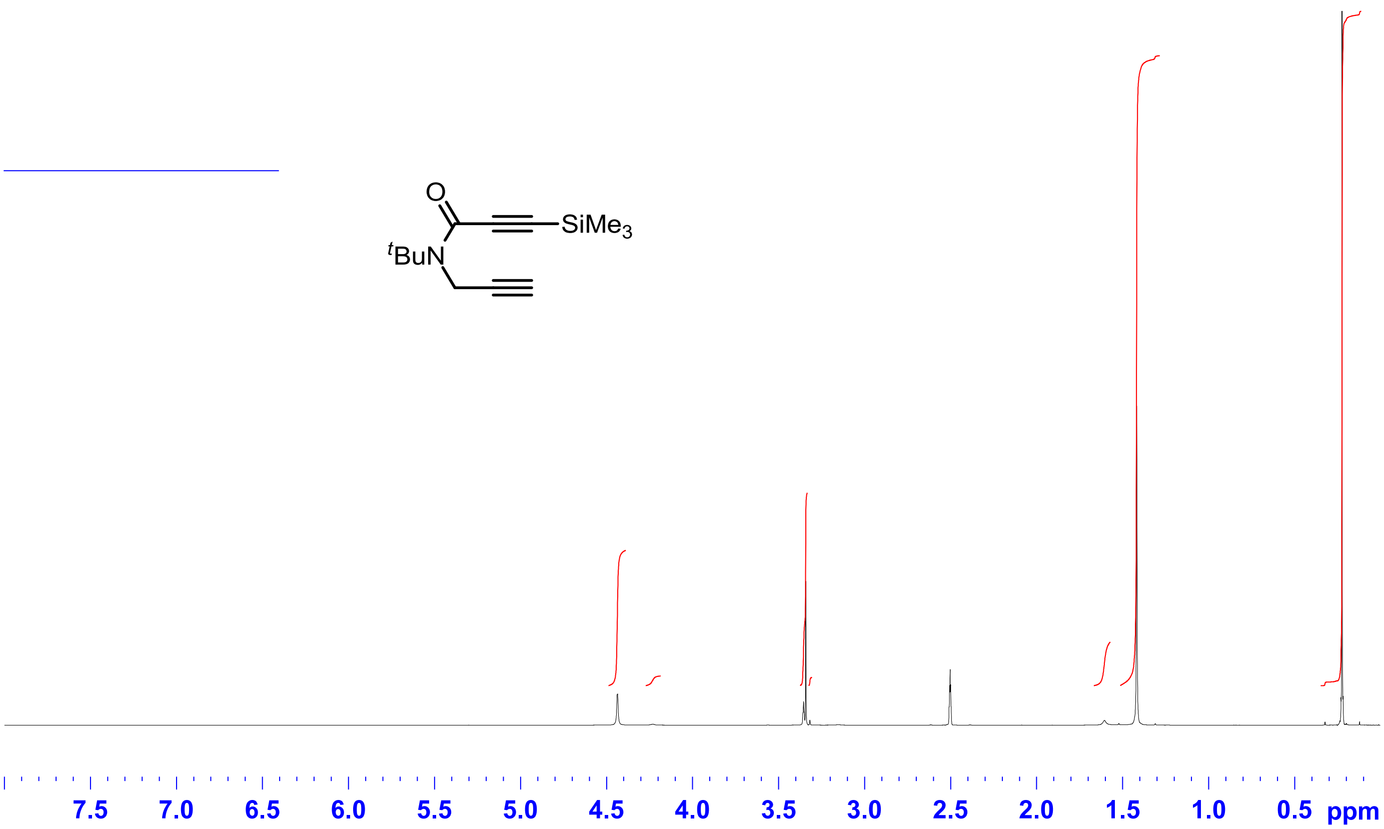

^{13}C NMR (150 MHz, DMSO-d$_6$)

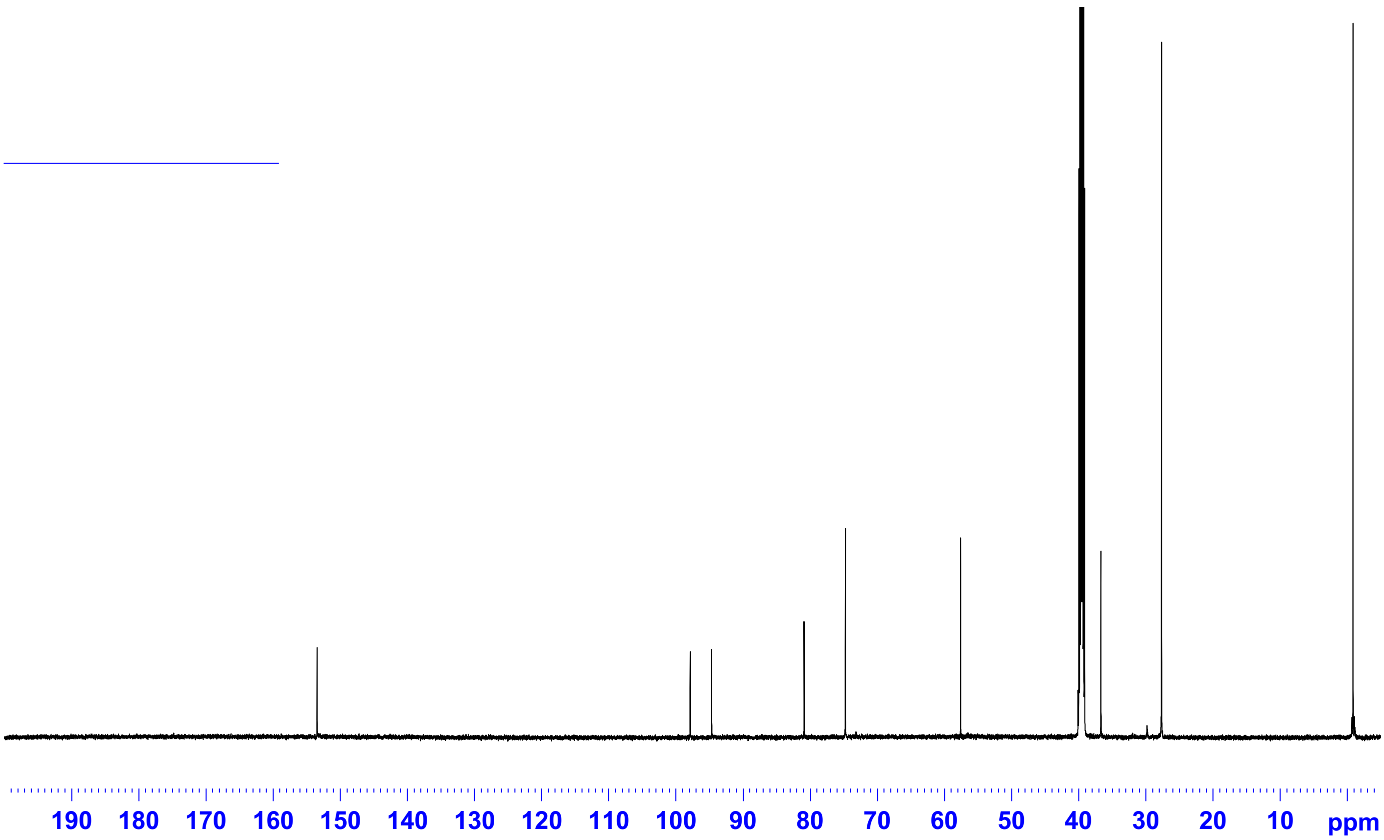

_N_-(Prop-2-yn-1-yl)-3-(trimethylsilyl)propiolamide 6c

^{1}H NMR (600 MHz, DMSO-d$_6$)

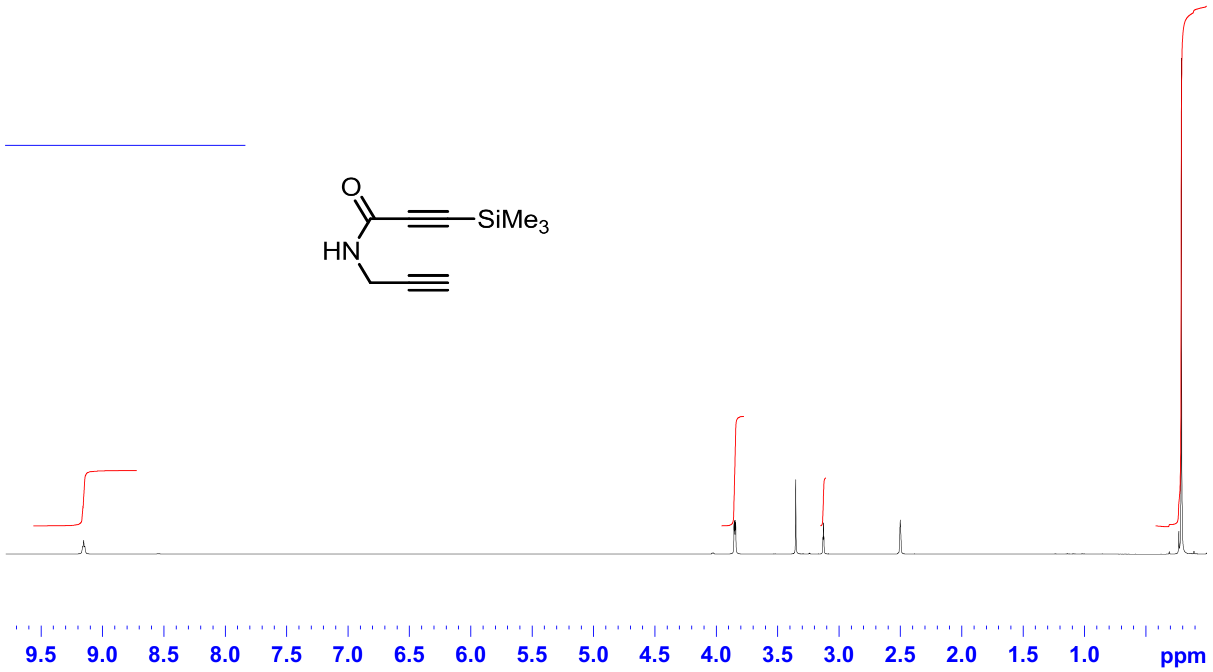

^{13}C NMR (150 MHz, DMSO-d$_6$)

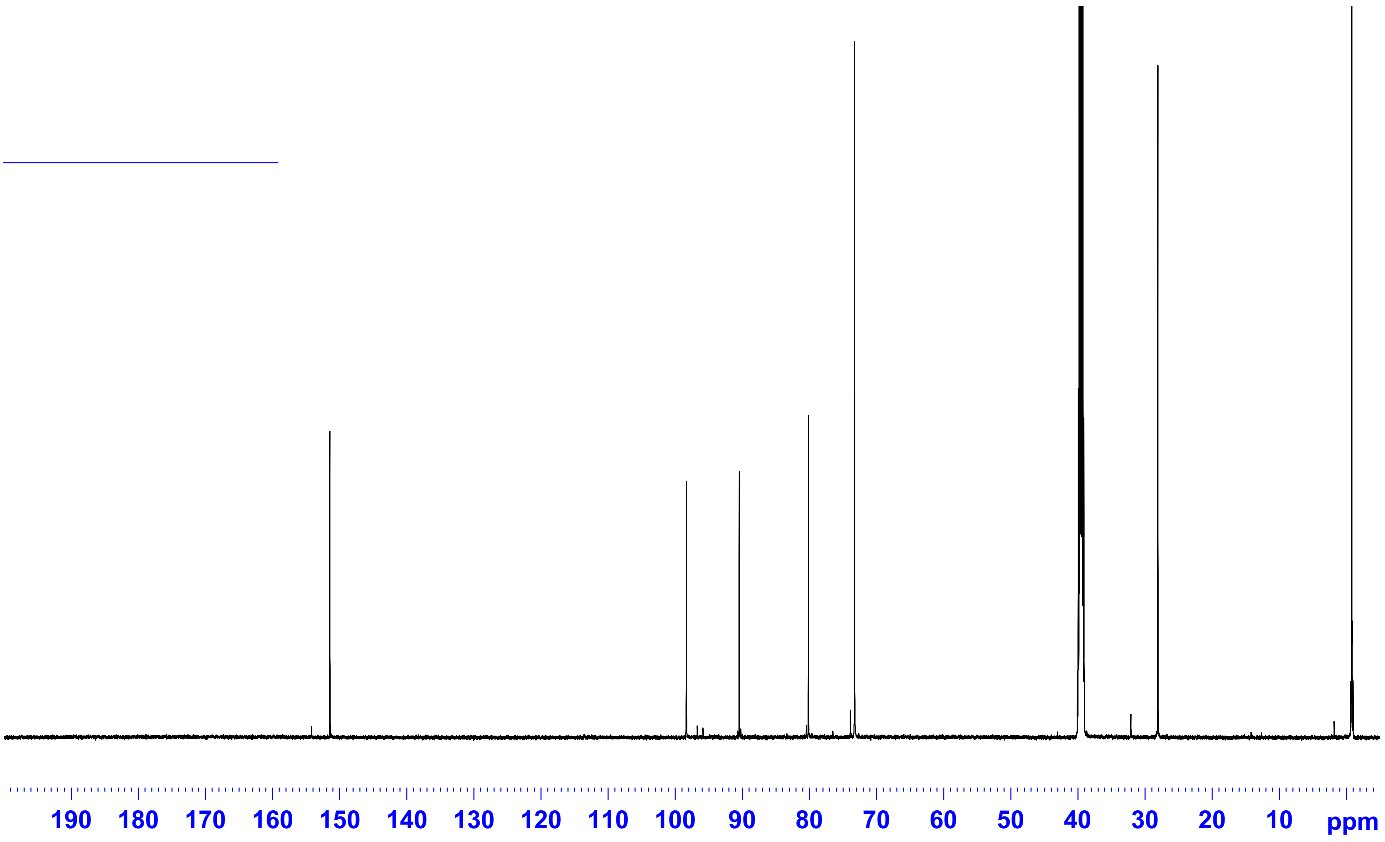

***N*-Benzyl-*N*-(but-2-yn-1-yl)-3-(trimethylsilyl)propiolamide 6d**

^{1}H NMR (600 MHz, DMSO-d_6)

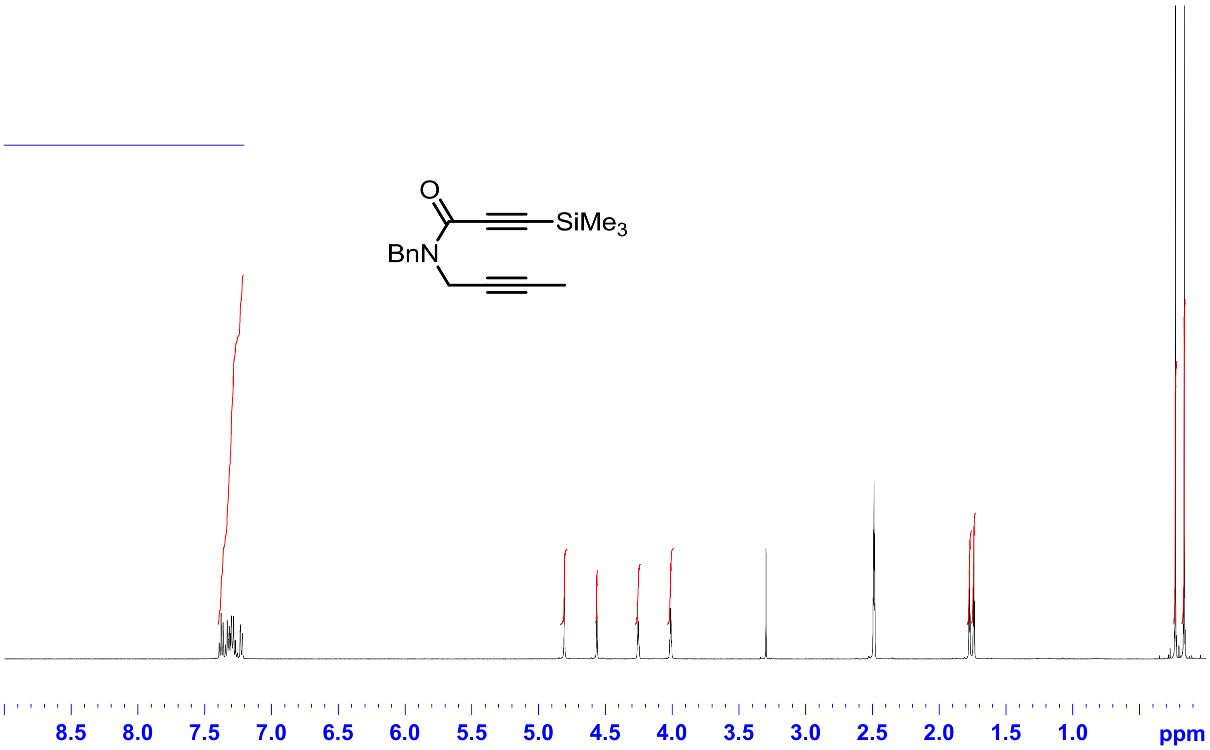

^{13}C NMR (150 MHz, DMSO-d_6)

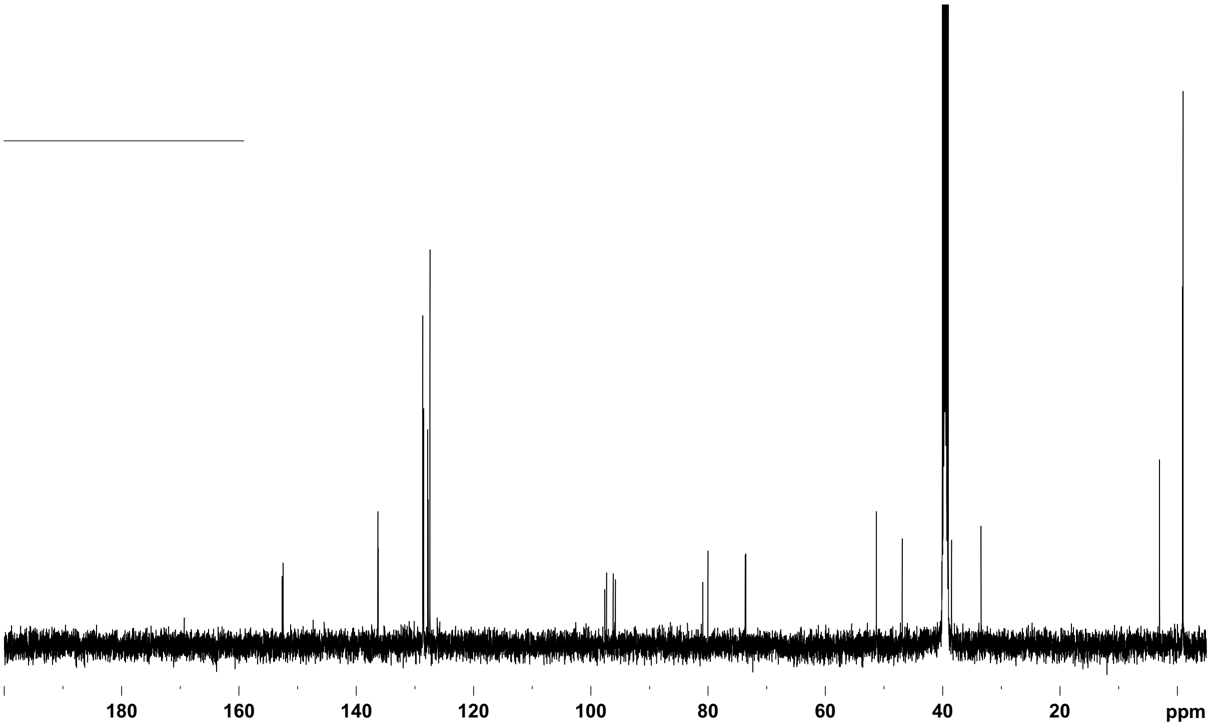

***N*-Benzyl-*N*-(pent-2-yn-1-yl)-3-(trimethylsilyl)propiolamide 6e**

^{1}H NMR (600 MHz, DMSO-d$_6$)

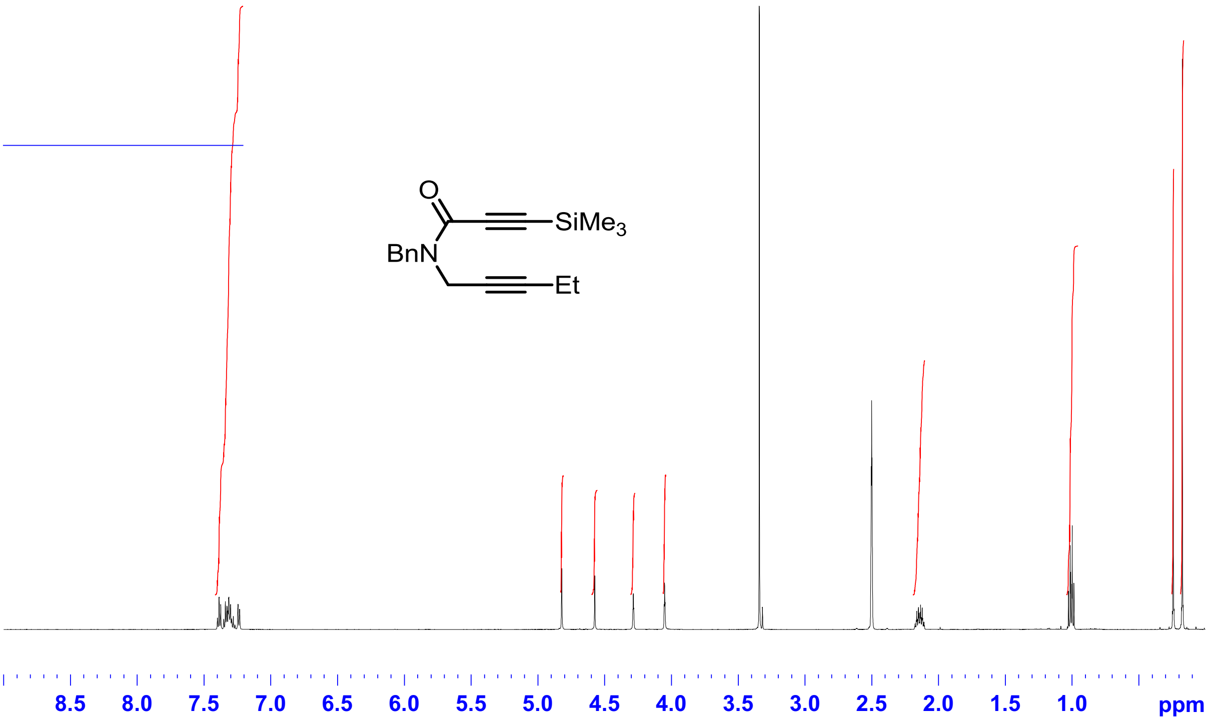

^{13}C NMR (150 MHz, DMSO-d$_6$)

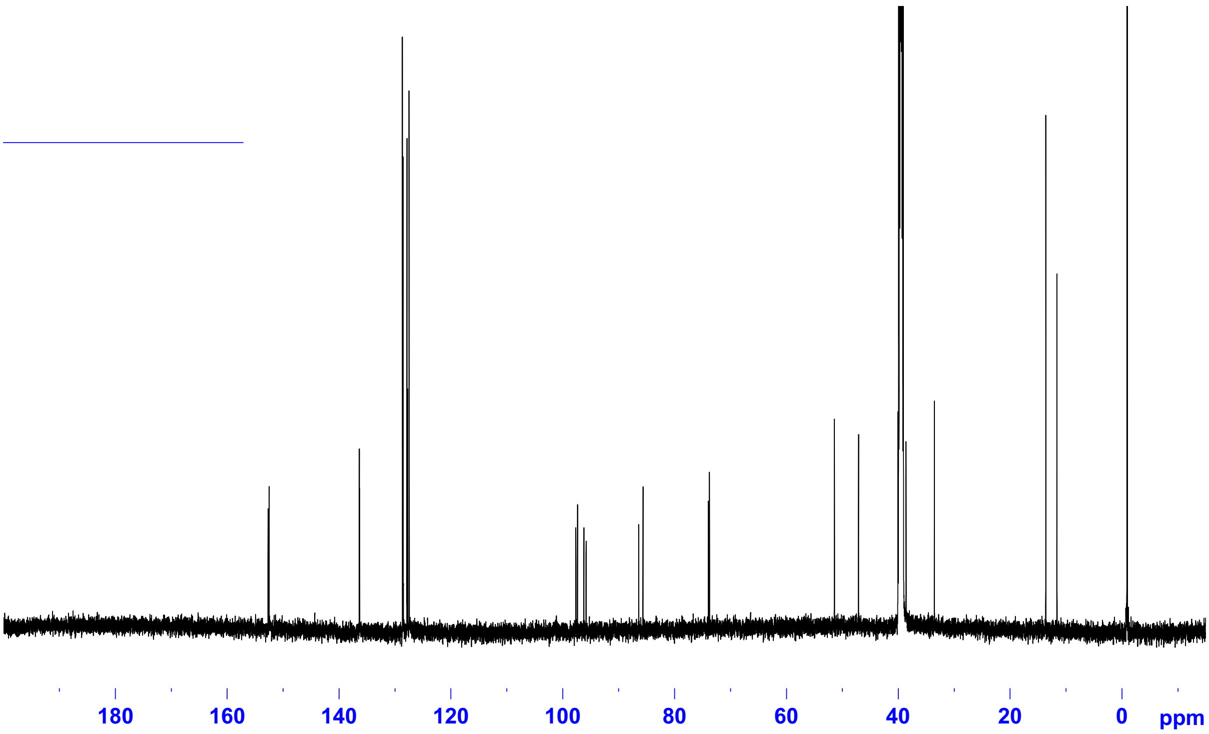

***N*-Benzyl-*N*-(prop-2-yn-1-yl)but-2-ynamide 6f**

^{1}H NMR (600 MHz, DMSO-d$_6$)

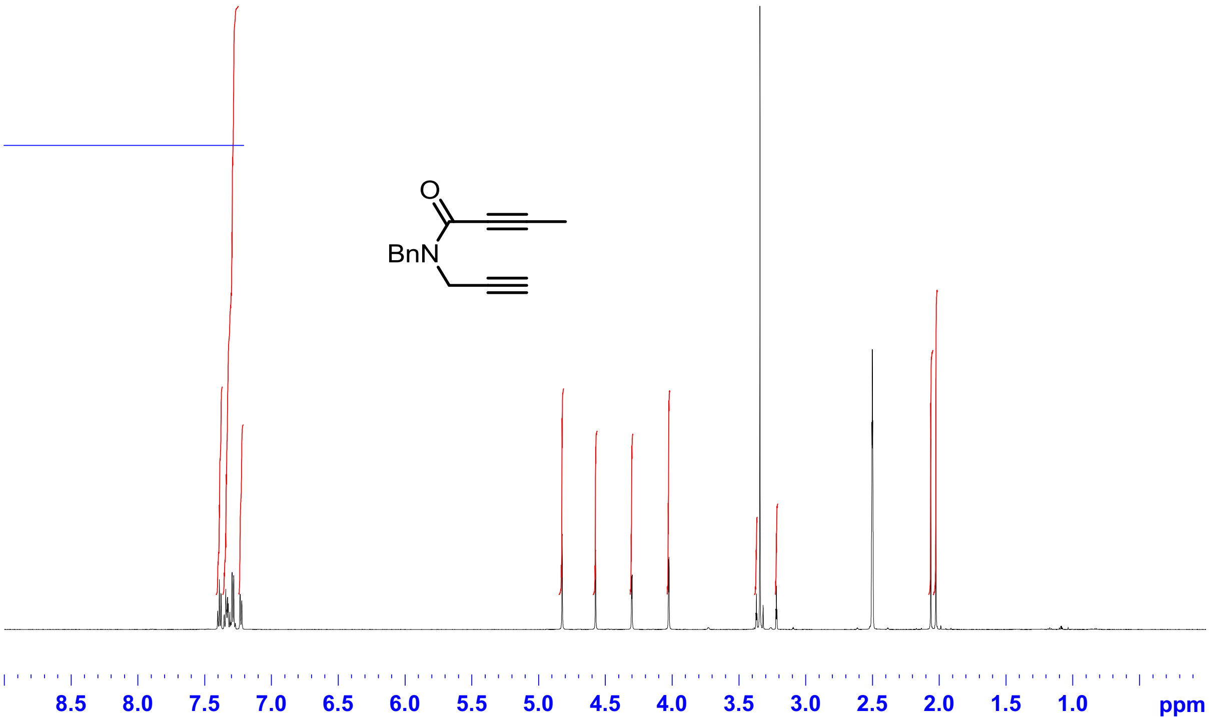

^{13}C NMR (150 MHz, DMSO-d$_6$)

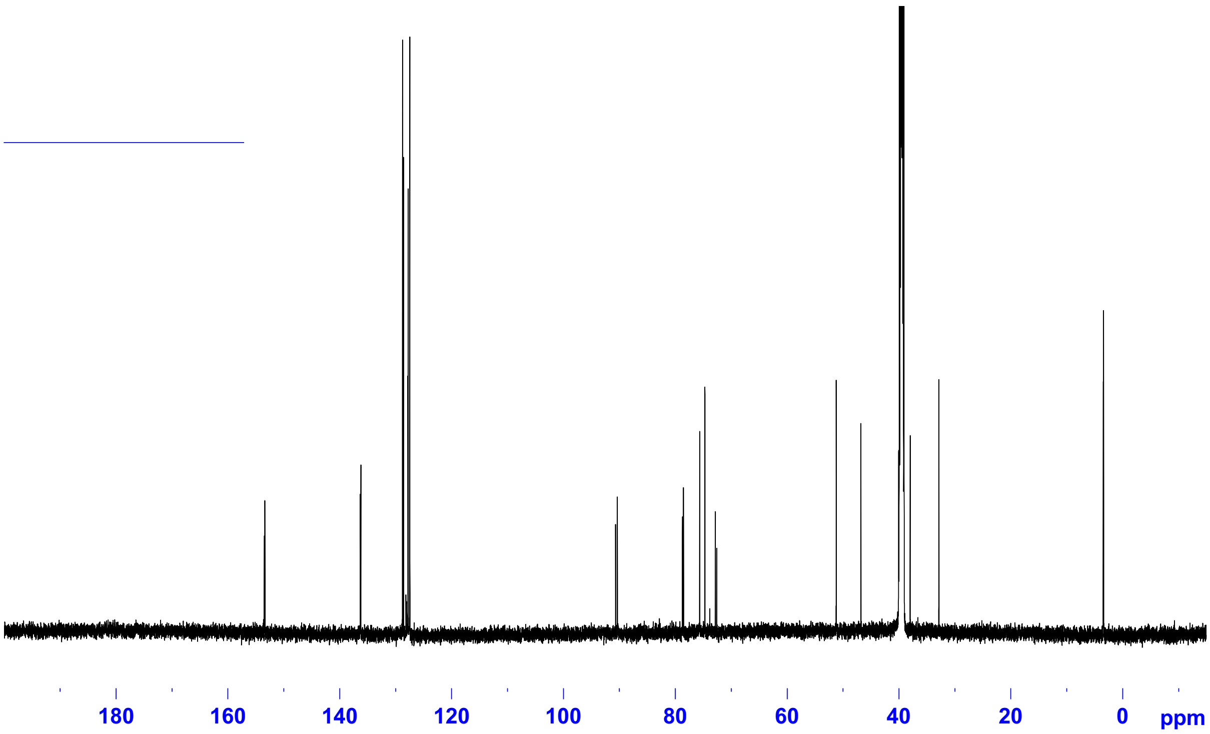

***tert*-Butyl prop-2-yn-1-ylcarbamate 9f**

^{1}H NMR (500 MHz, DMSO-d$_6$)

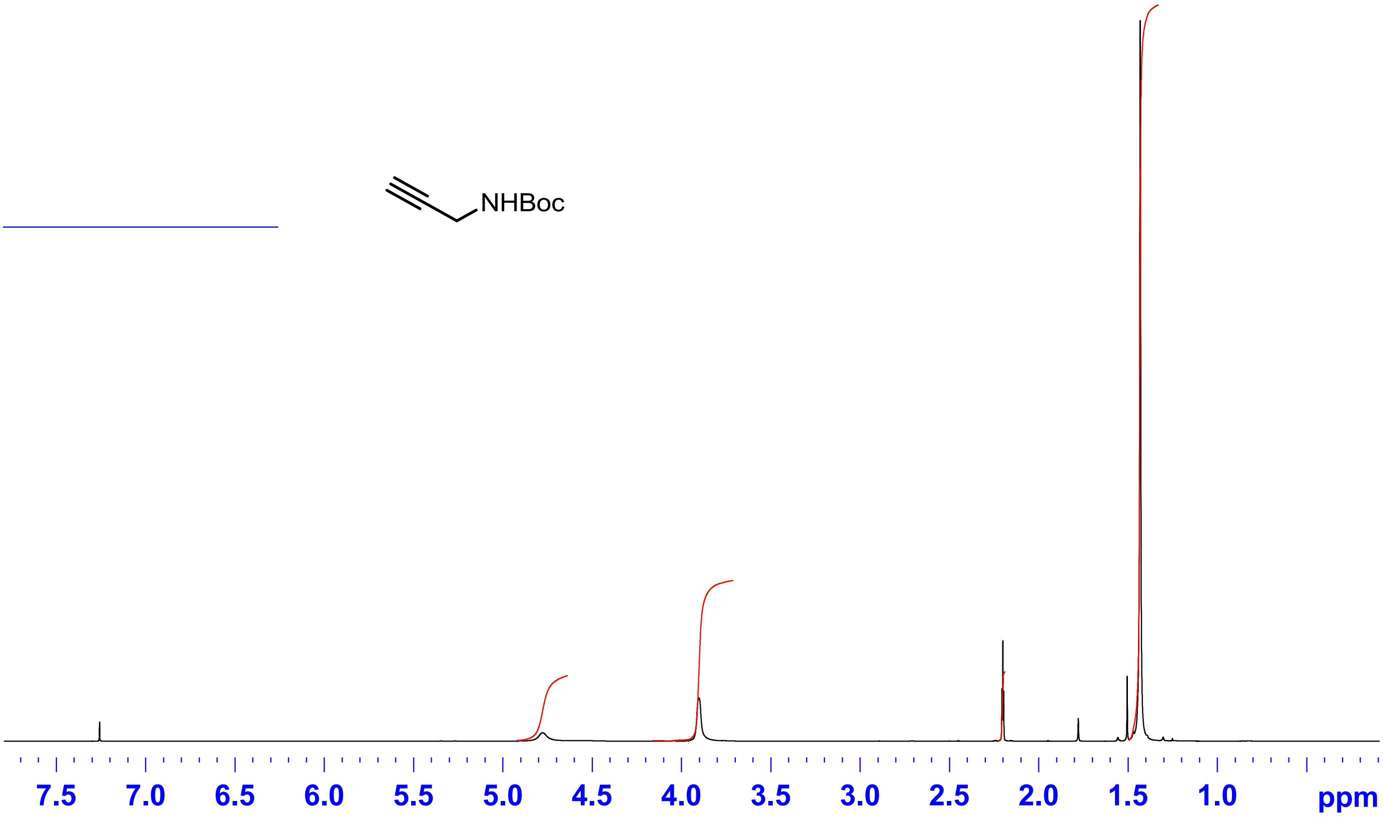

^{13}C NMR (125 MHz, DMSO-d$_6$)

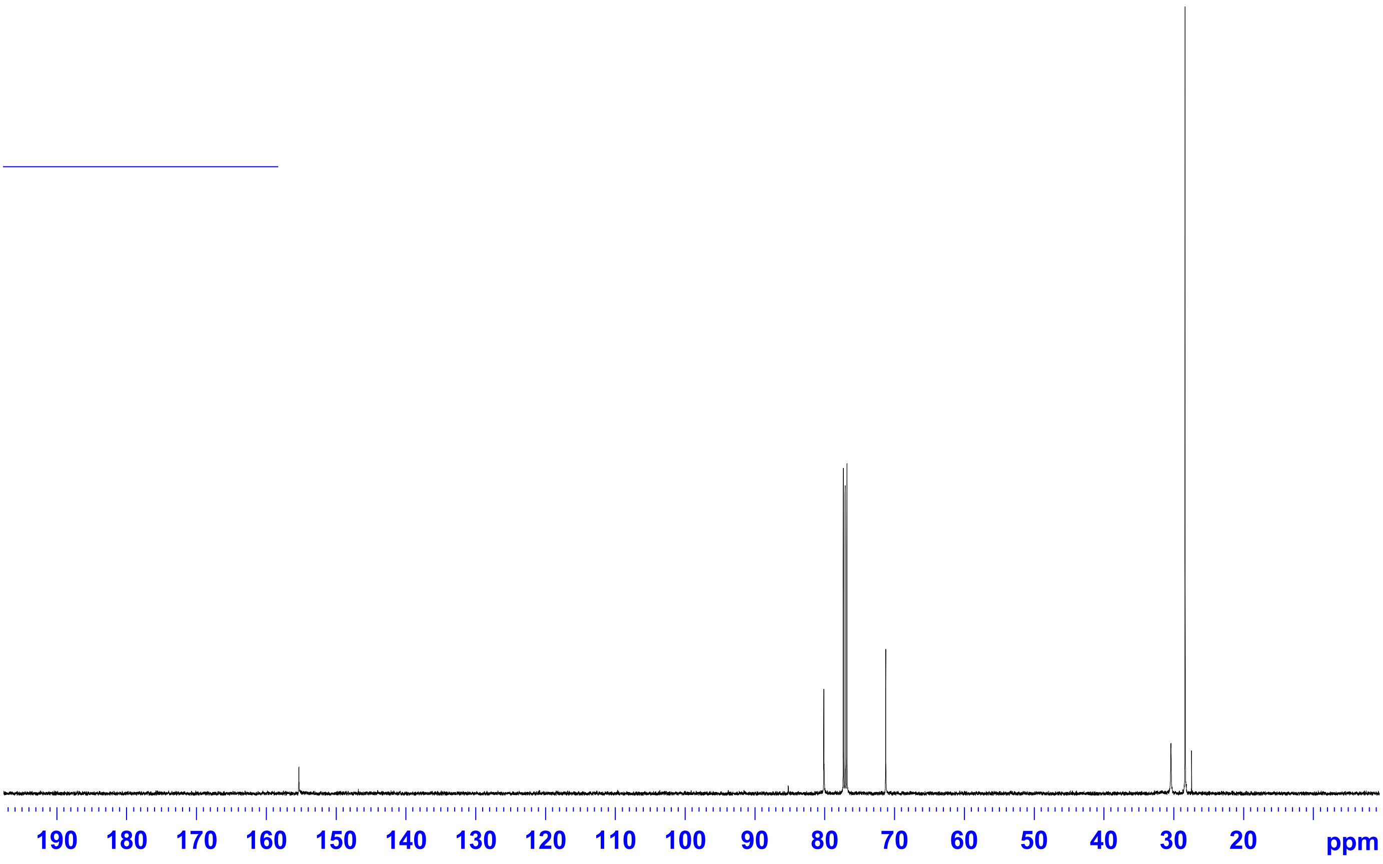

Ethyl 4-((trimethylsilyl)ethynyl)benzoate

^{1}H NMR (500 MHz, $CDCl_3$)

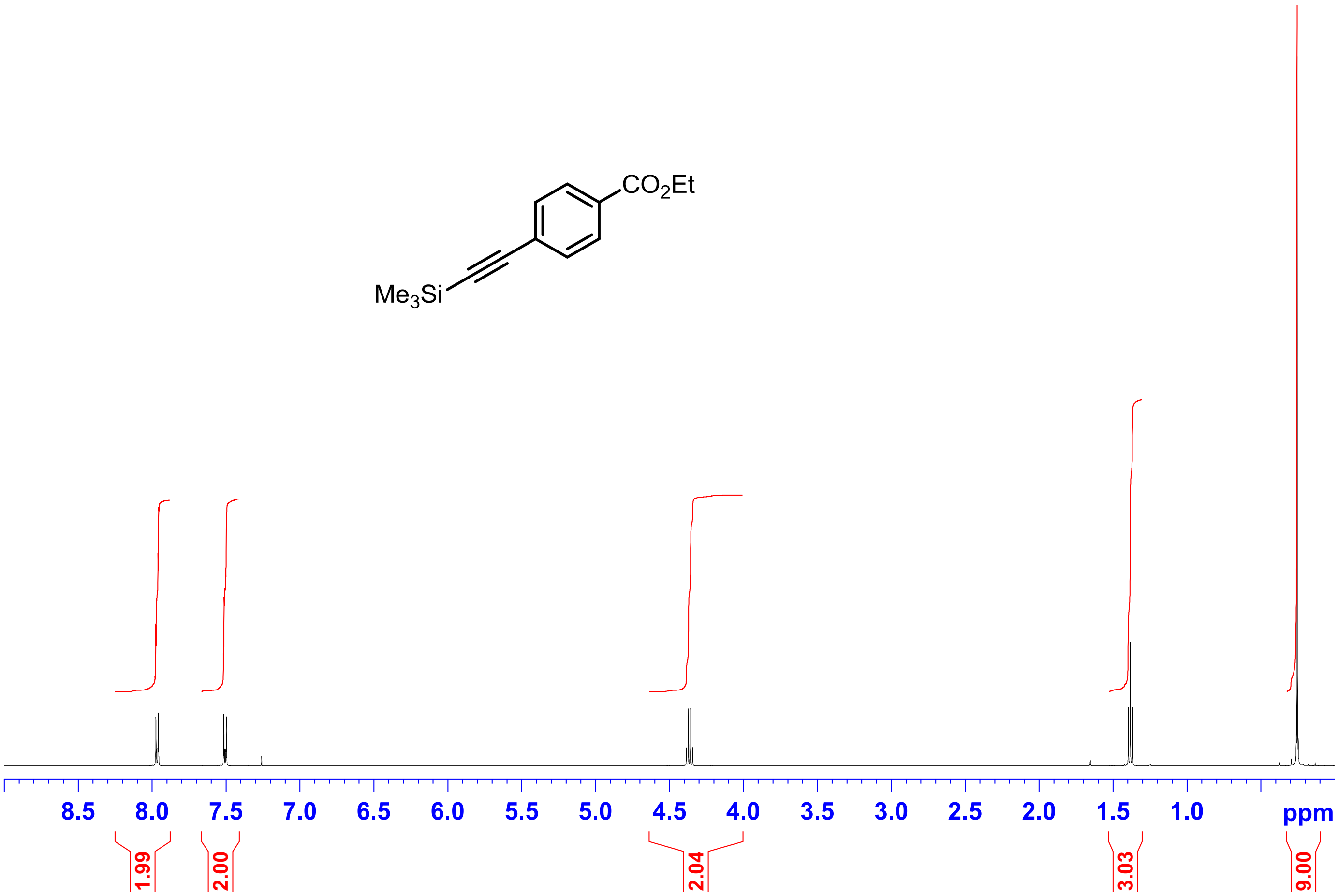

^{13}C NMR (125 MHz, $CDCl_3$)

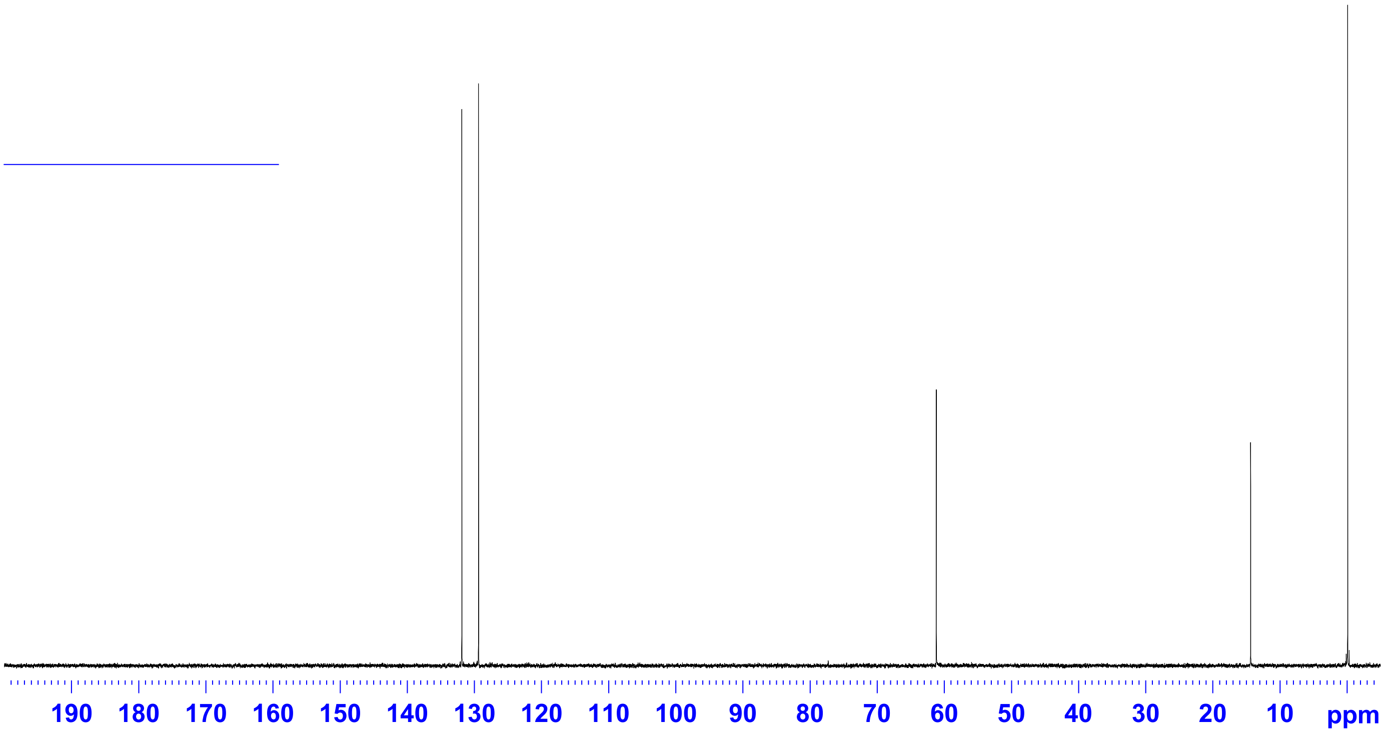

Methyl 4-ethynylbenzoate 9q

^{1}H NMR (500 MHz, $CDCl_3$)

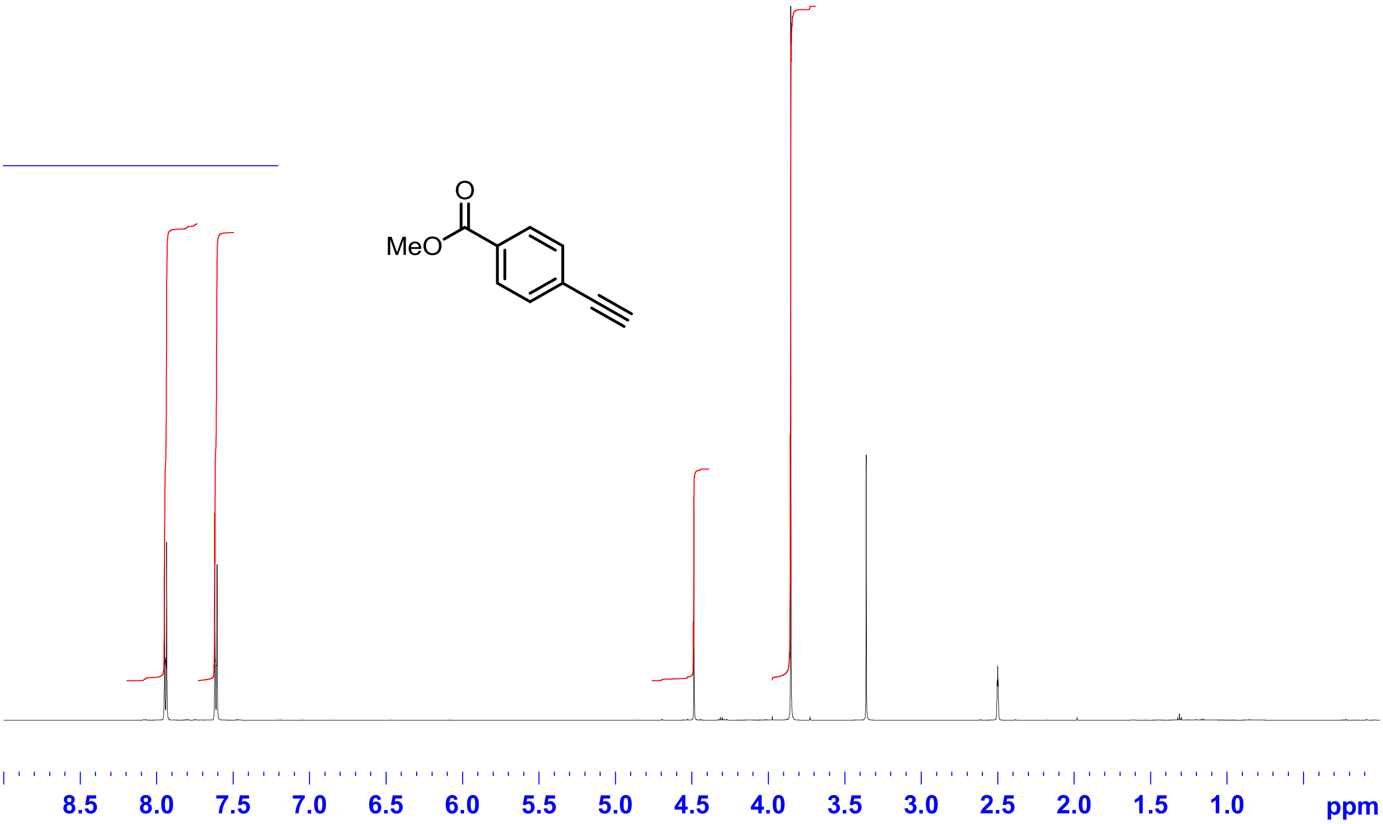

^{13}C NMR (125 MHz, $CDCl_3$)

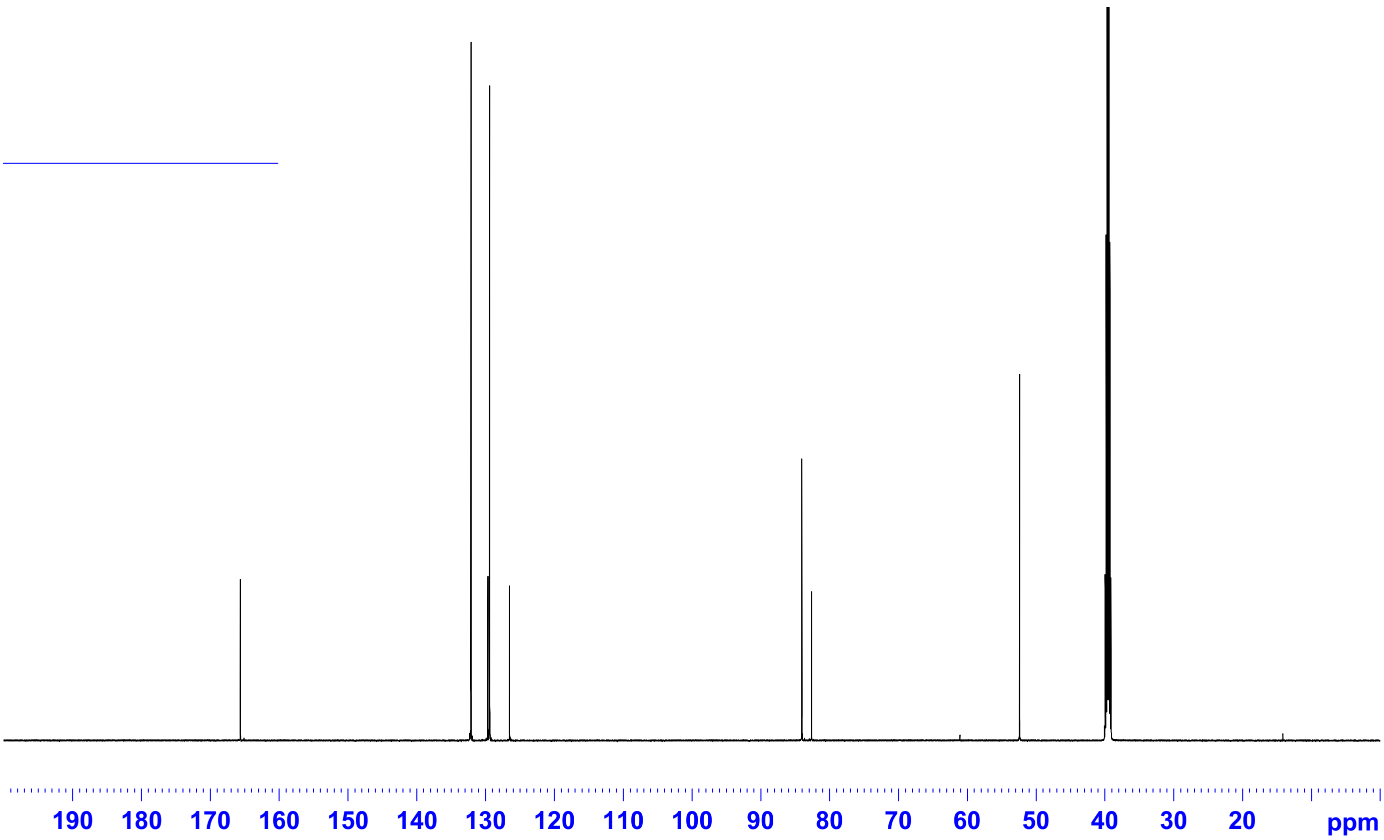

2,2,5,5,8,8-Hexamethyl-3,7-dioxa-2,8-disilanonane

^{1}H NMR (500 MHz, $CDCl_3$)

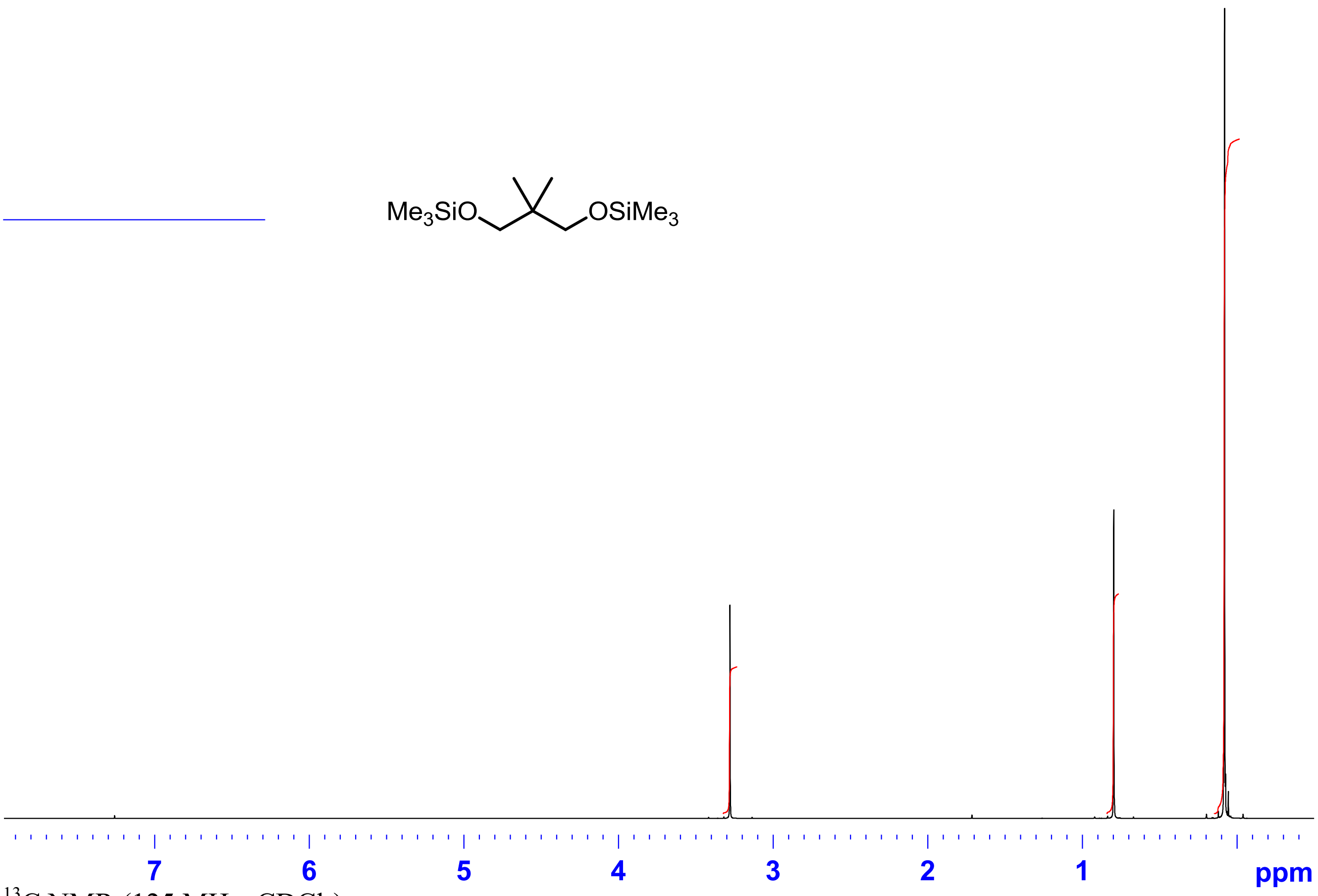

^{13}C NMR (125 MHz, $CDCl_3$)

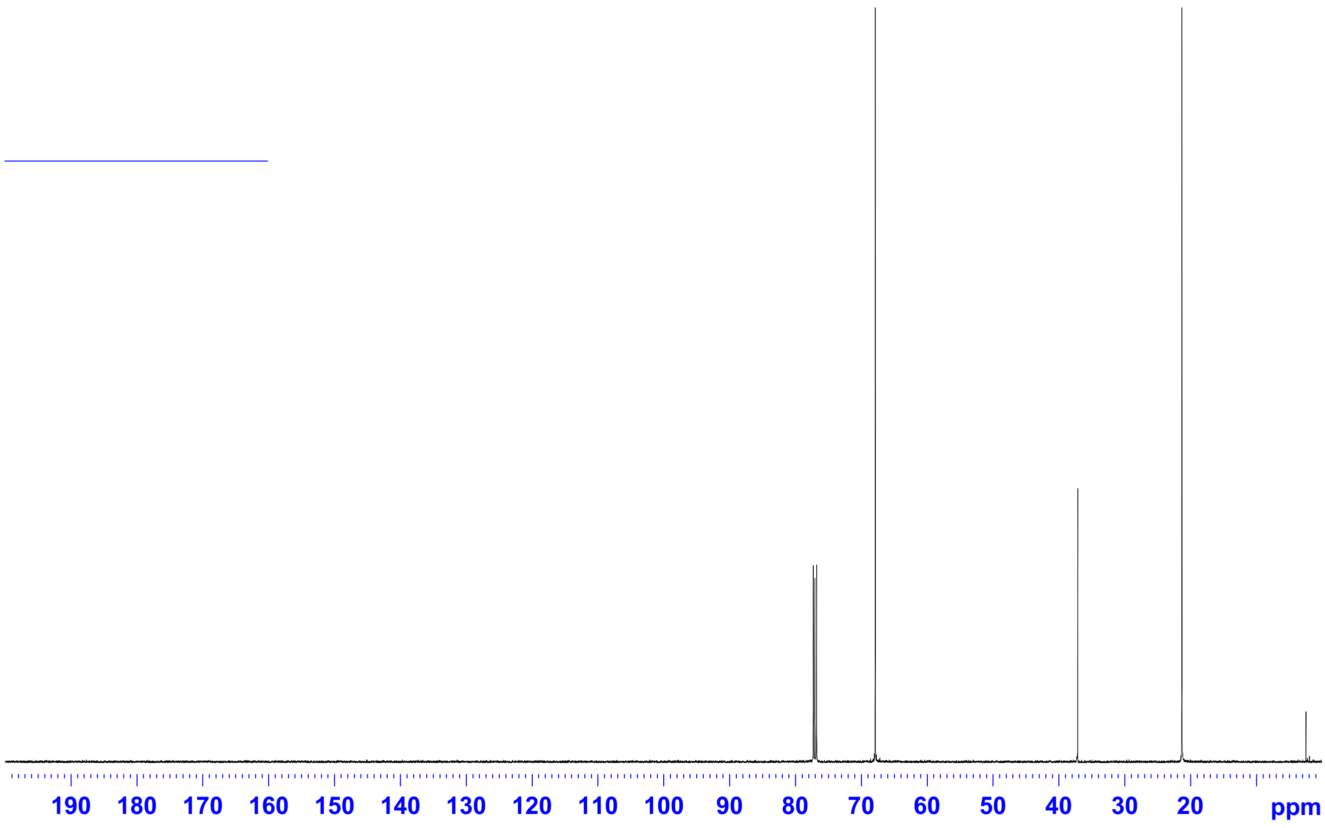

2-Ethynyl-2,3-dihydro-1*H*-naphtho[1,8-*de*][1,3,2]diazaborinine 9v

^{1}H NMR (600 MHz, DMSO-d$_6$)

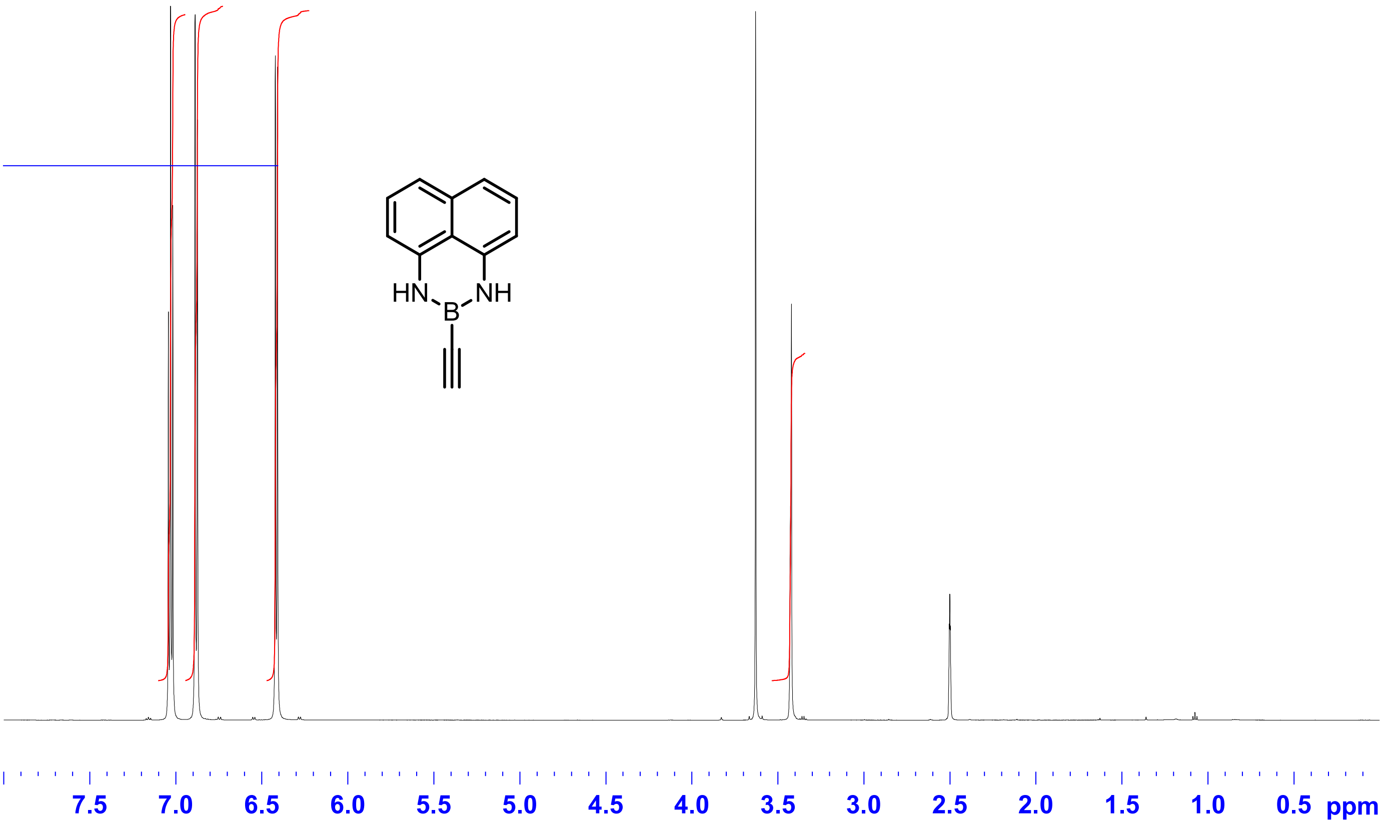

^{13}C NMR (150 MHz, DMSO-d$_6$)

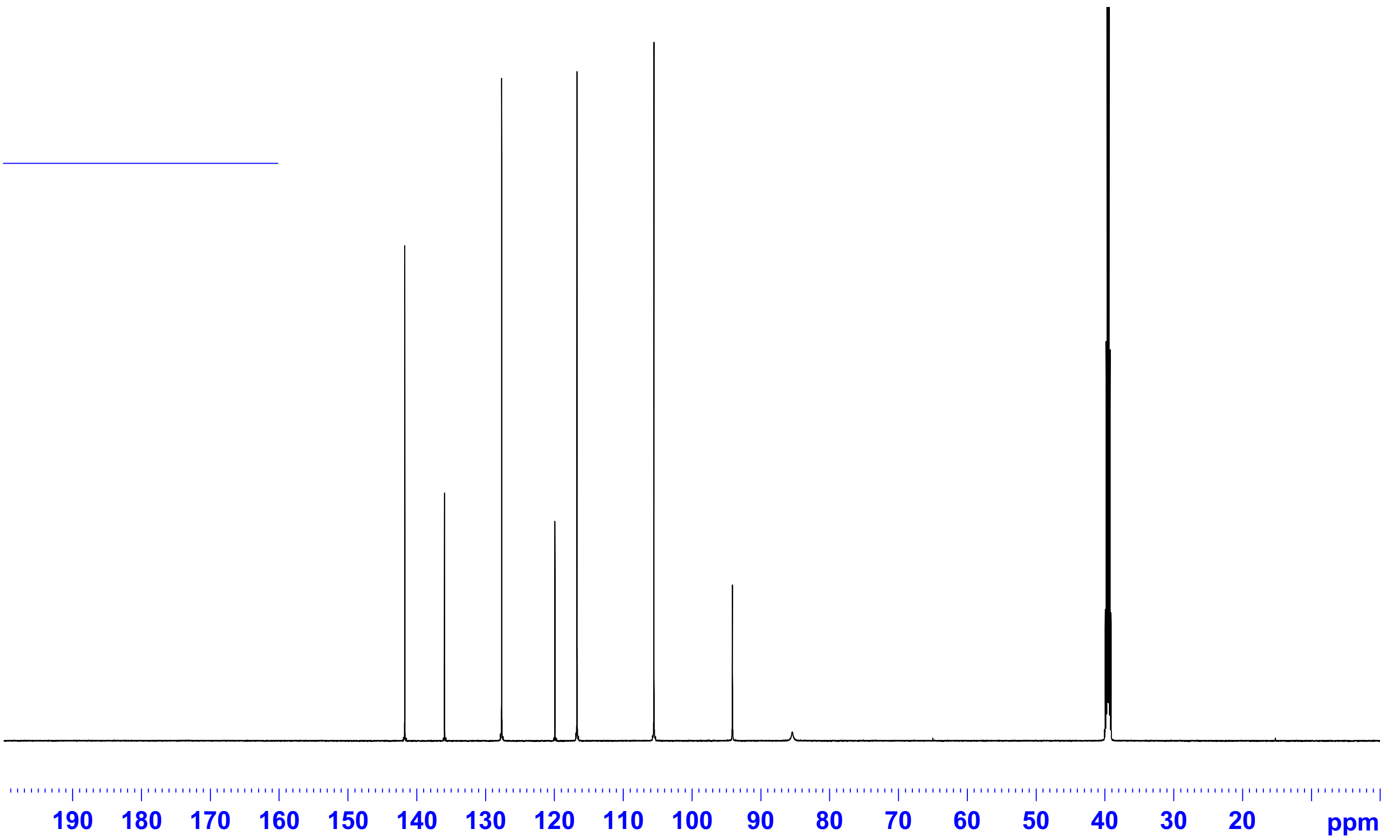

2-Benzyl-5-butyl-7-(trimethylsilyl)isoindolin-1-one 10b

^{1}H NMR (600 MHz, DMSO-d$_6$)

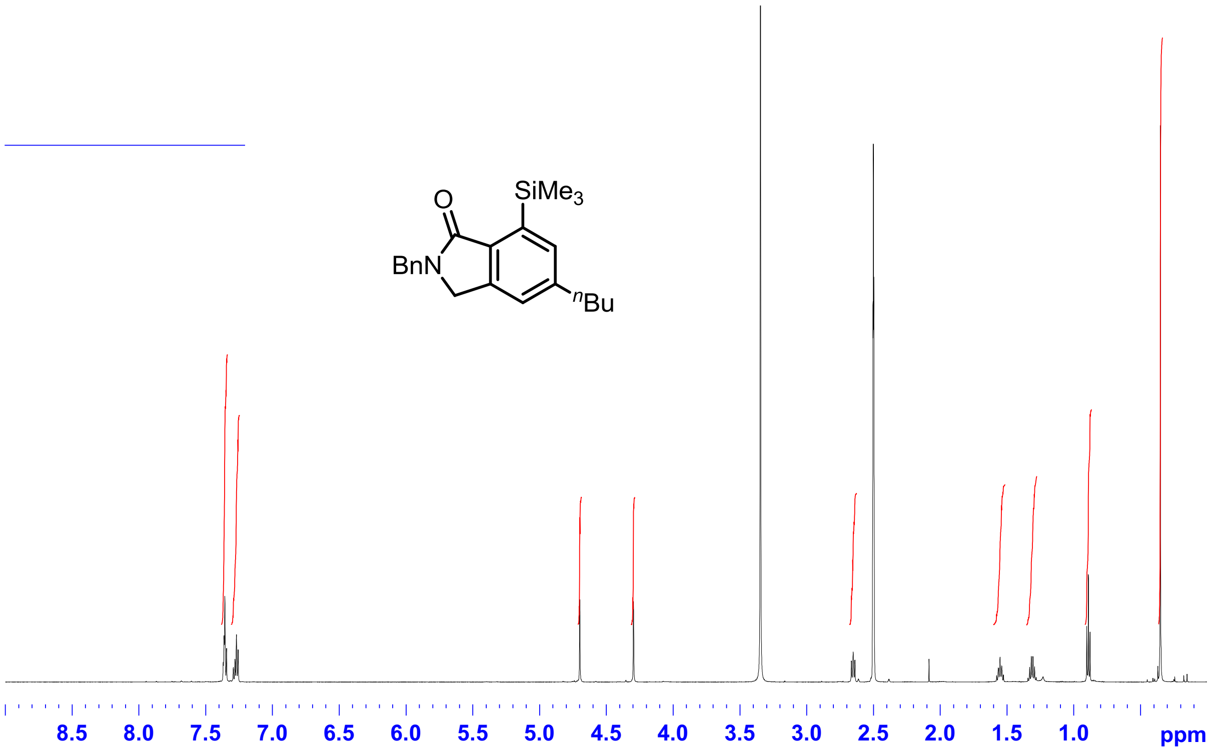

^{13}C NMR (150 MHz, DMSO-d$_6$)

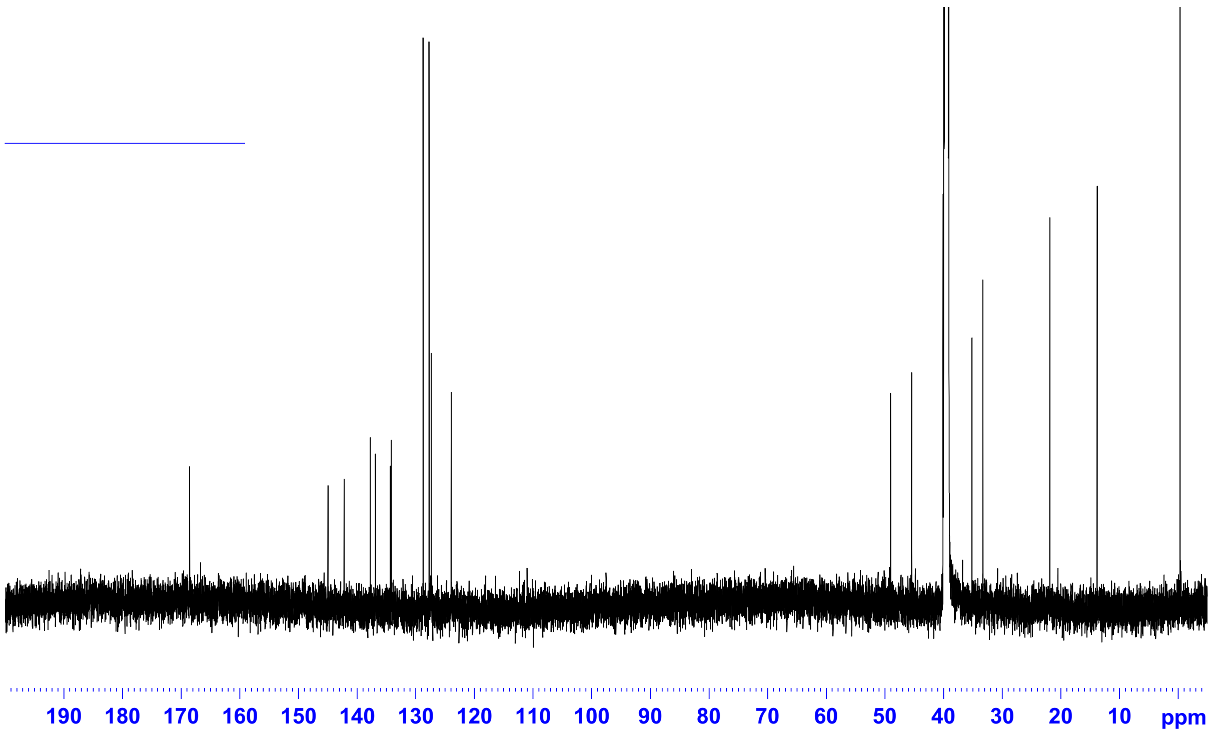

2-Benzyl-5-(*tert*-butyl)-7-(trimethylsilyl)isoindolin-1-one 10b

^{1}H NMR (600 MHz, DMSO-d$_6$)

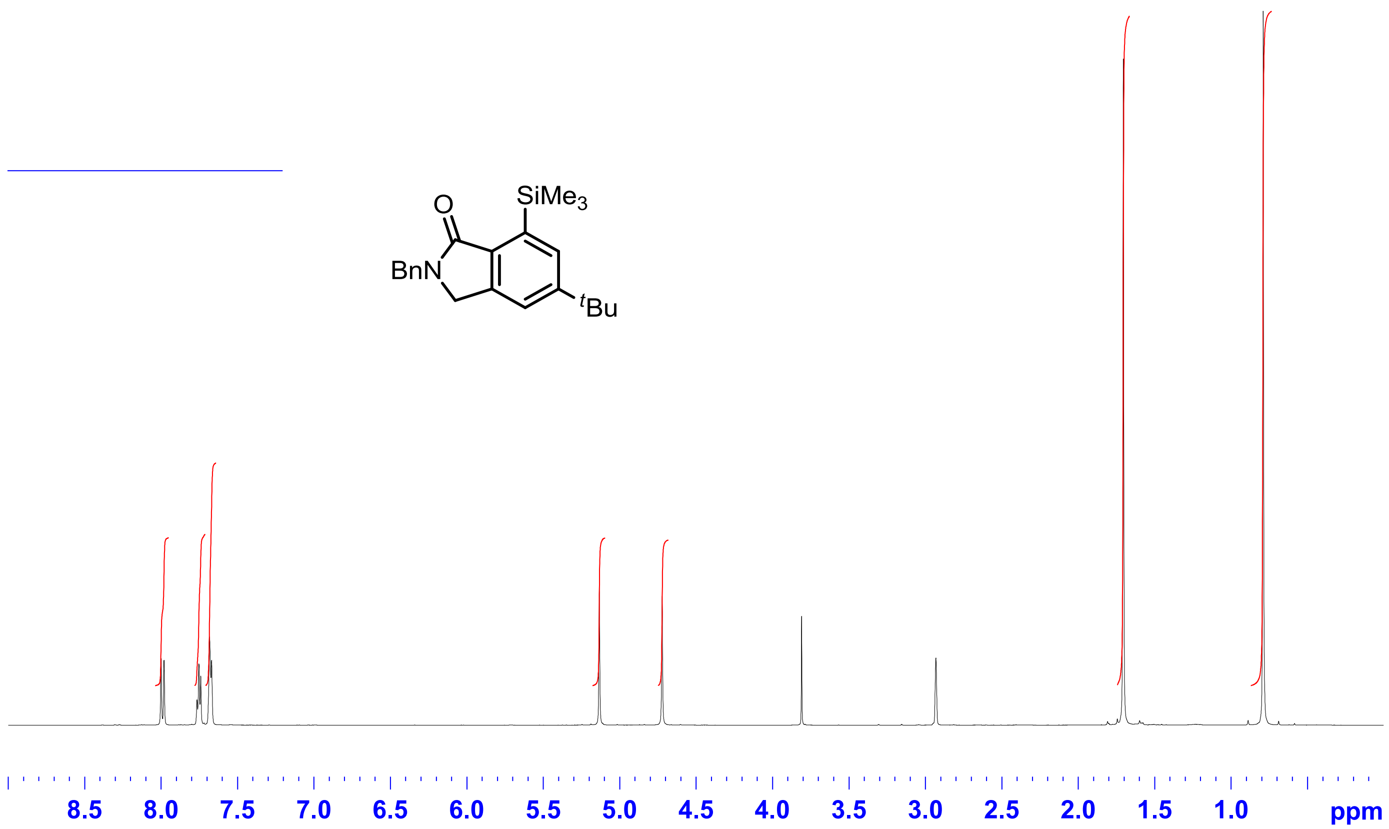

^{13}C NMR (150 MHz, DMSO-d$_6$)

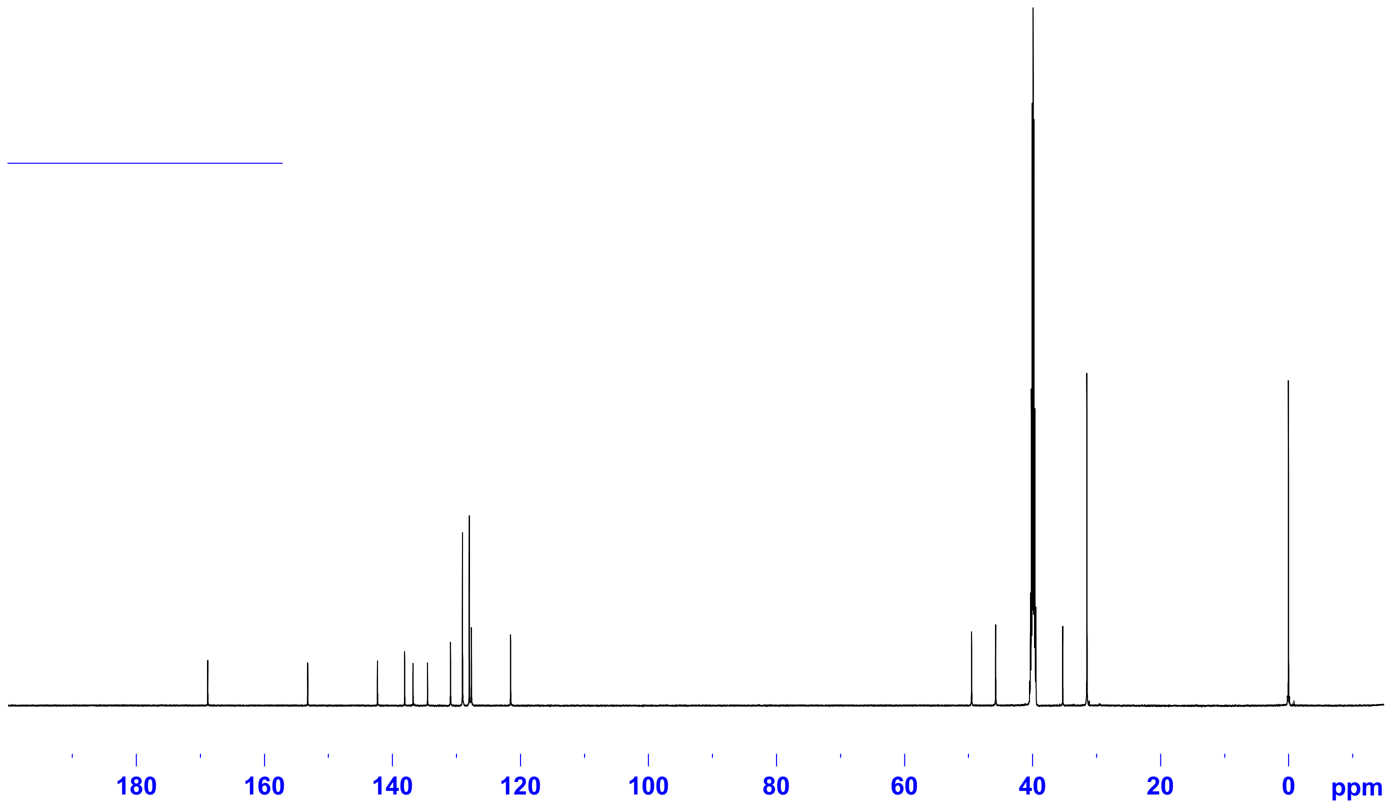

2-Benzyl-5-cyclopropyl-7-(trimethylsilyl)isoindolin-1-one 10c

^{1}H NMR (600 MHz, DMSO-d$_6$)

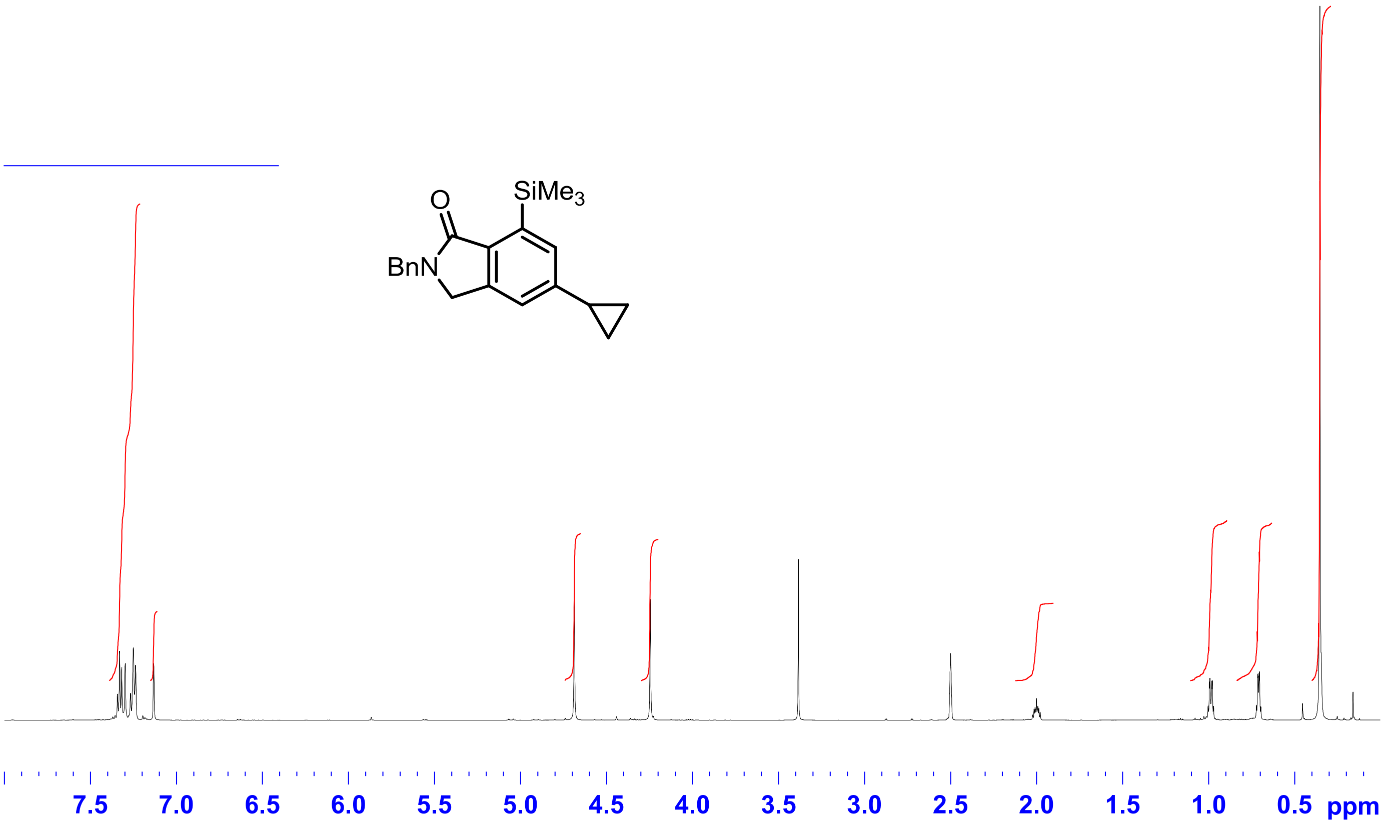

^{13}C NMR (150 MHz, DMSO-d$_6$)

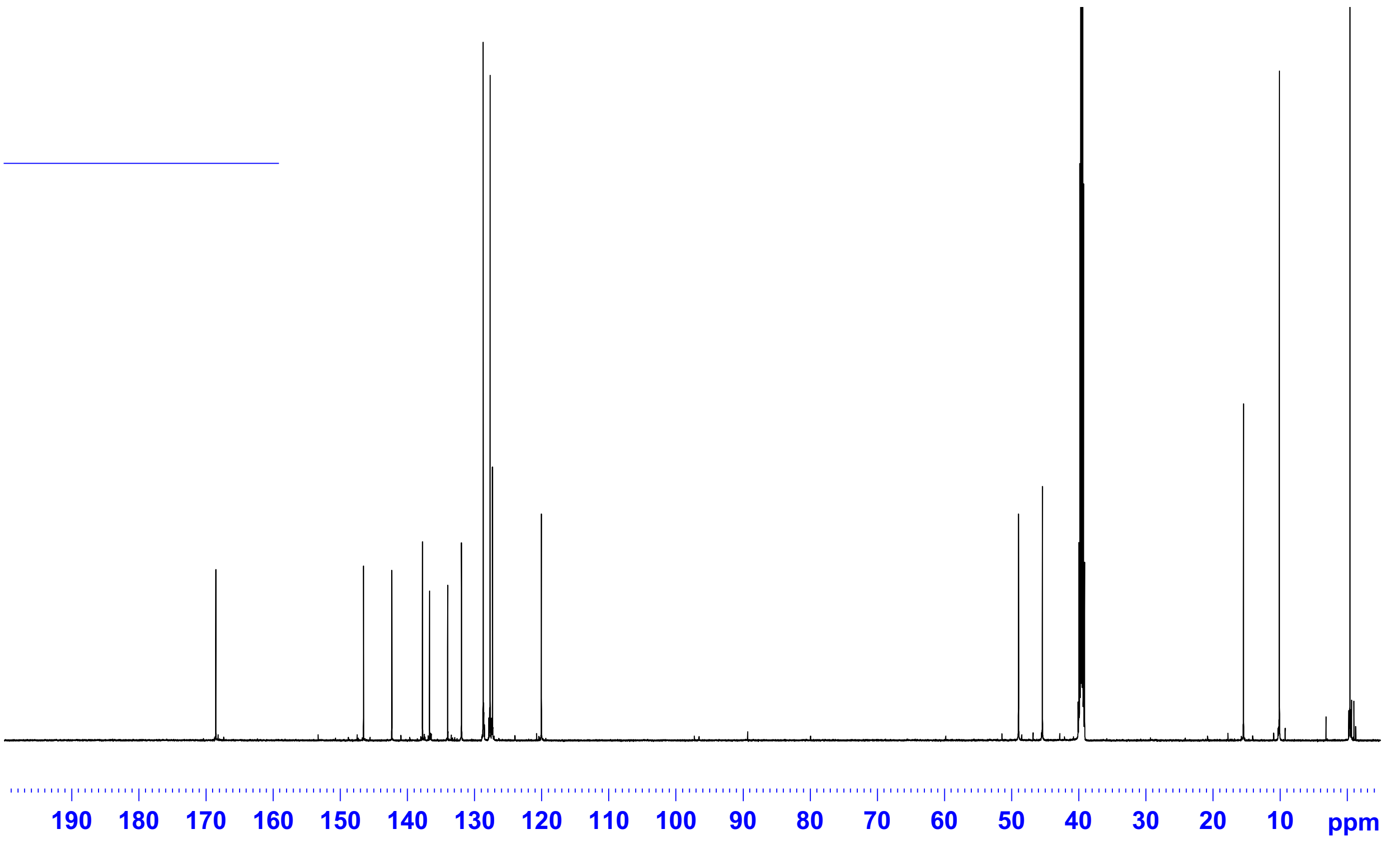

2-Benzyl-5-cyclopentyl-7-(trimethylsilyl)isoindolin-1-one 10d

^{1}H NMR (600 MHz, DMSO-d_6)

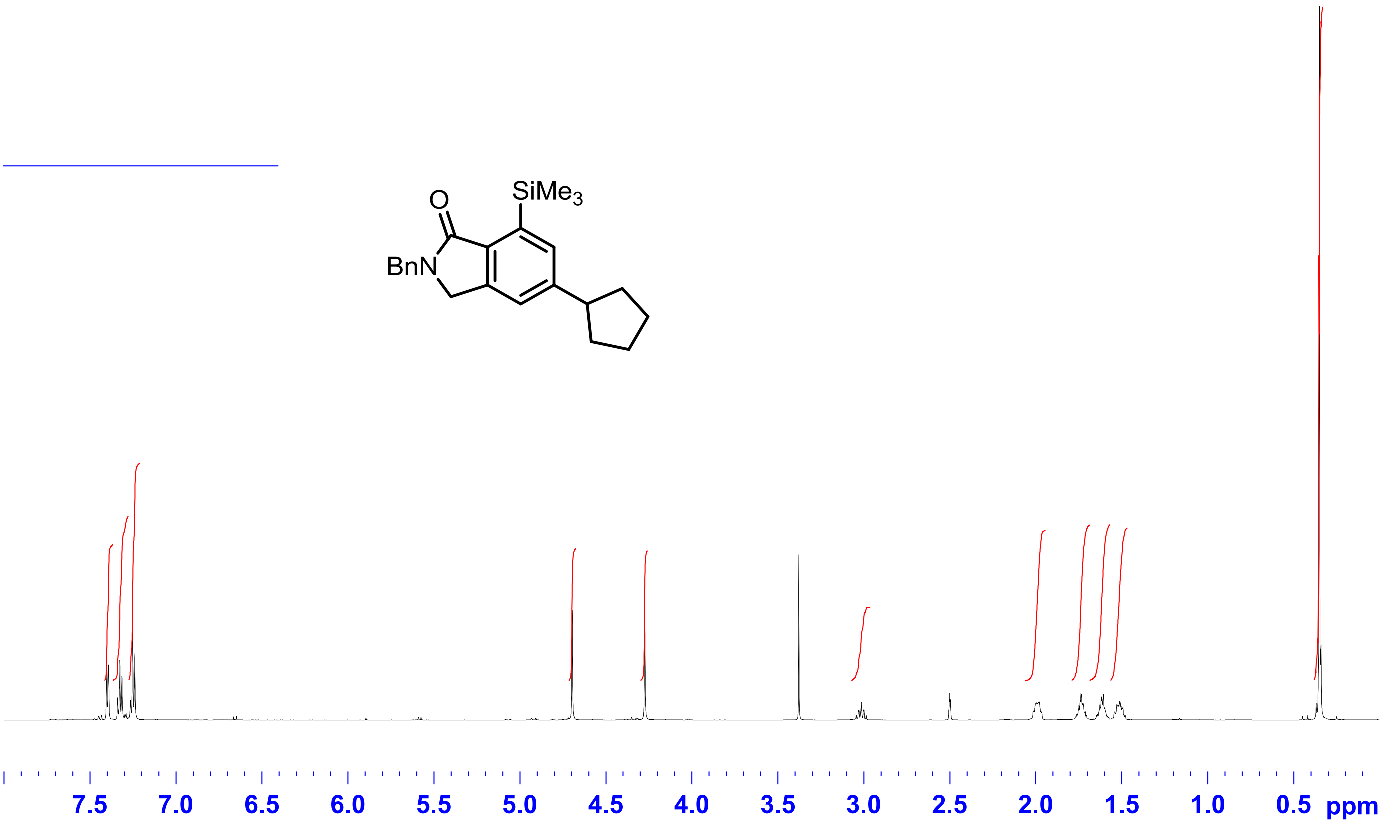

^{13}C NMR (150 MHz, DMSO-d_6)

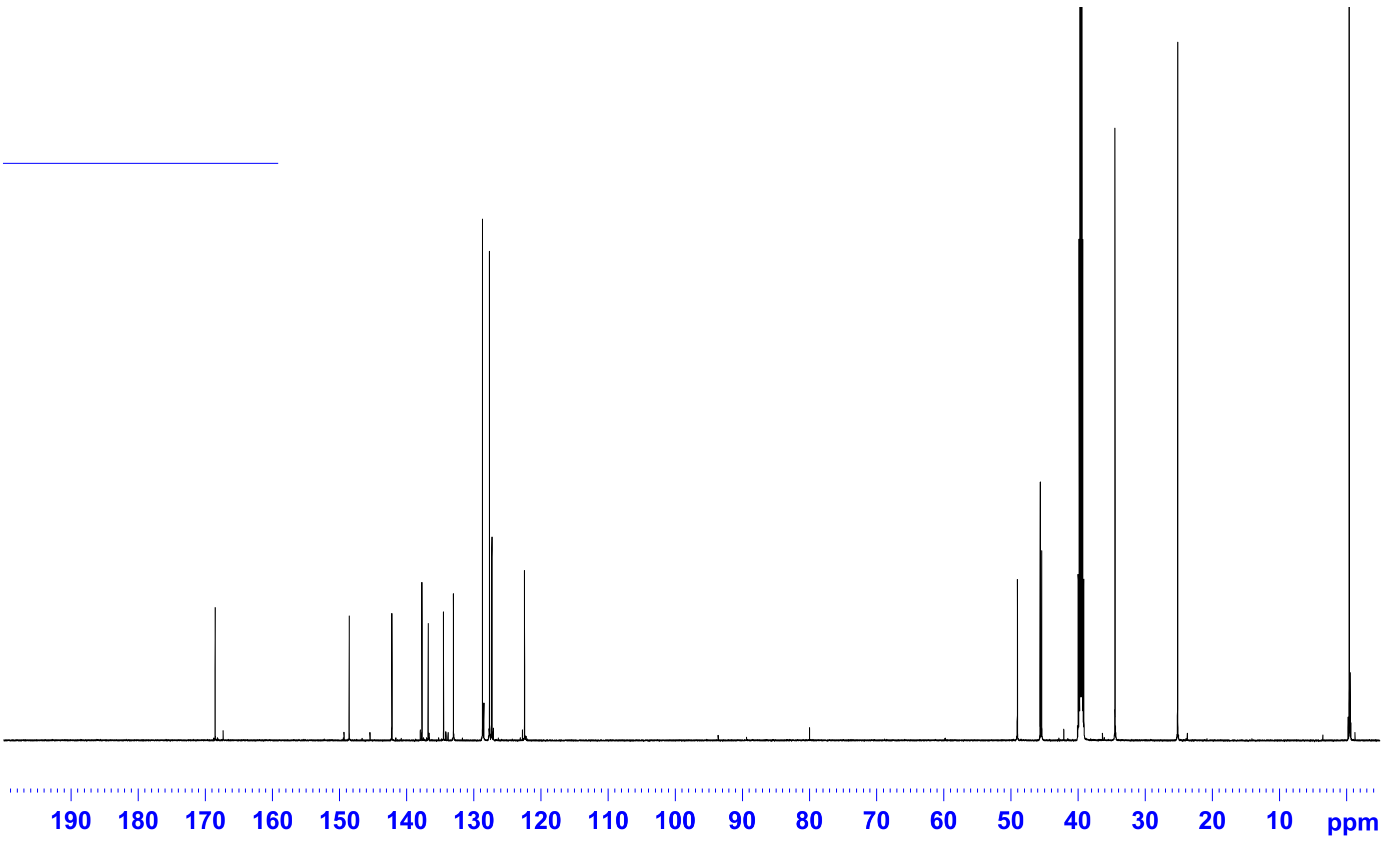

2-Benzyl-5-(3-chloropropyl)-7-(trimethylsilyl)isoindolin-1-one 10e

^{1}H NMR (600 MHz, DMSO-d$_6$)

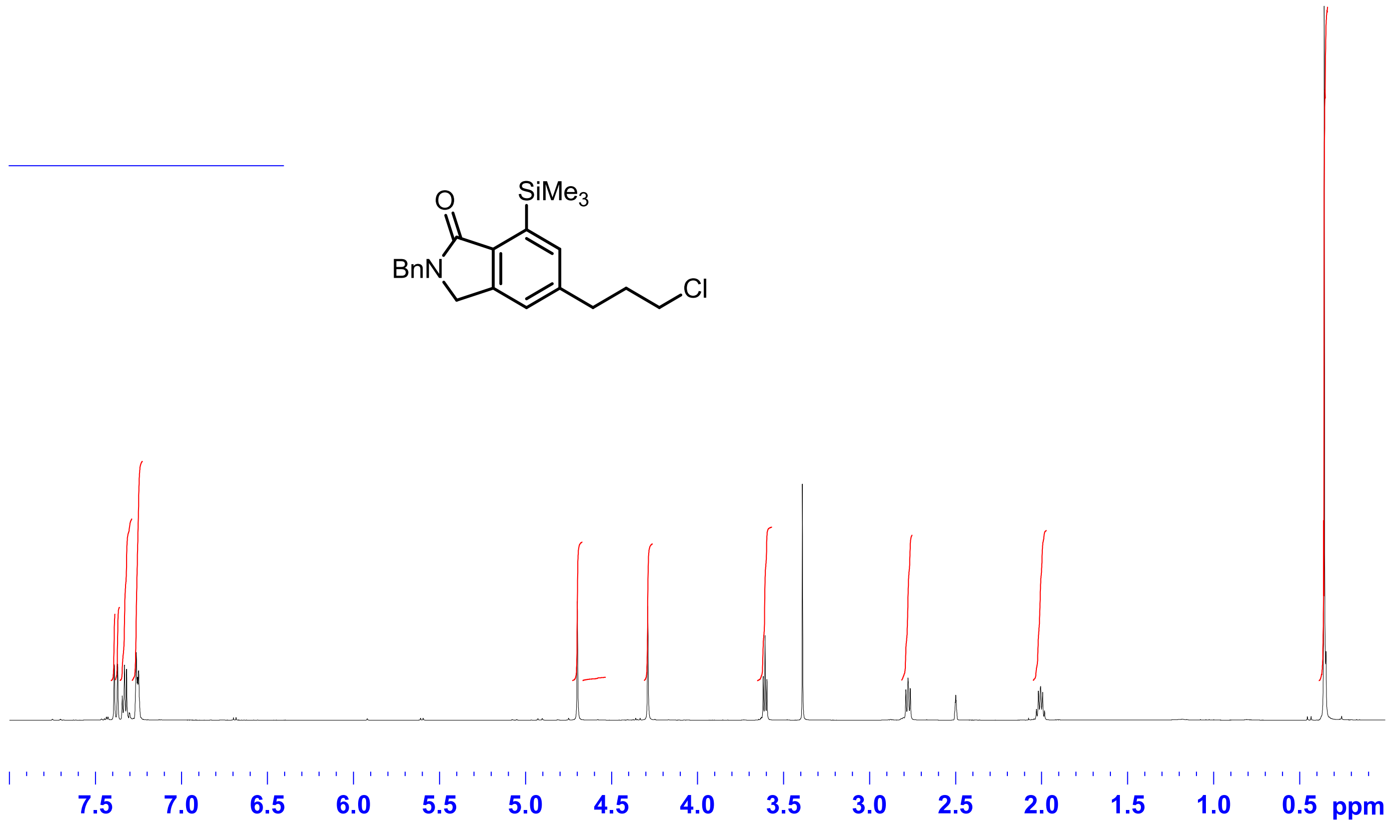

^{13}C NMR (150 MHz, DMSO-d$_6$)

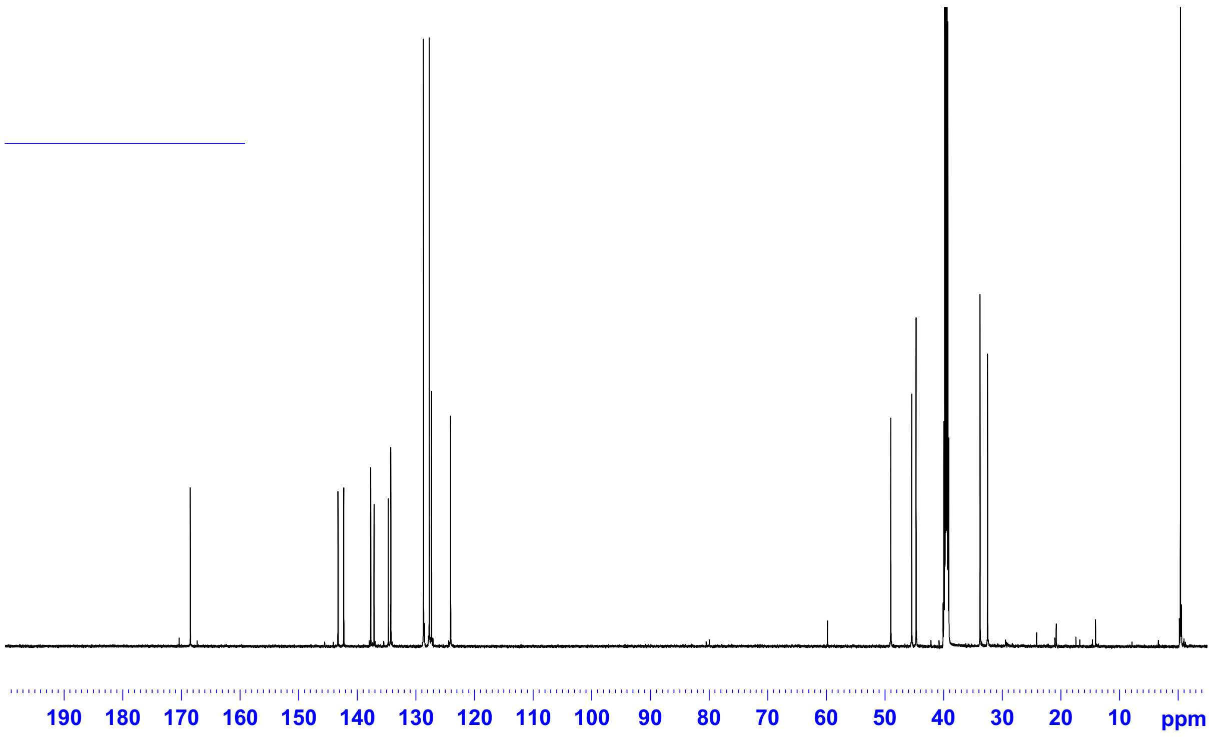

***tert*-Butyl ((2-benzyl-1-oxo-7-(trimethylsilyl)isoindolin-5-yl)methyl)carbamate 10f**

^{1}H NMR (600 MHz, DMSO-d_6)

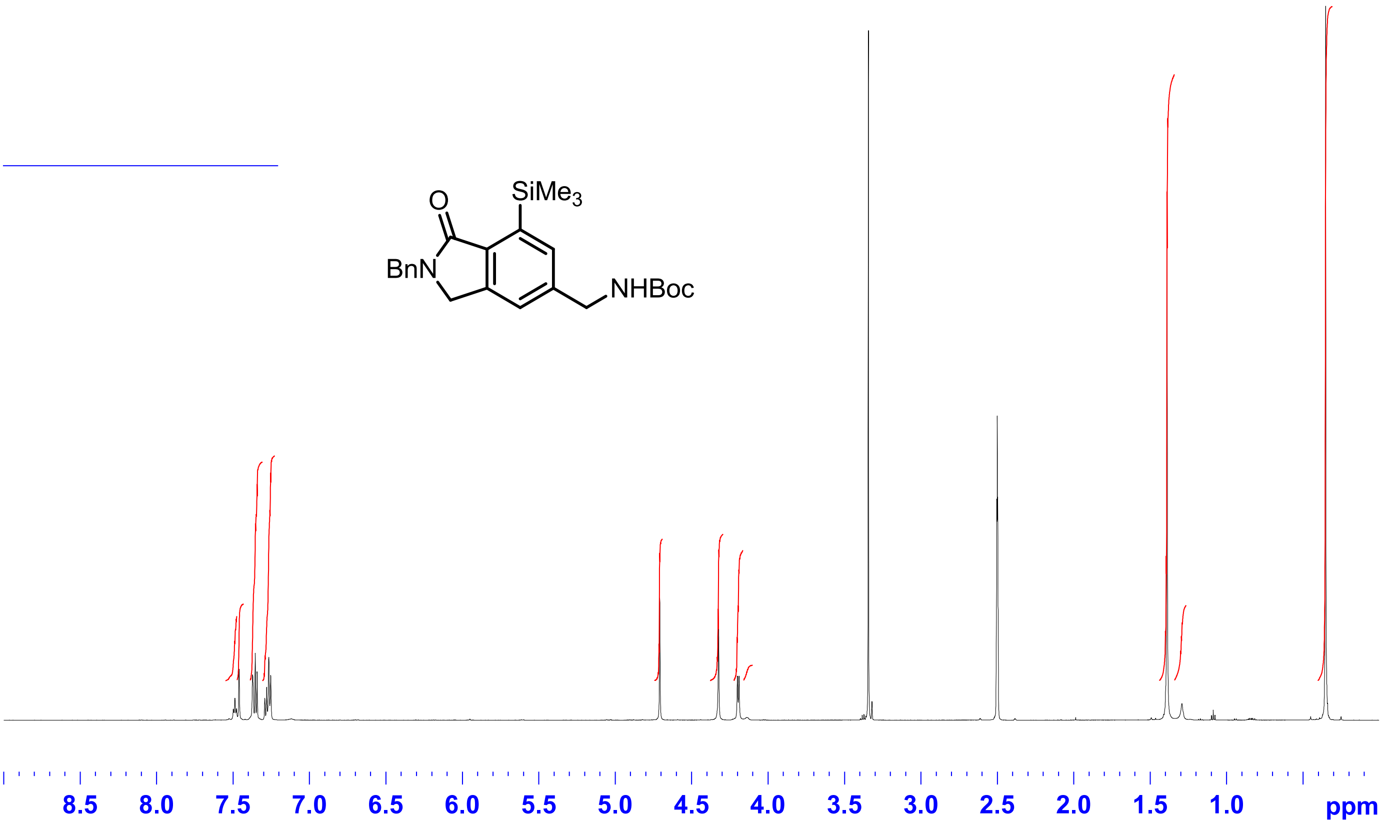

^{13}C NMR (150 MHz, DMSO-d_6)

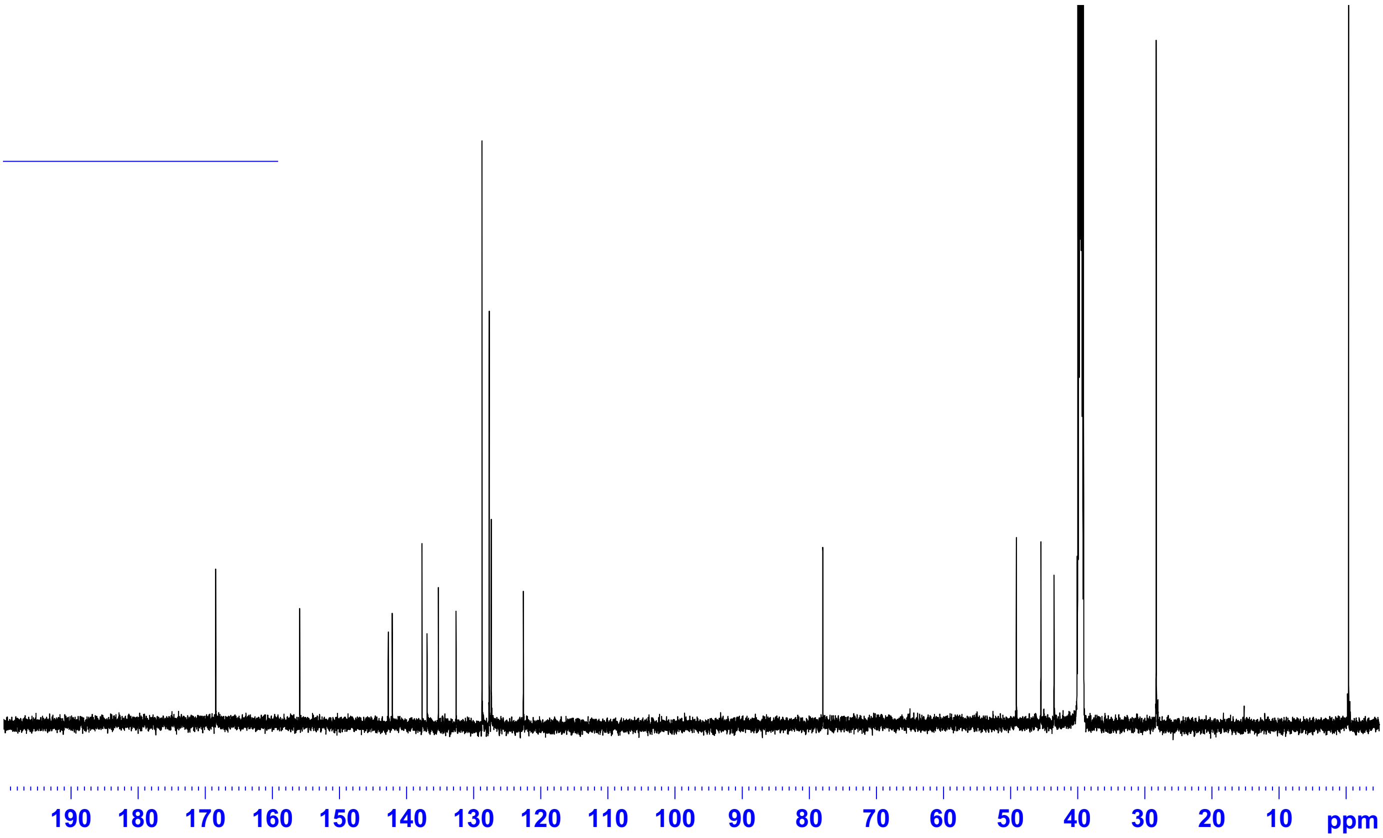

2-Benzyl-5-(methoxymethyl)-7-(trimethylsilyl)isoindolin-1-one 10g

^{1}H NMR (600 MHz, DMSO-d_6)

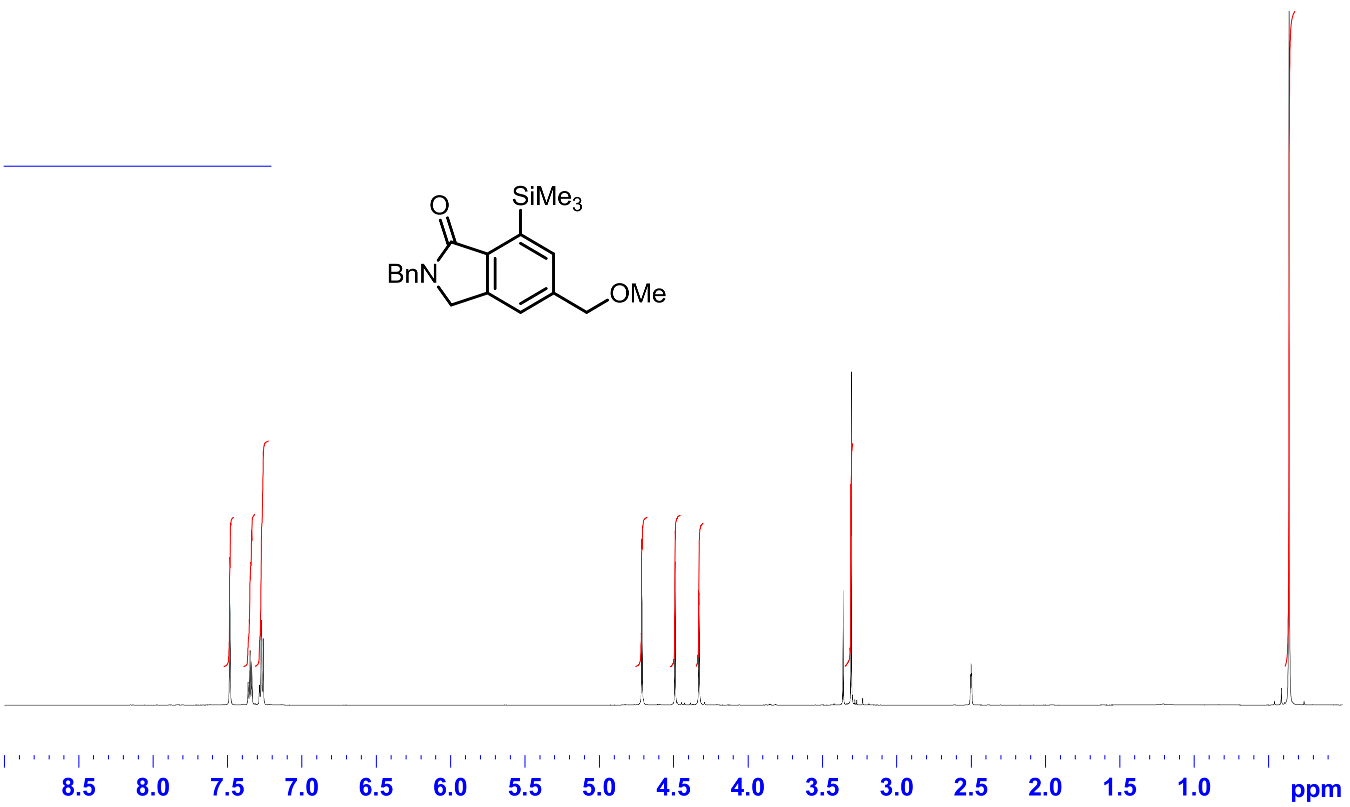

^{13}C NMR (150 MHz, DMSO-d_6)

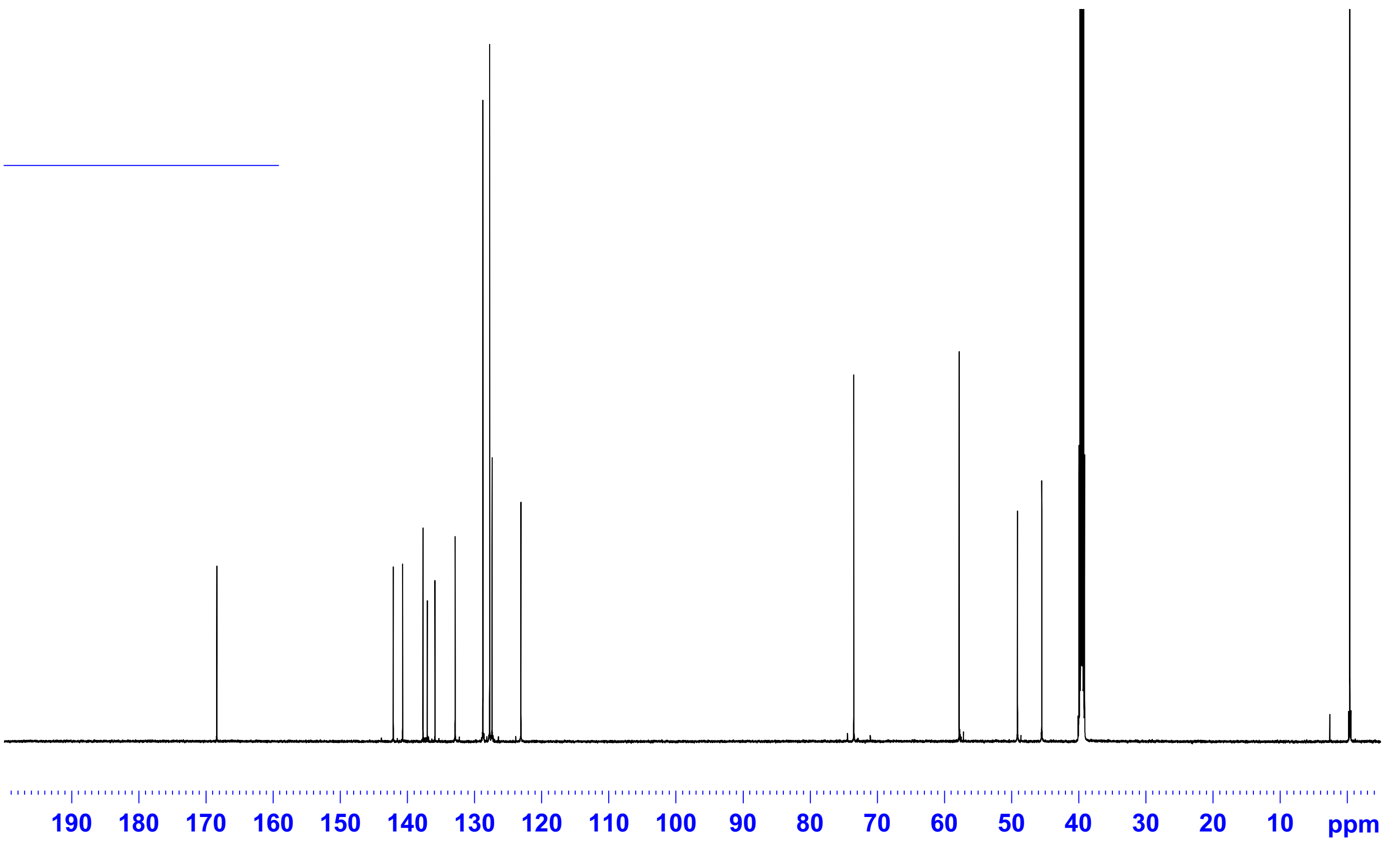

2-Benzyl-5-(diethoxymethyl)-7-(trimethylsilyl)isoindolin-1-one 10h

^{1}H NMR (600 MHz, DMSO-d$_6$)

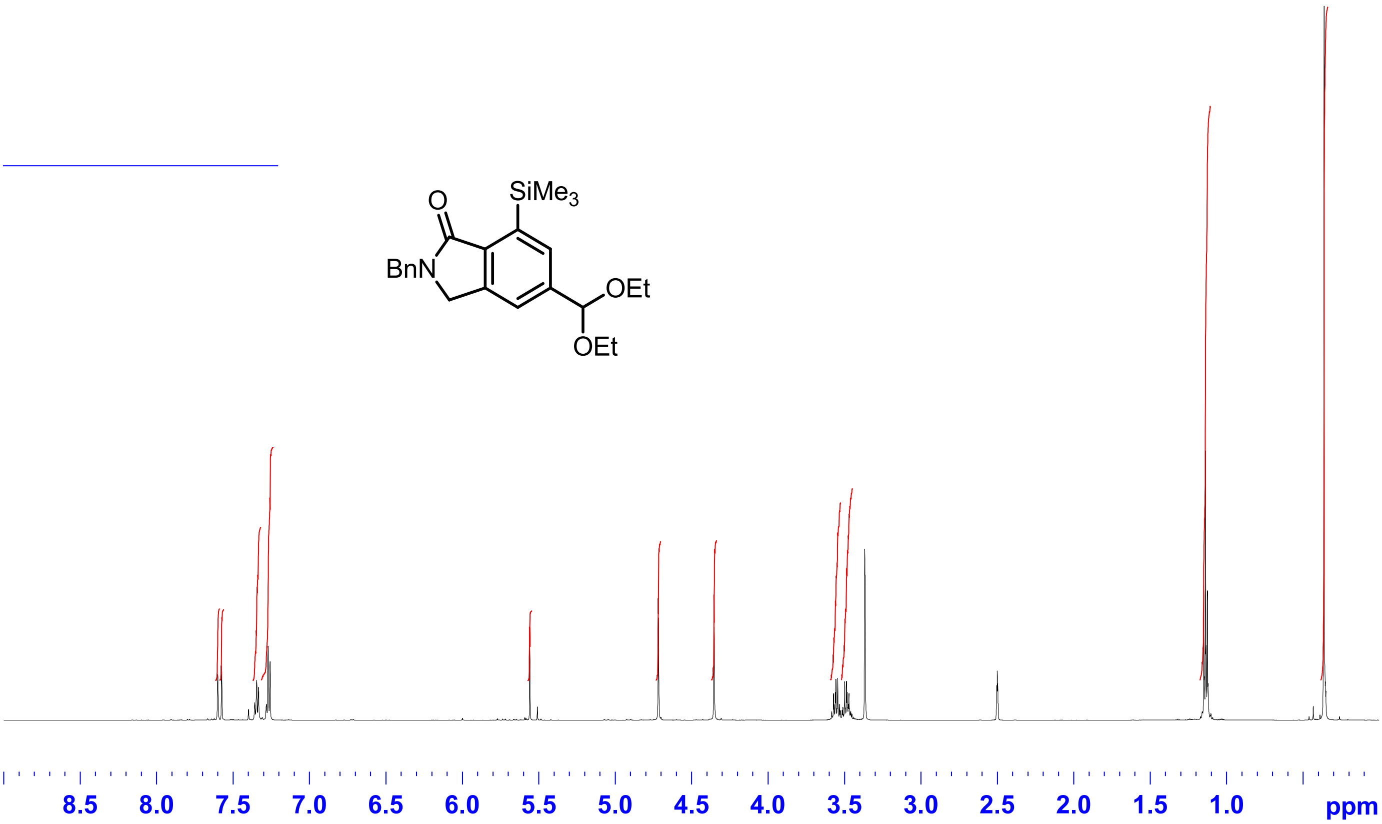

^{13}C NMR (150 MHz, DMSO-d$_6$)

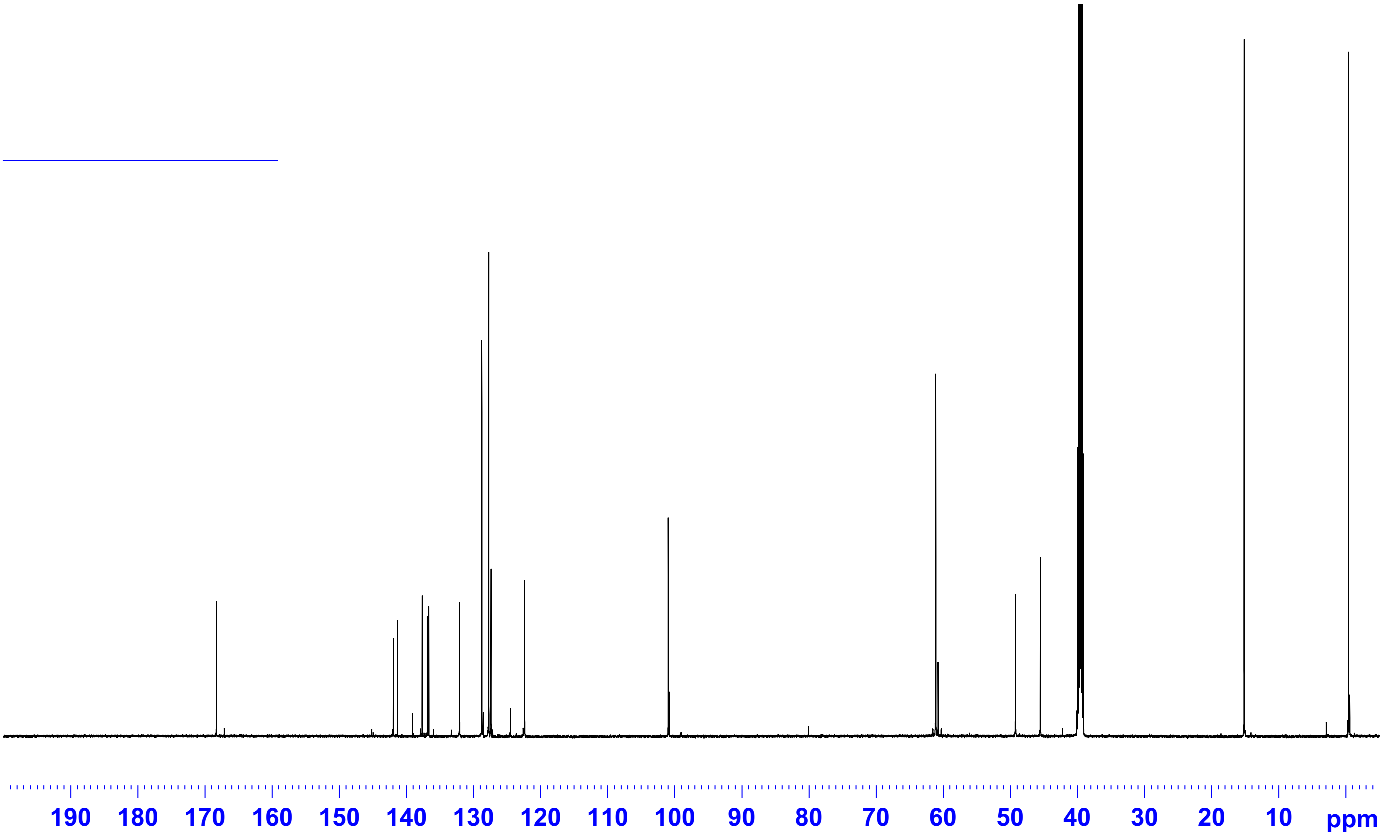

2-Benzyl-5-phenyl-7-(trimethylsilyl)isoindolin-1-one 10k

^{1}H NMR (600 MHz, DMSO-d_6)

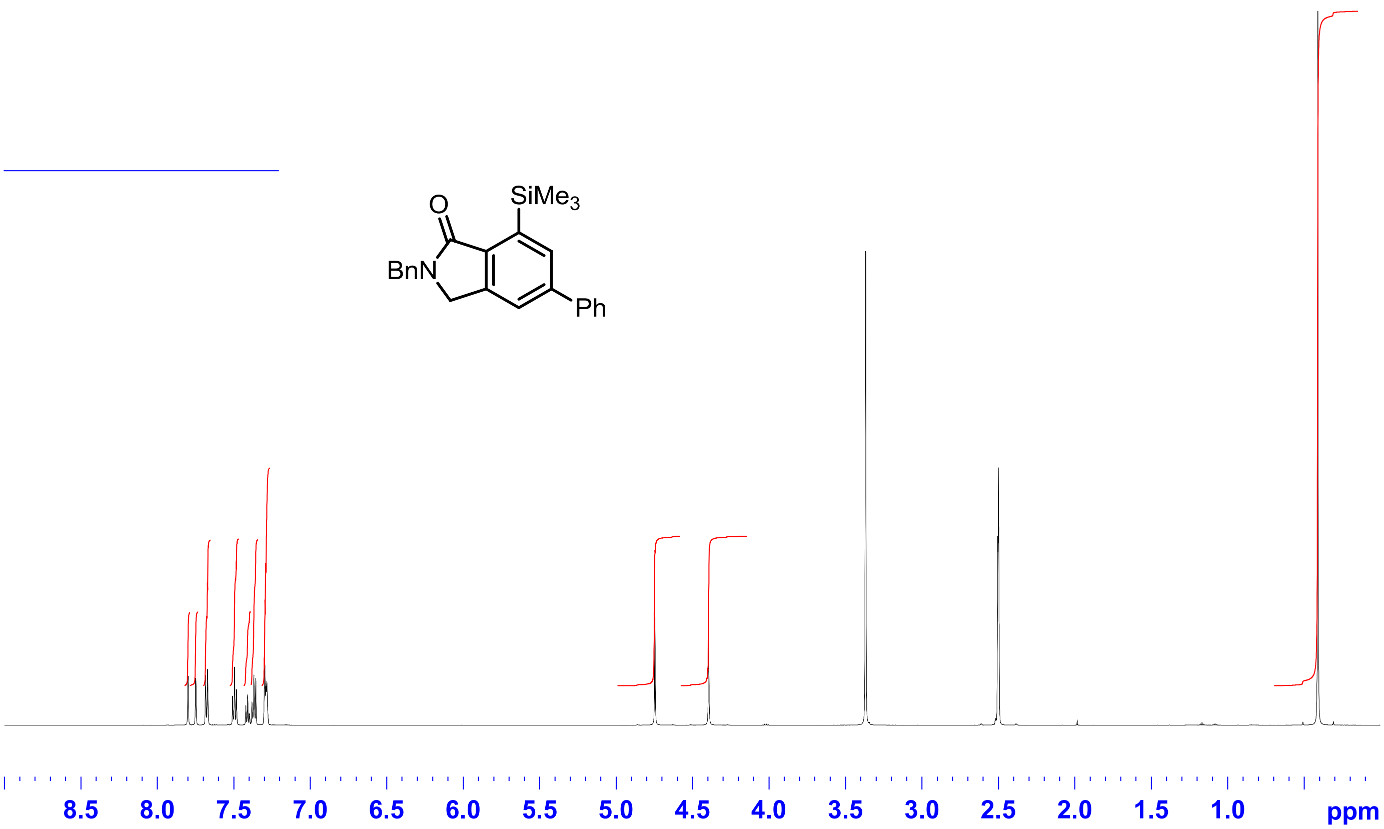

^{13}C NMR (150 MHz, DMSO-d_6)

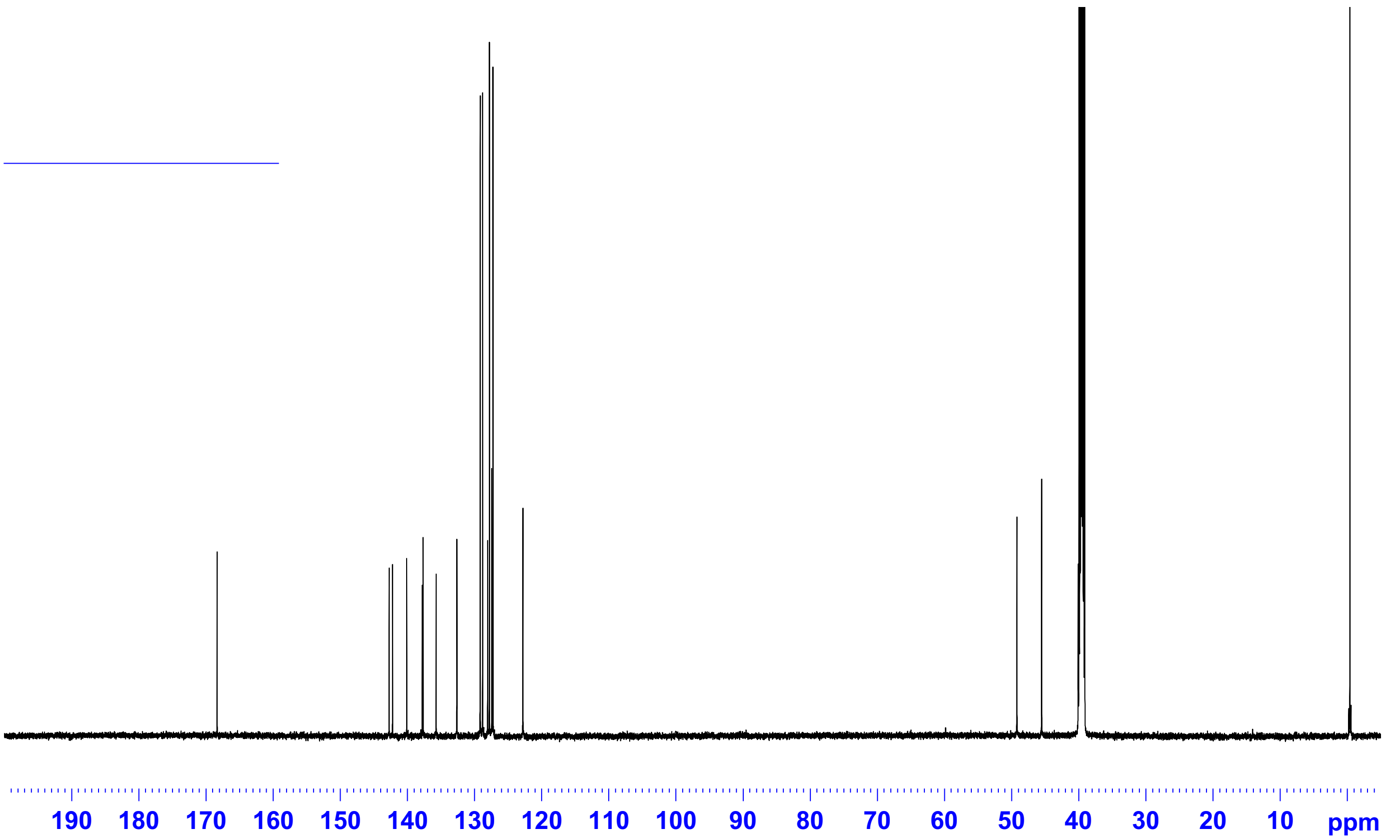

2-Benzyl-5-(*o*-tolyl)-7-(trimethylsilyl)isoindolin-1-one 10l

^{1}H NMR (600 MHz, DMSO-d$_6$)

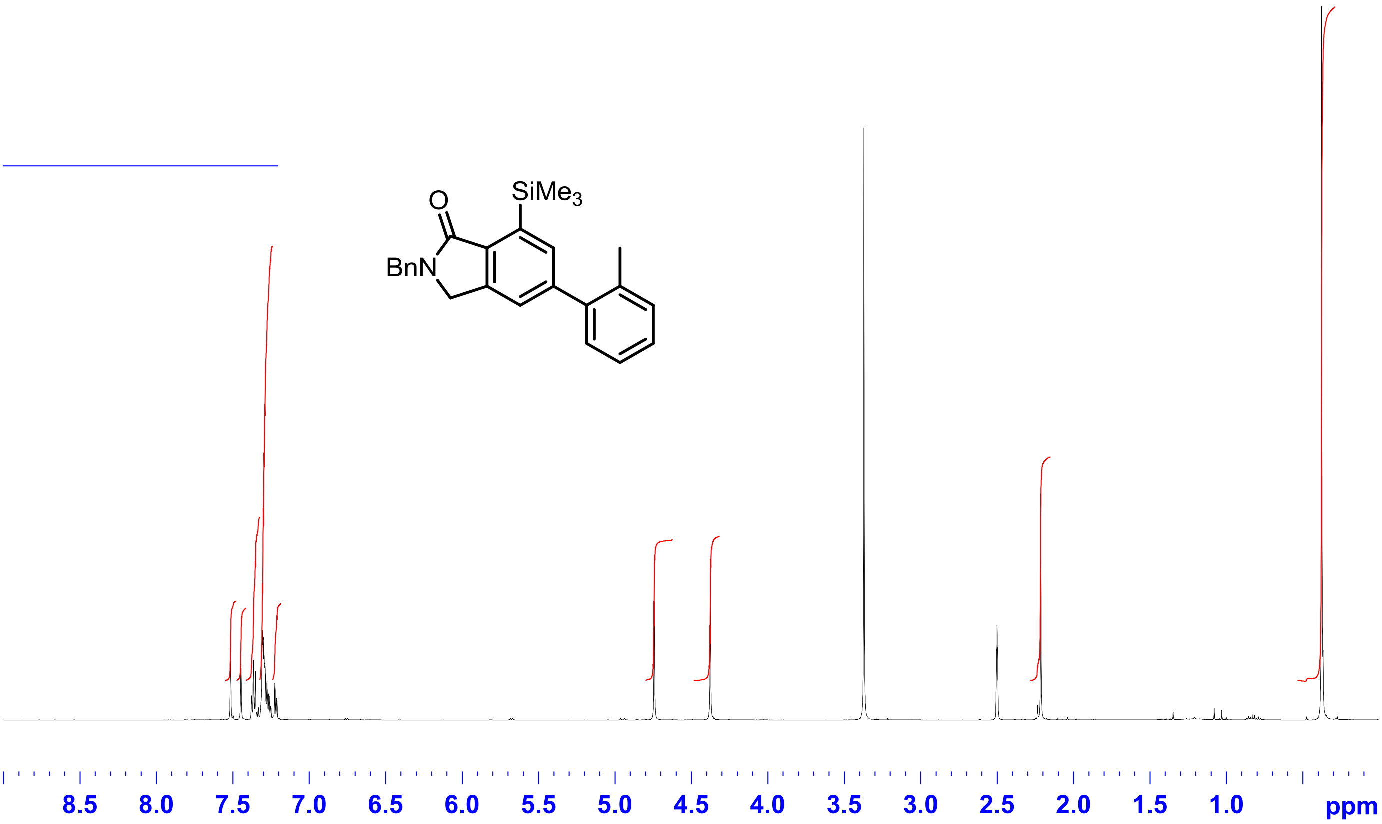

^{13}C NMR (150 MHz, DMSO-d$_6$)

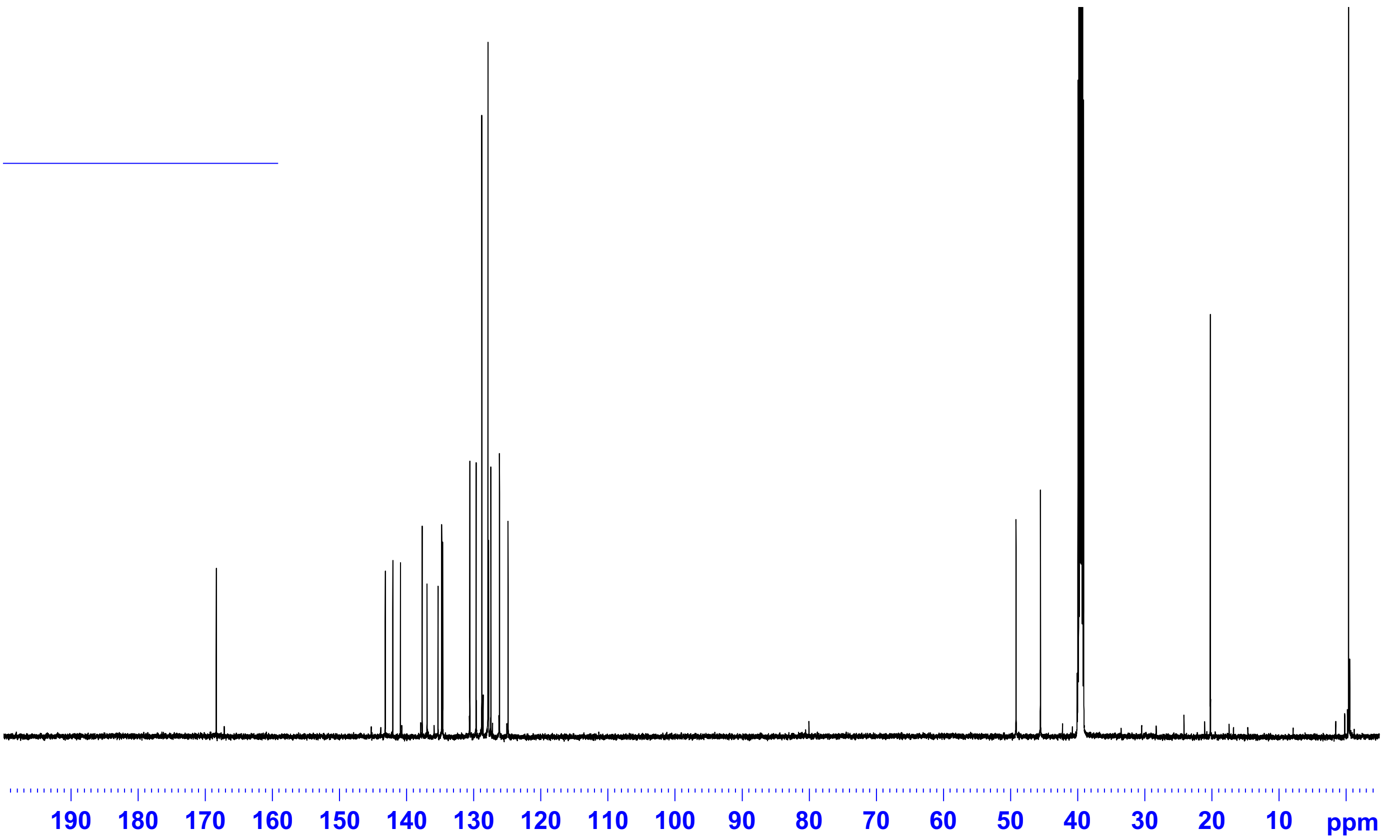

2-Benzyl-5-(*p*-tolyl)-7-(trimethylsilyl)isoindolin-1-one 10m

^{1}H NMR (600 MHz, DMSO-d_6)

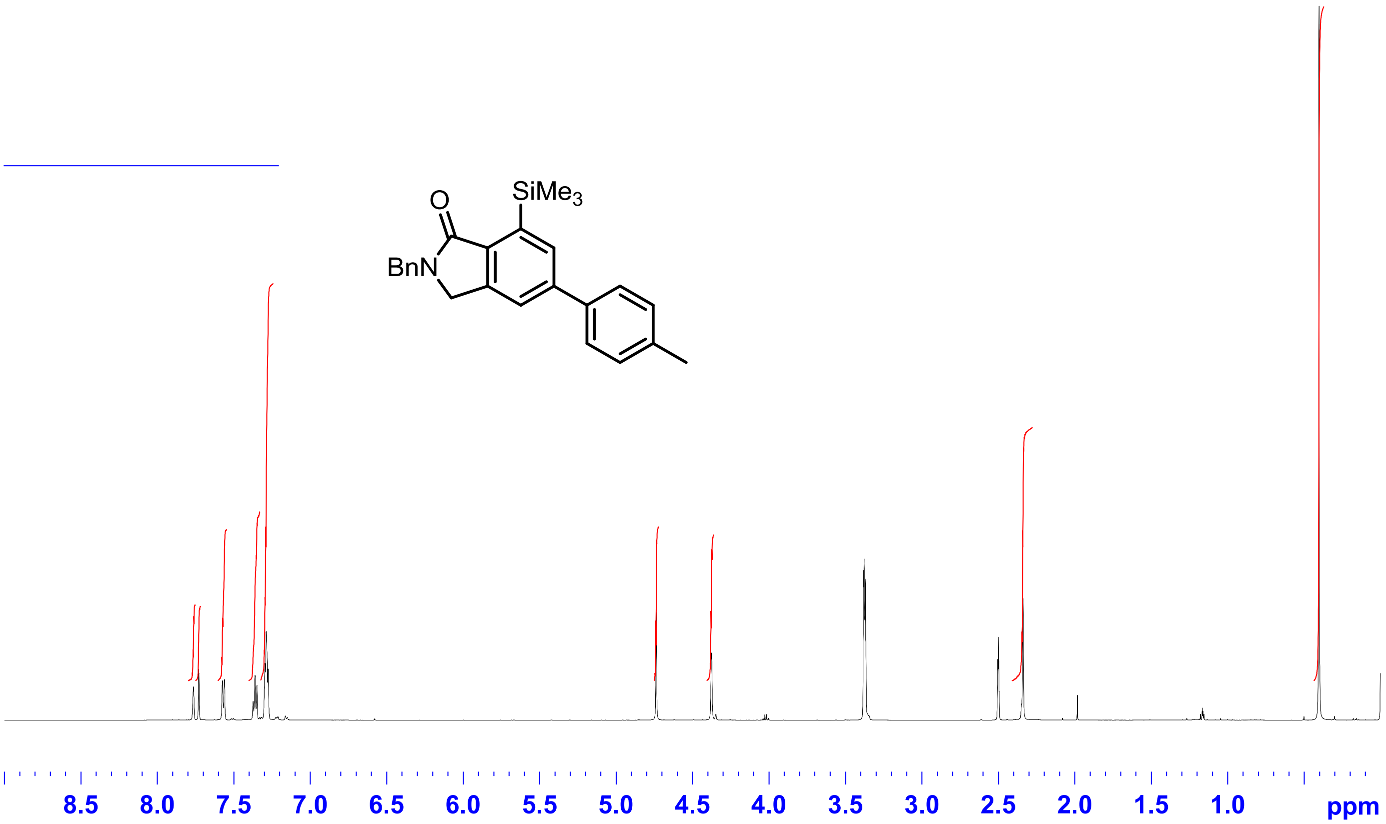

^{13}C NMR (150 MHz, DMSO-d_6)

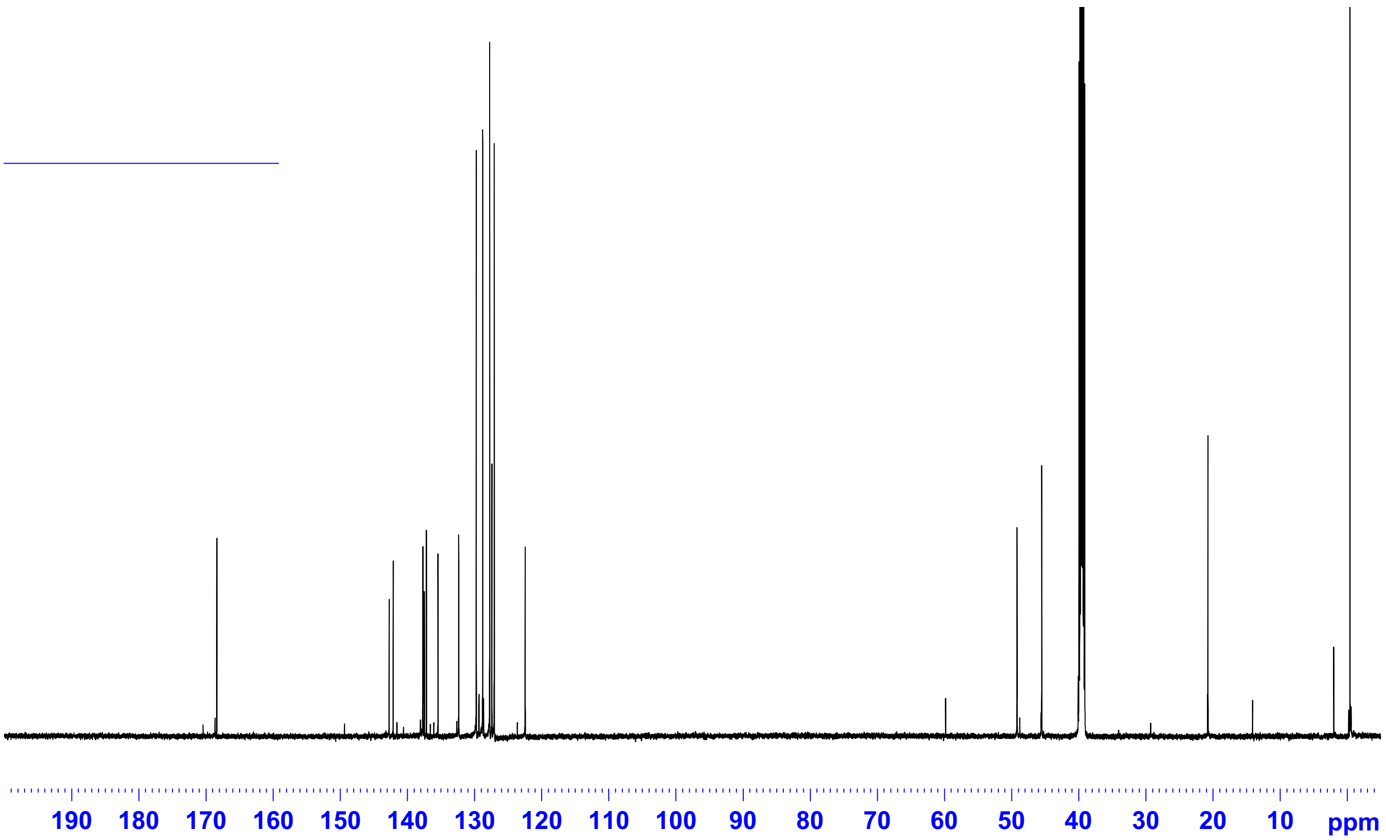

2-Benzyl-5-(2-bromophenyl)-7-(trimethylsilyl)isoindolin-1-one 10n

^{1}H NMR (600 MHz, DMSO-d$_6$)

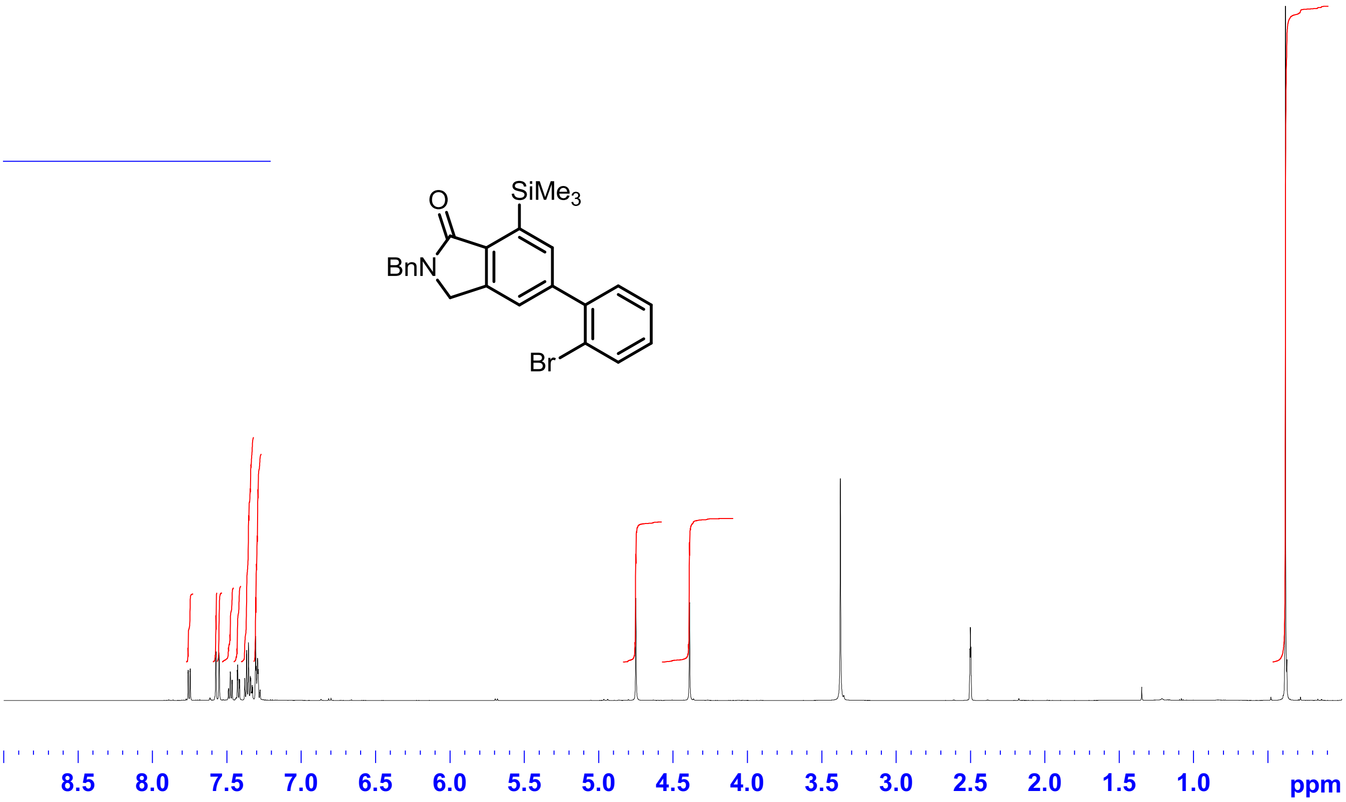

^{13}C NMR (150 MHz, DMSO-d$_6$)

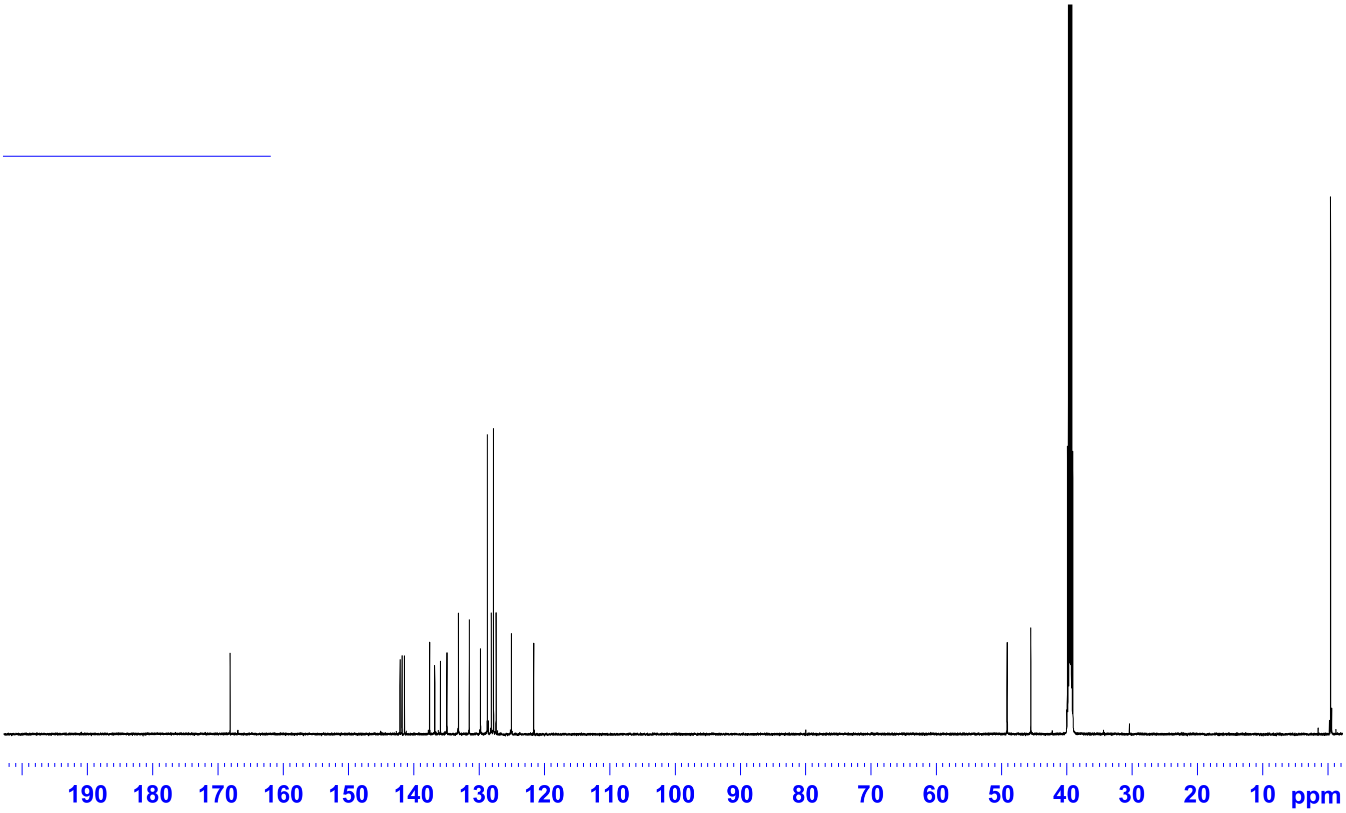

2-Benzyl-5-(4-bromophenyl)-7-(trimethylsilyl)isoindolin-1-one 10o

^{1}H NMR (600 MHz, DMSO-d$_6$)

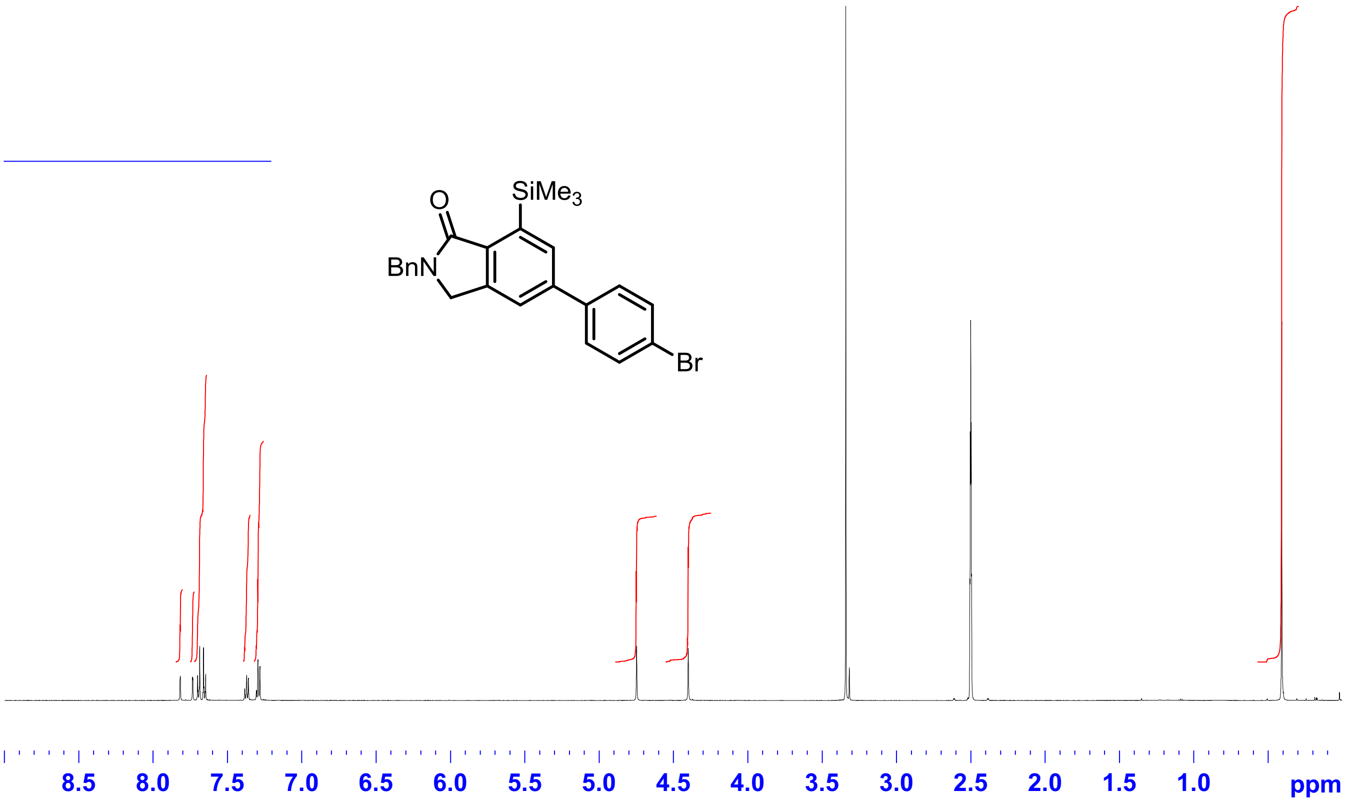

^{13}C NMR (150 MHz, DMSO-d$_6$)

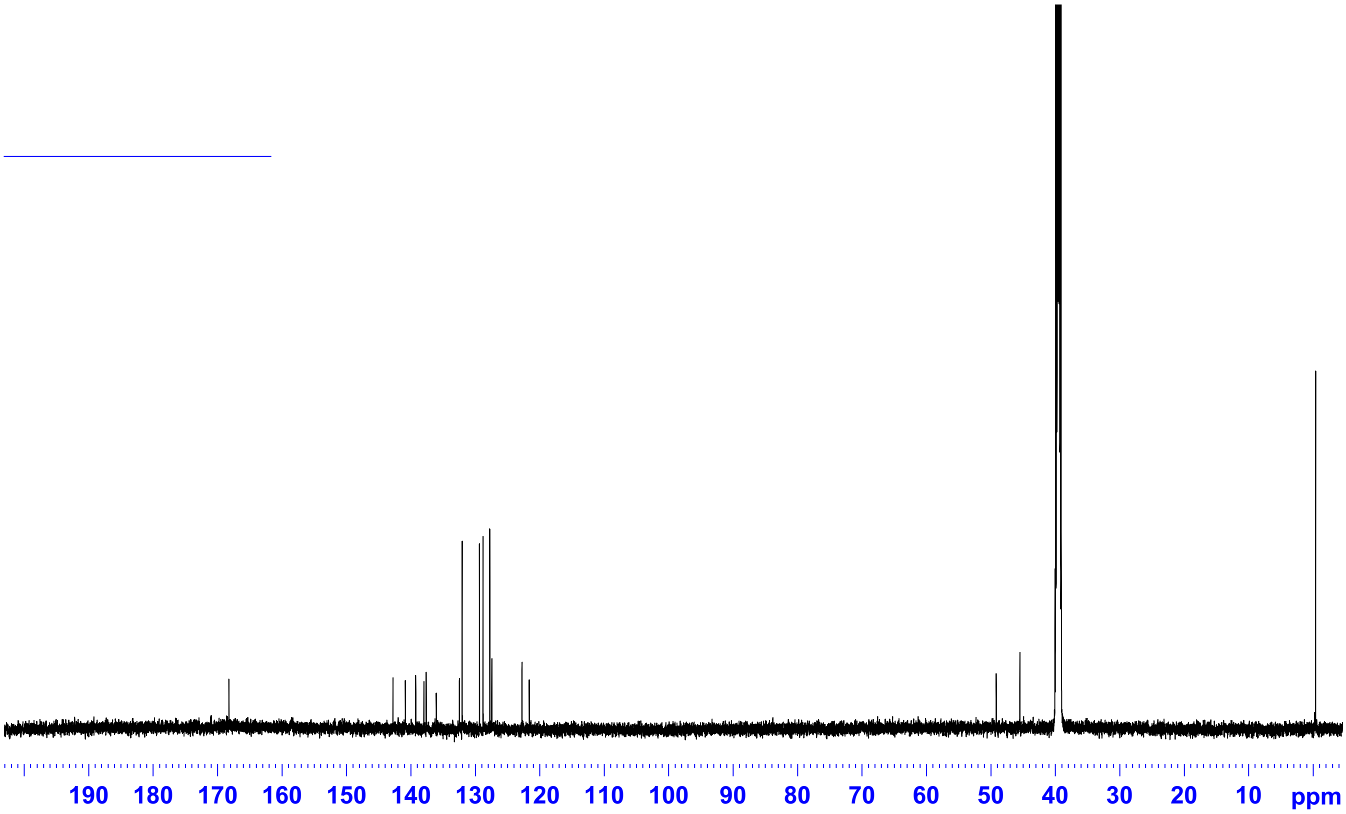

Methyl 4-(2-benzyl-1-oxo-7-(trimethylsilyl)isoindolin-5-yl)benzoate 10p

^{1}H NMR (600 MHz, DMSO-d$_6$)

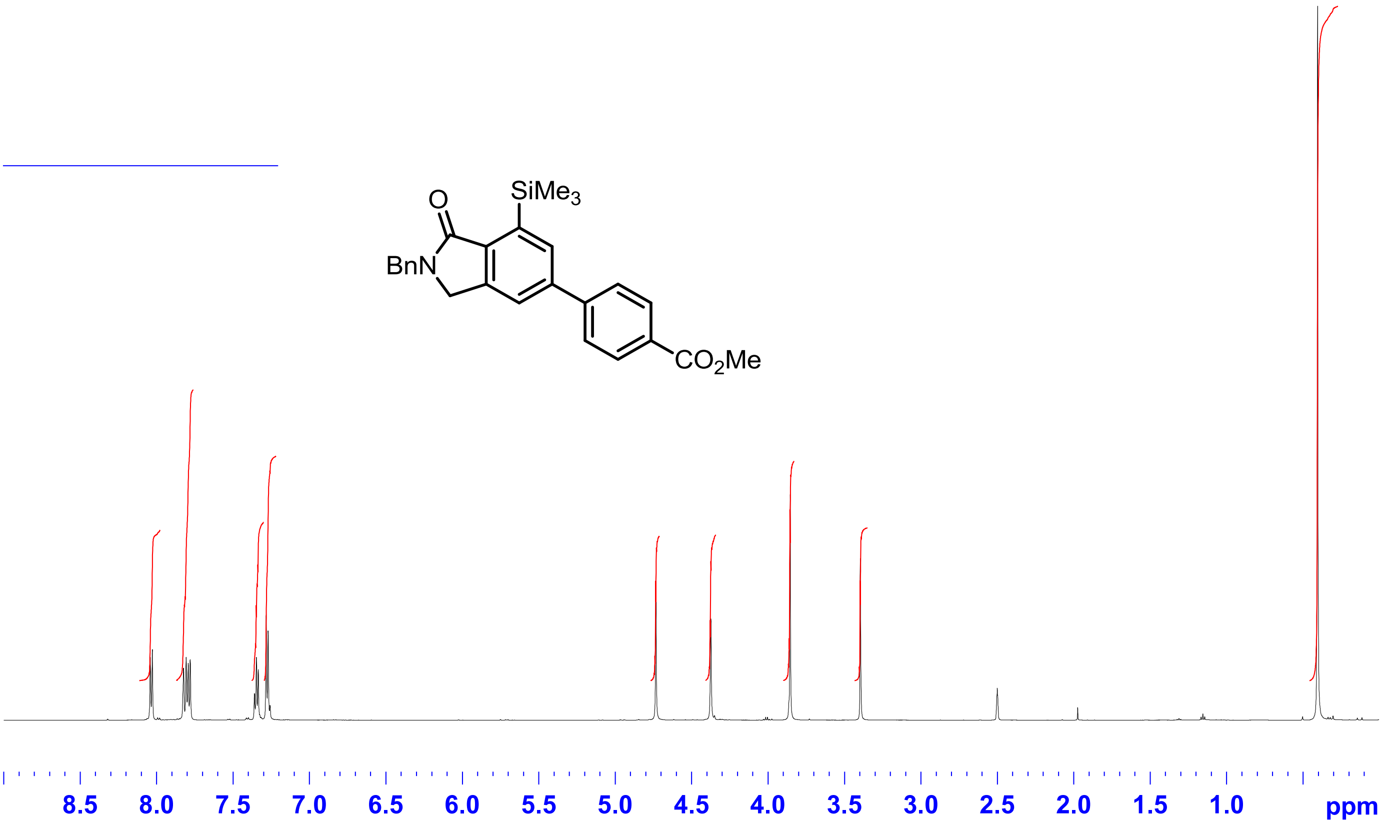

^{13}C NMR (150 MHz, DMSO-d$_6$)

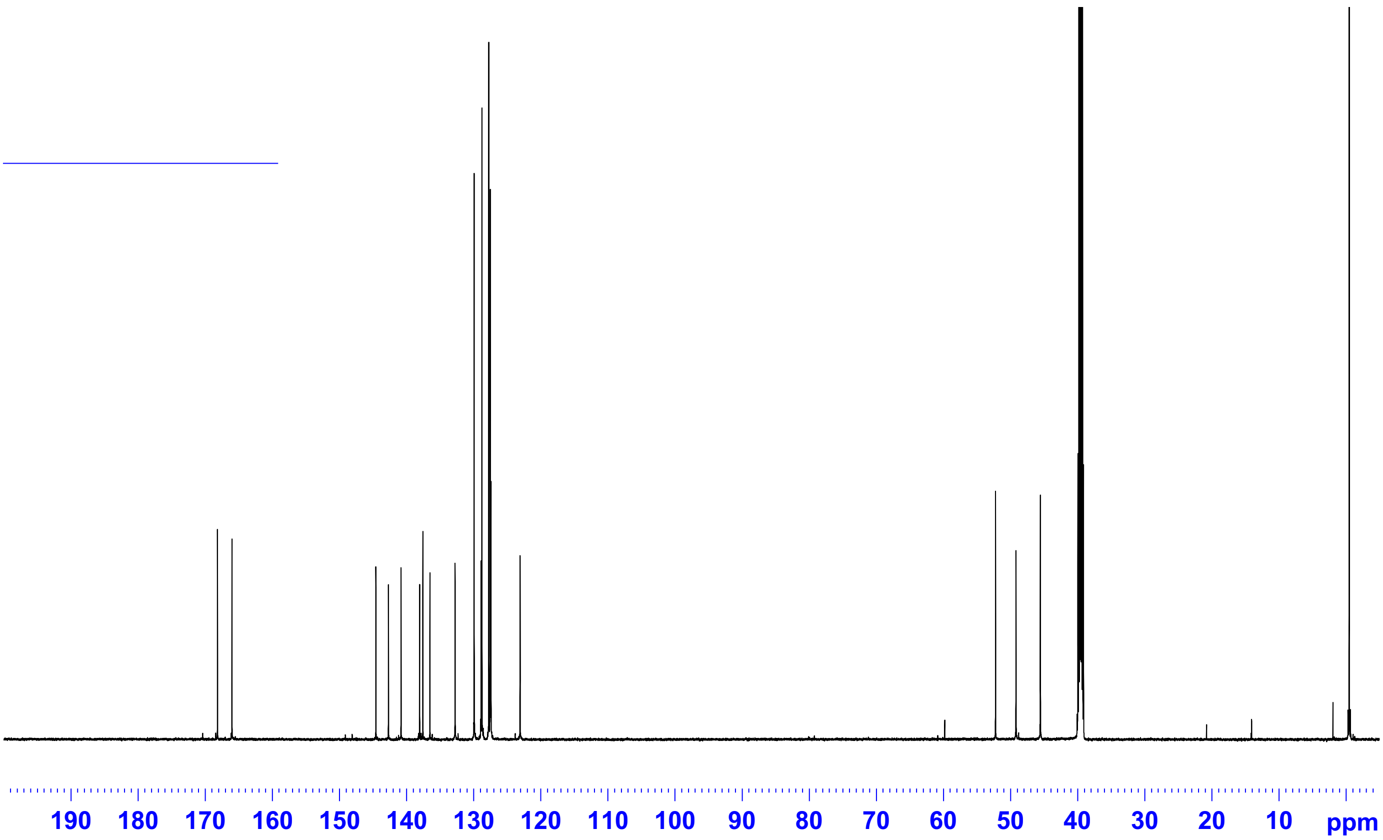

2-Benzyl-5-(4-methoxyphenyl)-7-(trimethylsilyl)isoindolin-1-one 10q

^{1}H NMR (600 MHz, DMSO-d$_6$)

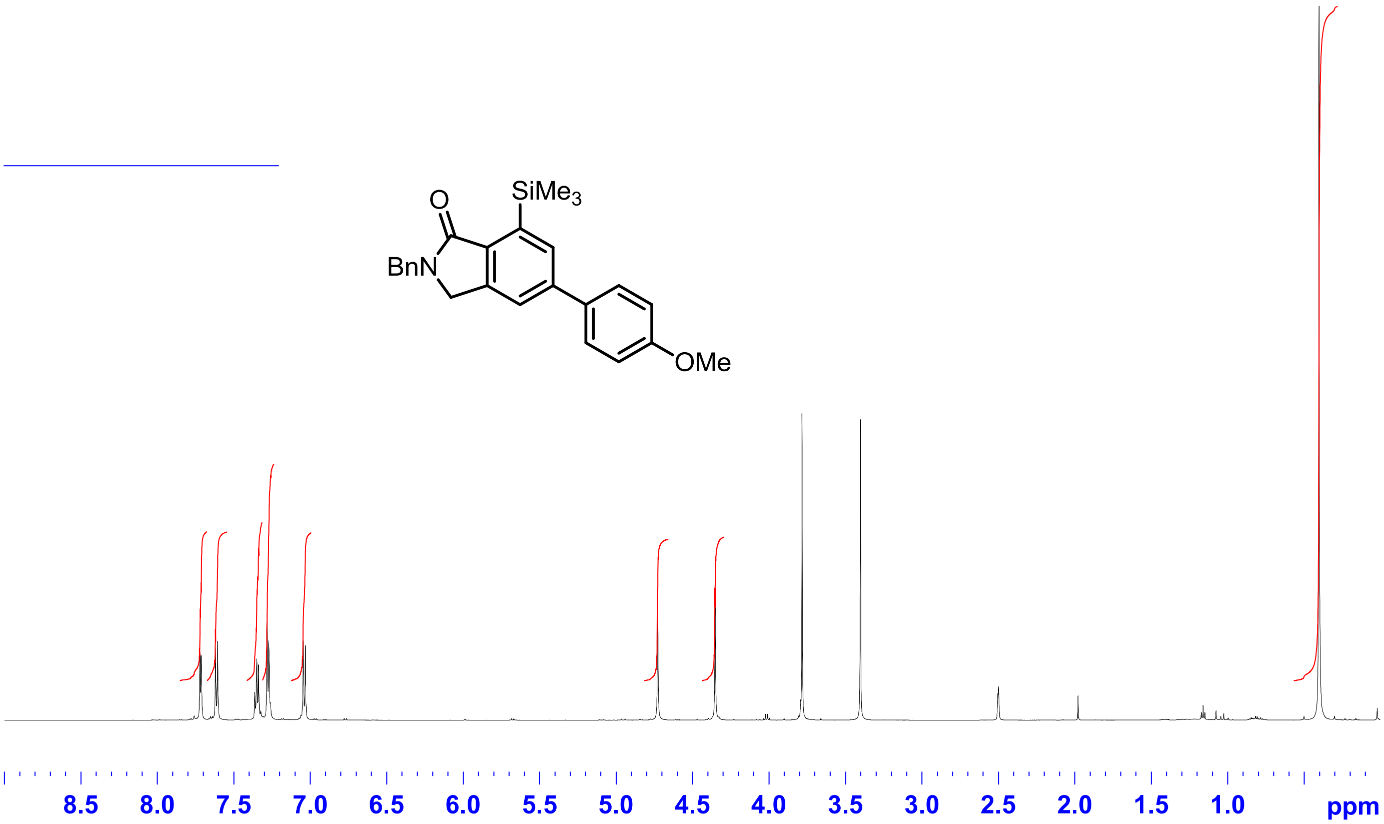

^{13}C NMR (150 MHz, DMSO-d$_6$)

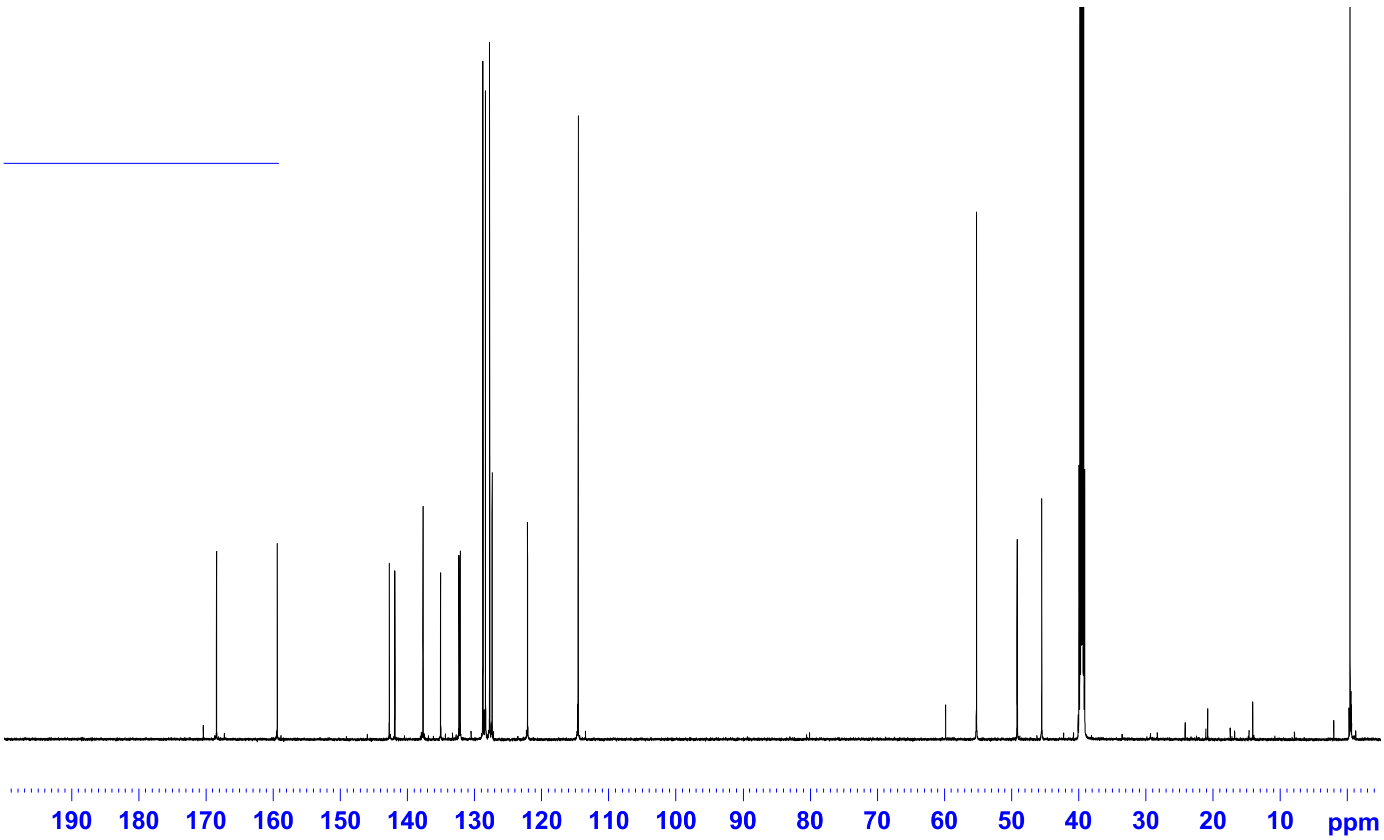

2-Benzyl-5-(4-(dimethylamino)phenyl)-7-(trimethylsilyl)isoindolin-1-one 10r

^{1}H NMR (600 MHz, DMSO-d$_6$)

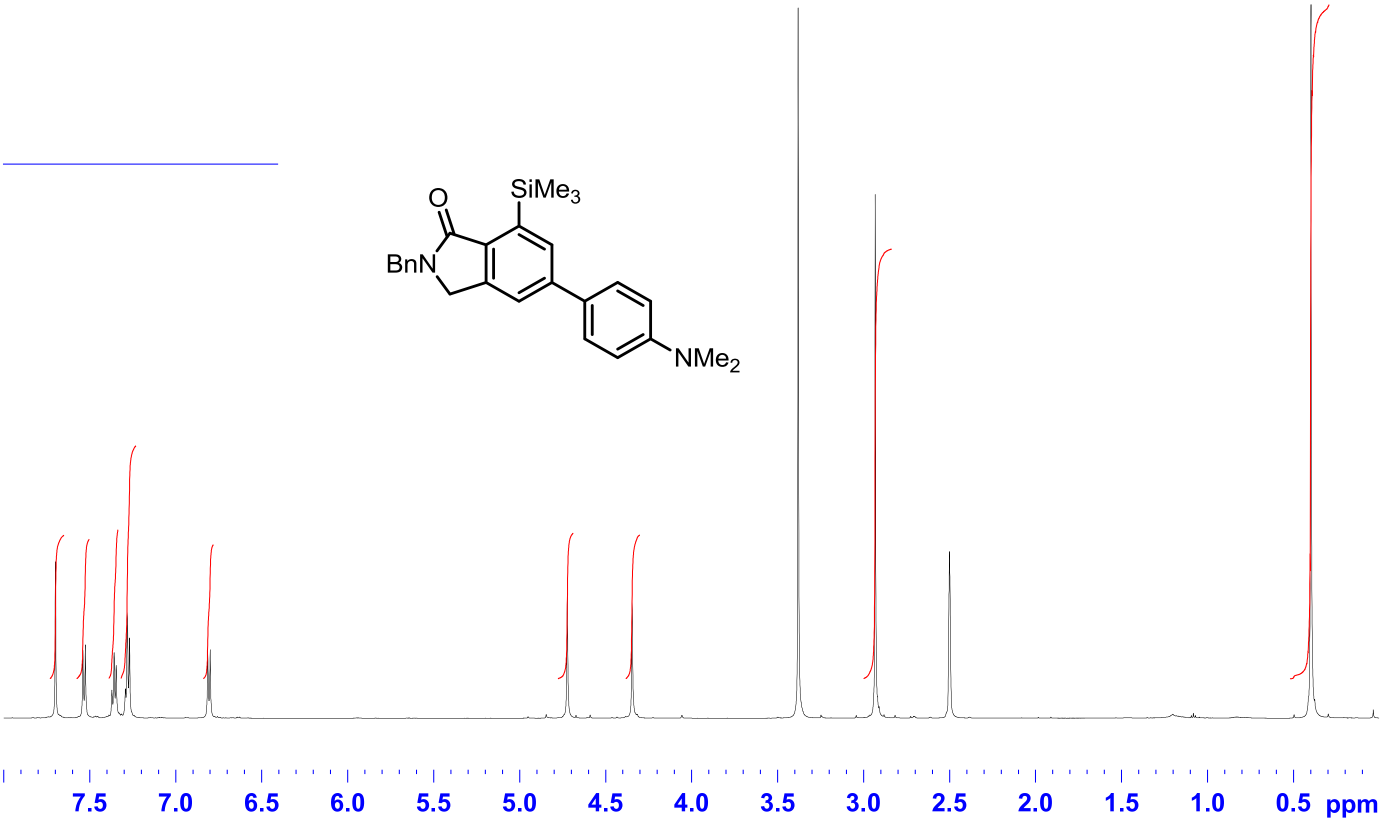

^{13}C NMR (150 MHz, DMSO-d$_6$)

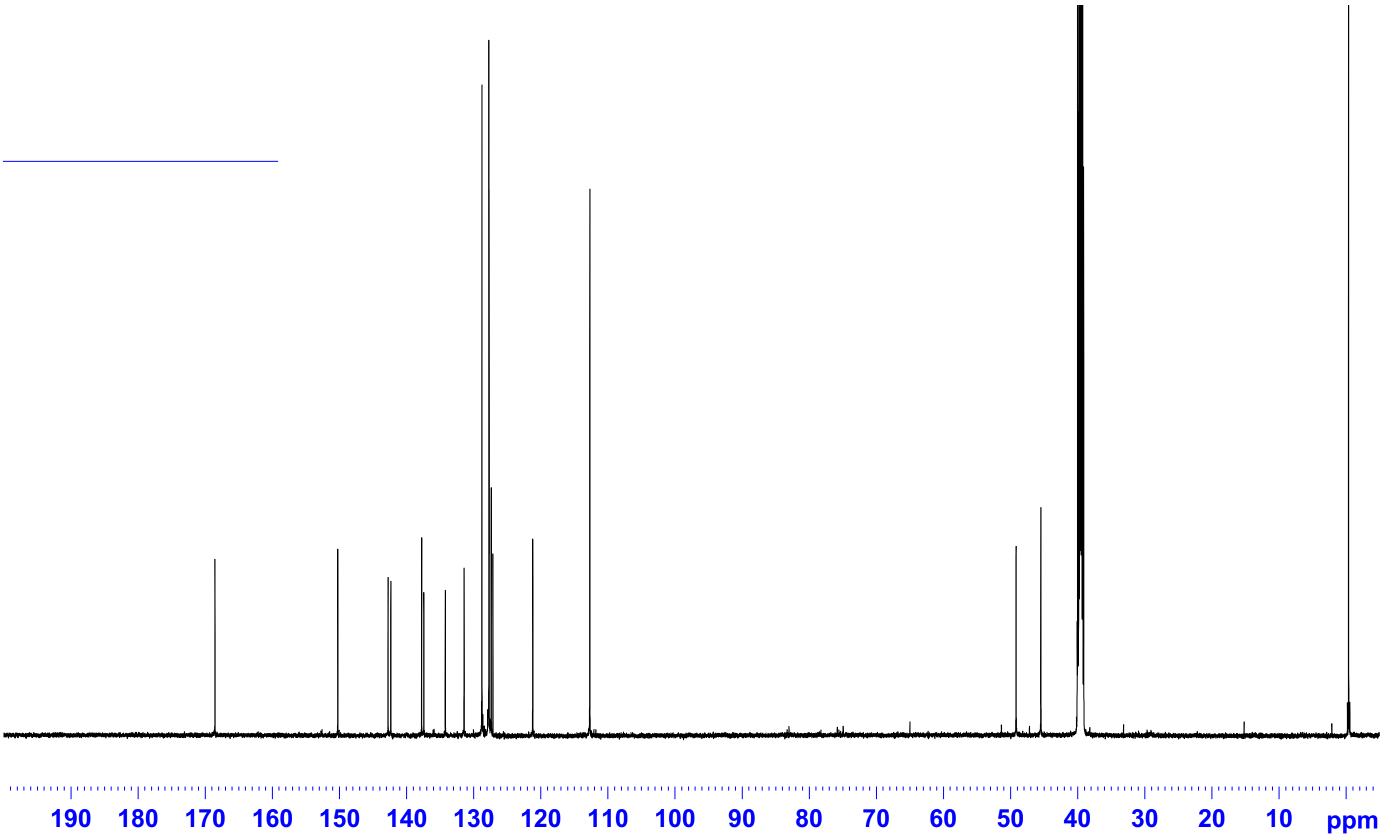

2-Benzyl-5-(pyridin-2-yl)-7-(trimethylsilyl)isoindolin-1-one 10t

^{1}H NMR (600 MHz, DMSO-d$_6$)

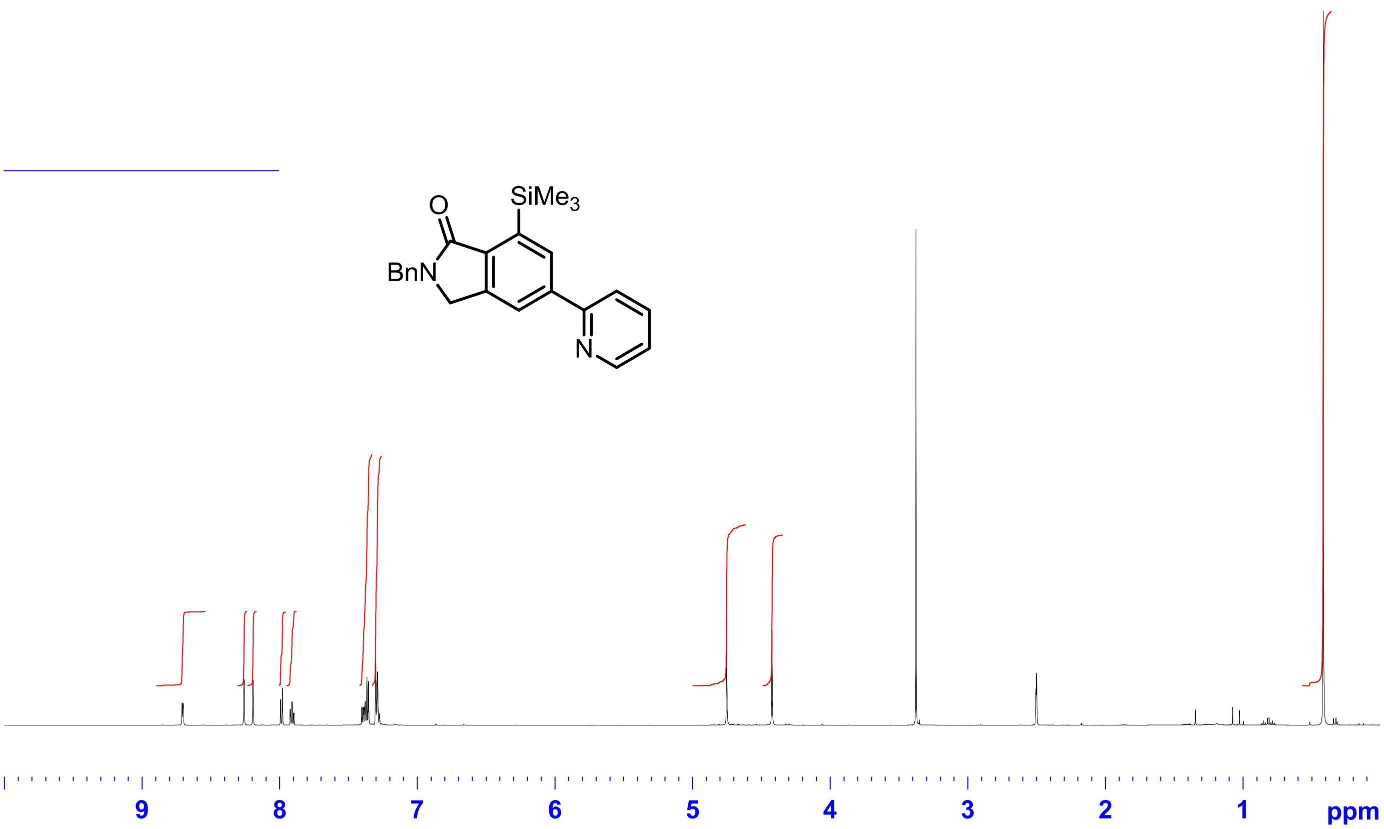

^{13}C NMR (150 MHz, DMSO-d$_6$)

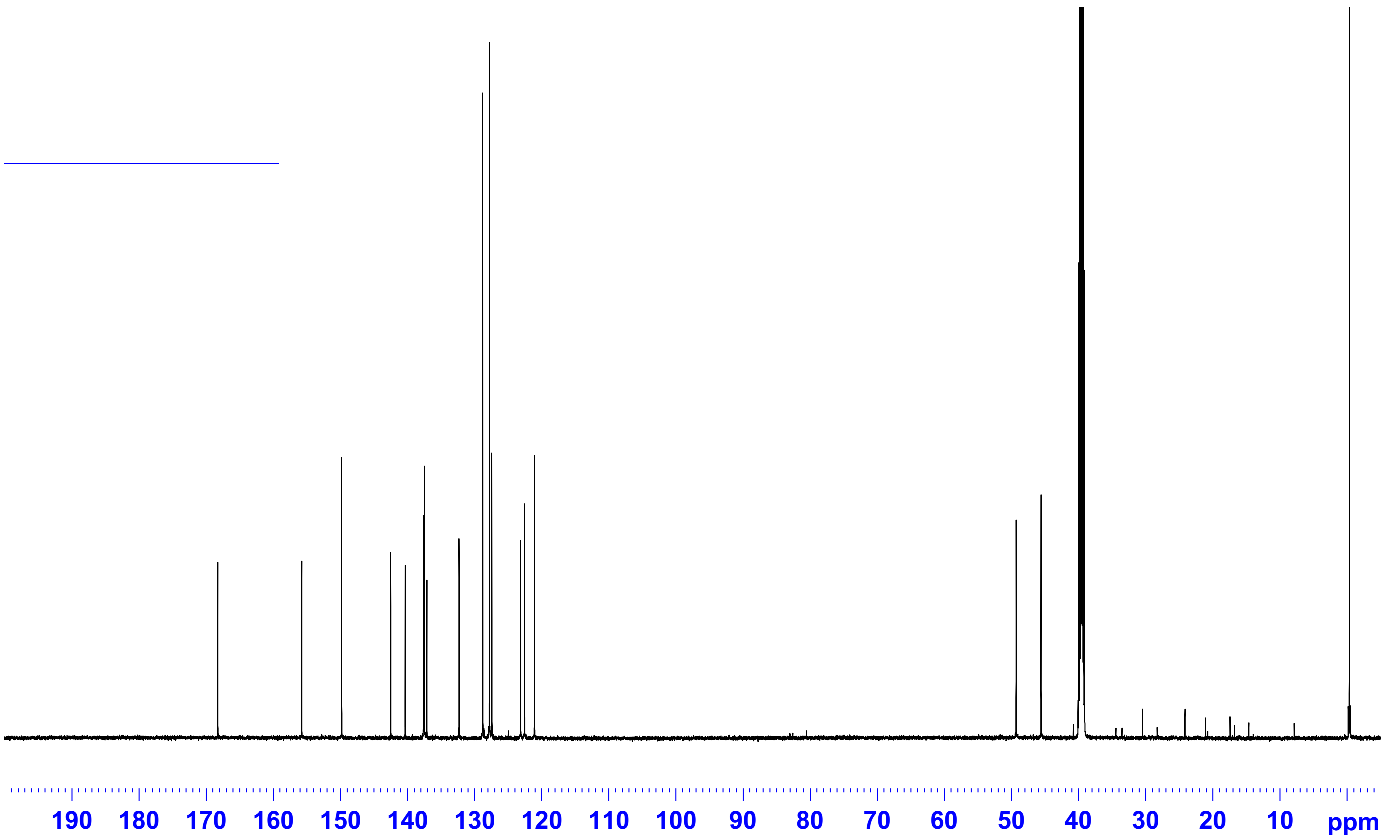

2-Benzyl-5-(1*H*-naphtho[1,8-*de*][1,3,2]diazaborinin-2(3*H*)-yl)-7-(trimethylsilyl)isoindolin-1-one 10v

^{1}H NMR (600 MHz, DMSO-d$_6$)

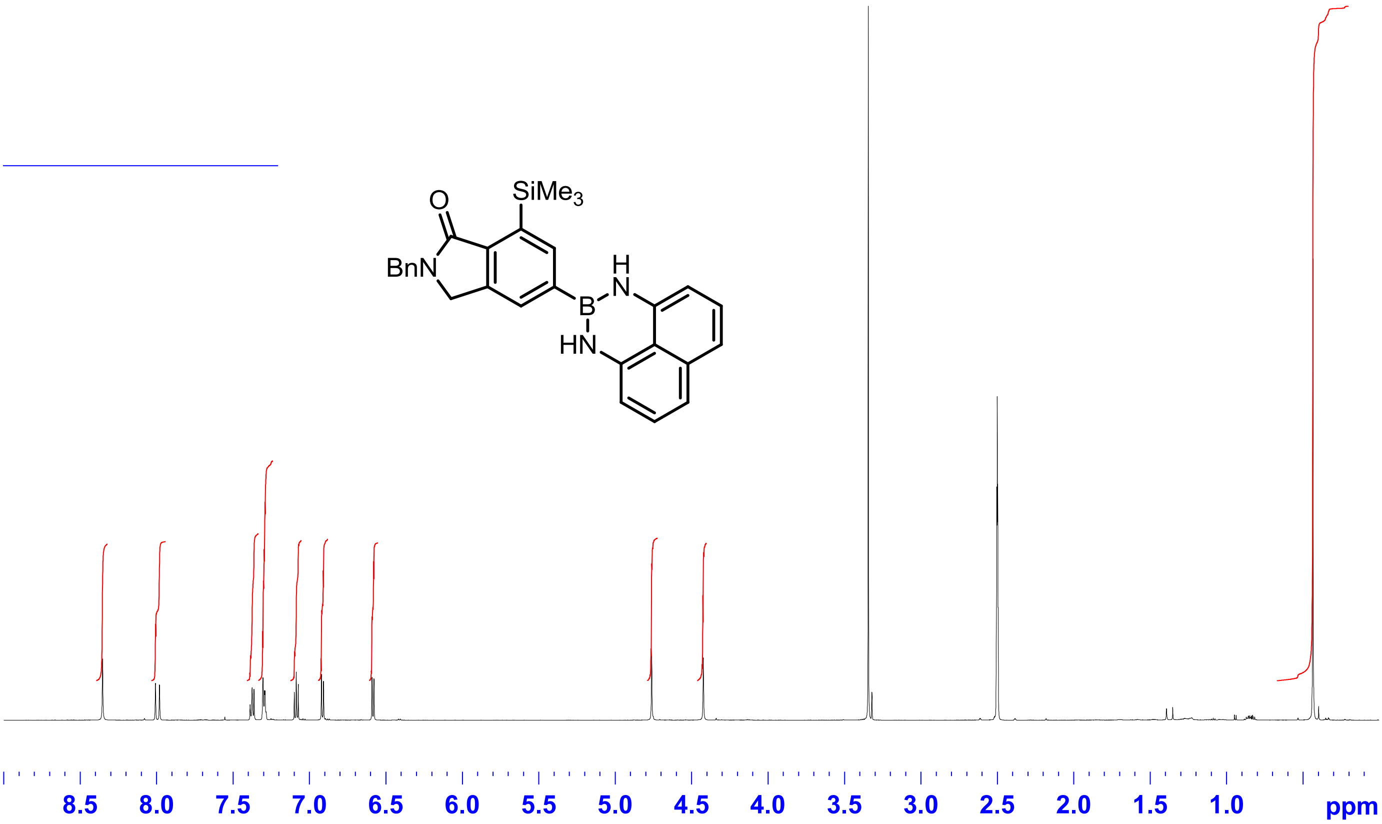

^{13}C NMR (150 MHz, DMSO-d$_6$)

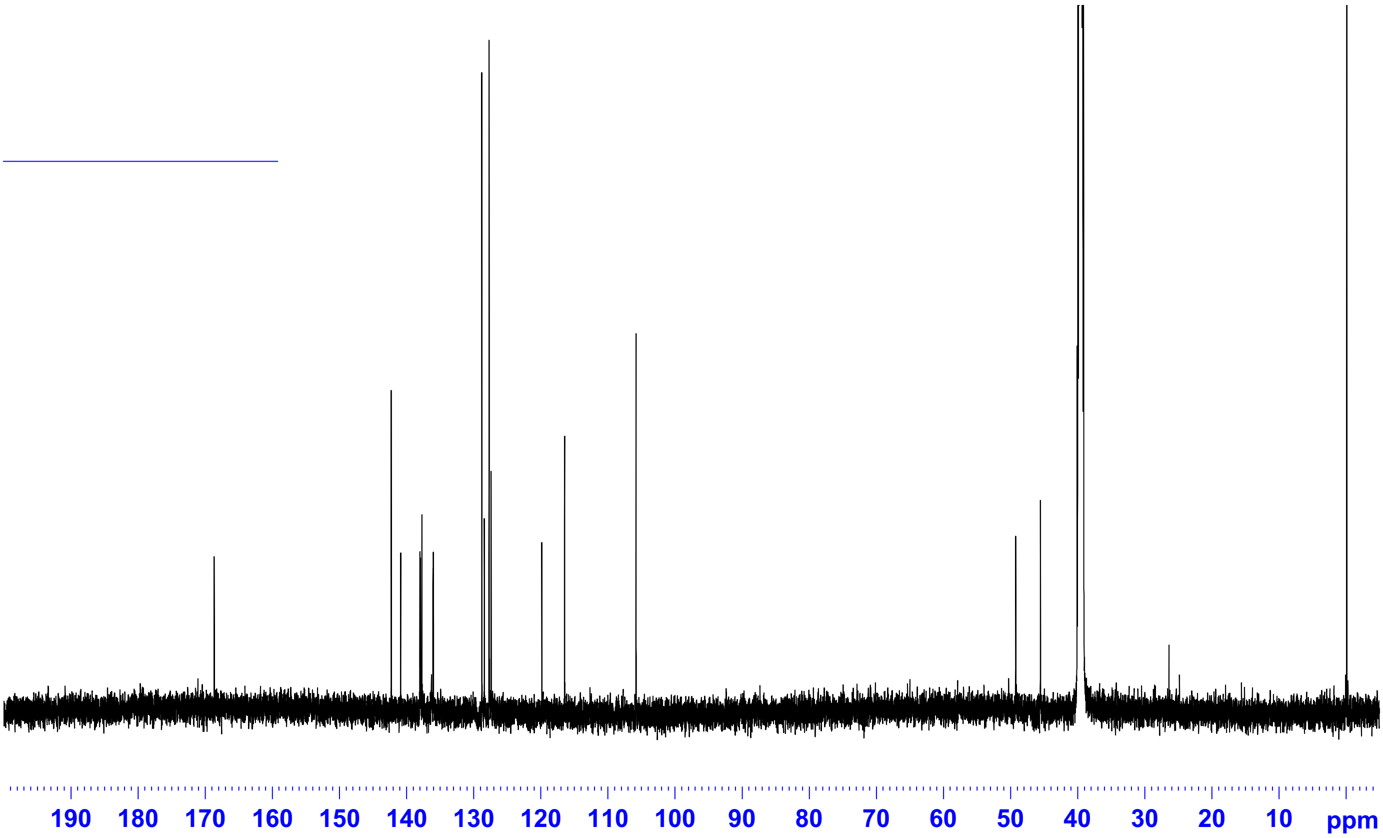

N-Benzyl-N-((2-benzyl-1-oxo-7-(trimethylsilyl)isoindolin-5-yl)methyl)-3-(trimethylsilyl)propiolamide 11

^{1}H NMR (600 MHz, DMSO-d$_6$)

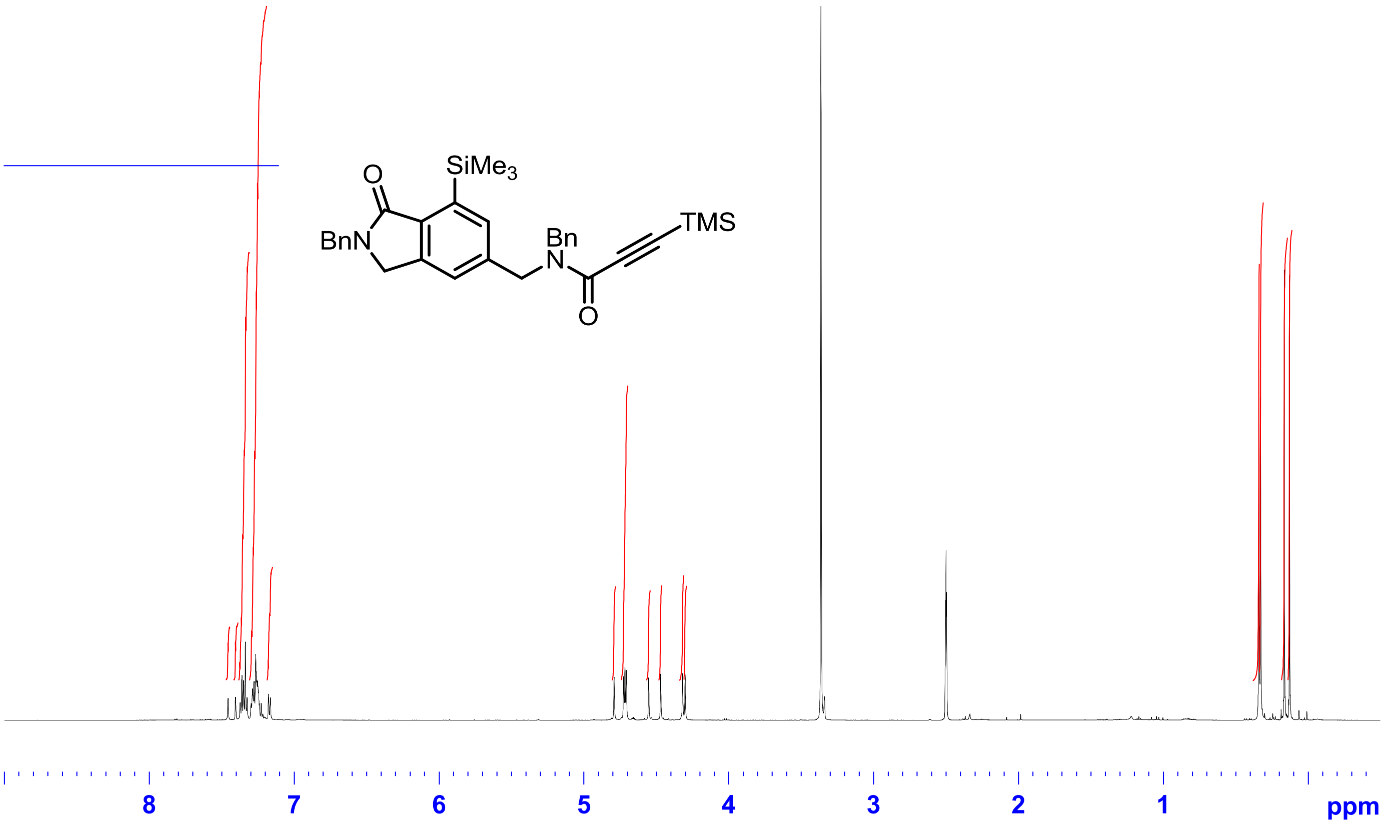

^{13}C NMR (150 MHz, DMSO-d$_6$)

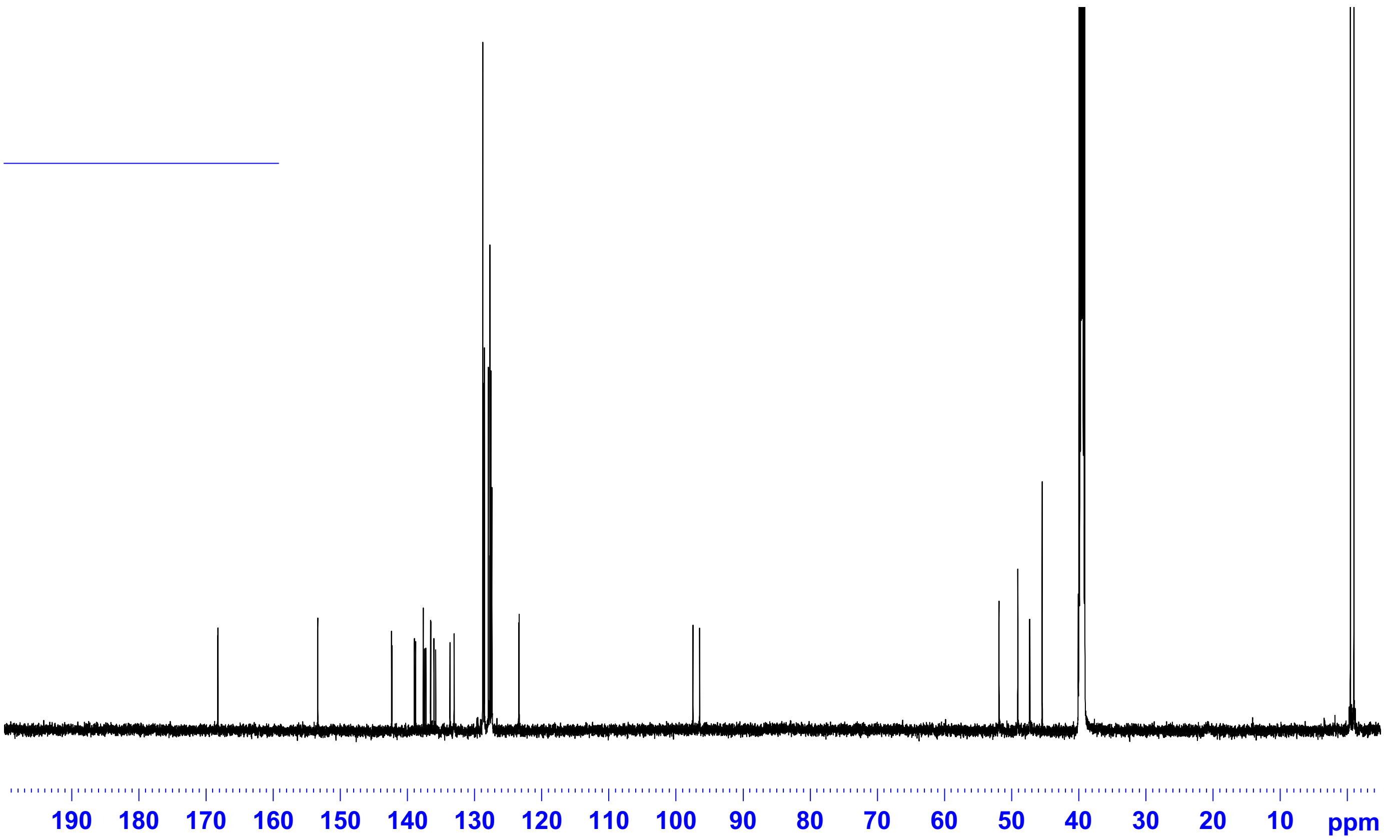

2-(*tert*-Butyl)-5-butyl-7-(trimethylsilyl)isoindolin-1-one 13a

^{1}H NMR (600 MHz, DMSO-d_6)

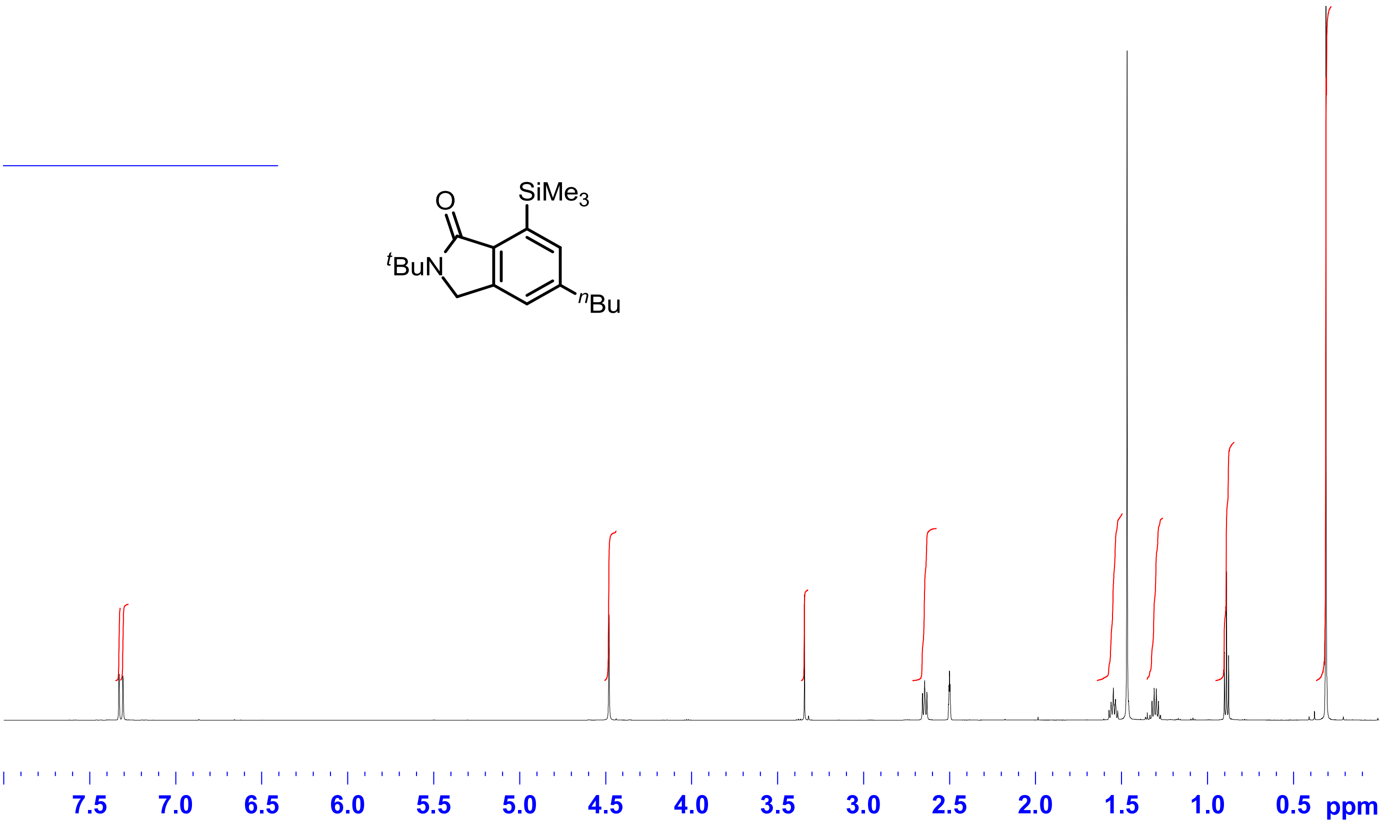

^{13}C NMR (150 MHz, DMSO-d_6)

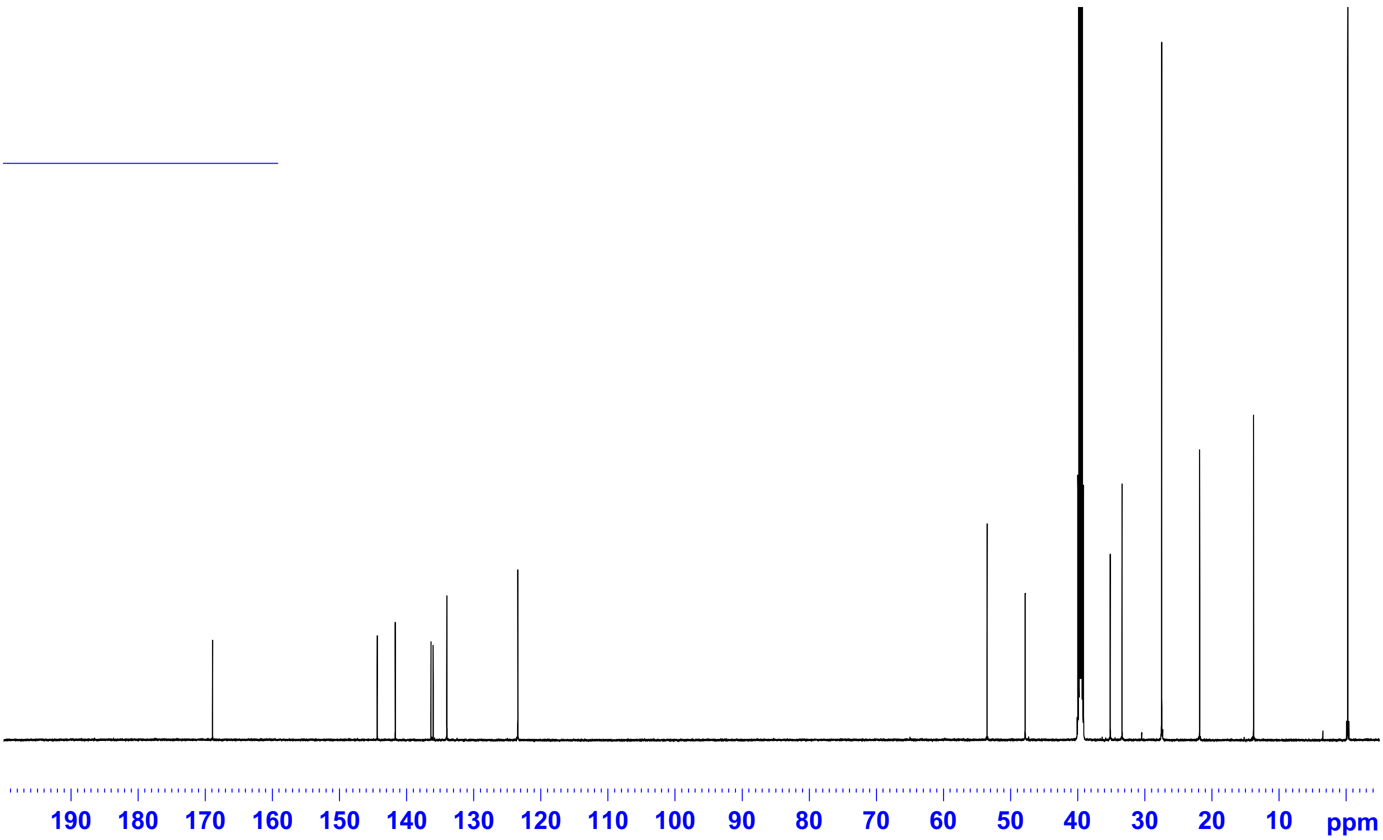

2-(*tert*-Butyl)-5-phenyl-7-(trimethylsilyl)isoindolin-1-one 13b

^{1}H NMR (600 MHz, DMSO-d$_6$)

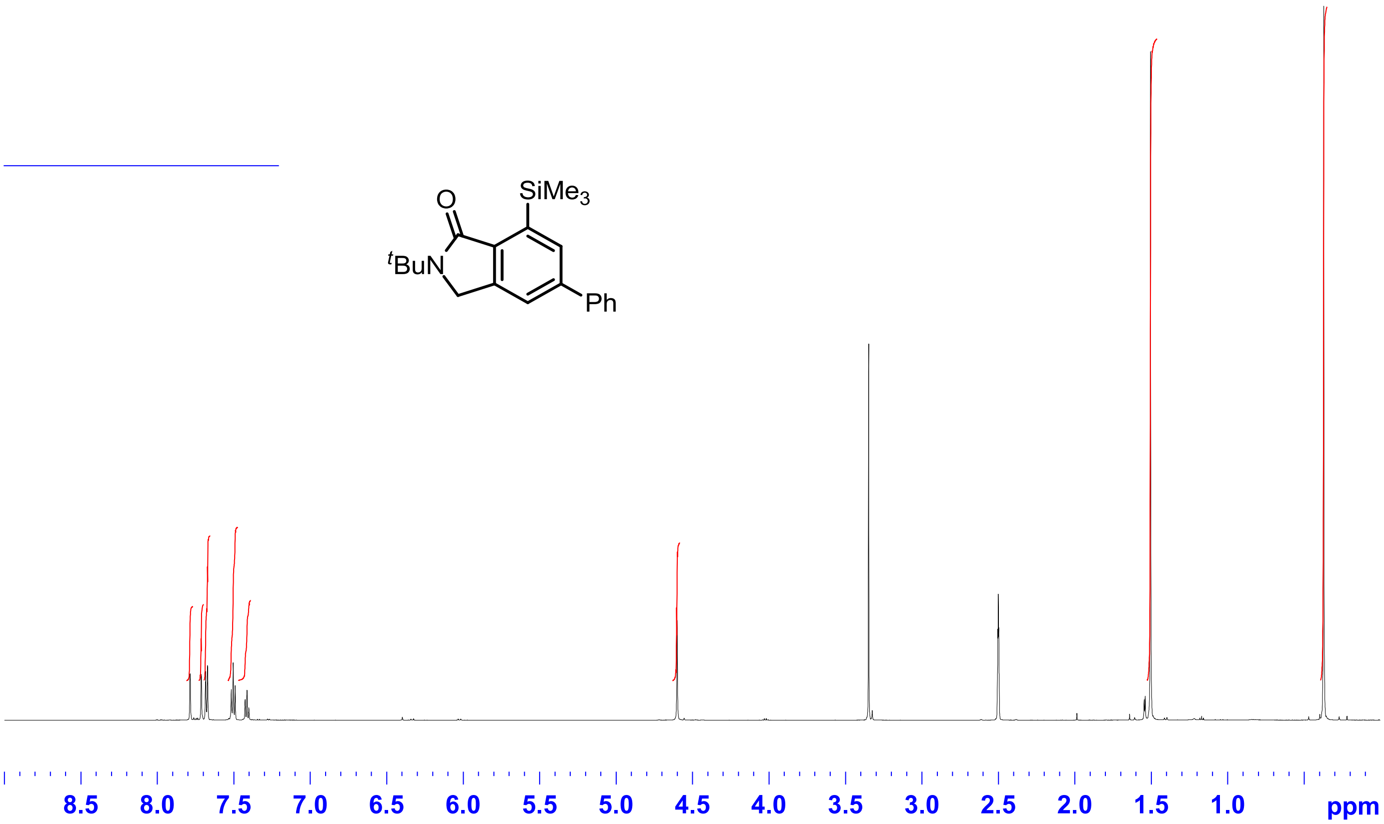

^{13}C NMR (150 MHz, DMSO-d$_6$)

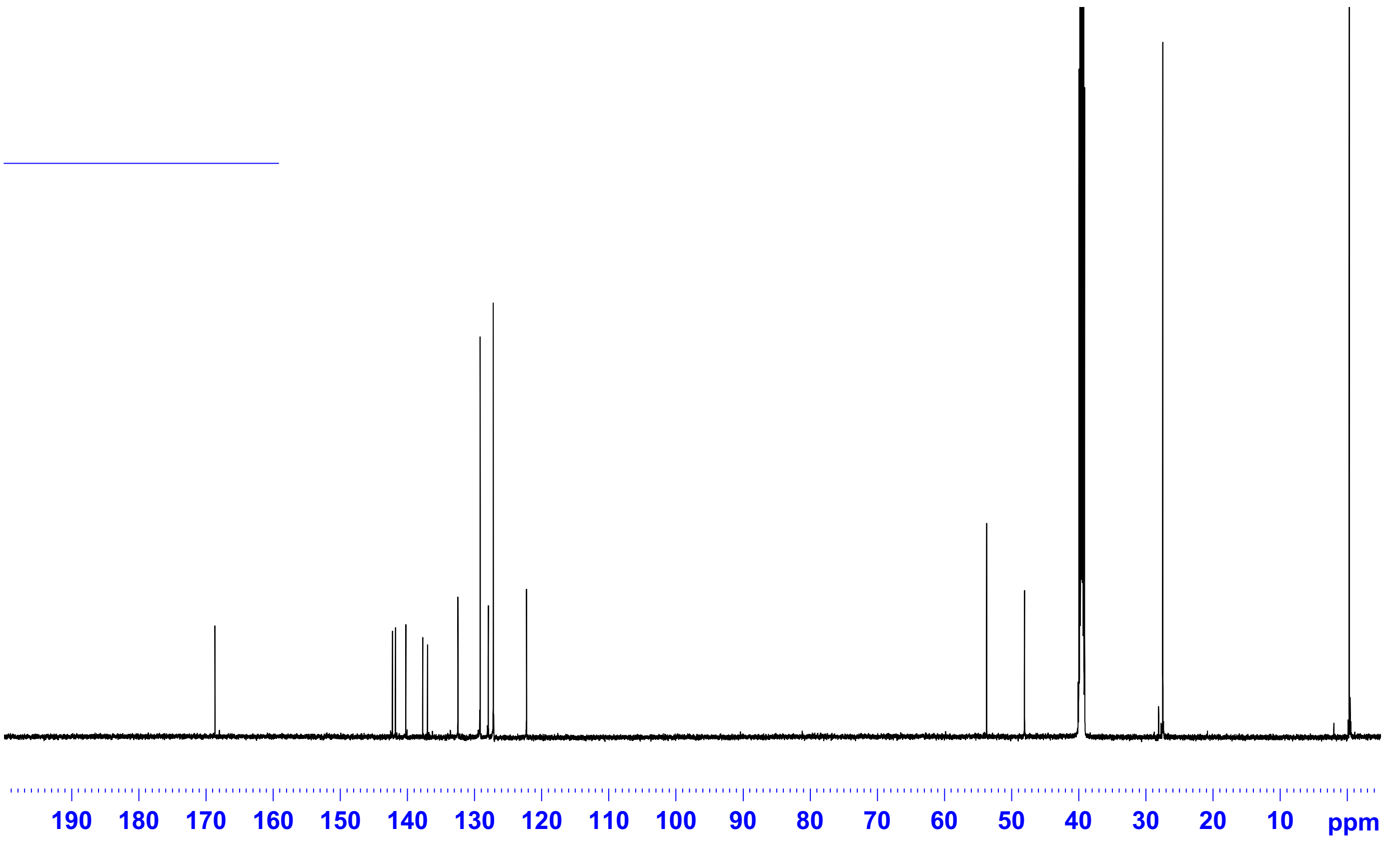

2-(*tert*-Butyl)-5-(*o*-tolyl)-7-(trimethylsilyl)isoindolin-1-one 13c

^{1}H NMR (600 MHz, DMSO-d$_6$)

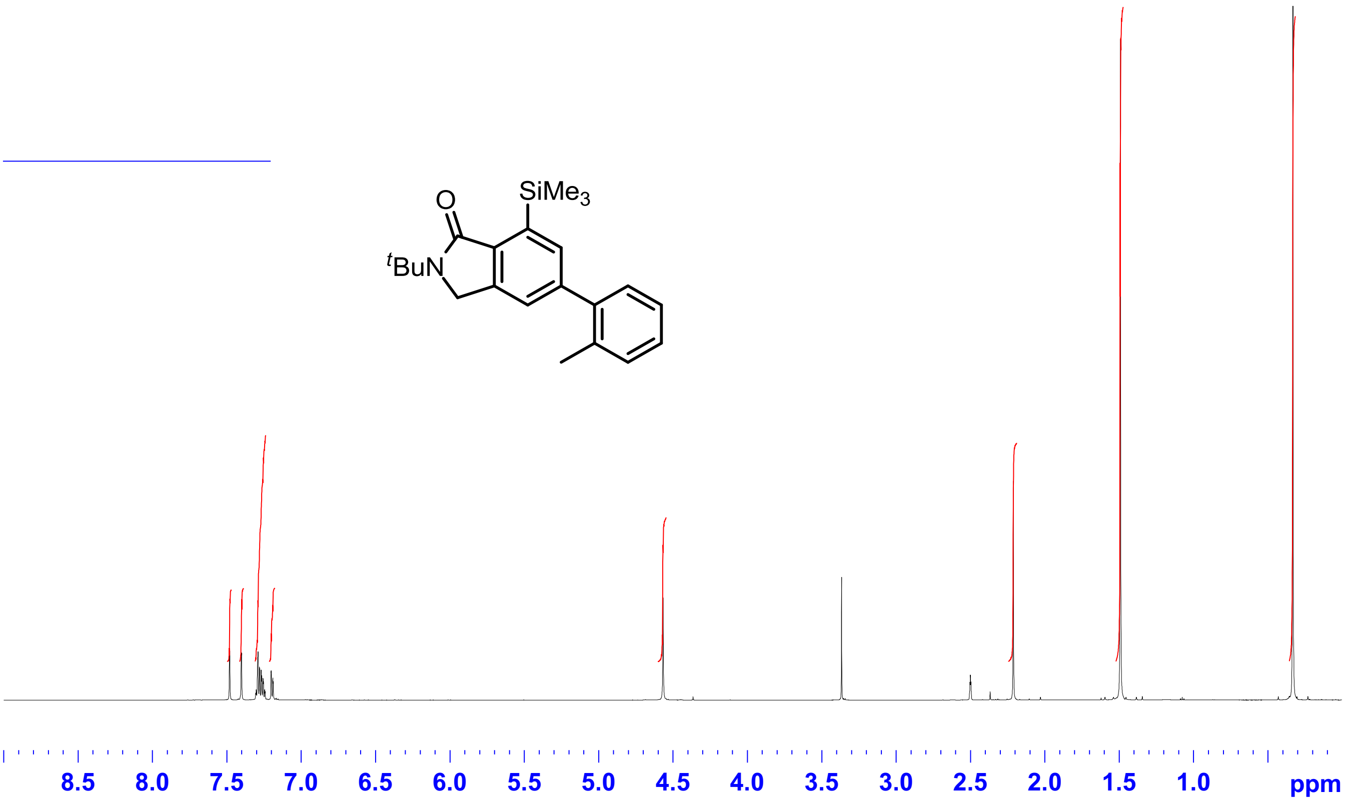

^{13}C NMR (150 MHz, DMSO-d$_6$)

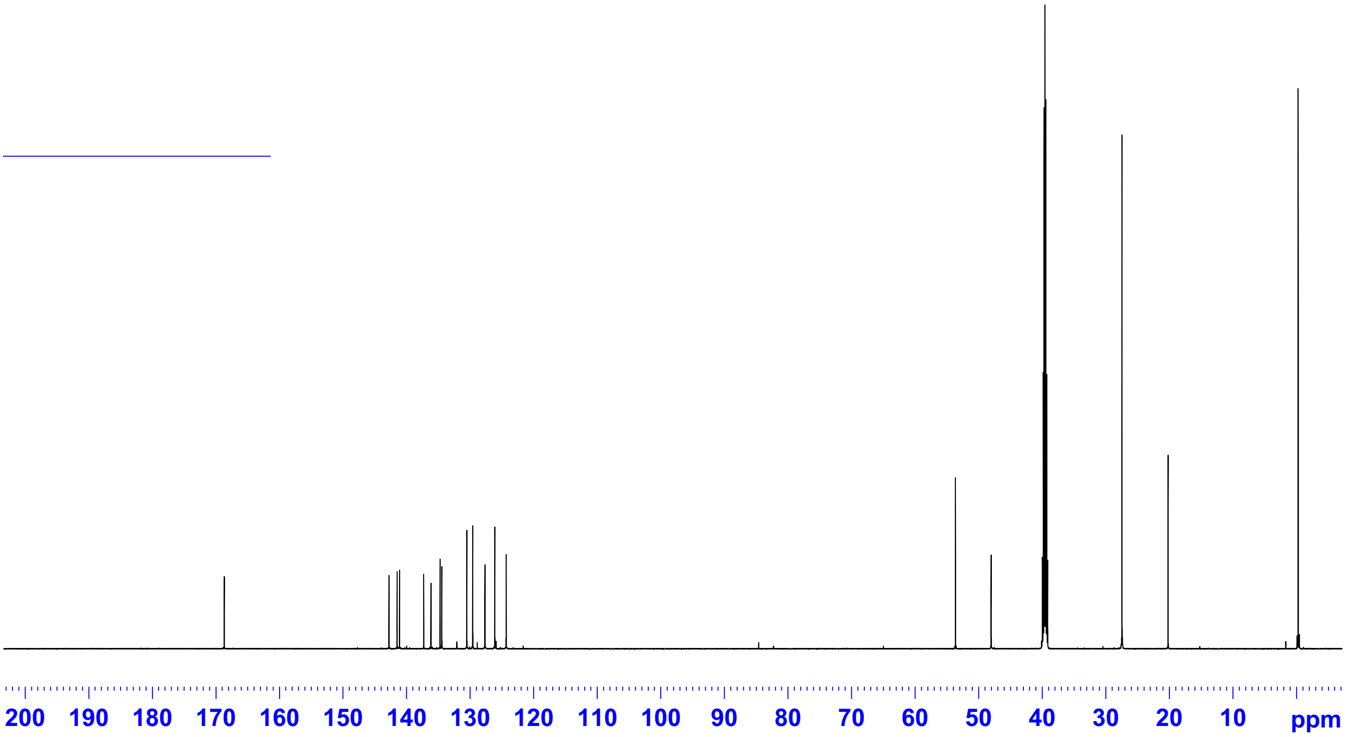

5-Butyl-7-(trimethylsilyl)isoindolin-1-one 13d

^{1}H NMR (600 MHz, DMSO-d$_6$)

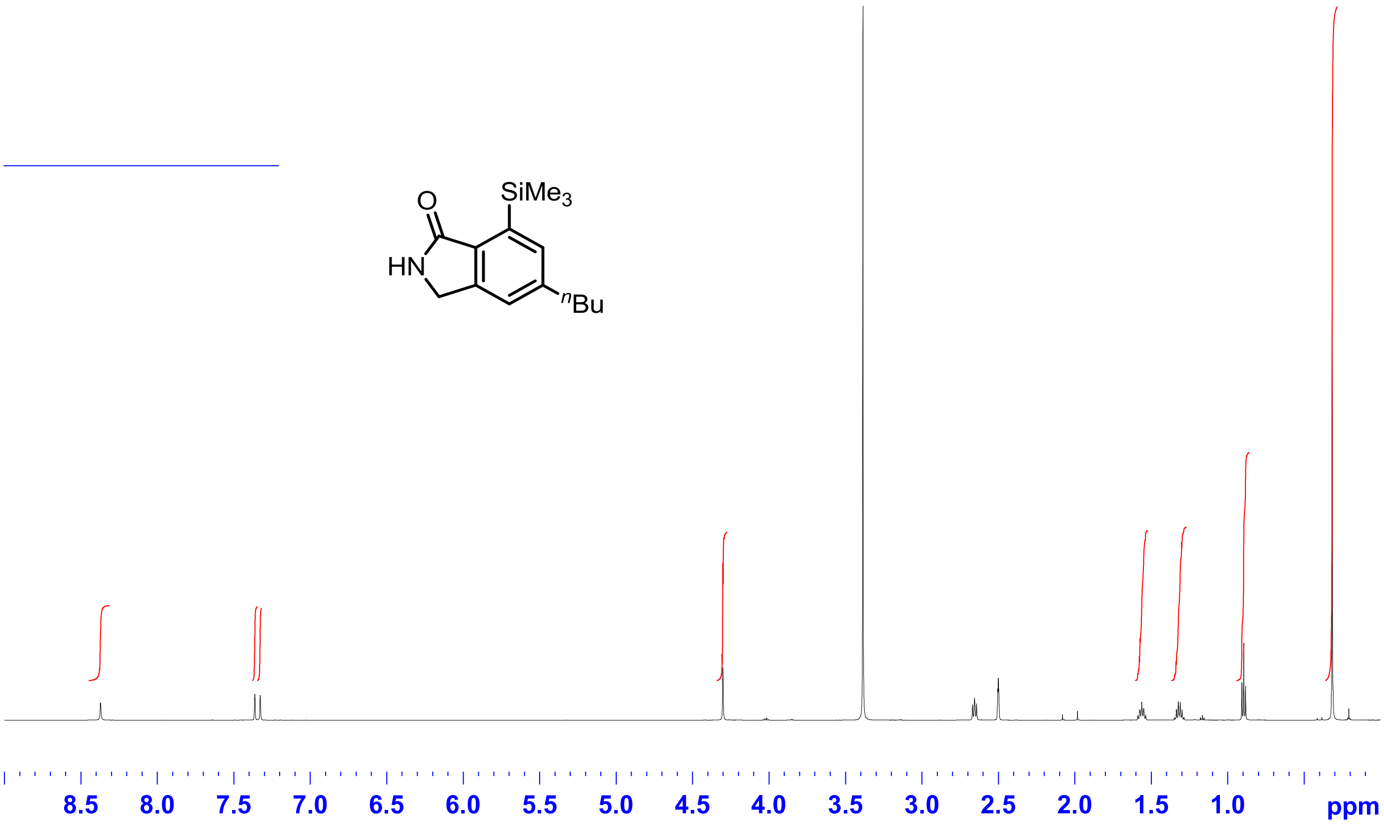

^{13}C NMR (150 MHz, DMSO-d$_6$)

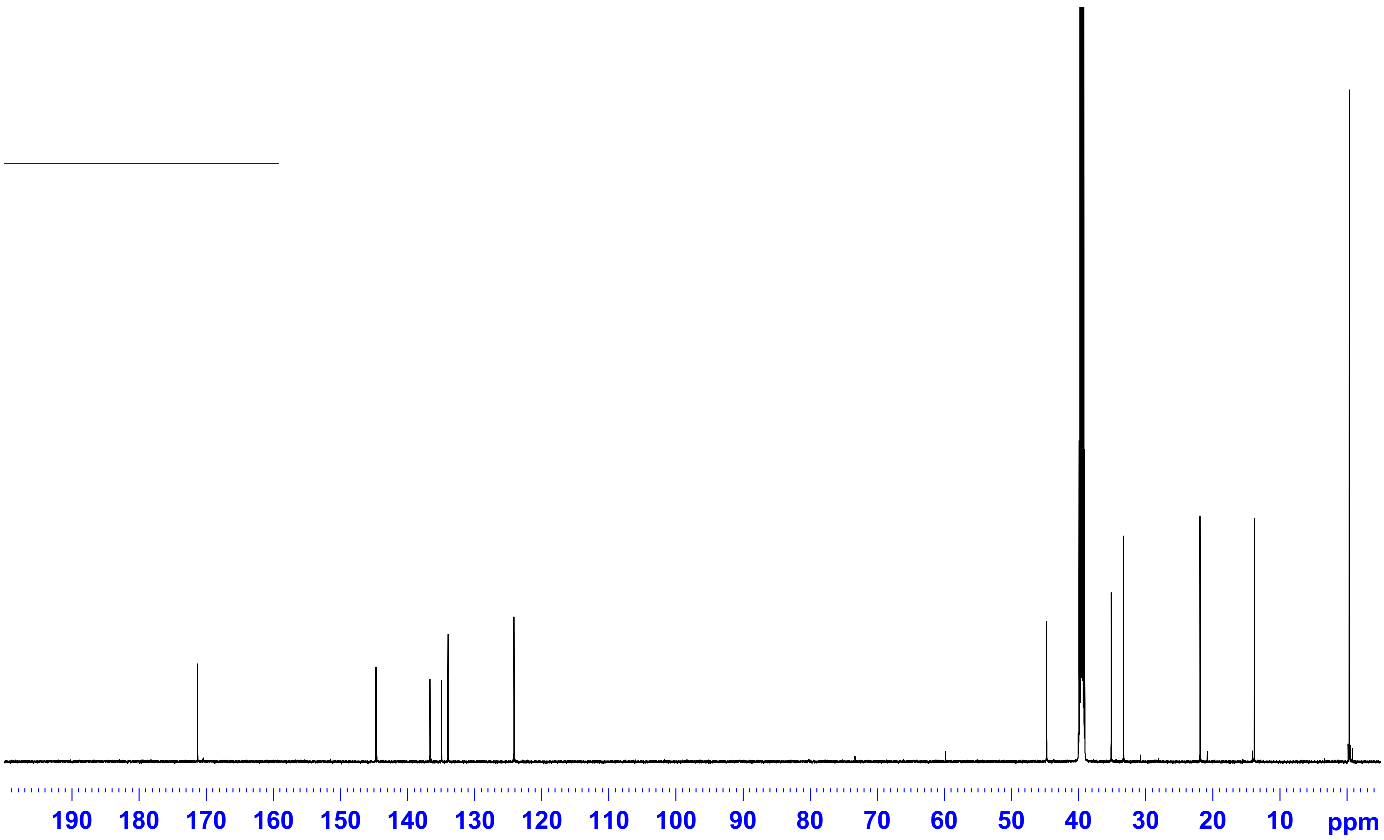

5-(*o*-Tolyl)-7-(trimethylsilyl)isoindolin-1-one 13e

^{1}H NMR (600 MHz, DMSO-d_6)

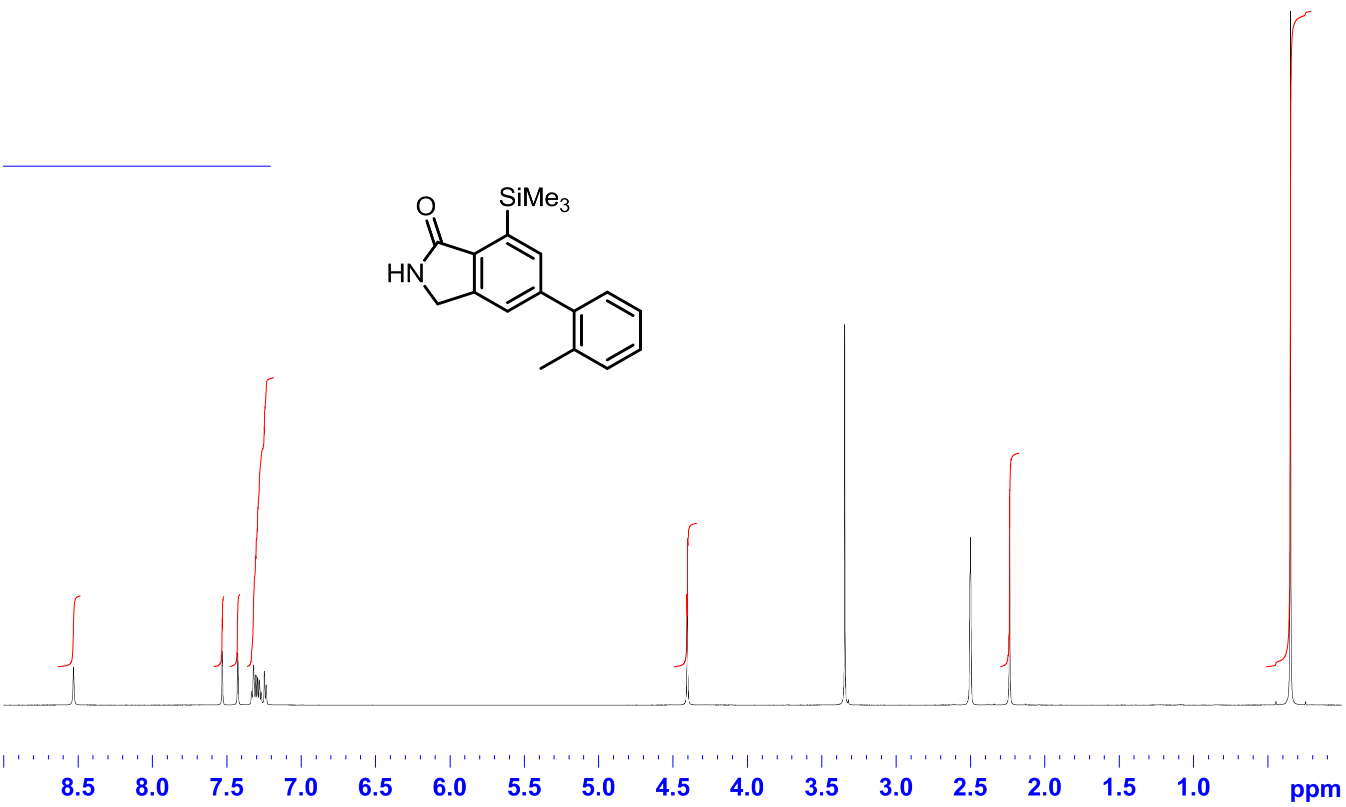

^{13}C NMR (150 MHz, DMSO-d_6)

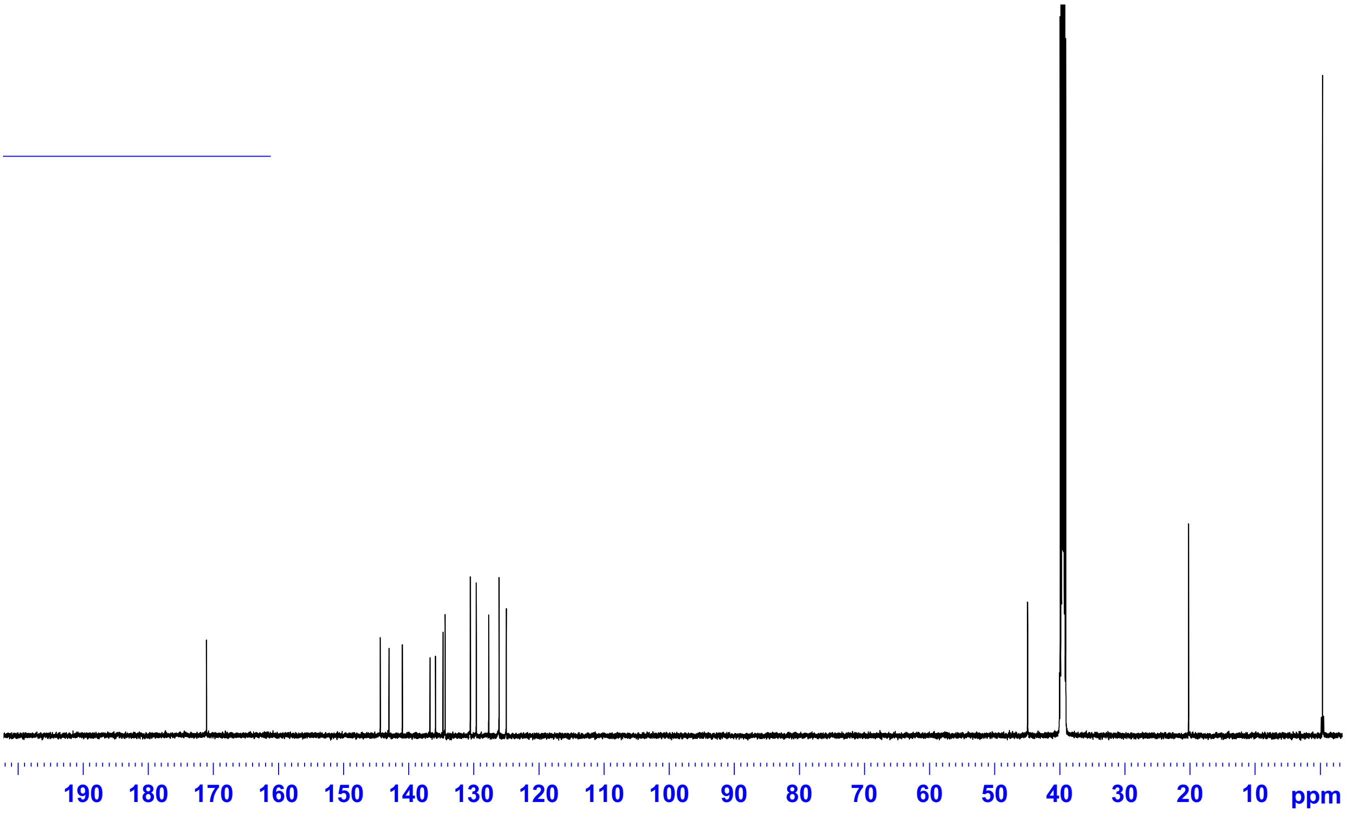

_N_-(_tert_-Butyl)-_N_-((2-(_tert_-butyl)-1-oxo-7-(trimethylsilyl)isoindolin-5-yl)methyl)-3-(trimethylsilyl)propiolamide 14a

^{1}H NMR (400 MHz, DMSO-d_6)

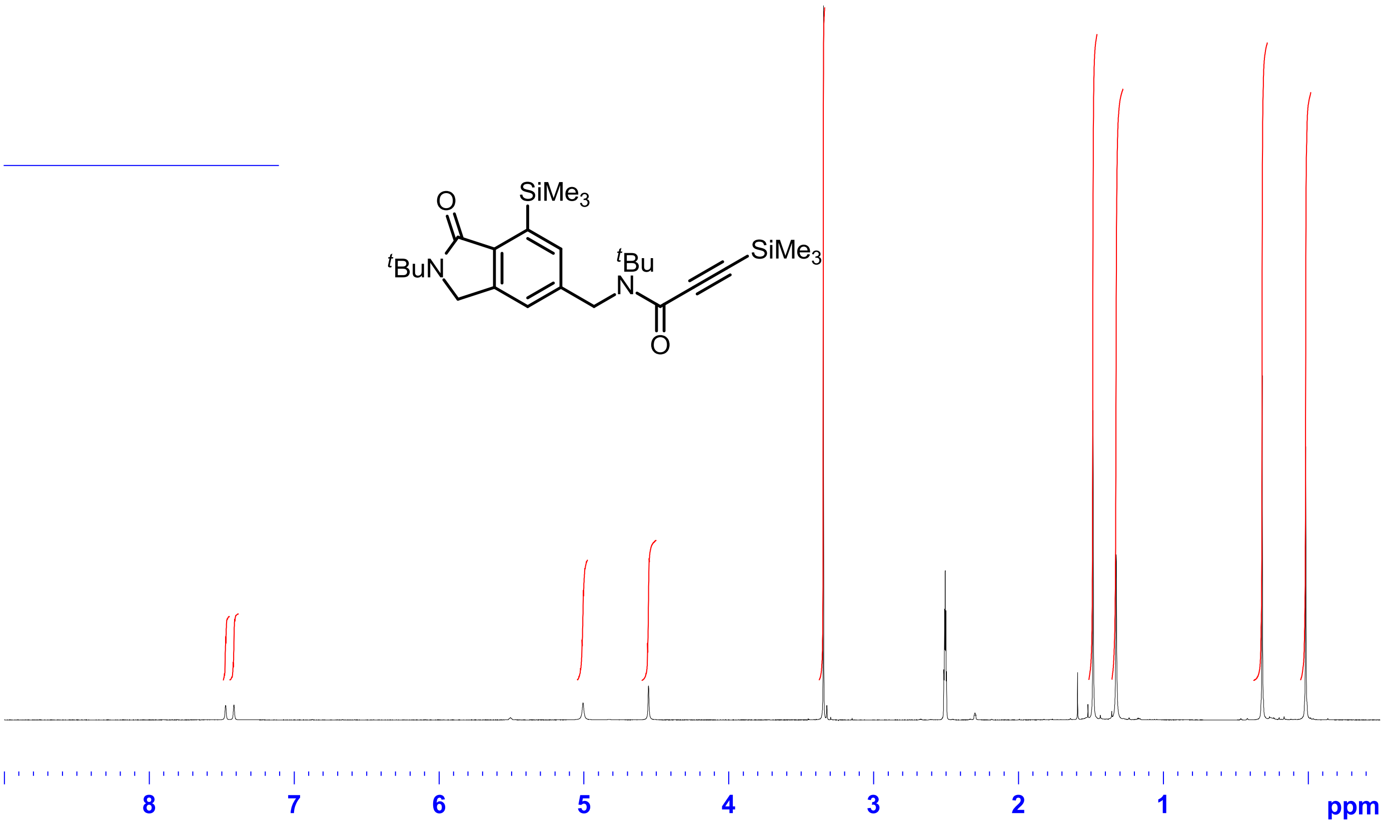

^{13}C NMR (125 MHz, DMSO-d_6)

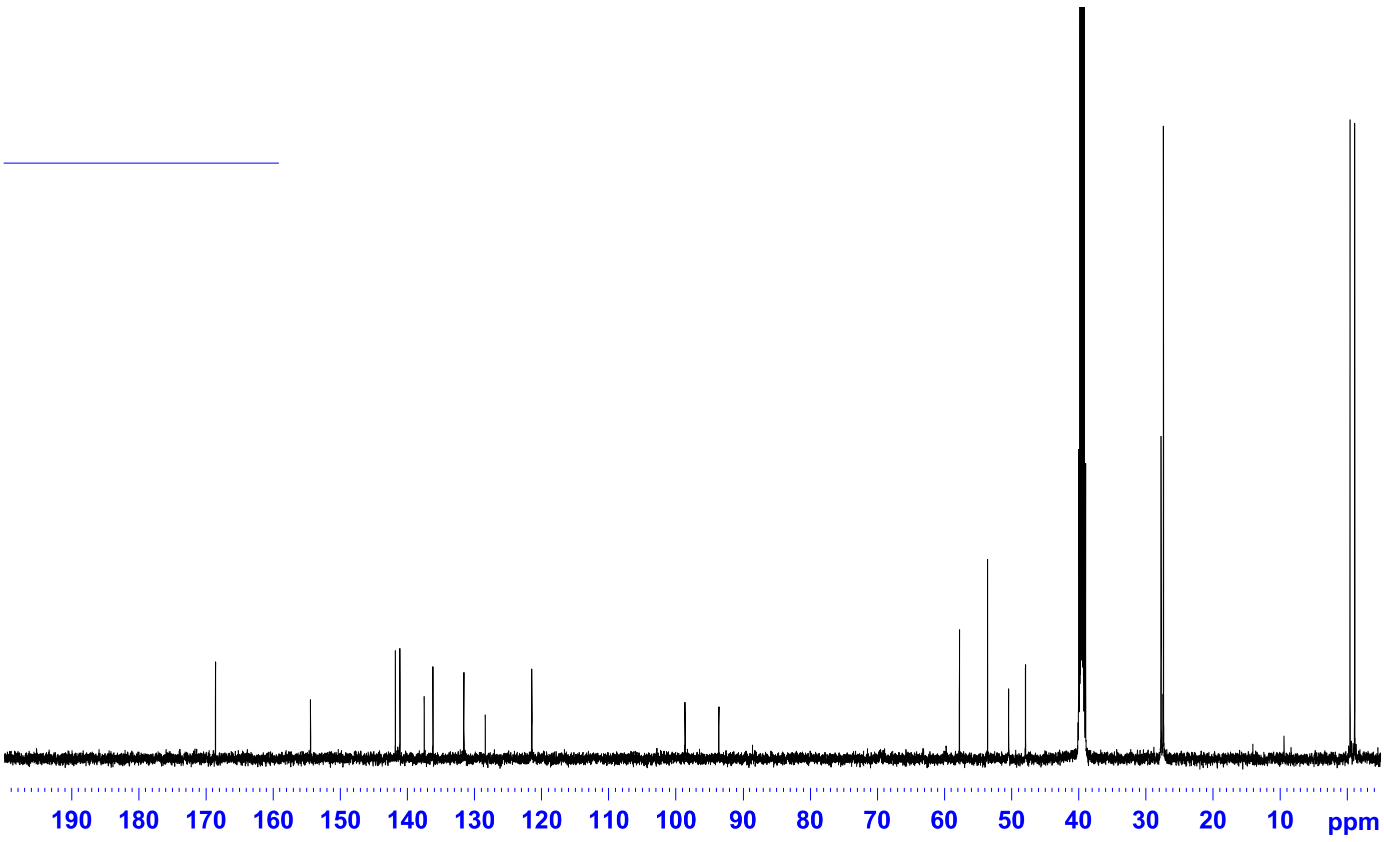

***N*-((1-oxo-7-(trimethylsilyl)isoindolin-5-yl)methyl)-3-(trimethylsilyl)propiolamide 14b**

^{1}H NMR (600 MHz, DMSO-d$_6$)

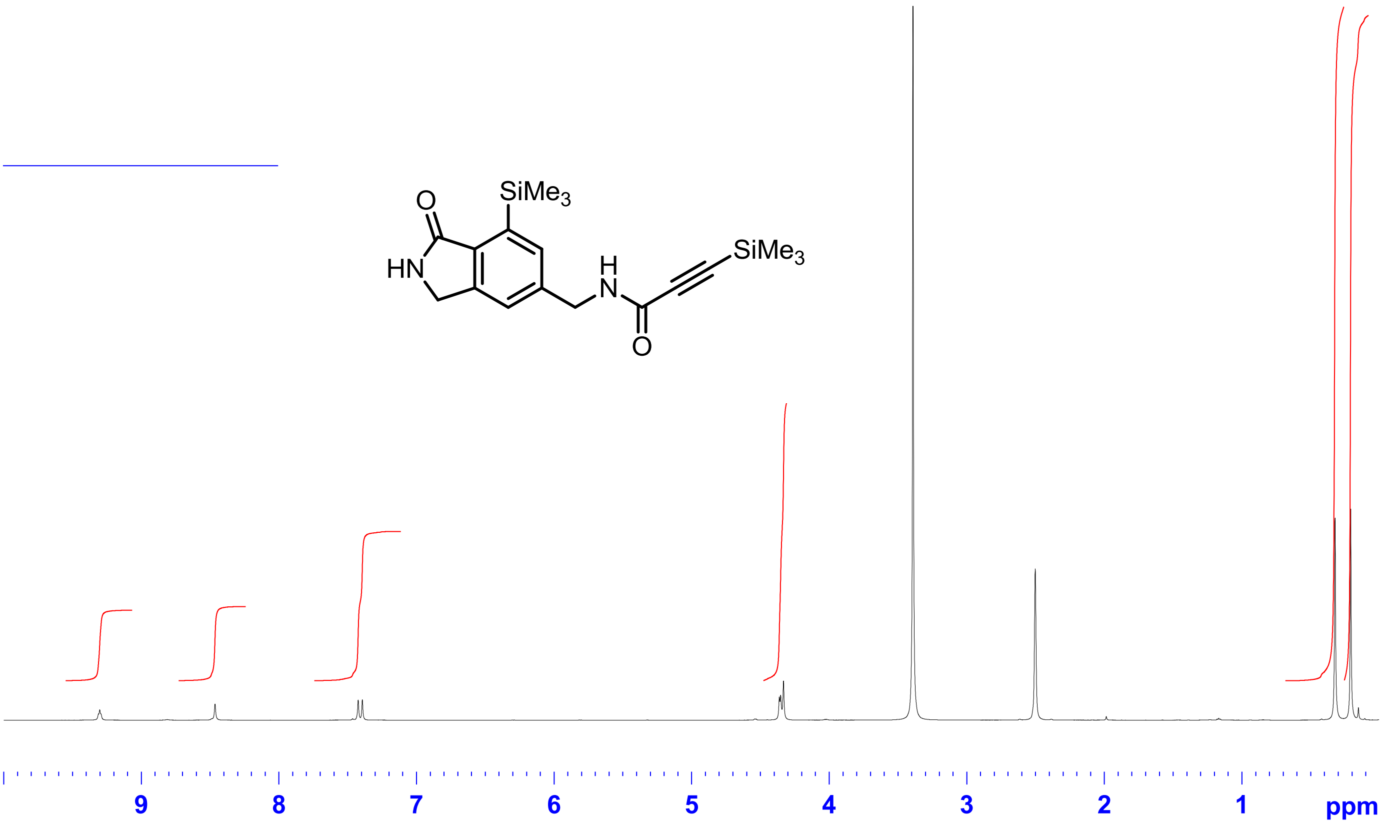

^{13}C NMR (150 MHz, DMSO-d$_6$)

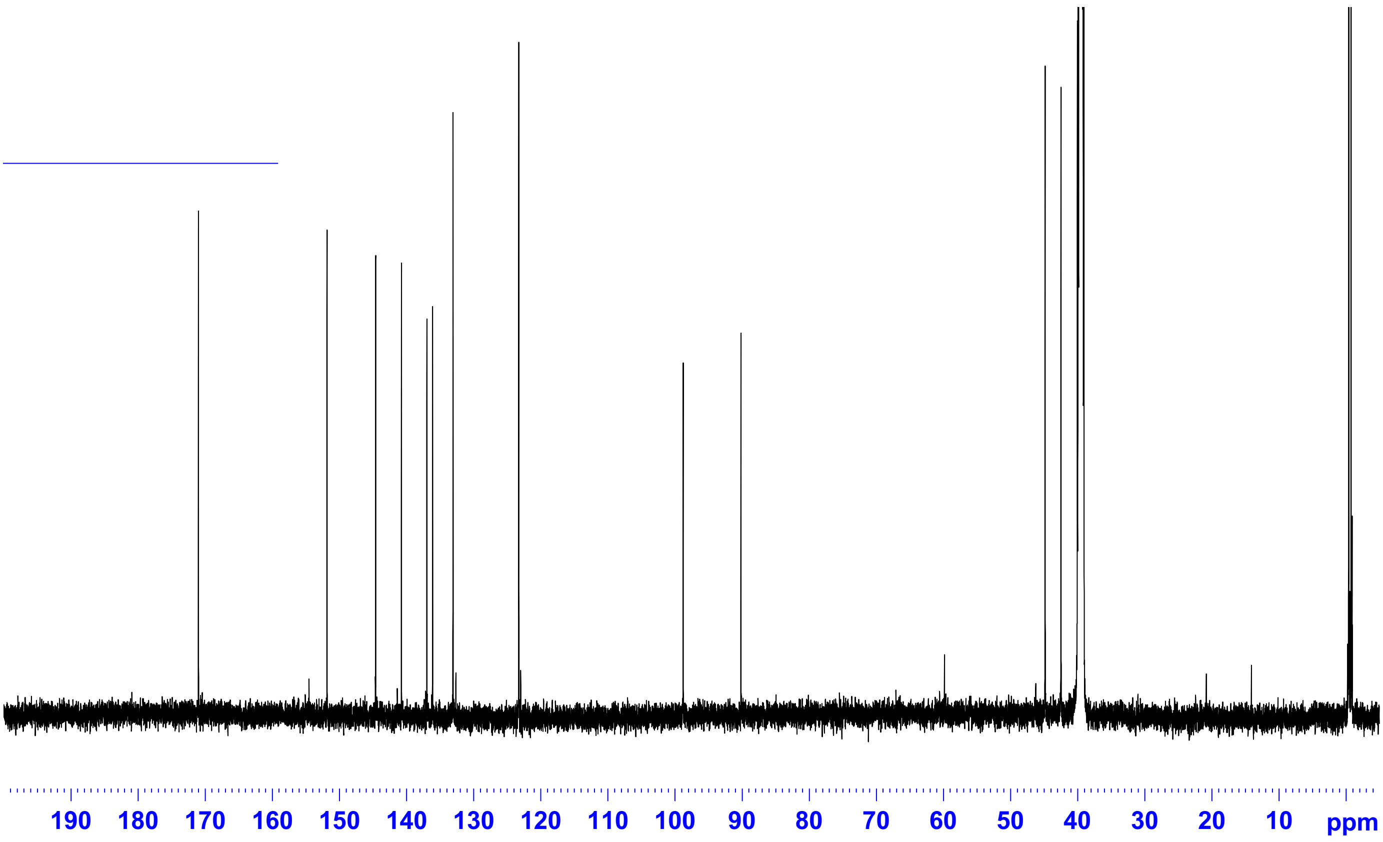

2-Benzyl-5-butyl-4-methyl-7-(trimethylsilyl)isoindolin-1-one 15a and 2-Benzyl-6-butyl-4-methyl-7-(trimethylsilyl)isoindolin-1-one 16a

^{1}H NMR (600 MHz, DMSO-d$_6$)

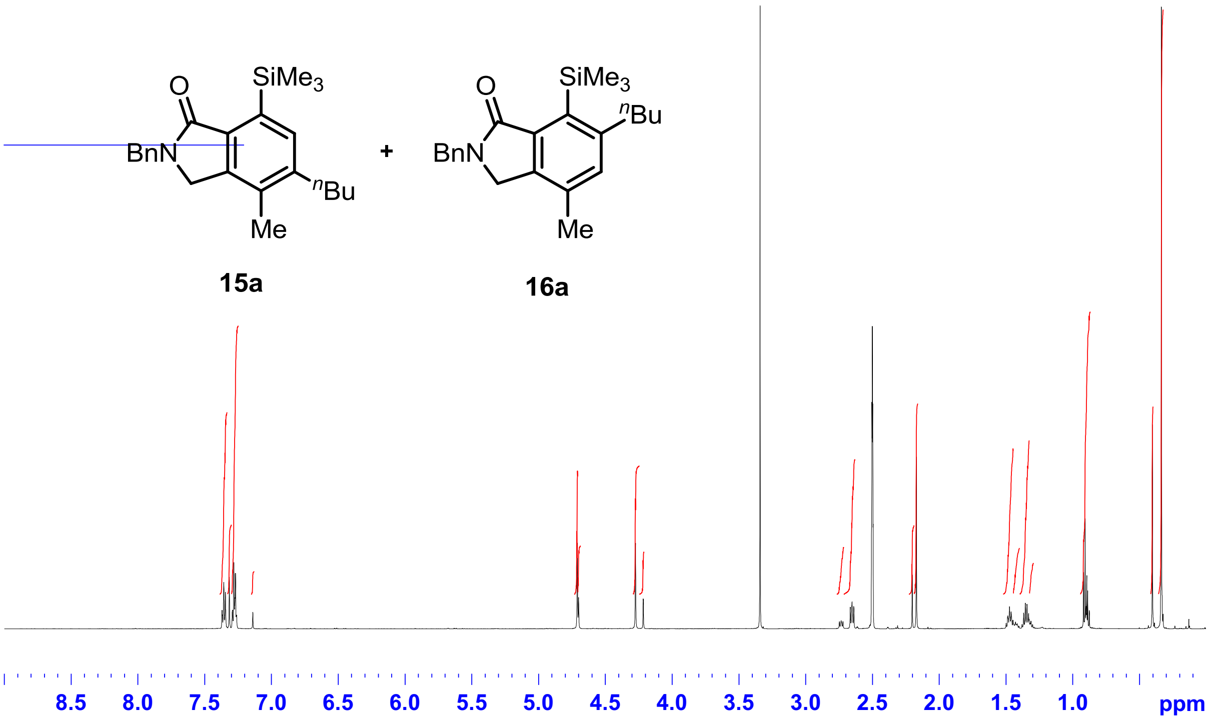

^{13}C NMR (150 MHz, DMSO-d$_6$)

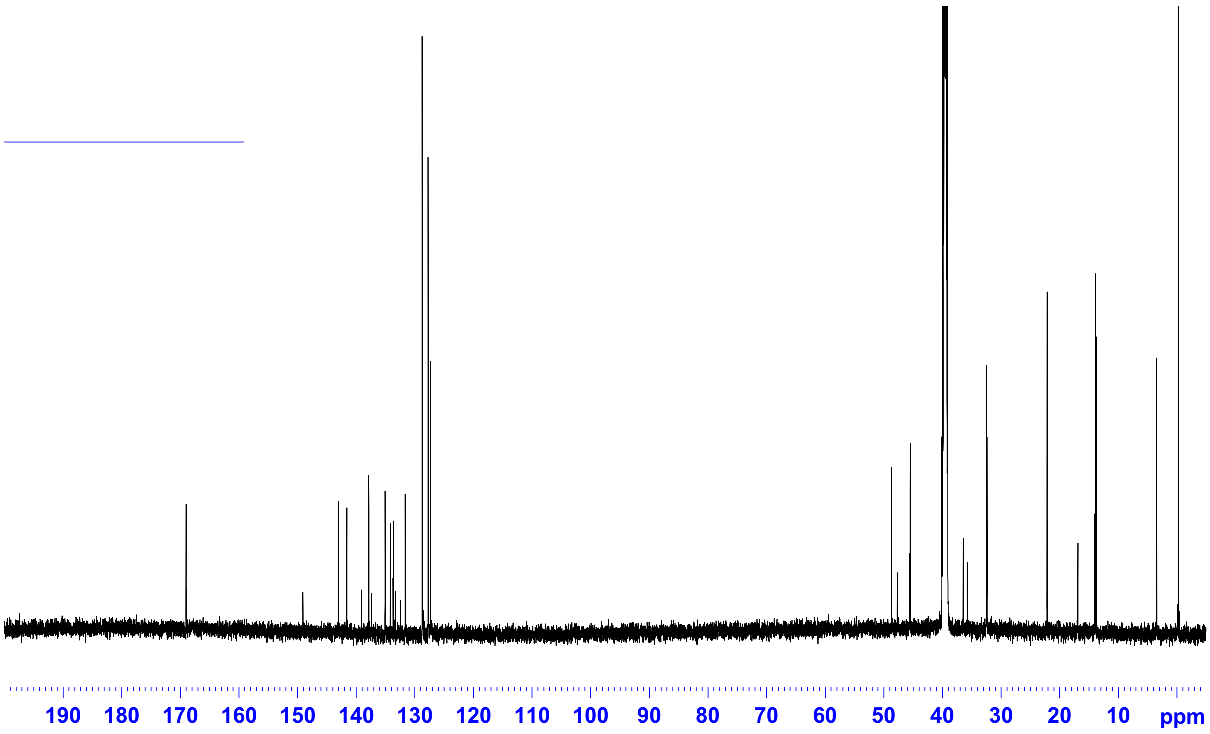

2-Benzyl-5-butyl-4-methyl-7-(trimethylsilyl)isoindolin-1-one 15a

^{1}H NMR (600 MHz, DMSO-d$_6$)

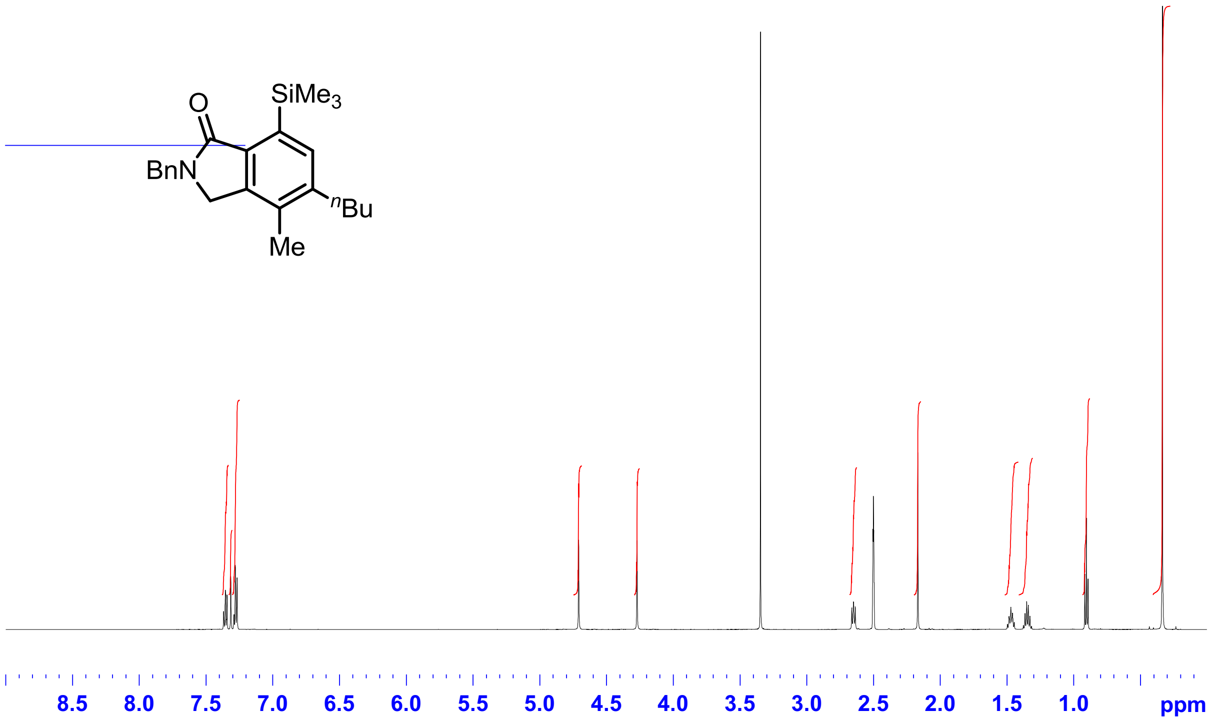

NOESY (600 MHz, DMSO-d$_6$)

δ 7.31

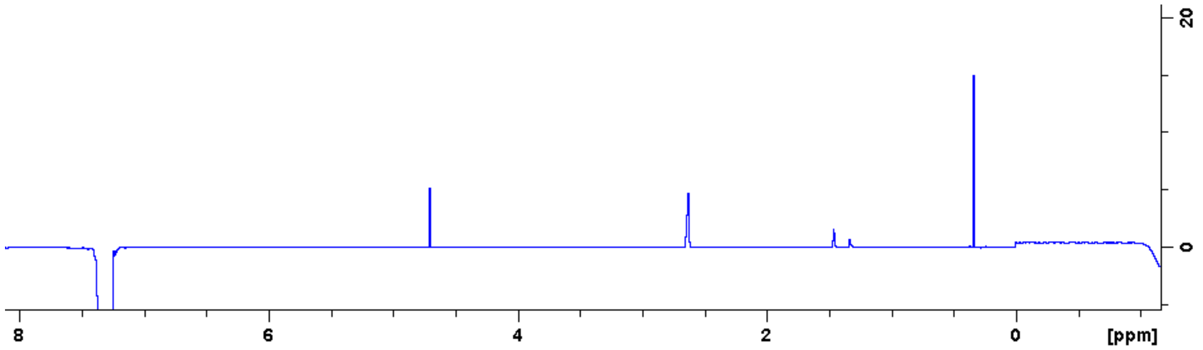

δ 2.63

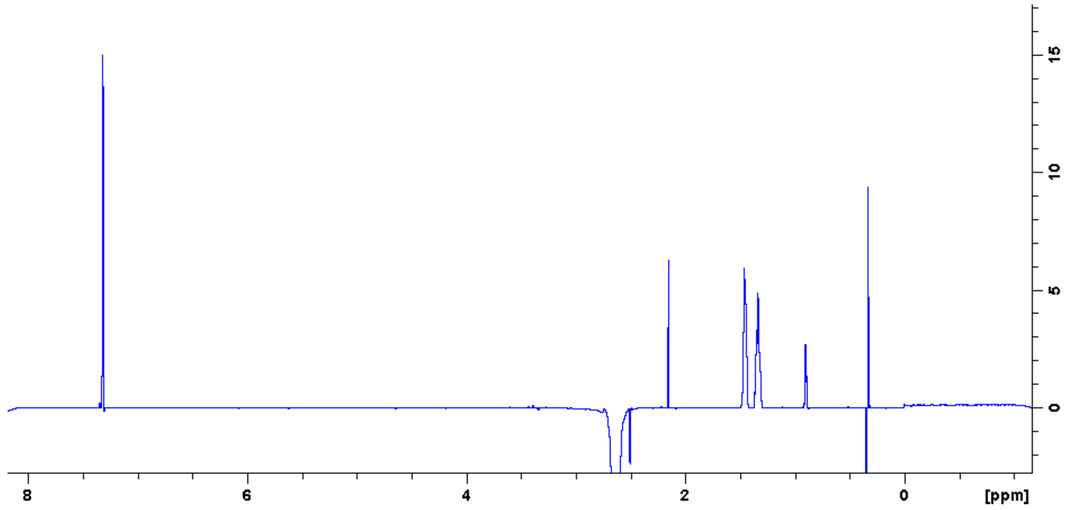

δ 2.15

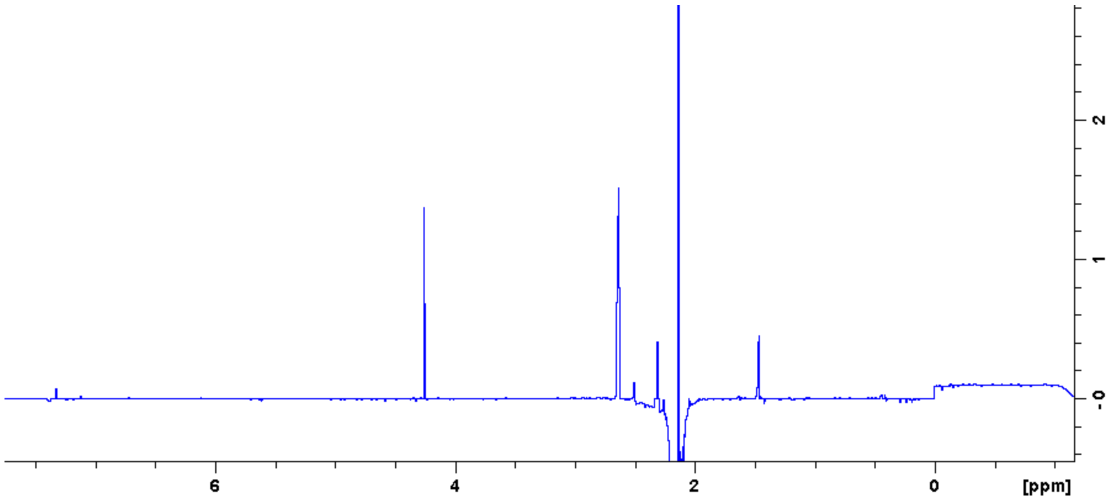

^{13}C NMR (150 MHz, DMSO-d_6)

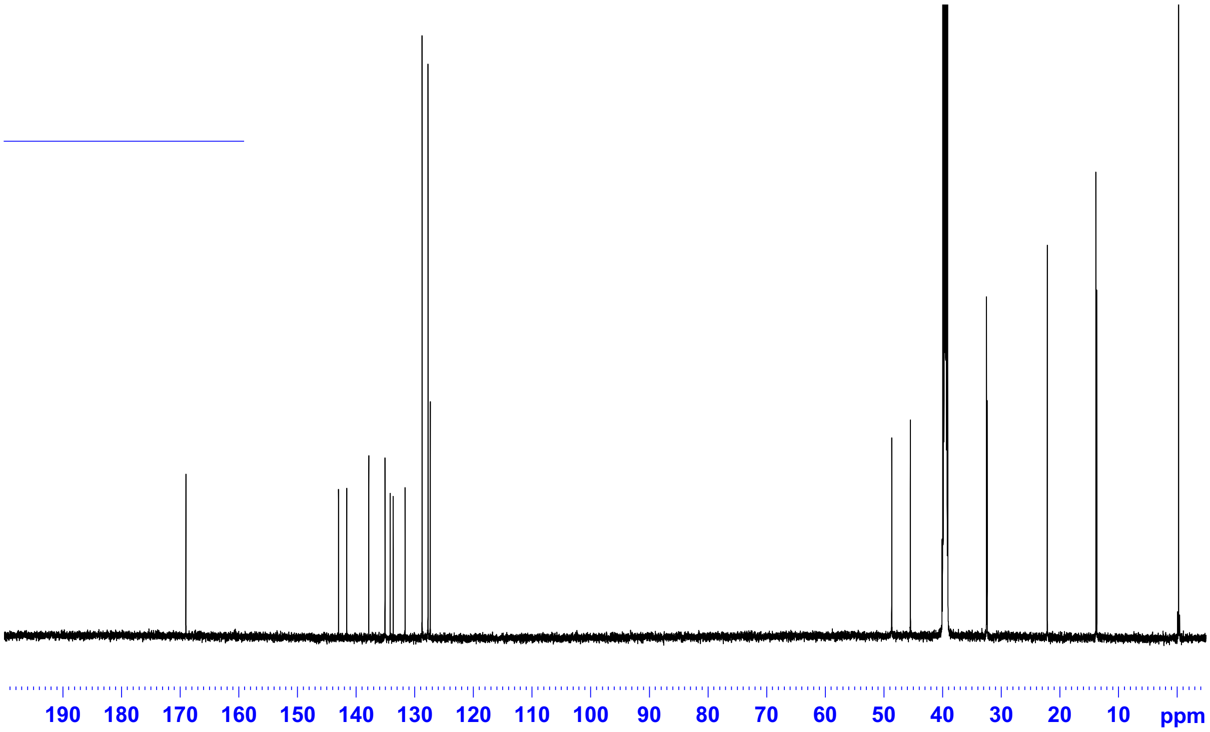

2-Benzyl-5-butyl-4-ethyl-7-(trimethylsilyl)isoindolin-1-one 15b and 2-Benzyl-6-butyl-4-ethyl-7-(trimethylsilyl)isoindolin-1-one 16b

^{1}H NMR (600 MHz, DMSO-d$_6$)

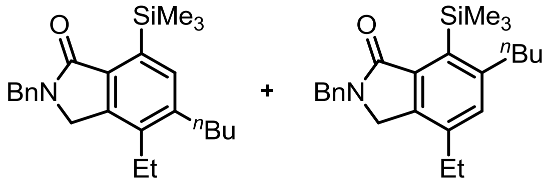

15b **16b**

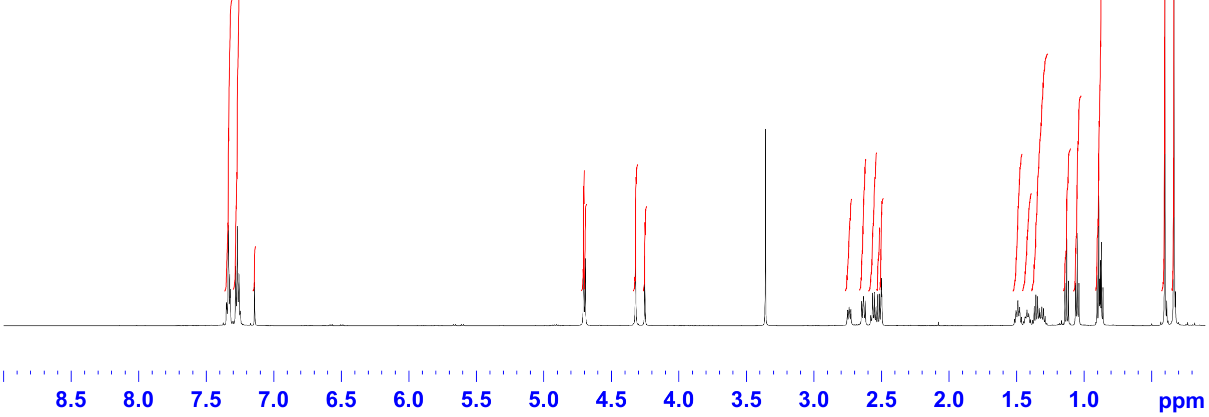

^{13}C NMR (150 MHz, DMSO-d$_6$)

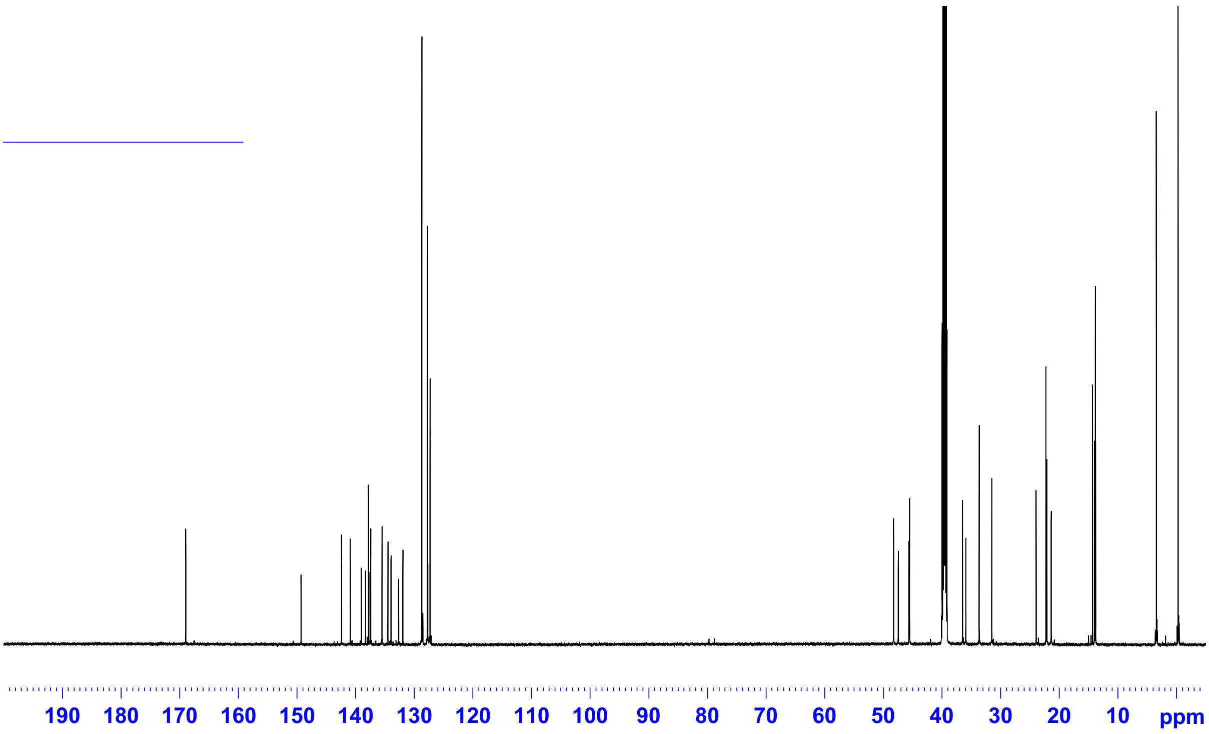

2-benzyl-5-butyl-4-ethyl-7-(trimethylsilyl)isoindolin-1-one 15b

^{1}H NMR (600 MHz, DMSO-d_6)

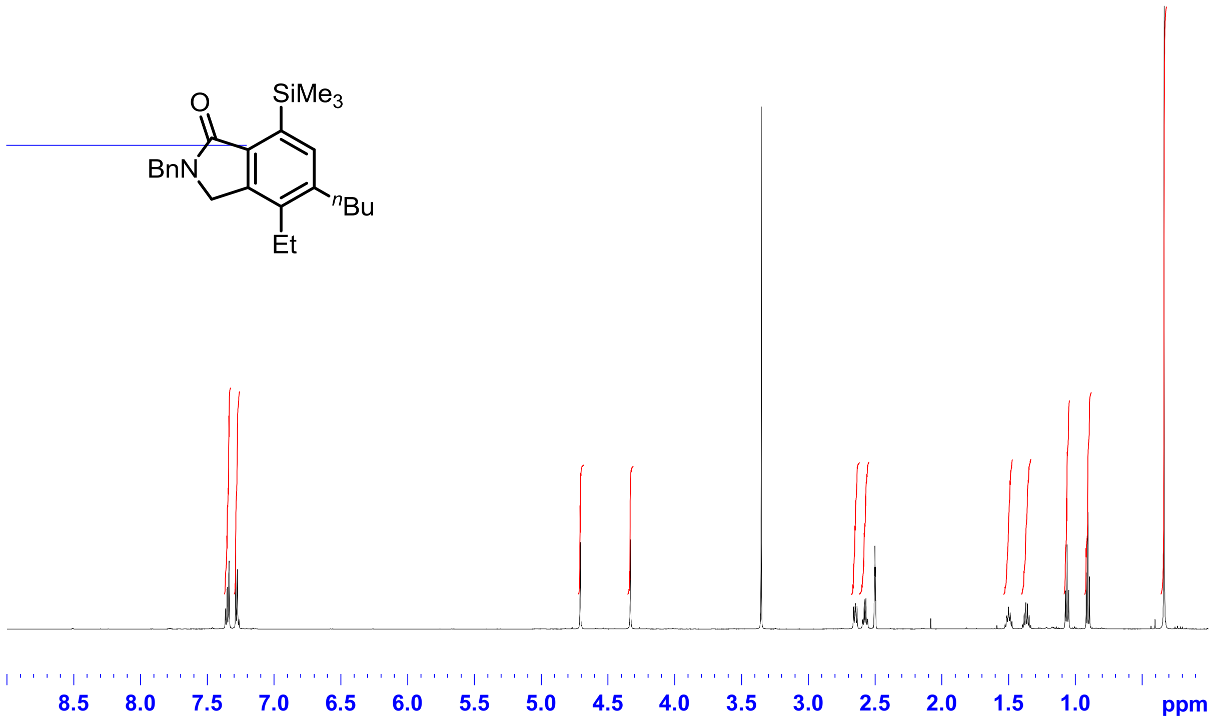

NOESY (600 MHz, DMSO-d_6)

δ 2.65

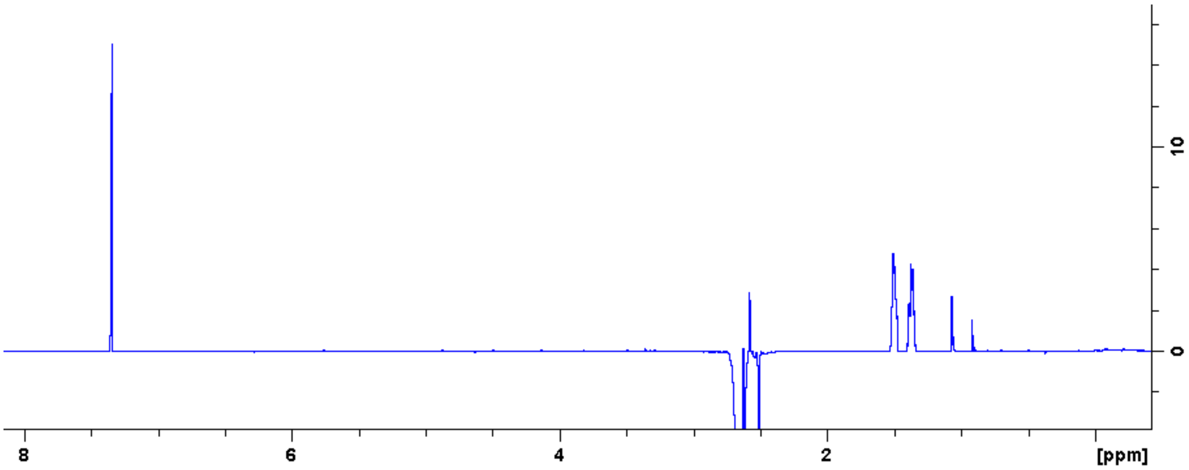

δ 2.57

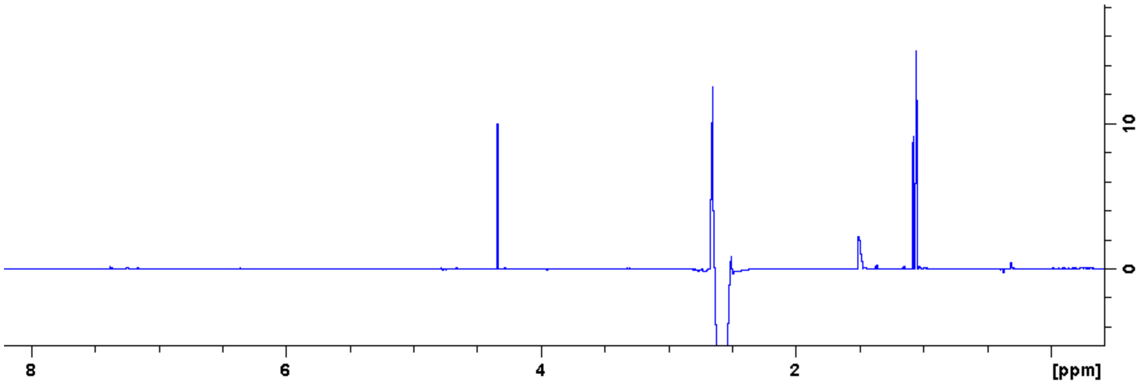

^{13}C NMR (150 MHz, DMSO-d_6)

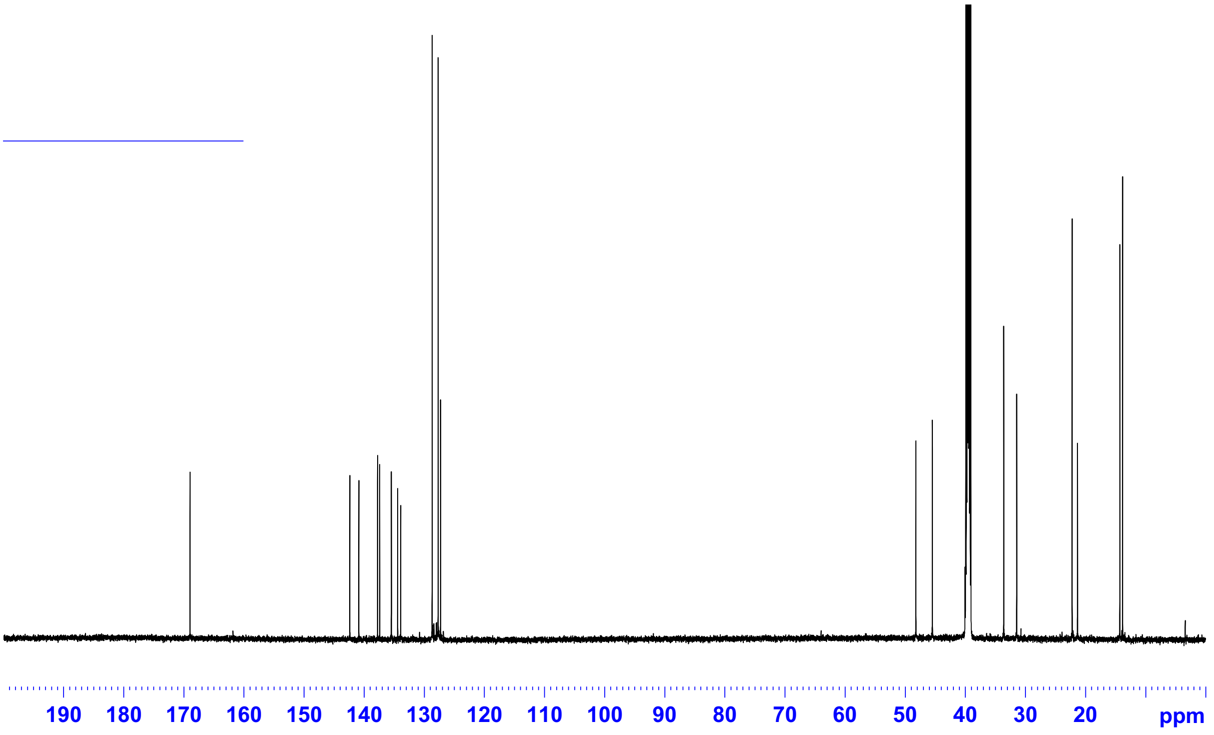

2-Benzyl-4-ethyl-5-(*o*-tolyl)-7-(trimethylsilyl)isoindolin-1-one 15c and 2-Benzyl-4-ethyl-6-(*o*-tolyl)-7-(trimethylsilyl)isoindolin-1-one 16c

^{1}H NMR (600 MHz, DMSO-d_6)

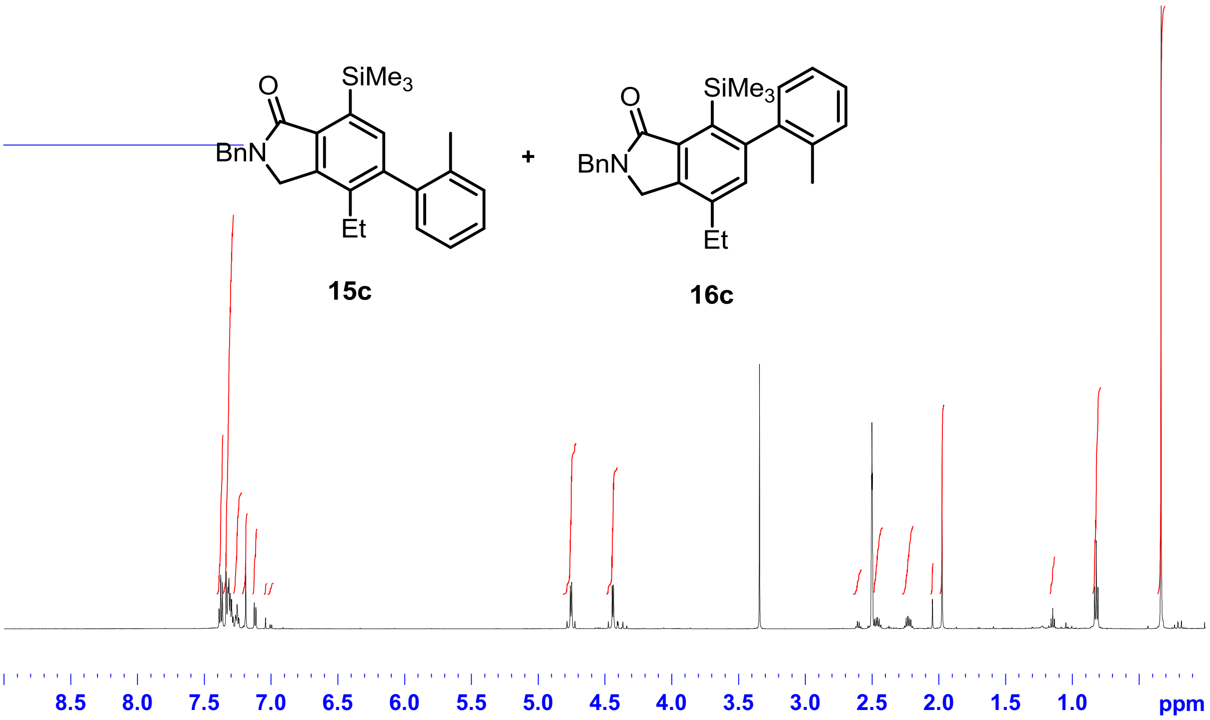

NOESY (600 MHz, DMSO-d_6)

δ 7.19

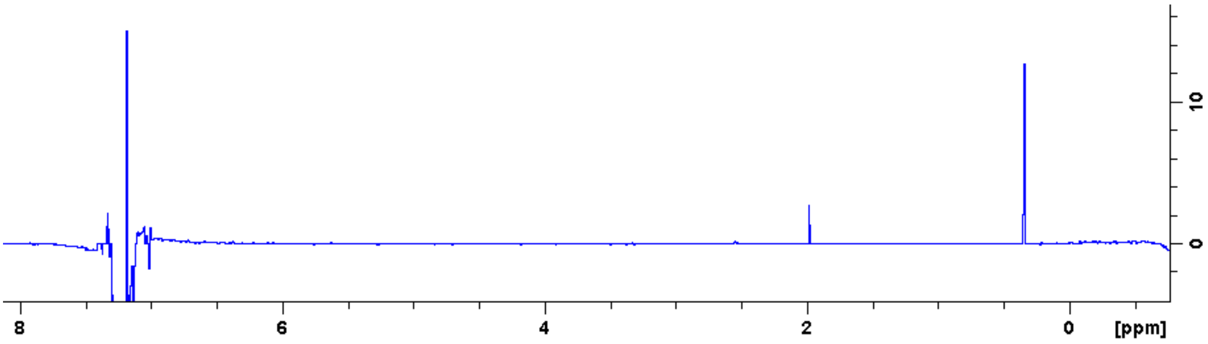

δ 0.36

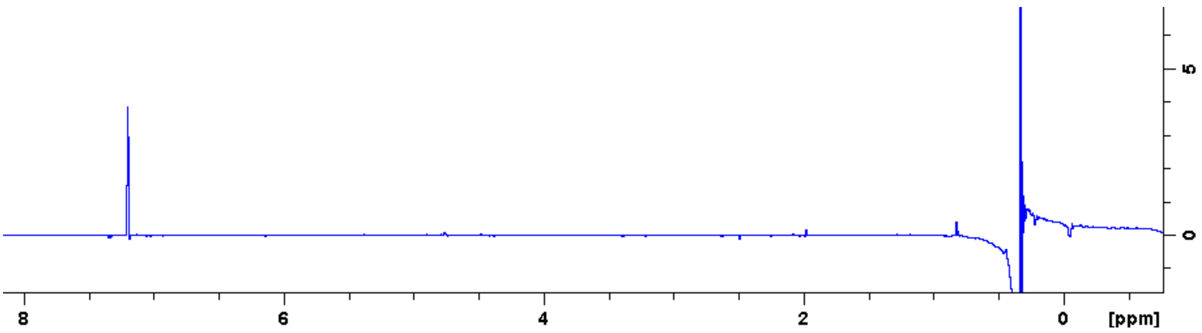

^{13}C NMR (150 MHz, DMSO-d_6)

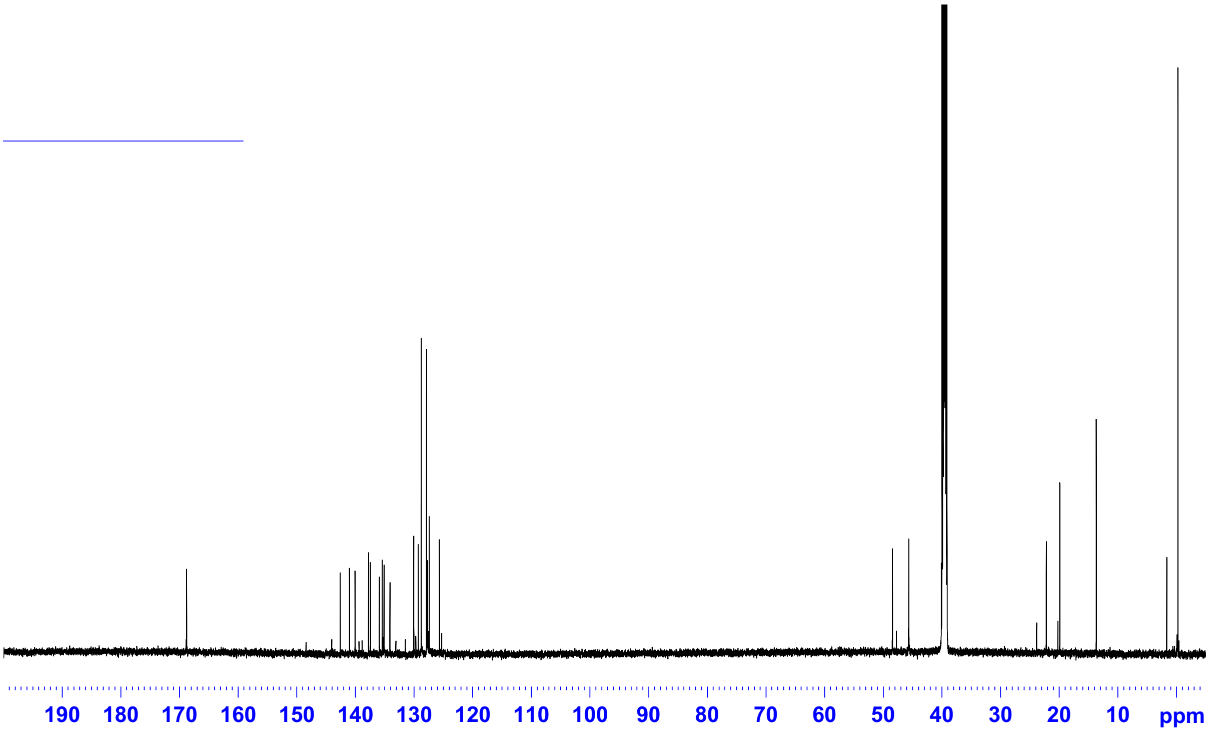

2-Benzyl-5-butyl-7-methylisoindolin-1-one 15d

^{1}H NMR (600 MHz, DMSO-d$_6$)

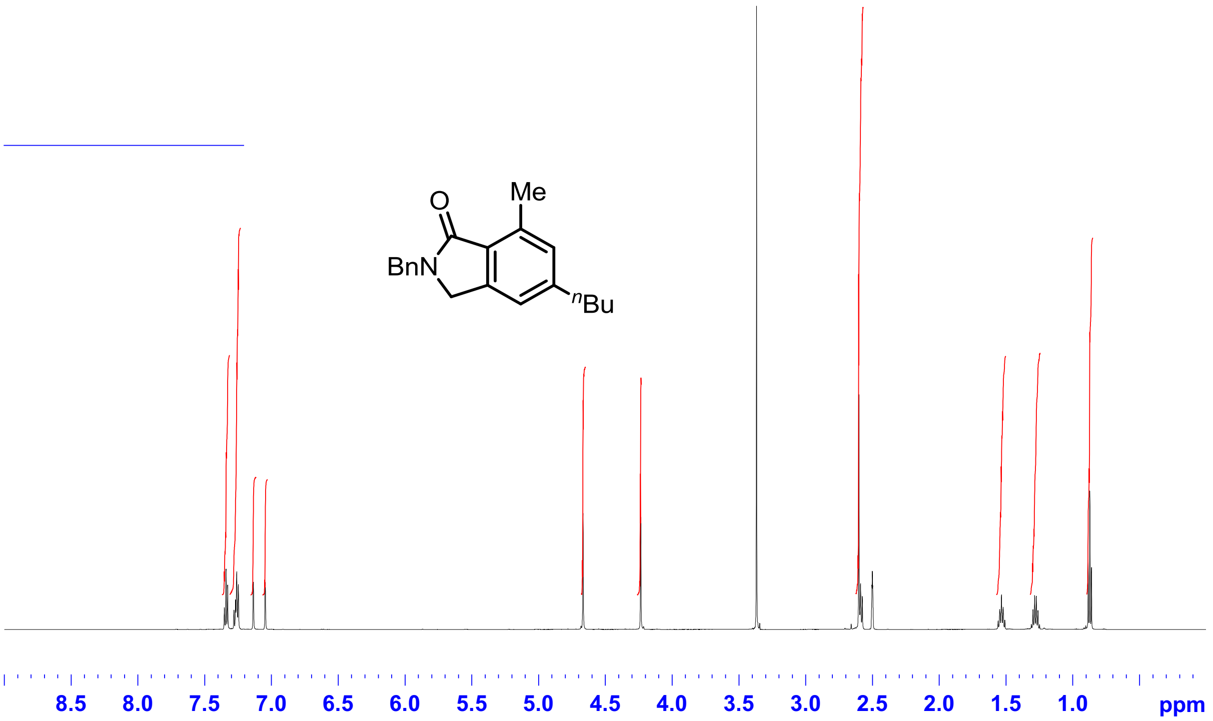

^{13}C NMR (150 MHz, DMSO-d$_6$)

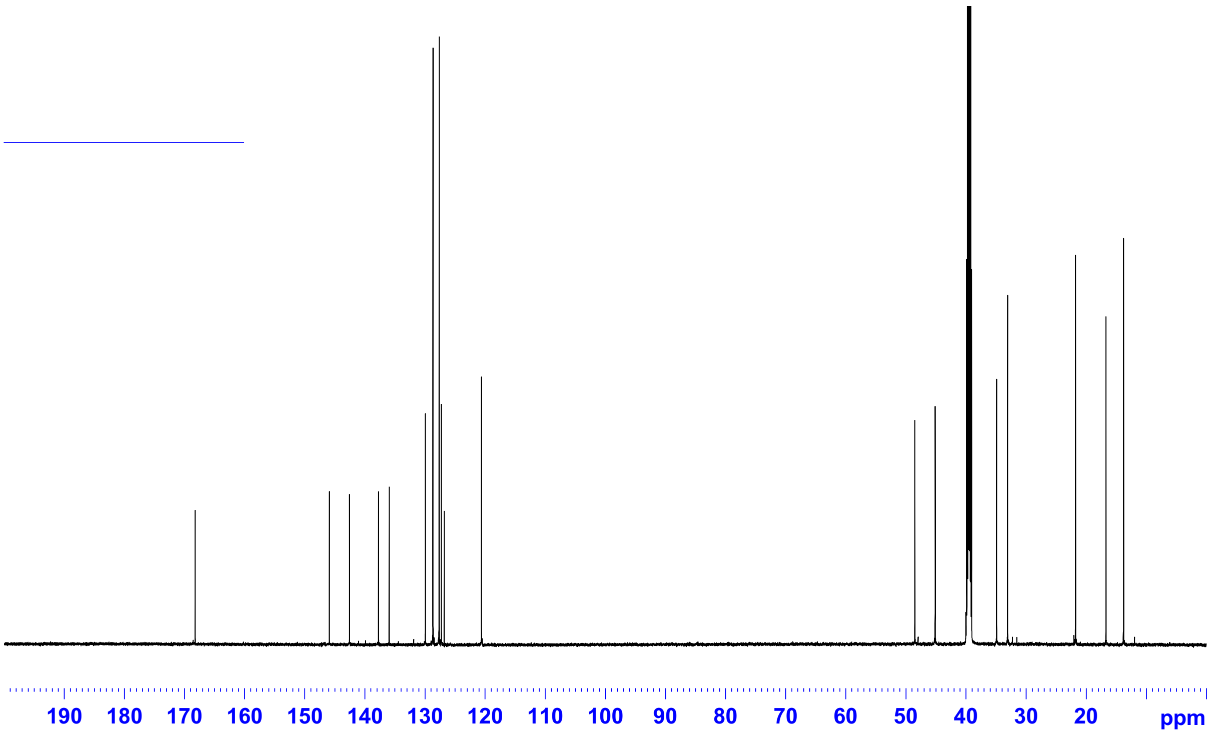

2-Benzyl-7-methyl-5-(*o*-tolyl)isoindolin-1-one 15e

^{1}H NMR (600 MHz, DMSO-d$_6$)

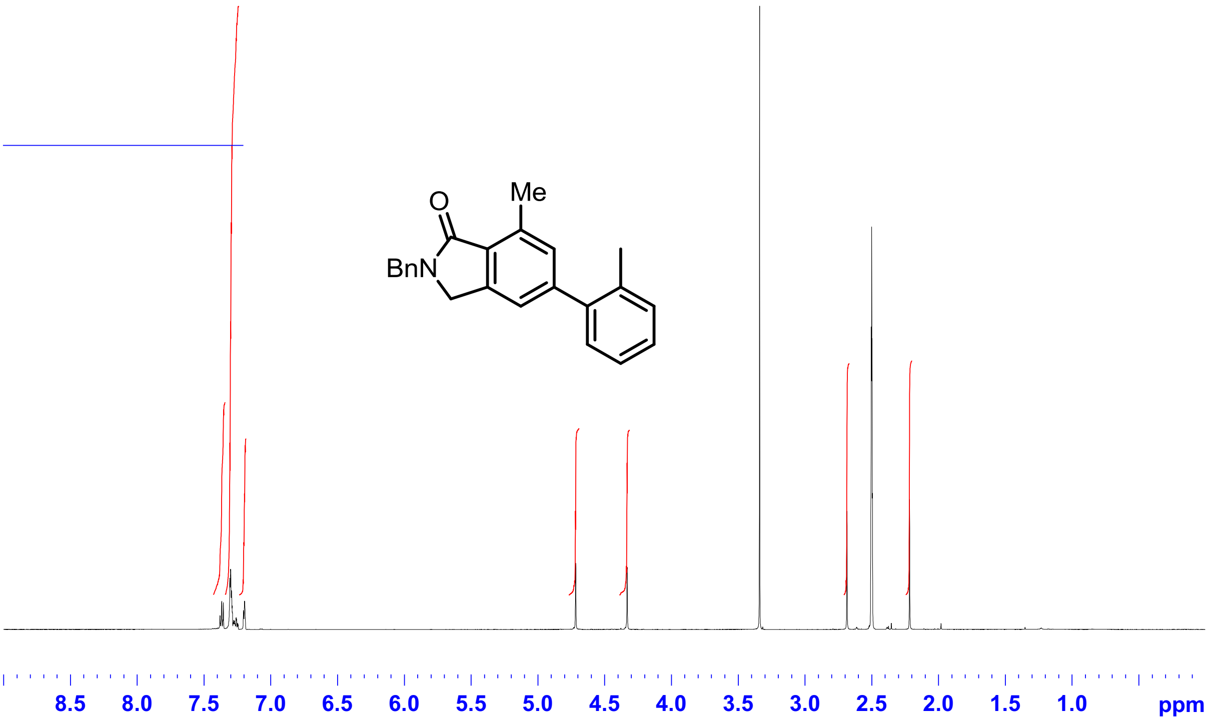

^{13}C NMR (150 MHz, DMSO-d$_6$)

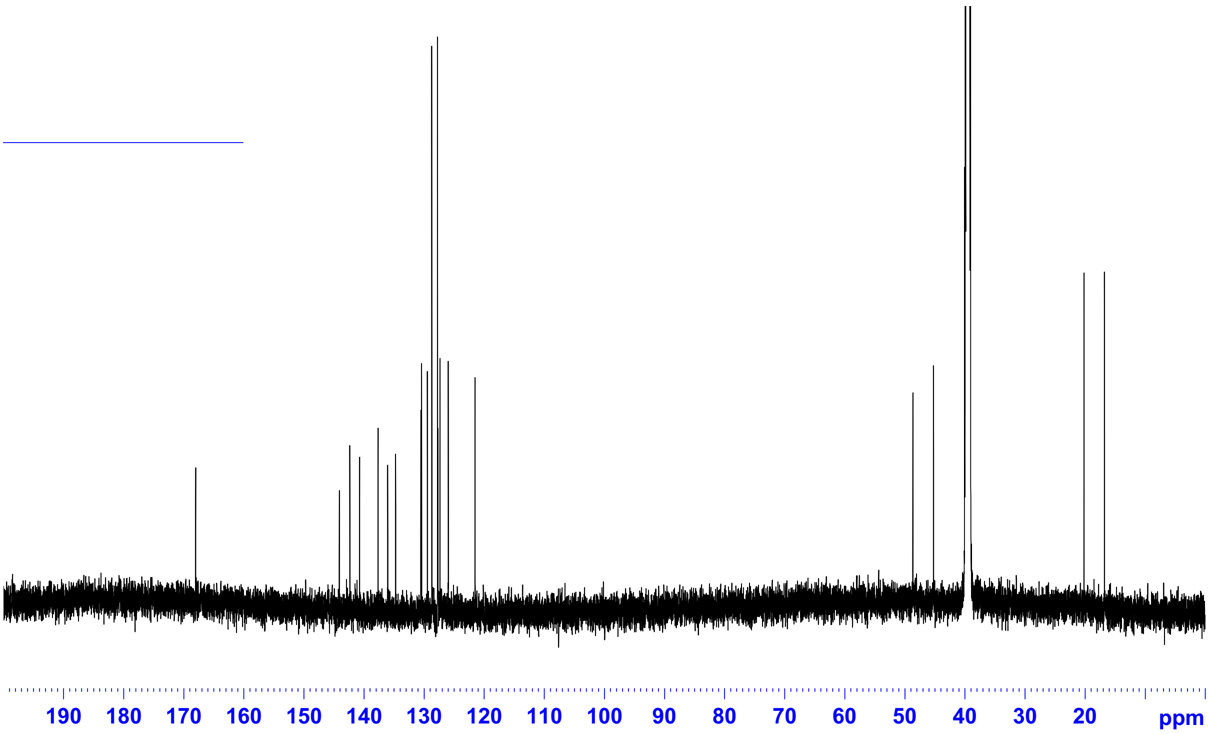

2-Benzyl-7-bromo-5-butylisoindolin-1-one 17

^{1}H NMR (600 MHz, DMSO-d_6)

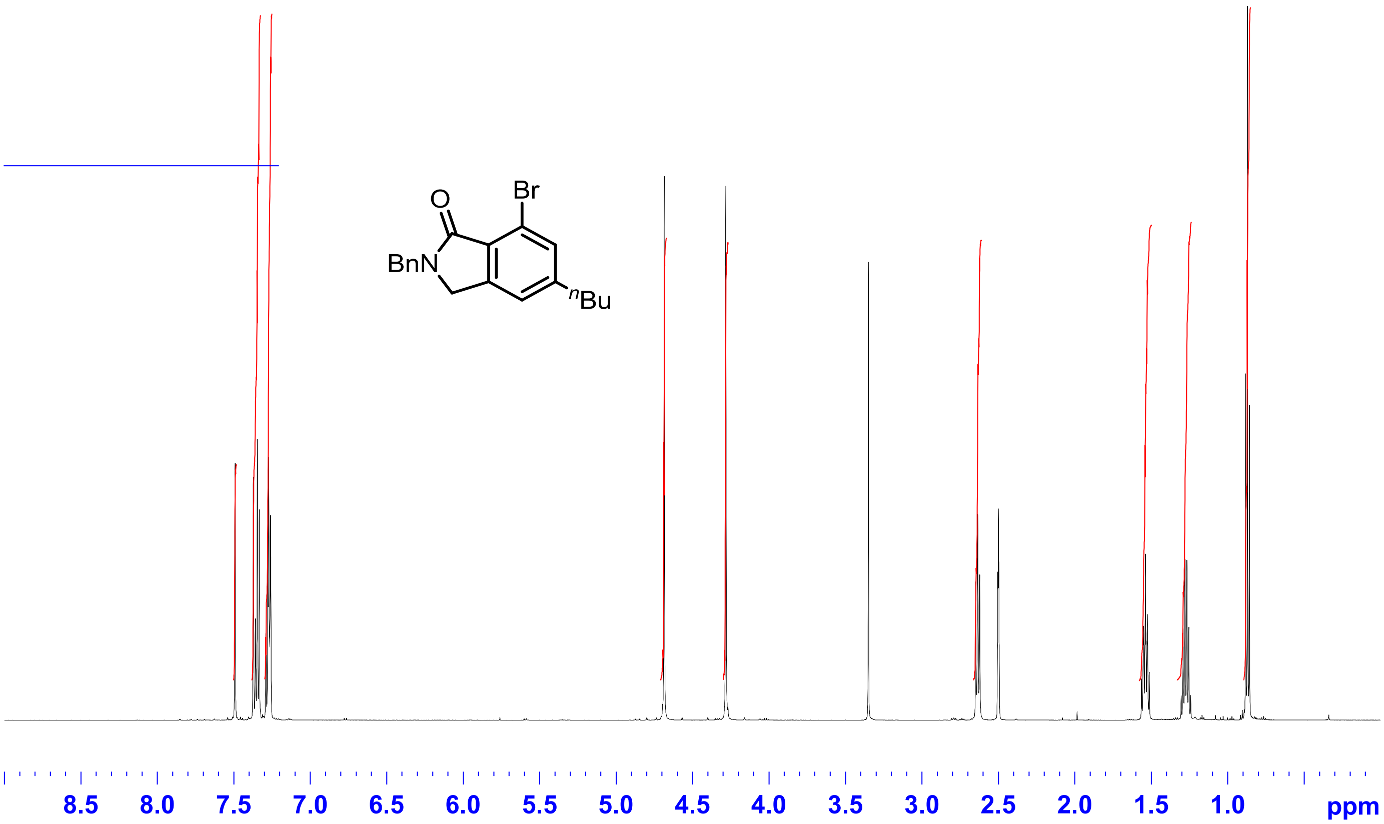

^{13}C NMR (600 MHz, DMSO-d_6)

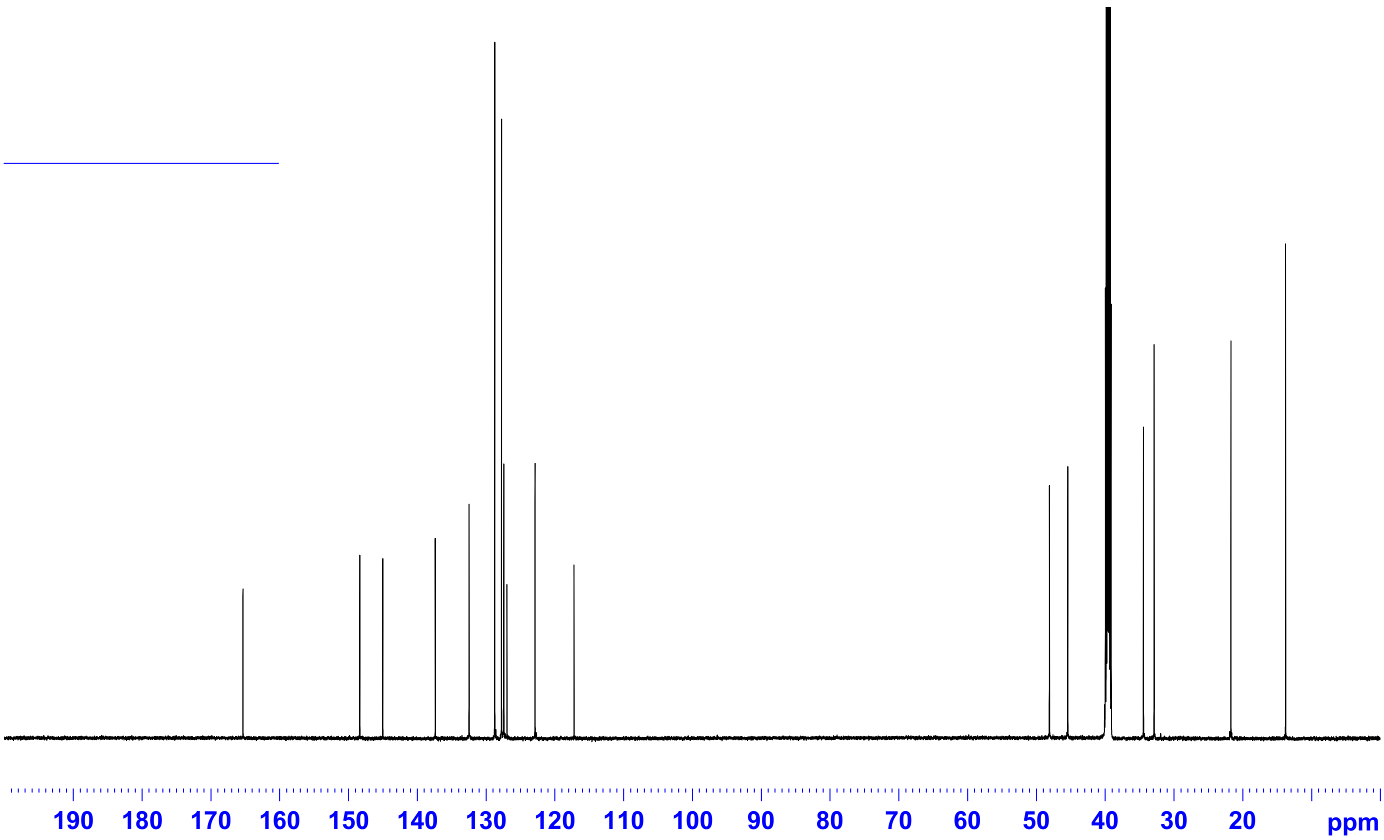

2-Benzyl-5-butyl-7-iodoisoindolin-1-one 18

^{1}H NMR (600 MHz, DMSO-d_6)

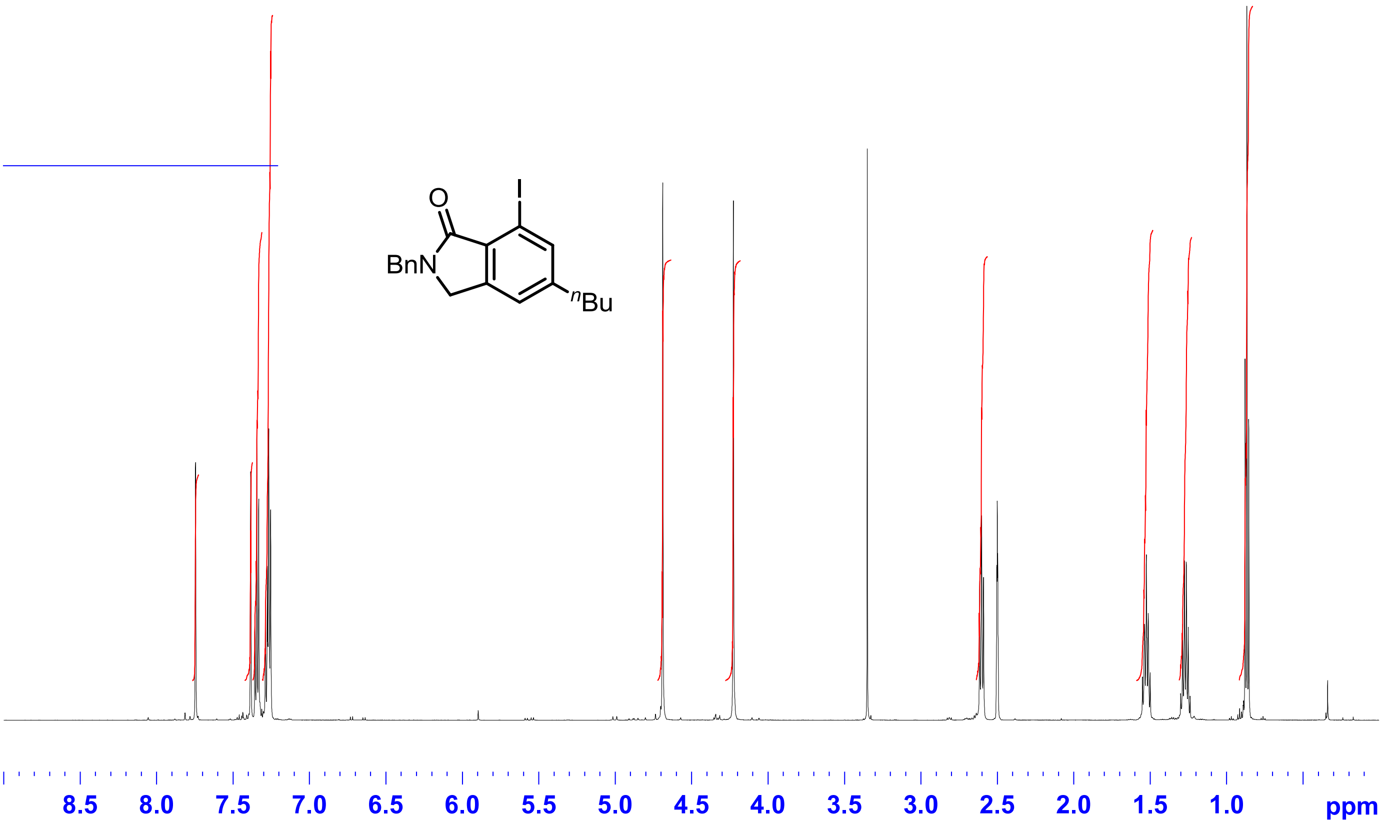

^{13}C NMR (600 MHz, DMSO-d_6)

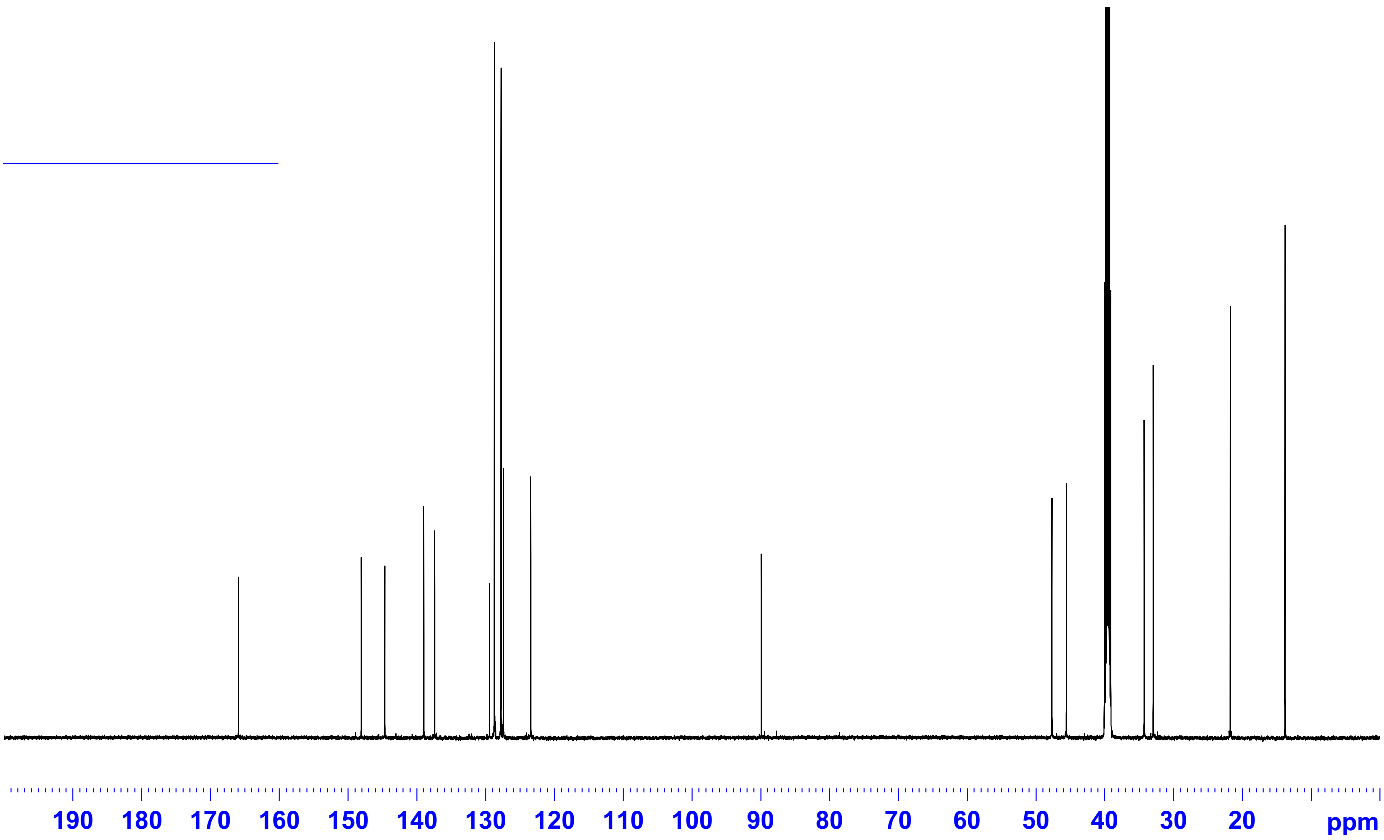

2-(*tert*-Butyl)-5-butyl-7-iodoisoindolin-1-one

^{1}H NMR (600 MHz, DMSO-d$_6$)

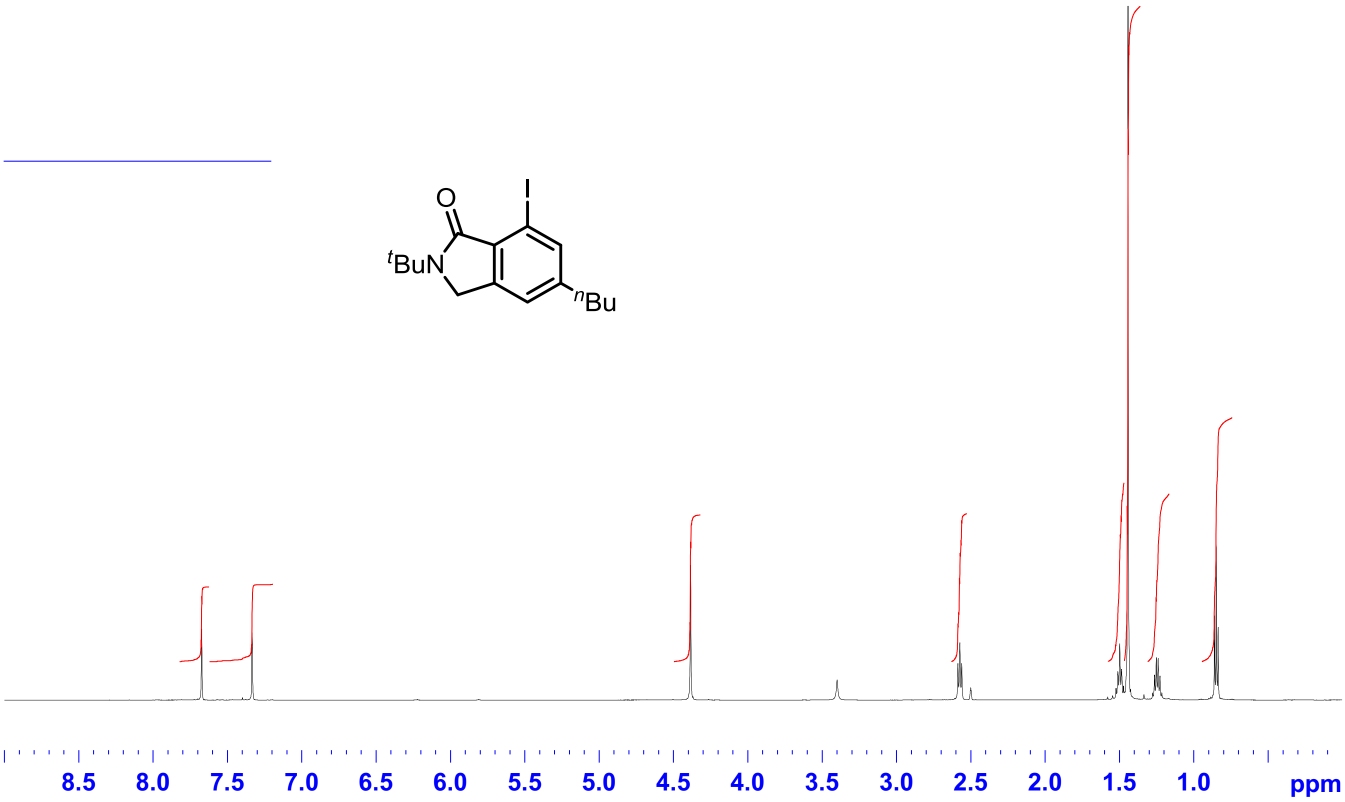

^{13}C NMR (600 MHz, DMSO-d$_6$)

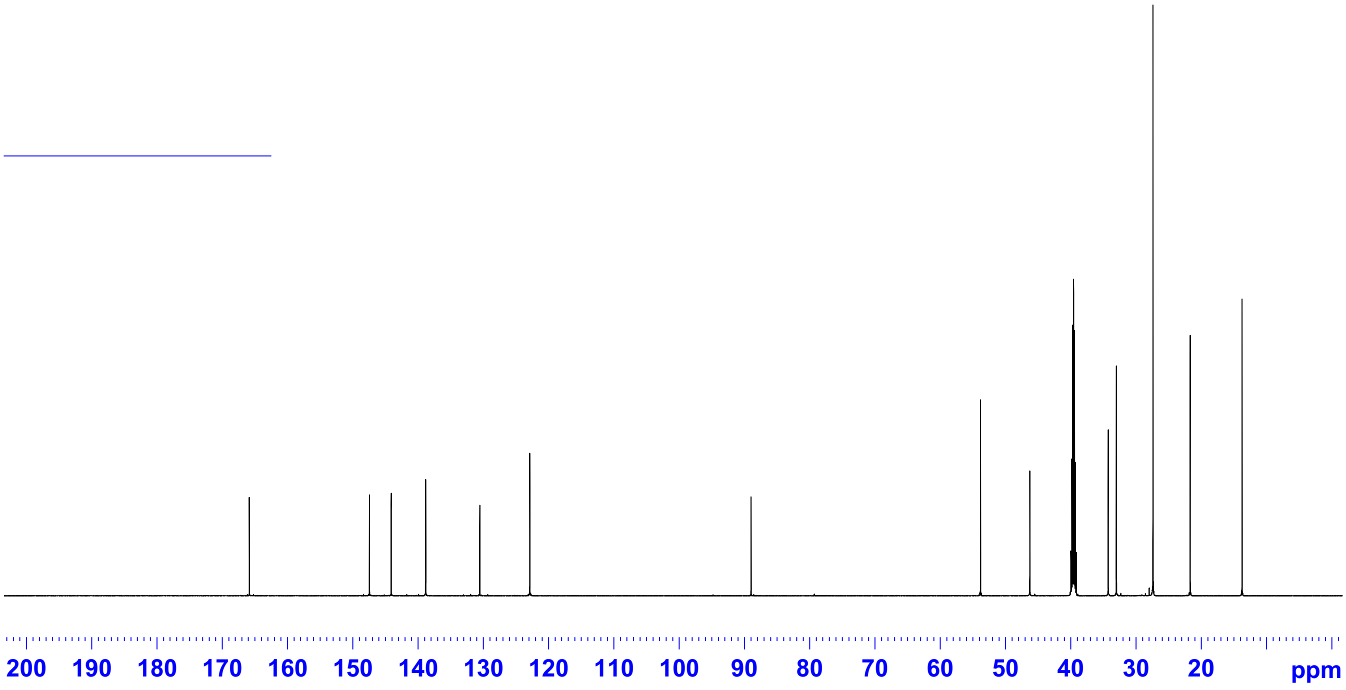

5-Butyl-7-iodoisoindolin-1-one 19

^{1}H NMR (600 MHz, DMSO-d$_6$)

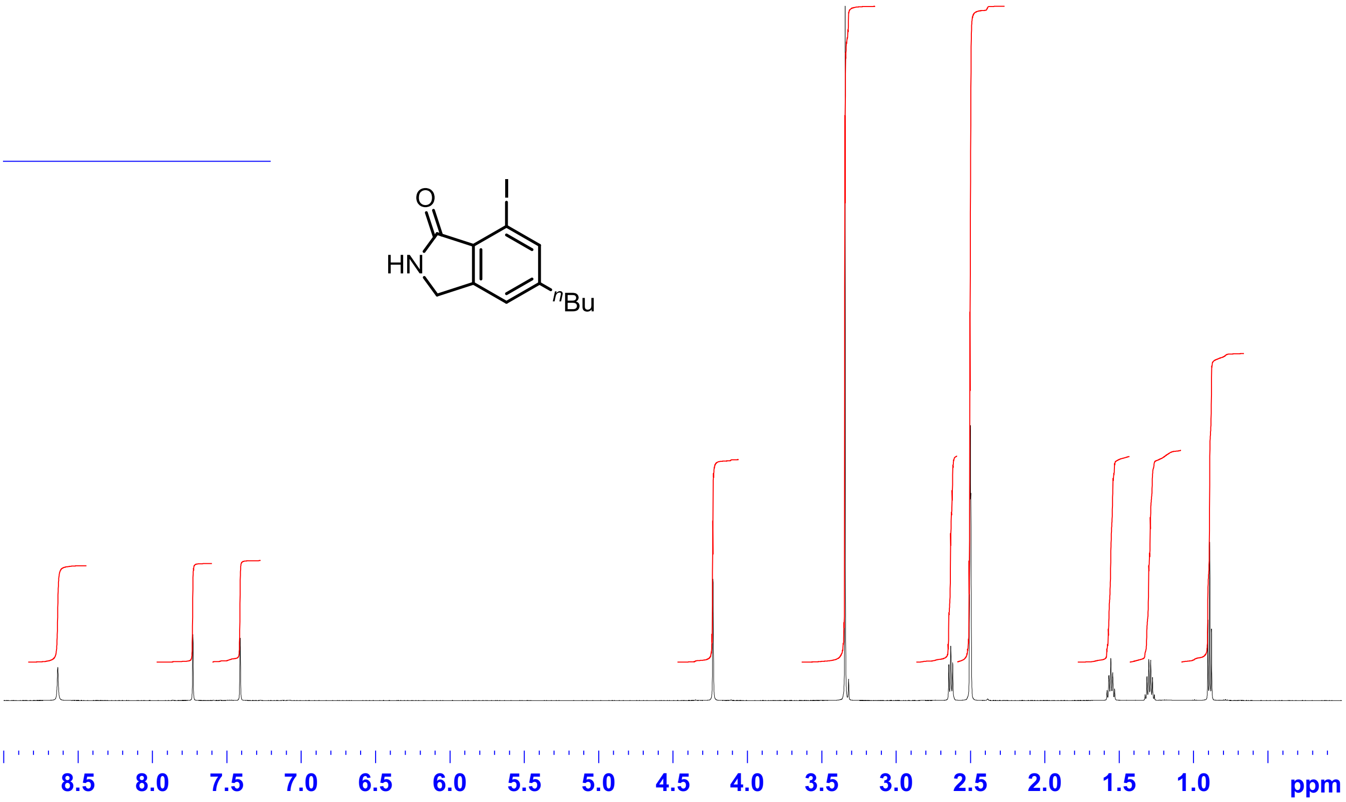

^{13}C NMR (600 MHz, DMSO-d$_6$)

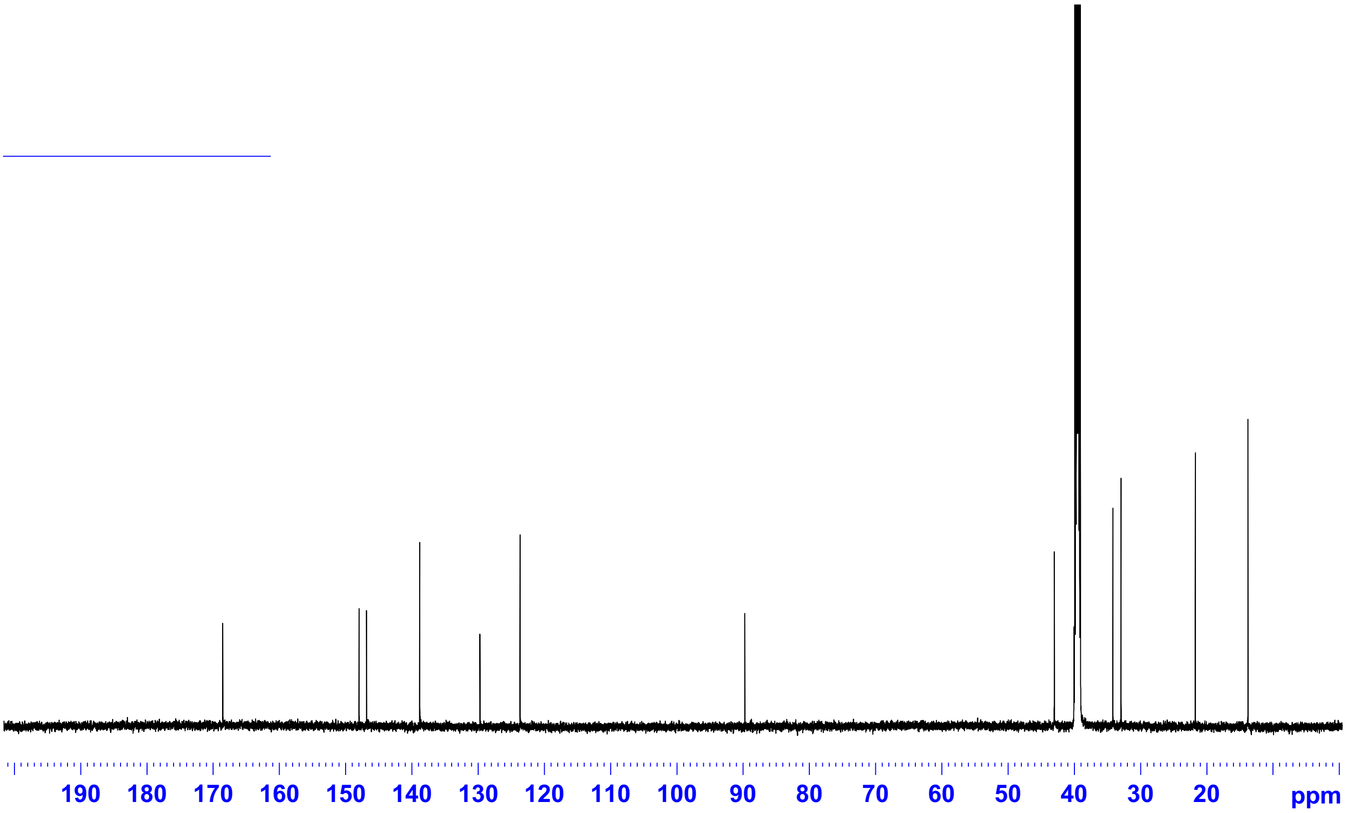

5-Butylisoindolin-1-one 20

^{1}H NMR (600 MHz, DMSO-d_6)

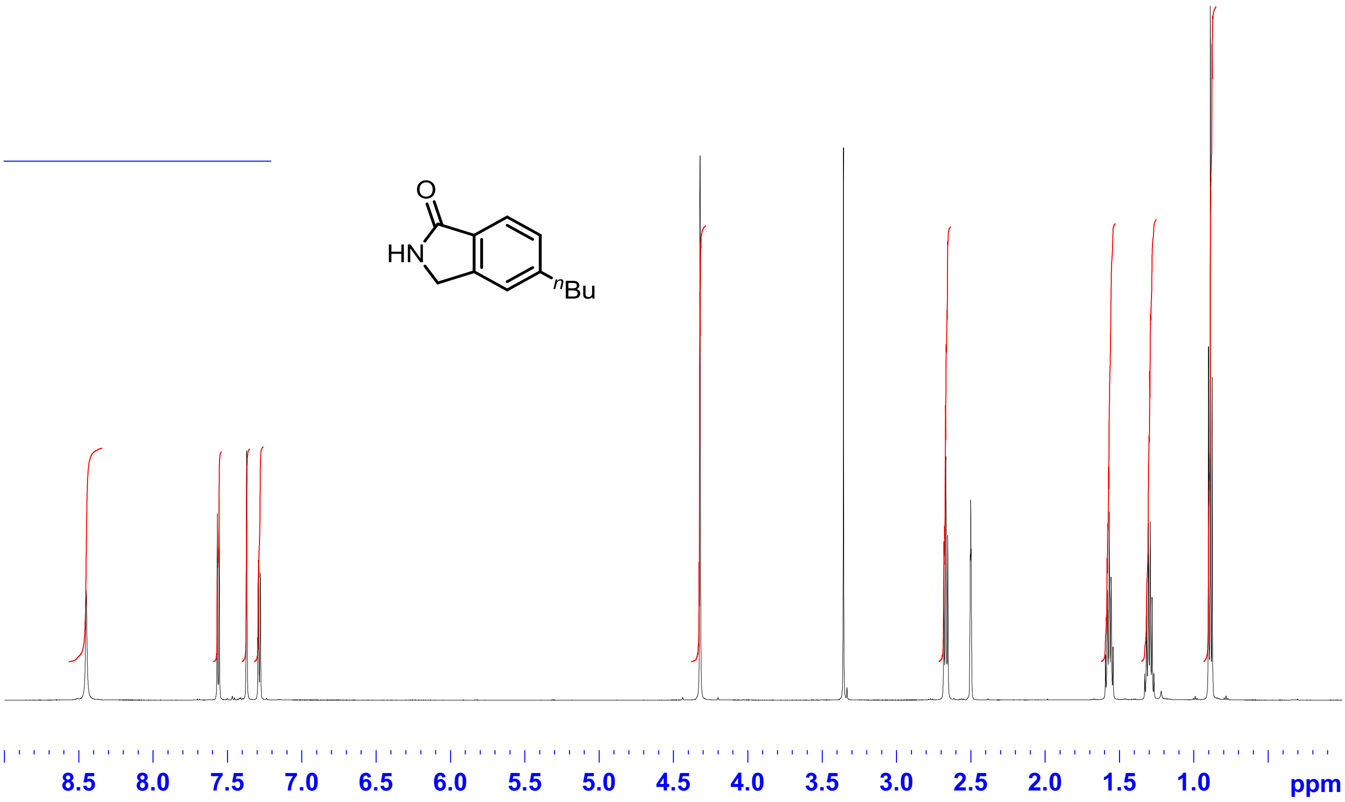

^{13}C NMR (600 MHz, DMSO-d_6)

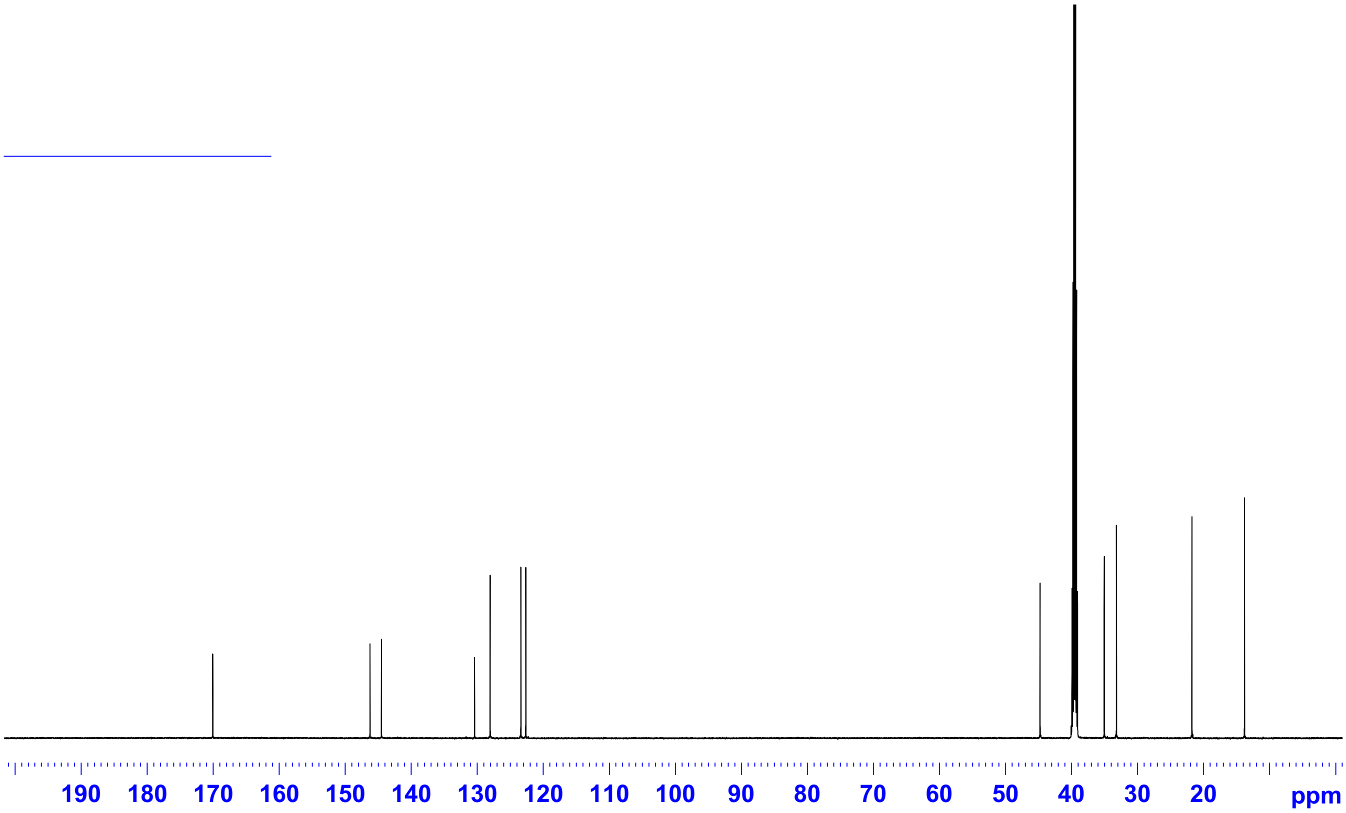

***tert*-Butyl 5-butyl-7-iodo-1-oxoisoindoline-2-carboxylate 21**

^{1}H NMR (600 MHz, DMSO-d$_6$)

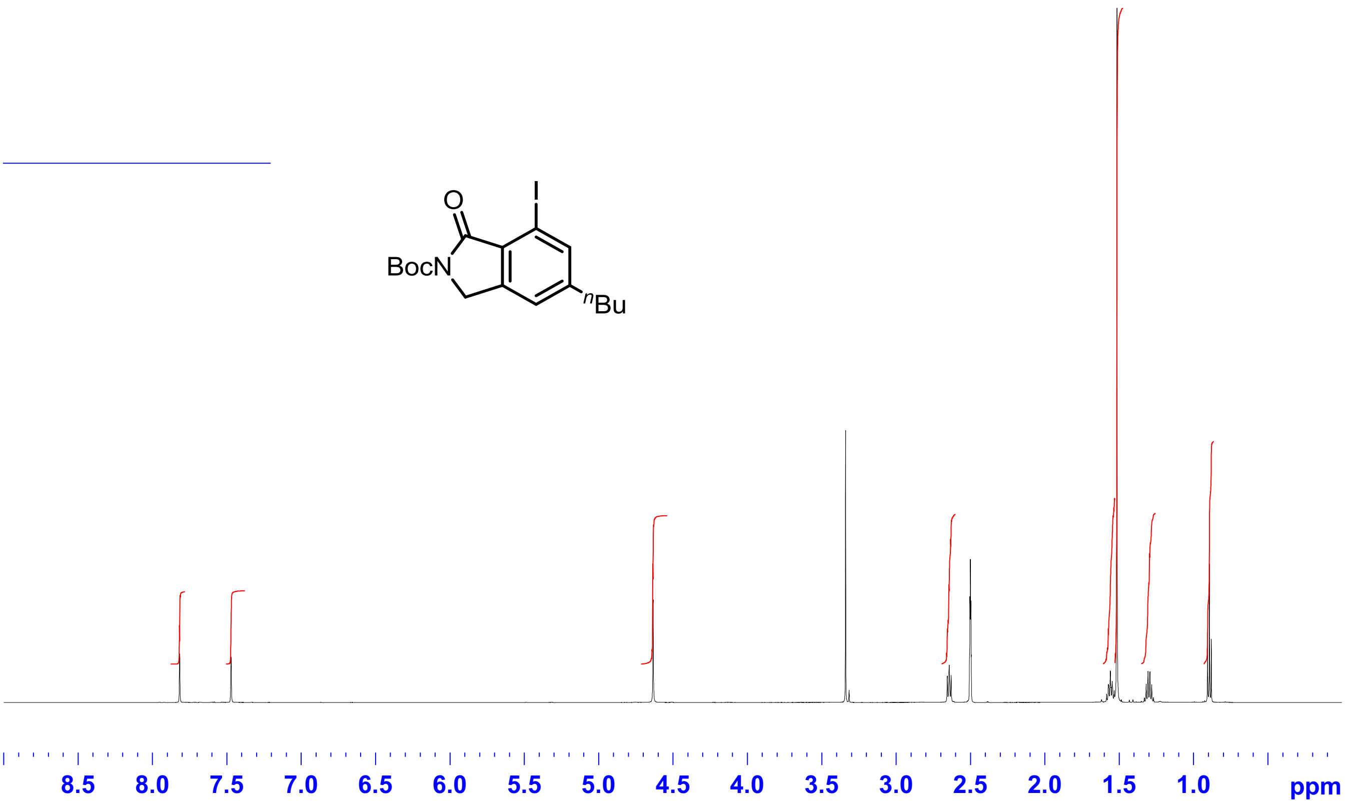

^{13}C NMR (600 MHz, DMSO-d$_6$)

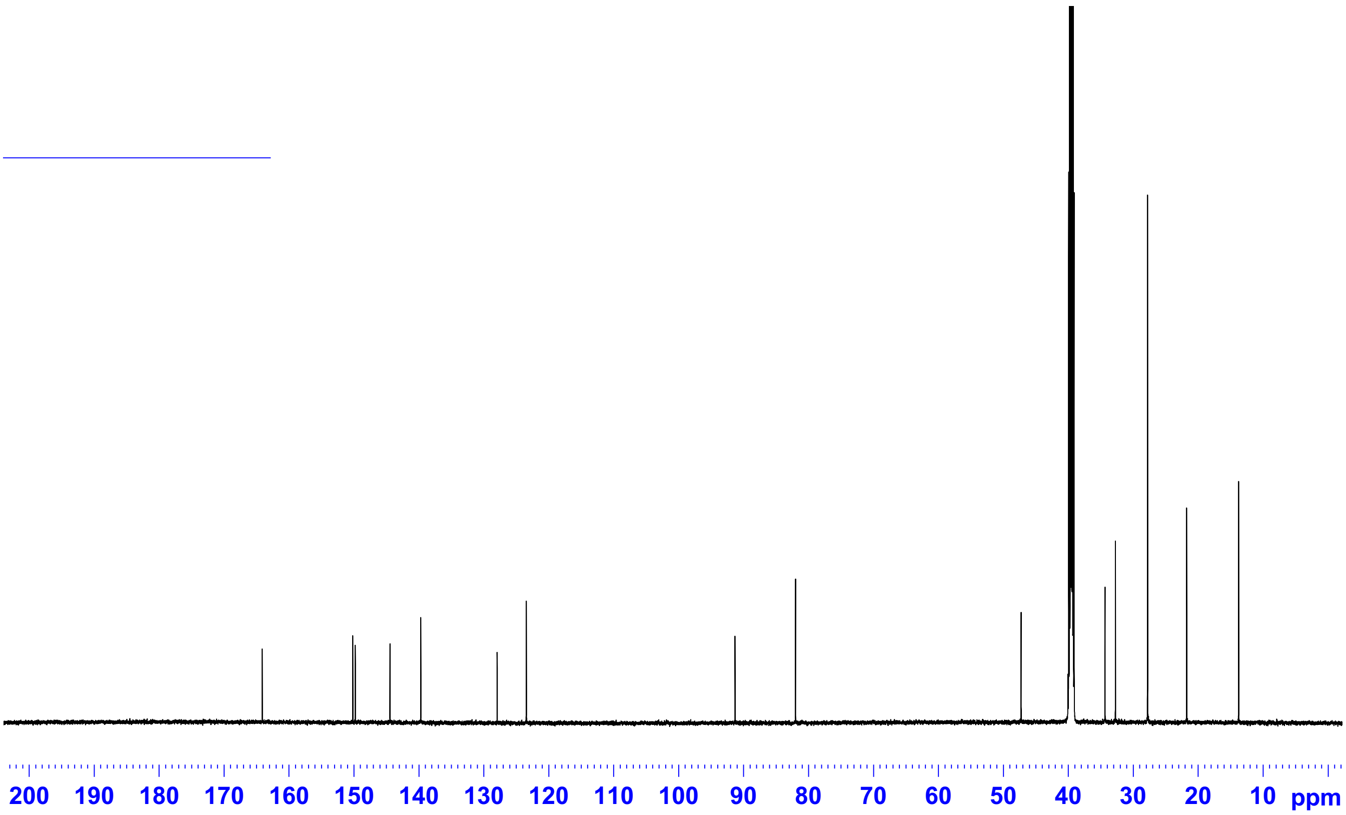

***tert*-Butyl 5-butyl-2-(hydroxymethyl)-3-iodobenzylcarbamate 22**

^{1}H NMR (600 MHz, DMSO-d$_6$)

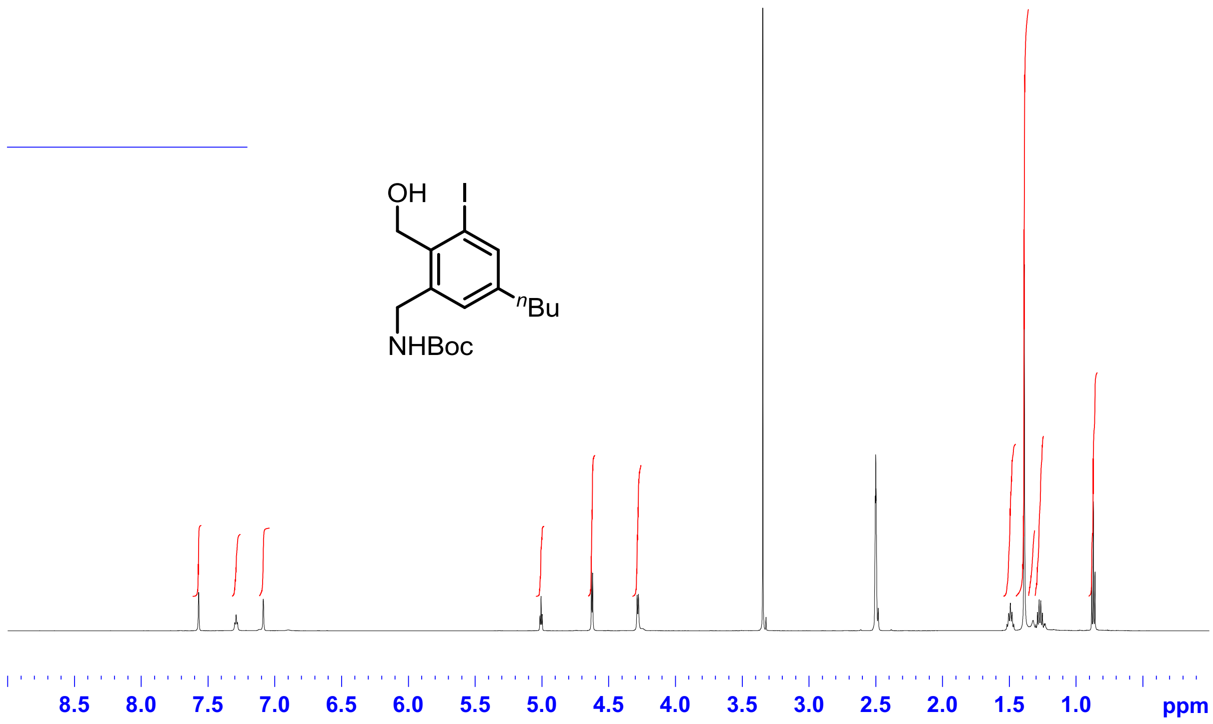

^{13}C NMR (600 MHz, DMSO-d$_6$)

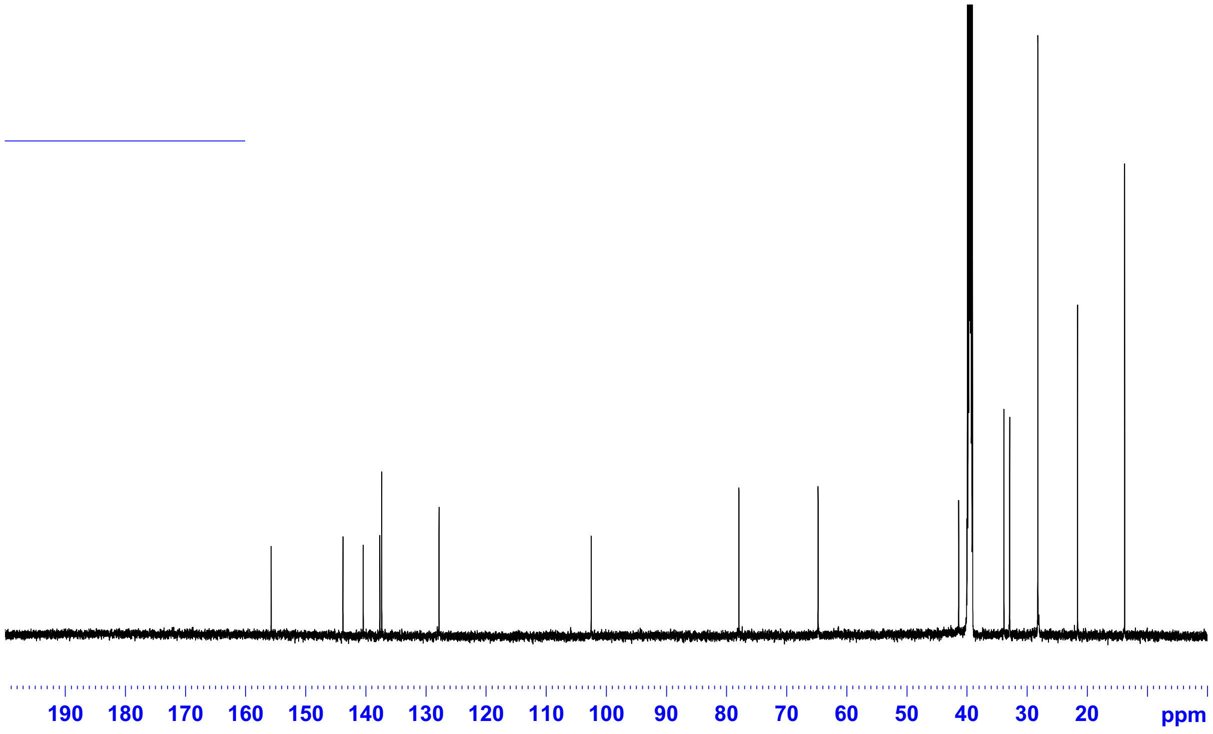

***tert*-Butyl 5-butyl-1-hydroxy-7-iodoisoindoline-2-carboxylate 23**

^{1}H NMR (600 MHz, DMSO-d$_6$)

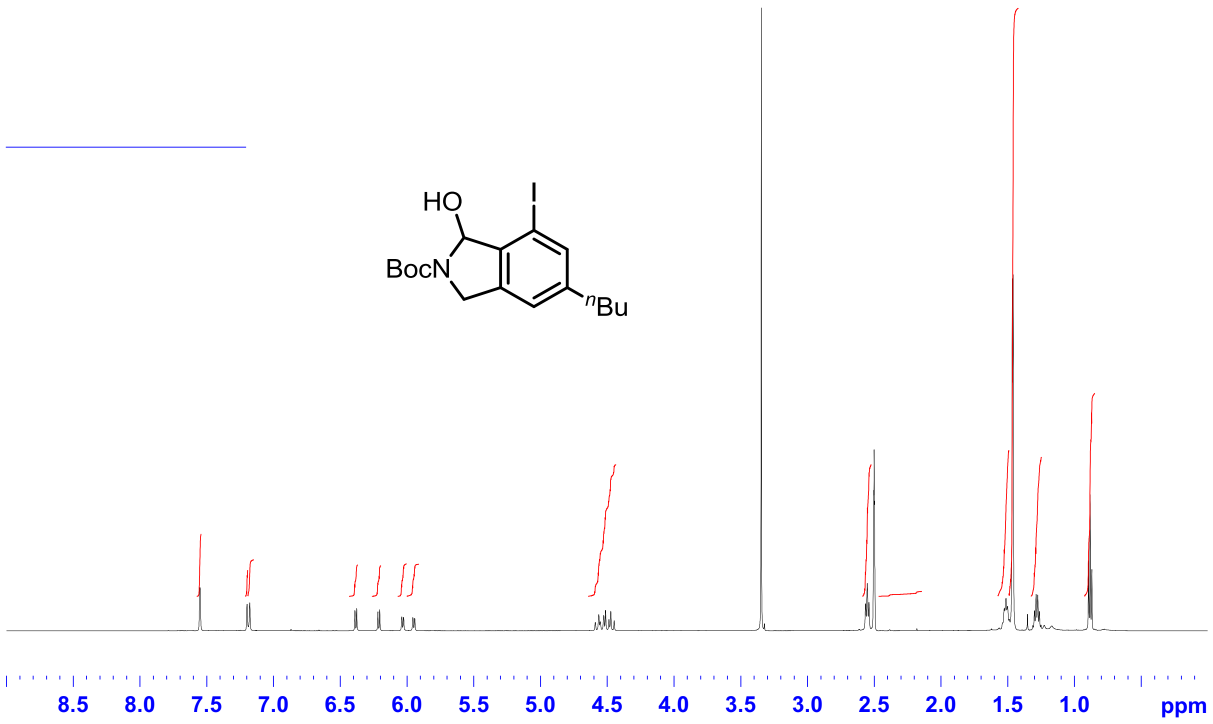

^{13}C NMR (600 MHz, DMSO-d$_6$)

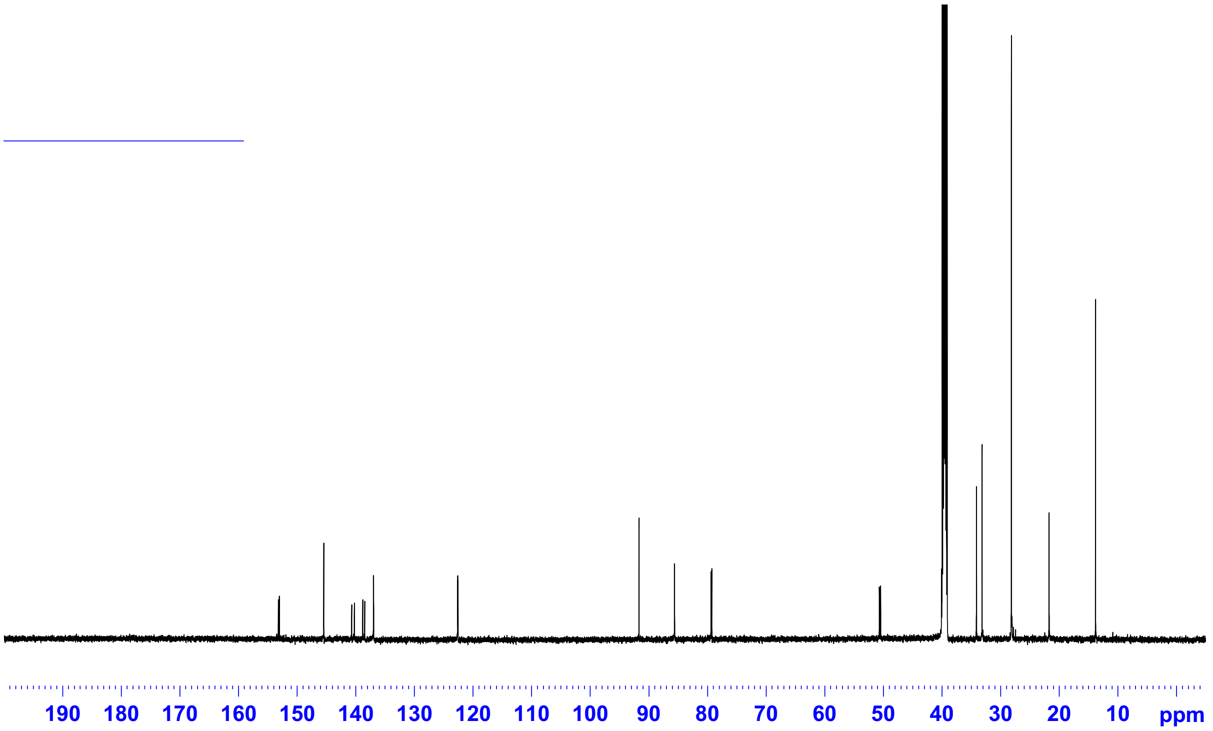

Supplement: Supplementary file 1 [file adsc0355-2353-sd1.pdf]
